# Development of an enolate alkynylation approach towards the synthesis of the taiwanschirin natural products†

**DOI:** 10.1039/d1sc04247e

**Published:** 2021-09-20

**Authors:** Maxwell B. Haughey, Kirsten E. Christensen, Darren L. Poole, Timothy J. Donohoe

**Affiliations:** Department of Chemistry, University of Oxford, Chemistry Research Laboratory Mansfield Road Oxford OX1 3TA UK timothy.donohoe@chem.ox.ac.uk; GlaxoSmithKline Medicines Research Centre Stevenage SG1 2NY UK

## Abstract

Through the use of model studies, an approach was conceived towards the synthesis of the taiwanschirin family of natural products. These are structurally complex compounds which represent highly challenging and biologically active targets for total synthesis. This work describes a successful synthesis of the complex taiwanschirin fused [8,6,5] core through a novel alkynylation reaction coupled with an intramolecular Heck reaction used to construct the 8-membered ring.

## Abstract 2

Key enolate alkynylation and ring closing Heck reactions have been used to make the core of the taiwanschirin natural products.

## Graphical abstract

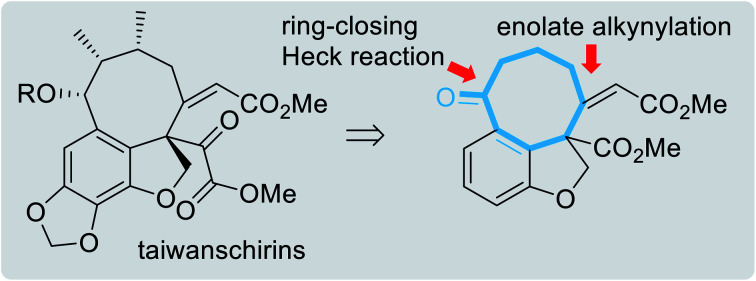

In nature, pyruvic acids and their derivatives are crucial to a myriad of metabolic pathways, including the synthesis of amino acids.^1^ Seminal work by Krebs and Johnson disclosed the central role of pyruvic acid in the citric acid (Krebs) cycle, responsible for the supply of energy to living cells through the aerobic oxidation of acetyl-CoA.^2^ In stark contrast to the ease by which Nature uses pyruvates and alpha-keto acid derivatives, there are few mild synthetic methods available for the manipulation of pyruvates. Accordingly, a recent program of research within our group has led to the development of novel methodologies for the alpha-arylation of oxabicyclo[2.2.2]octyl (OBO) *ortho*-ester protected pyruvate equivalents.^3^ Work by Johnson has also developed a complementary method for the alpha-arylation of *tert*-butyl pyruvates.^4^ Subsequent chemistry was later realised to assemble a wide variety of alpha-acyl substituted heterocycles,^3*b*^ resulting in the bioinspired syntheses of lamellarins D and Q in 7 steps from pyruvic acid.^3*c*^ This chemistry demonstrated the application of the pyruvate alpha-arylation methodology in a more complex setting. Our continued interest in masked pyruvate equivalents led us to identify the taiwanschirin family of natural products as suitable targets for total synthesis (Fig. 1).

**Fig. 1:**
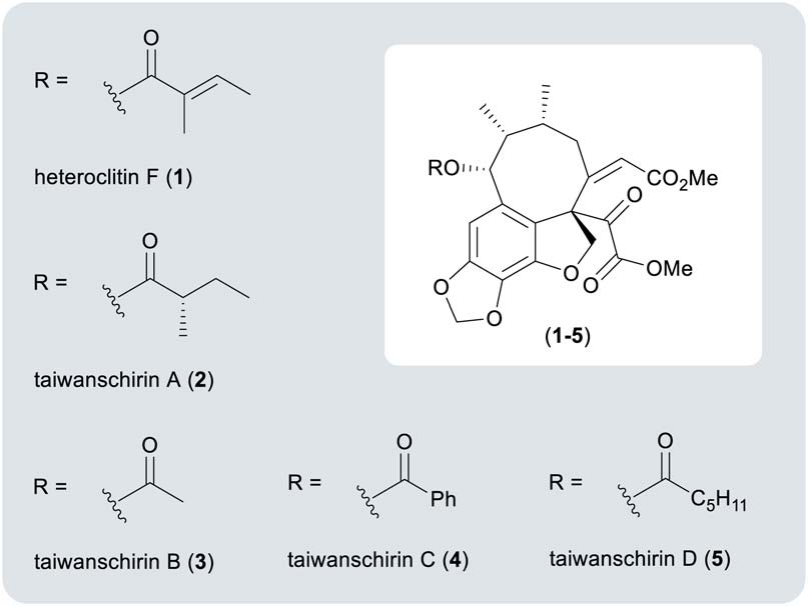
The taiwanschirin family of natural products.

Isolated from the stems of *Kadsura heteroclita*, heteroclitin F (**1**) was the first member of the taiwanschirin family to be discovered (Fig. 1).^5^ In 1999, the isolation of taiwanschirins A (**2**), B (**3**) and C (**4**) from *Schisandra arisanensis* was reported,^6^ and the final member of the family to be discovered was taiwanschirin D (**5**), isolated from *Kadsura matsudai*.^7^ The therapeutic properties of the *Schisandraceae* family have long been applied in traditional Chinese medicine.^8,9^ The stems of *Kadsura heteroclita* have been used for the treatment of many diseases and activity against human type B hepatitis has been demonstrated by taiwanschirins C (**4**) and D (**5**).^6,7^

The taiwanschirins are highly structurally complex natural products containing an extremely rare *ortho-peri*-fused [8,6,5] cyclooctabenzofuran skeleton, together with a fully substituted pyruvate unit. A synthesis of this class of natural product must overcome multiple synthetic challenges including formation of the thermodynamically unstable (*Z*)-enone and the *peri*-fused 8-membered ring featuring three contiguous stereogenic centres including one quaternary stereogenic centre. There is little literature precedent regarding approaches to these compounds,^10^ but during the preparation of this manuscript Lumb reported an elegant biomimetically inspired synthesis of several related lignan natural products.^11^ In this work a redox-neutral photocatalytic approach was used to assemble the key structural fragments.

Our retrosynthesis began with the acylation of alcohol **7** (Scheme 1) which would in turn be obtained by a diastereoselective reduction of ketone **8**.^12^ A double ozonolysis of alkene **9** (containing a sulphur ylide masked pyruvate) would result in concomitant formation of the ketone and pyruvate functionality. We decided to install the pyruvate group at a late-stage due to its general instability and sensitivity to chemical manipulations. Next, a key *exo*-selective Heck cyclisation would lead to the formation of the 8-membered ring, thereby forming the carbon skeleton. The precursor for the Heck cyclisation would be obtained from the conjugate addition of an organocuprate derived from iodide **12** into alkyne **11**. This alkyne intermediate was a key component of our retrosynthesis and we proposed that it could be synthesised *via* an alkynylation reaction of an enolate derived from cyanoketothiane **13** with beta-haloalkyne **14**.

**Scheme 1:**
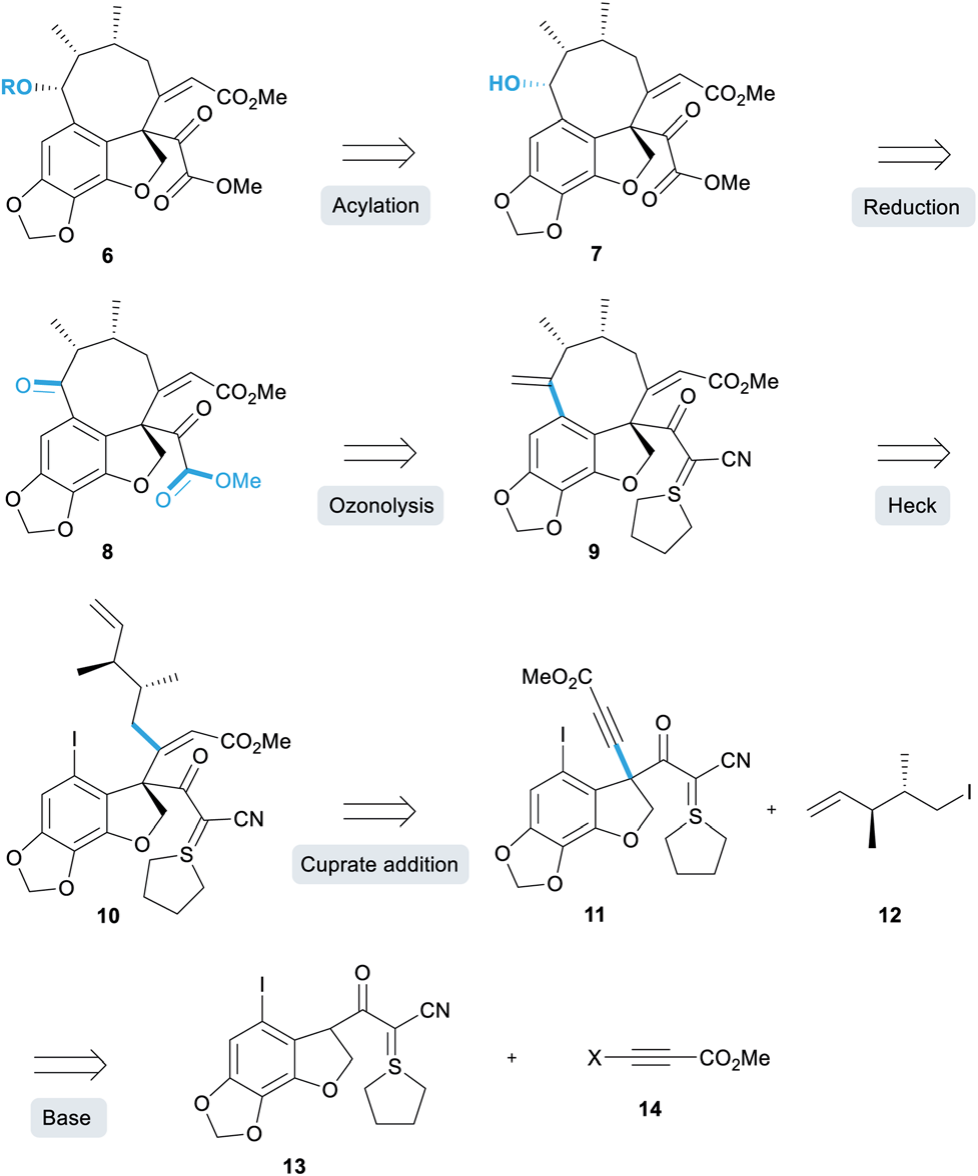
Proposed retrosynthesis.

Our inspiration for the alkynylation reaction derived from work by Jørgensen detailing the enantioselective alpha-alkynylation of cyclic beta-ketoesters.^13^ Here, beta-haloalkynes incorporating an electron withdrawing group underwent a conjugate addition–elimination reaction with cyclic beta-ketoesters under asymmetric phase-transfer conditions. Earlier workers have developed methodologies for the reaction of haloalkynes with ester enolates,^14^ and as partners in cycloaddition with alkenes.^15^ There are also reports of acetylenic substitution employing alternative classes of reagents, including notable work on hypervalent iodine reagents by Waser^16^ and lead triacetate reagents.^17^ To this end, we sought to further develop this general reaction to enable the reaction of haloacetylene **14** with enolates derived from a wide range of carbonyl compounds. We anticipated that a powerful alkynylation methodology could be developed that would not only enable the synthesis of alkyne **11**, and aid our synthesis of the taiwanschirins, but would also be highly useful in its own right.

Owing to the extended synthesis of cyanoketothiane **13**, we looked towards a model compound to test our synthetic strategy. Consequently, dihydrobenzofuran **17** was prepared in two steps from 2-hydroxybenzaldehyde **15** following a report by Hossain (Scheme 2a).^18^ Following this protocol, 2-hydroxybenzaldehyde **15** was reacted with ethyl diazoacetate and tetrafluoroboric acid. After treatment with concentrated sulfuric acid, benzofuran **16** was obtained in 85% yield. The reduction of benzofuran **16** using magnesium turnings in MeOH then afforded the model dihydrobenzofuran **17** in 70% yield.^19^ Transesterification of the ester from ethyl to methyl was also observed in this reaction.

**Scheme 2:**
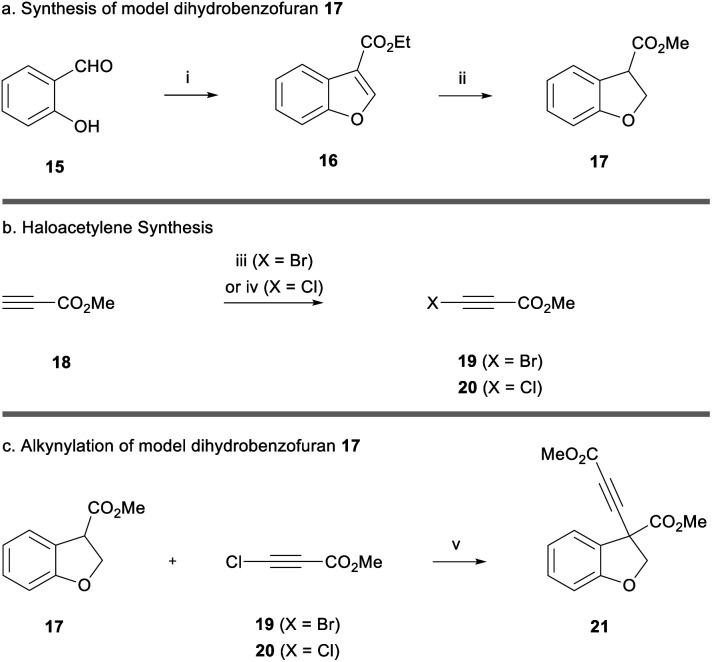
(a) Synthesis of model dihydrobenzofuran **17**. Reagents and conditions: (i) ethyl diazoacetate, HBF_4_·Et_2_O, CH_2_Cl_2_, rt, 30 min, then H_2_SO_4_, 30 min, 85%; (ii) Mg, MeOH, rt, 1 h, 70%. (b) Haloacetylene synthesis. Reagents and conditions: (iii) *N-*bromosuccinimide, acetone, rt, 30 min, 76%; (iv) ^*t*^BuOCl, ^*t*^BuOK, ^*t*^BuOH, rt, 30 min, 60%. (c) Alkynylation of model dihydrobenzofuran **17**. Reagents and conditions: (v) LiHMDS, THF, −78 °C, 5 min, then **19**, −78 °C, 30 min, 38%, or **20**, 94%.

Bromoacetylene **19** was prepared in 76% yield through reaction of *N*-bromosuccinimide with methylpropiolate (Scheme 2b).^20^ Chloroacetylene **20** was synthesised in 60% yield according to Jørgensen's procedure. We were now in a position to examine the alkynylation of dihydrobenzofuran **17**.^21^ Early work had shown that the enolate derived from **17** was only stable at low temperatures (*i.e.* −78 °C) because it underwent rapid beta-elimination and we observed by-products derived from opening of the heterocyclic ring. Deprotonation with weaker alkoxide bases was unsatisfactory, and thus we found it optimal to use a strong lithium amide base to deprotonate the ester at −78 °C. Pleasingly, after deprotonation with LiHMDS, dihydrobenzofuran **17** formed an enolate that went on to react with bromoacetylene **19** (reaction maintained at low temperature) to afford alkyne **21** in 38% yield (Scheme 2c). The use of chloroacetylene **20** gave a much cleaner reaction profile and a considerably higher yield of 94%. It has been suggested that the chloroalkyne electrophile is less likely to act as a halogenating agent towards the nucleophile, compared to the bromoalkyne, which may explain the significantly improved yield.^13*a*^

In addition to developing a general route to the taiwanschirins, we were keen to explore the general applicability of this dihydrobenzofuran enolate alkynylation methodology. Therefore, the scope of this reaction was demonstrated using a wide range of substituted acyl dihydrobenzofurans. A summary of the different products of this new reaction is presented below (**21–29**, Scheme 3). As shown in Scheme 3, the alkynylation reaction afforded good yields for ester substrates featuring electron donating groups including methylenedioxy (**22**-as found in the target natural products) and trimethylsilyl (**23**) affording the product alkynes in 68% and 69% yields respectively. The highest yields, however, were obtained from dihydrobenzofuran esters that were not substituted on the aromatic ring, for example esters **21** and **24** were obtained in 94% and 96% yields respectively. It was also found that the alkynylation reaction could be performed with *tert*-butyl chloroacetylene **30** to afford ester **25** and amide **26** in 100% and 85% yield.

**Scheme 3:**
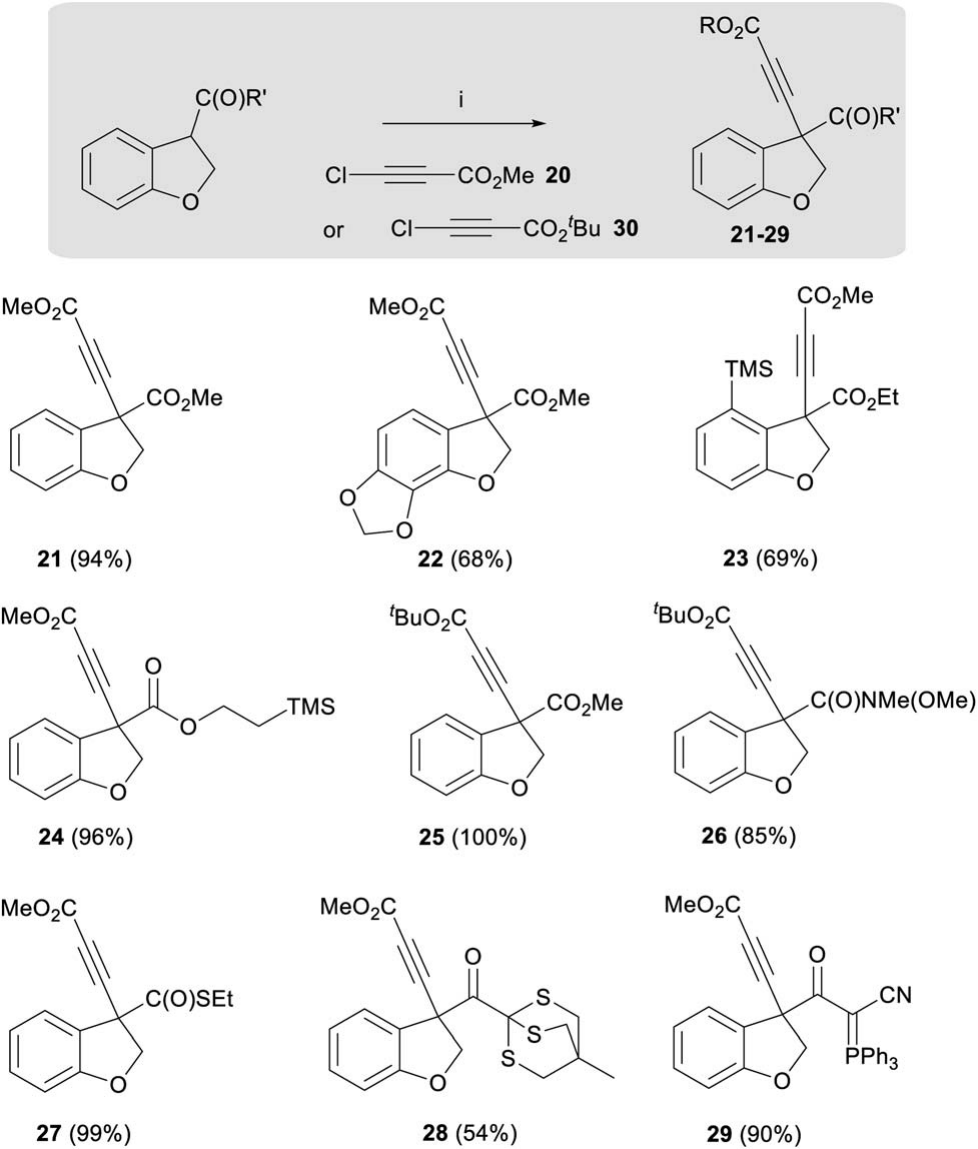
Alkynylation substrate scope. Reagents and conditions: (i) LiHMDS, THF, −78 °C, 5 min, then **20** or **30**, −78 °C, 30 min.

Notably, a thioester analogue was also found to display high reactivity, affording alkyne **27** in near quantitative yield. Further unprecedented reactivity was obtained with a thioether, leading to the isolation of alkyne **28** in 54% yield. The alkynylation reaction could also be applied to cyanoketophosphoranes enabling the synthesis of alkyne **29** in 90% yield.

The successful development of the alkynylation methodology meant that we could next explore methods to functionalise the alkyne leading to trisubstituted olefins as found in the target. The addition of organocuprate reagents to ynones has been described as a method of accessing trisubstituted enones with overall *cis*-addition.^22^ Pleasingly, we found that the organocuprate reagent derived from MeLi reacted with alkyne **21** to afford the desired (*Z*)-enone **31** exclusively as a single diastereoisomer in 92% yield (Scheme 4). This process was shown to be general, and organocuprate reagents derived from various organolithium reagents were tolerated, for example ^*n*^BuLi and ^*i*^BuLi afforded enones **32** and **33** in yields of 89% and 91% respectively.

**Scheme 4:**
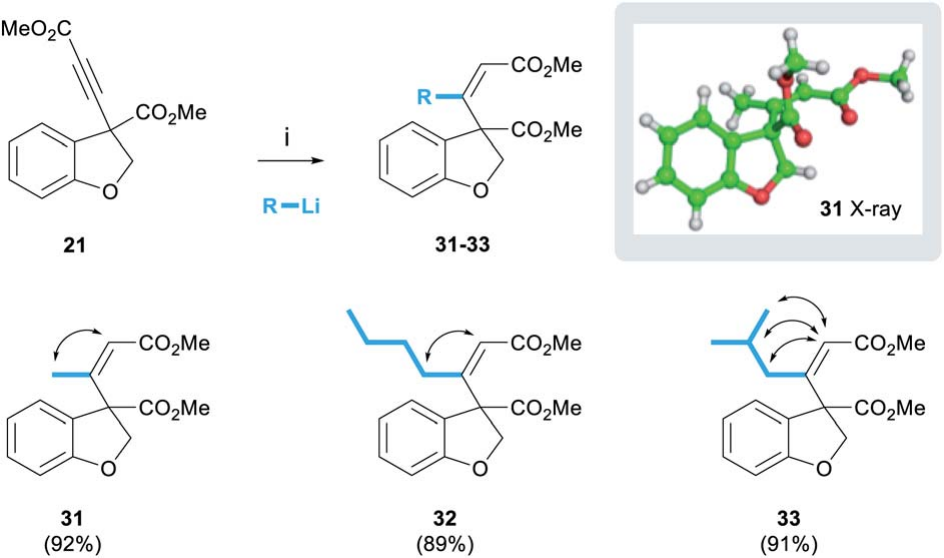
(*Z*)-Enone incorporation. Reagents and conditions: (i) 4 eq. RLi, 2 eq. CuI, THF, −78–0 °C, 1 h, then −78 °C, 21, 30 min.

The alkene stereochemistry of these products was proven unequivocally. NOESY spectra were obtained for the three enones below (cross-peaks represented by double-headed arrows) and the (*Z*)-configuration was proven in each case (see ESI† for spectra). Furthermore, a crystal of enone **31** was grown and the structure proven by single crystal X-ray analysis.^23^ We also observed that the ^1^H NMR chemical shift of the olefinic proton was diagnostic of enone stereochemistry. In the case of the desired (*Z*)-enones, the olefinic proton was typically in the range of 5.86–5.93 ppm. However, in preliminary work using the same procedure but quenching the reaction with aqueous NH_4_Cl, rather than MeOH, we isolated quantities (approximately 20%) of the undesired (*E*)-enone whose olefinic protons appeared in a lower range of 5.62–5.63 ppm (see ESI†). We speculate that the reasons behind this difference in selectivity lie in the rate of reaction quenching *versus* temperature as the reaction warms to room temperature.

Next, we turned our attention towards the fused 8-membered ring of the taiwanschirin target. In order to test the intramolecular Heck cyclisation approach, it was necessary to incorporate a halogen functional group at the *peri*-position of the benzofuran. To this end, 3-bromophenol **34** was deprotonated with NaH and reacted with diethylcarbamoyl chloride to form a carbamate (Scheme 5). With this protecting group in place we performed a directed deprotonation using LDA in THF at −78 °C. Following reaction with DMF and subsequent deprotection under acidic conditions, aldehyde **36** was formed in 69% yield over 3 steps. As before, aldehyde **36** was then reacted with ethyl diazoacetate to afford *peri*-bromobenzofuran **37** in 84% yield.

**Scheme 5:**
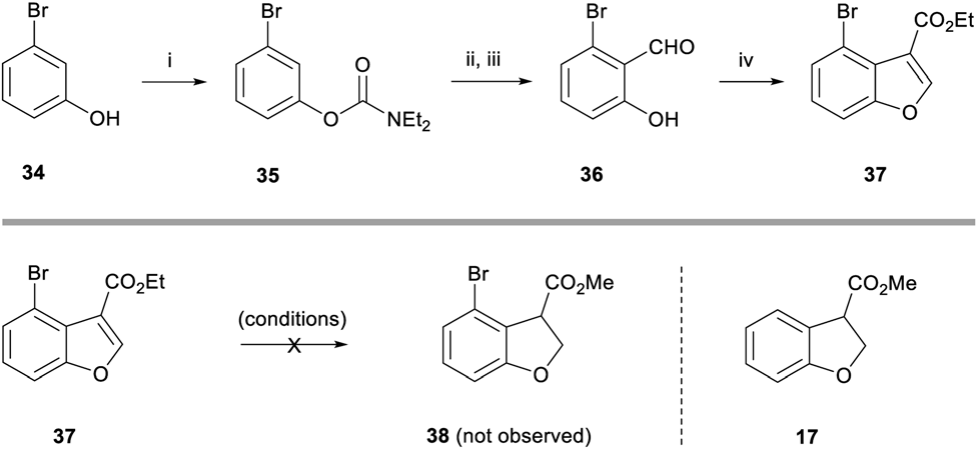
Synthesis of benzofuran **37** and attempted reduction. Reagents and conditions: (i) NaH, diethylcarbamoyl chloride, THF, rt, 16 h; (ii) LDA, THF, −78 °C, 30 min, then DMF, −78 °C, 30 min; (iii) 2 M HCl (aq.), 0 °C, 20 min, 69% over 3 steps; (iv) ethyl diazoacetate, HBF_4_·OEt_2_, rt, 30 min, then H_2_SO_4_, rt, 30 min, 84%.

We then subjected benzofuran **37** to a range of reducing conditions in order to form the corresponding dihydrobenzofuran **38**. Disappointingly, the magnesium-promoted reduction conditions used previously were unsuccessful (Scheme 5). Dihydrobenzofuran **38** was not observed in the reaction mixture by ^1^H NMR spectroscopy, however the de-brominated dihydrobenzofuran **17** was isolated in 23% yield from a complex mixture. Conducting the reaction at 0 °C resulted in only starting material being observed by ^1^H NMR spectroscopy.

A variety of different conditions for the partial reduction of benzofuran **37** were then investigated as detailed below. Surprisingly, the use of palladium-catalysed hydrogenation conditions failed to afford the desired product; the de-brominated benzofuran was not observed.^24^ Both triethylsilane/trifluoracetic acid (TFA),^25^ and samarium(ii) iodide also resulted in only starting material being observed by ^1^H NMR spectroscopy of the crude reaction mixtures.^26^

In order to overcome this problem, a palladium-catalysed stannylation reaction was proposed to convert the bromobenzofuran **37** to the corresponding tributylstannane **40**. We postulated that the aryl stannane may be reduced to the dihydrobenzofuran under the magnesium-promoted reduction, and the stannane then exchanged for a halogen, thereby enabling the key intramolecular Heck cyclisation. Initially, only starting material was observed when Pd(PPh_3_)_4_ was used as the catalyst for the stannylation of **37** (Scheme 6, entry 1).^27^ However, a change of catalyst to PdCl_2_(PhCN)_2_, resulted in a 33% yield of the desired product (entry 2).^28^ Pleasingly, the use of 2.5 mol% Pd_2_(dba)_3_ in combination with 15 mol% JohnPhos resulted in 65% yield (entry 3).

**Scheme 6:**
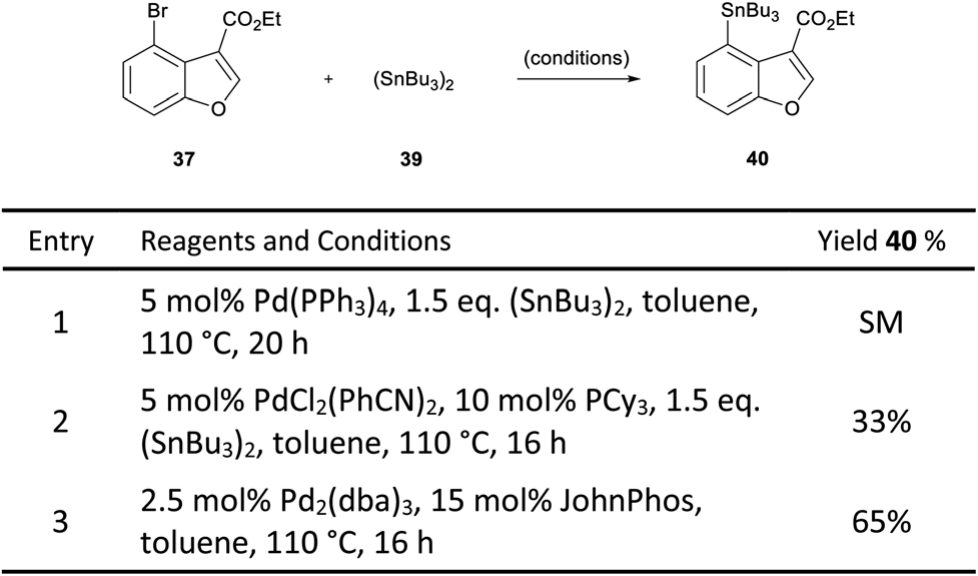
Stannylation of benzofuran **37**.

With benzofuran **40** in hand, we were able to test the magnesium-promoted reduction step and observed the formation of stannane **41** in 92% yield (Scheme 7). Furthermore, a comparable yield of 84% was achieved when the reaction was performed on a 1.6 g scale. Crucially, heating at 70 °C was necessary for complete transesterification to form the methyl ester.

**Scheme 7:**
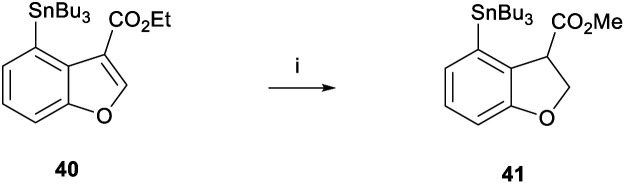
Magnesium reduction of benzofuran **40**. Reagents and conditions: (i) Mg, MeOH, rt, 4 h then 70 °C, 1 h, 92%.

Stannane **41** was then subjected to the newly-developed alkynylation reaction conditions to form alkyne **42** in 48% yield (Scheme 8). Tin-iodine exchange transformed stannane **42** to iodide **43** in 87% yield. The following cuprate addition also progressed cleanly to afford alkene **45** in 45% yield as the single (desired) (*Z*)-diastereomer. This assignment was made by analogy with the previous examples (Scheme 4); additional data included the presence of the olefinic proton resonance at 5.90 ppm, comfortably within the range found for (*Z*)-enones of 5.86–5.93 ppm.

**Scheme 8:**
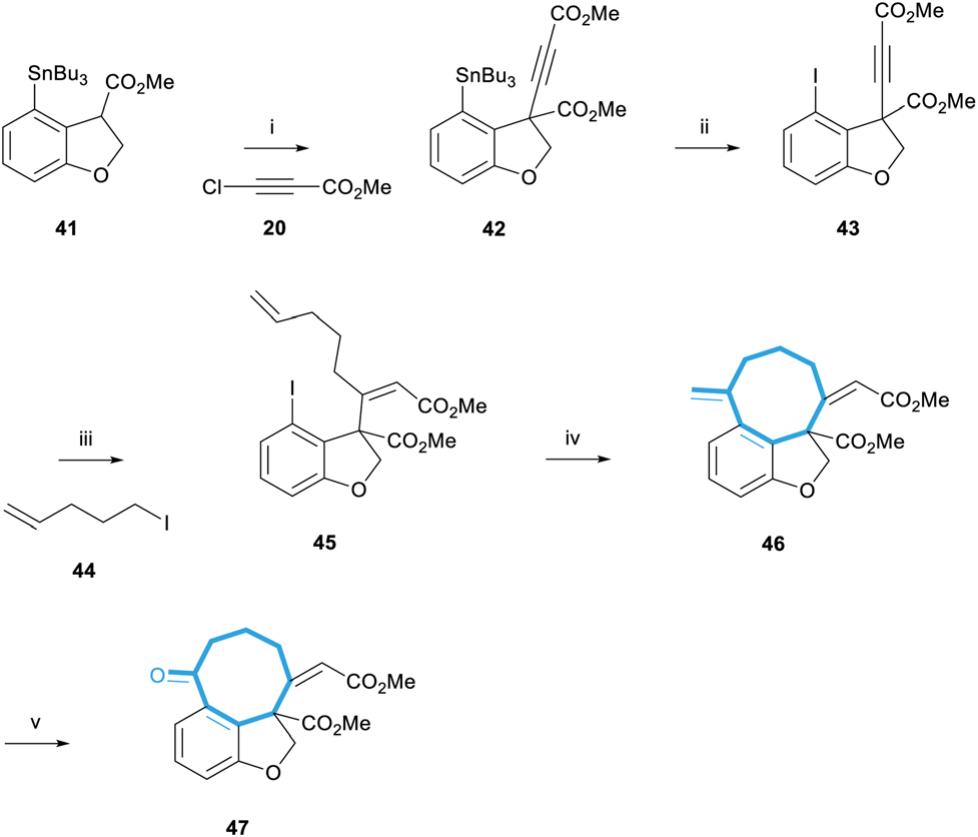
Synthesis of iodide **45** and subsequent Heck cyclisation and dihydroxylation-periodate cleavage to afford alkene **47**. Reagents and conditions: (i) LiHMDS, **41**, THF, −78 °C, 10 min, then **20**, −78 °C, 1 h, 48%; (ii) I_2_, CHCl_3_, rt, 1 h, 87%; (iii) **44**, ^*t*^BuLi, pentane/Et_2_O 3 : 2, −78 °C, 10 min, CuI, then 0 °C, 1 h, then −78 °C, **43**, 30 min, 45%; (iv) 10 mol% Pd(OAc)_2_, 40 mol% PPh_3_, 10 eq. Et_3_N, MeCN, 80 °C, 1 h, 81%; (v) 5 mol% OsO_4_, 5 eq. NaIO_4_, THF/H_2_O 2 : 1, rt, 2 h, 69%.

With the pendant alkene installed, we could now test the key Heck-cyclisation reaction. We were delighted to observe that a slight modification of literature conditions resulted in an 81% yield of the desired alkene **46** (Scheme 8).^29^ Furthermore, the reaction was regioselective, affording the 8-*exo*-regioisomer exclusively, and the undesired 9-*endo*-cyclisation product was not observed in the ^1^H NMR spectrum of the crude reaction mixture. The selectivity of this reaction is noteworthy given previous reports detailing the low selectivity of intramolecular Heck cyclisation reactions during the formation of medium-sized rings.^30*a*^ We note that in this reaction, the tether between the aryl iodide and the alkene contains a (*Z*)-alkene, 3 trigonal carbons and a single quaternary-substituted carbon. Thus, we suggest that the high *exo*-selectivity of this reaction is a consequence of having a relatively restricted and sterically encumbered framework which is unable to accommodate the larger steric requirements of an endo cyclication to form a 9-membered ring.^30*b*,*c*^

Finally, alkene **46** was reacted with catalytic OsO_4_ in the presence of NaIO_4_.^31^ This dihydroxylation-periodate cleavage reaction afforded ketone **47** in 69% yield, representing the most advanced compound en-route to the taiwanschirin family synthesised. Overall, ketone **47** was synthesised in 4% yield over 11 steps from 3-bromophenol **34**. With the carbon skeleton of the taiwanschirin family now nearly complete, the final challenge to be solved was incorporation of the pyruvate group.

Installing the pyruvate proved to be the most challenging part of this project. A wide range of strategies were explored before we examined the use of the Bode salt **49** as a pyruvate equivalent, itself prepared in a single step from tetrahydrothiophene and bromoacetonitrile (Scheme 9).^32^ Dihydrobenzofuran **17** was subjected to ester hydrolysis conditions to afford carboxylic acid **48**. Pleasingly, the reaction of **48** and **49** in the presence of EDC·HCl provided cyanoketothiane **50** in 78% yield. Cyanoketothiane **50** was subsequently deprotonated with LDA and reacted with chloroacetylene **20** to afford alkyne **51** in 60% yield. Here, LDA base was found to afford optimum yields of the desired product. The addition of an organocuprate reagent derived from ^*i*^BuLi to alkyne **51** afforded (*Z*)-enone **52** in 79% yield. The stereochemical assignment of **52** was made by analogy with the previous examples of this reaction (Scheme 4) and the ^1^H NMR chemical shift of the olefinic proton of alkene **52** was observed at 5.90 ppm. We were delighted to observe that enone **52** reacted with ozone at −78 °C in the presence of methanol, to form the desired pyruvate **53** in 34% yield.^33^ The crude ^1^H NMR spectrum of this reaction showed complete conversion of enone **52** to pyruvate **53**, with almost no other contaminants; the modest yield was attributed to the small scale of this model reaction.

**Scheme 9:**
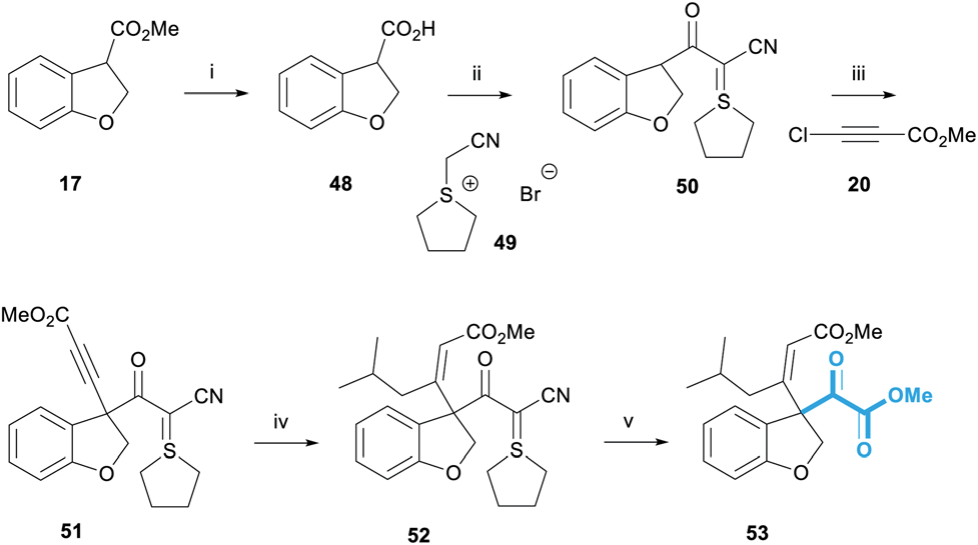
Pyruvate incorporation. Reagents and conditions: (i) Na_2_CO_3_, THF/H_2_O 1 : 1, rt, 16 h, 54% (ii) EDC·HCl, DIPEA, DMAP, CH_2_Cl_2_, rt, 16 h, 78%; (iii) LDA, **50**, THF, −78 °C, 10 min, then **20**, −78 °C, 30 min, 60%; (iv) ^*i*^BuLi, CuI, THF, −78–0 °C, 1 h, then −78 °C, **51**, 5 min, 79%; (v) O_3_, CH_2_Cl_2_/MeOH 4 : 1, −78 °C, 10 min, 34%.

Thus, we have demonstrated a comprehensive approach towards the taiwanschirin family of natural products that incorporate the key structural fragments identified at the outset. Future work will combine these elements to complete the synthesis of these targets and also address the issue of an asymmetric alkynylation reaction using controlling factors such as chiral auxiliaries, chiral lithium amides or asymmetric catalysis.^34^

## Conclusions

This work describes model studies developed towards the structural core of the taiwanschirin family of natural products. An approach was conceived to enable installation of the (*Z*)-enone as a single diastereoisomer. A novel alkynylation reaction has been successfully applied to a wide substrate scope including esters, amides, ketones and thioesters. Furthermore, these studies have led to the assembly of an 8-membered ring in high yield through the development of a regioselective Heck cross-coupling reaction. Under palladium-catalysed conditions, we observed the formation of the desired alkene as a single (*exo*)-regioisomer in 81% yield. Through the use of Bode salt, the pyruvate functional group could also be accessed, and this approach enabled the assembly of a model compound that incorporated both the pyruvate group and the (*Z*)-enone.

## Data availability

Full experimental and characterisation data are provided as part of the ESI.†

## Author contributions

MBH performed the experiments. KEC performed the X-ray crystallography experiments. All authors contributed to the discussion and prepared the manuscript. TJD directed the project.

## Conflicts of interest

There are no conflicts to declare.

## Supplementary Material

SC-012-D1SC04247E-s001

SC-012-D1SC04247E-s002
