# Supplementary material for: Development of an enolate alkynylation approach towards the synthesis of the taiwanschirin natural products
Source: Chem Sci. 2021 Sep 20;12(40):13392–7. doi: 10.1039/d1sc04247e (PMC8528008; doi:10.1039/d1sc04247e)
Supplement: SC-012-D1SC04247E-s001 [file SC-012-D1SC04247E-s001.pdf]

Electronic Supplementary Material (ESI) for Chemical Science.
This journal is © The Royal Society of Chemistry 2021

Development of an Enolate Alkynylation Approach Towards the Synthesis of the Taiwanschirin Natural Products

Maxwell B. Haughey[a], Kirsten E. Christensen[a†], Darren L. Poole[b], and Timothy J. Donohoe[*a]

[a]*Department of Chemistry, University of Oxford, Chemistry Research Laboratory, Mansfield Road, Oxford, OX1 3TA, UK.*

[b]*GlaxoSmithKline Medicines Research Centre, Gunnels Wood Road, Stevenage, SG1 2NY, UK.*

[†]*Chemical Crystallography, Chemistry Research Laboratory, University of Oxford.*

Supporting Information

Contents

1.1 Experimental techniques S2

1.2 Experimental procedures S4

1.3 NMR spectra S46

1.4 NOESY spectra of enone **31**, **32**, **33**, **74**, and **75** S101

1.5 Supplementary CIF data for enone **31** S106

1.6 References S117

1.1 Experimental Techniques

General: All glassware was flame–dried under vacuum and reactions carried out under argon atmosphere unless otherwise noted. Compounds were named based on IUPAC guidelines using ChemDraw software and atom numbering may not be consistent with this name. This is for clarity in assignment and consistency between different classes of molecules.

Solvents and reagents: CH_2Cl_2, THF, toluene, Et_2O, acetonitrile and MeOH were dried by filtration through an activated alumina purification column under a positive pressure of nitrogen. When required, CH_2Cl_2 was degassed by bubbling an inert gas (argon or nitrogen) through the solvent for 15–30 min. All other solvents and reagents requiring purification were purified using standard laboratory techniques. Reagents obtained from commercial sources (Acros Organics, Alfa Aesar, Apollo Scientific, Fluorochem, Sigma Aldrich, TCI) were used as supplied unless otherwise stated.

Chromatography: Flash column chromatography (FCC) was performed using Merck Geduran silica gel 60 (40–63 µm). The solvent systems employed are quoted in parentheses. Reactions and column chromatography were monitored by thin layer chromatography (TLC) analysis using Merck Kieselgel 60 F254 0.25 mm precoated aluminium backed plates. Product spots were visualised under UV light (λ_{max} = 254 nm) and stained with basic potassium permanganate solution, phosphomolybdic acid solution or acidic vanillin solution as deemed appropriate for the compound.

NMR spectroscopy: ^{1}H and ^{13}C nuclear magnetic resonance spectra (NMR) were recorded on Bruker spectrometers at 400 MHz or 500 MHz (for ^{1}H NMR), at 101 MHz or 126 MHz (for ^{13}C NMR), at 377 MHz or 471 MHz (for ^{19}F NMR), and at 126 MHz (for ^{31}P NMR) as specified. Chemical shifts (δ) are reported relative to residual protic solvent peaks or tetramethylsilane (TMS) internal standard and quoted in parts per million (ppm) to the nearest 0.01 ppm for ^{1}H NMR and 0.1 ppm for ^{13}C NMR, ^{19}F NMR and ^{31}P NMR. ^{19}F NMR and ^{31}P NMR spectra were not externally referenced. Coupling constants (*J*) are quoted in Hertz (Hz) to the nearest 0.1 Hz for ^{1}H NMR. Signal splittings are recorded as singlet (s), doublet (d), triplet (t), quartet (q), septet (sept), multiplet (m). Structural assignments were based on COSY, HSQC, HMBC, DEPT, and NOESY experiments.

Mass spectrometry: High resolution mass spectra (HRMS) under electrospray ionisation (ESI) or field ionisation (FI) were recorded on a Bruker MicroTof or a Thermo Exactive orbitrap spectrometer. HRMS under chemical ionisation (CI) or electron ionisation (EI)

conditions were recorded on a Waters LCT Premier or an Agilent 7200 quadrupole time of flight (Q–ToF) spectrometer. HRMS under matrix assisted laser desorption/ionisation (MALDI) were recorded on a Waters MALDI micro MX spectrometer. Masses are reported as a ratio of mass to charge in Daltons and are given to four decimal places.

Infrared spectroscopy: Fourier transformation infrared spectra (FTIR) were recorded on a Bruker Tensor 27 FT–IR spectrometer equipped with Attenuated Total Reflectance sampling accessory as evaporated films or powders. Absorption maxima (ν_{max}) are reported in wavenumbers (cm^{-1}).

Polarimetry: Specific optical rotations were recorded on a Schmidt–Haensch UniPol 2000 polarimeter at the sodium D line (λ = 589.3 nm) in $CHCl_3$ and are quoted in the units of 10^{-1} deg cm^2 g^{-1}. Solution concentrations are given in units of 10^{-2} g mL^{-1}.

Melting points: Melting points (m.p.) were obtained using a Leica VMTG heated stage microscope and are uncorrected.

1.2 Experimental Procedures

Ethyl benzofuran-3-carboxylate (16)[1]

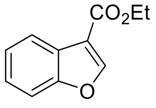

16

To a solution of salicylaldehyde (6.10 g, 50.0 mmol) and $HBF_4·OEt_2$ (680 µL, 5.00 mmol) in CH_2Cl_2 (12 mL) at 0 °C was added dropwise a solution of ethyl diazoacetate (8.42 mL, 80 mmol) in CH_2Cl_2 (80 mL) over a period of 10 min. The reaction mixture was stirred at room temperature for 30 min and then concentrated *in vacuo.* After nitrogen evolution had ceased, conc. H_2SO_4 (3.00 mL) was added and the reaction mixture was stirred at room temperature for 30 min. CH_2Cl_2 (100 mL) was added followed by solid $NaHCO_3$ until gas evolution had ceased. The solution was washed successively with water (50 mL), 1 M aq. NaOH (50 mL) and sat. aq. NaCl (50 mL), dried over $MgSO_4$ and concentrated *in vacuo*. Purification by flash column chromatography (SiO_2, Pentane:Et_2O, 90:10) afforded the title compound as a colourless oil (8.05 g, 85%).

$\boldsymbol{R_f}$ = 0.2 (Pentane:Et_2O, 90:10).
^{1}H NMR ($CDCl_3$, 400 MHz) δ = 8.26 (1H, s, Ar*H*), 8.07 (1H, ddd, *J* = 6.0, 3.2, 0.7 Hz, Ar*H*), 7.60 – 7.47 (1H, m, Ar*H*), 7.42 – 7.30 (2H, m, Ar*H* × 2), 4.41 (2H, q, *J* = 7.1 Hz, $CO_2CH_2CH_3$), 1.43 (3H, t, *J* = 7.1 Hz, $CO_2CH_2CH_3$).
^{13}C NMR ($CDCl_3$, 101 MHz) δ = 163.5 ($CO_2CH_2CH_3$), 155.7 (C_{Ar}), 151.0 (C_{Ar}H), 125.3 (C_{Ar}H), 124.7 (C_{Ar}), 124.2 (C_{Ar}H), 122.1 (C_{Ar}H), 114.8 (C_{Ar}), 111.8 (C_{Ar}H), 60.7 ($CO_2CH_2CH_3$), 14.5 ($CO_2CH_2CH_3$).
Analytical data are in accordance with those previously reported for this compound.[1]

Methyl 2,3-dihydrobenzofuran-3-carboxylate (17)[2]

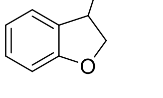

17

Mg turnings (1.96 g, 80.7 mmol) were added to a solution of ester **16** (3.07 g, 16.1 mmol) in MeOH (107 mL). After 1 h the reaction mixture was evaporated to dryness and re–dissolved in CH_2Cl_2 (200 mL). The solution was washed successively with sat. aq. NH_4Cl (100 mL), sat.

aq. NaCl (50 mL), dried over $MgSO_4$ and concentrated *in vacuo*. Purification by flash column chromatography (SiO_2, Pentane:Et_2O, 90:10) afforded the title compound as a colourless oil (2.01 g, 70%).

R_f = 0.25 (Pentane:Et_2O, 90:10).
^{1}H NMR ($CDCl_3$, 400 MHz) δ = 7.38 (1H, dt, *J* = 7.4, 1.3 Hz, Ar*H*), 7.23 – 7.14 (1H, m, Ar*H*), 6.89 (1H, td, *J* = 7.5, 1.0 Hz, Ar*H*), 6.86 – 6.80 (1H, m, Ar*H*), 4.93 (1H, dd, *J* = 9.2, 6.6 Hz, CH_AH_B), 4.66 (1H, t, *J* = 9.2 Hz, CH_AH_B), 4.34 (1H, dd, *J* = 9.7, 6.5 Hz, C*H*), 3.78 (3H, s, CO_2CH_3).
^{13}C NMR ($CDCl_3$, 101 MHz) δ = 171.7 (CO_2CH_3), 159.9 (C_{Ar}), 129.5 (C_{Ar}H), 125.4 (C_{Ar}H), 124.2 (C_{Ar}), 120.7 (C_{Ar}H), 110.0 (C_{Ar}H), 72.5 (CH_AH_B), 52.6 (CO_2CH_3), 47.2 (*C*H).
Analytical data are in accordance with those previously reported for this compound.[2]

Methyl 3-bromopropiolate (19)[3]

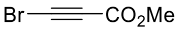

19

To a solution of *N*-bromosuccinimide (4.89 g, 27.5 mmol) in acetone (80 mL) was added methyl propiolate (2.22 mL, 25.0 mmol). The reaction mixture was stirred for 30 min and the solvent removed *in vacuo*. The residue was re–dissolved in Et_2O (100 mL) and filtered through Celite®. Distillation of the residue (bp 28 °C, 5 mbar) afforded the title compound as a colourless oil (3.12 g, 76%).

R_f = 0.5 (Pentane:Et_2O, 90:10).
^{1}H NMR ($CDCl_3$, 400 MHz) δ = 3.76 (3H, s, CO_2CH_3).
^{13}C NMR ($CDCl_3$, 101 MHz) δ = 152.9 (CO_2CH_3), 72.6 ($C\equiv CBr$), 53.1 ($C\equiv CBr$), 53.0 (CO_2CH_3).
Analytical data are in accordance with those previously reported for this compound.[3]

Methyl 3-chloropropiolate (20)[4]

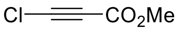

20

To a solution of methyl propiolate (10.2 mL, 115 mmol) and ^{t}BuOCl (12.4 g, 115 mmol) in ^{t}BuOH (20 mL) was added portion–wise ^{t}BuOK (1.28 mg, 11.5 mmol). The reaction mixture

was stirred at rt for 30 min, then water (30 mL) and pentane (50 mL) was added. The two layers were separated and the organic layer washed with brine (20 mL), dried over Mg_2SO_4 and concentrated *in vacuo*. Purification by flash column chromatography (SiO_2, Pentane:Et_2O, 95:5) afforded the title compound as a colourless oil (8.10 g, 60%).

***R*f** = 0.5 (Pentane:Et_2O, 90:10).
^{1}H NMR ($CDCl_3$, 400 MHz) δ = 3.78 (3H, s, CO_2CH_3).
^{13}C NMR ($CDCl_3$, 101 MHz) δ = 152.9 (CO_2CH_3), 68.7 (*C*≡CCl), 61.8 (C≡*C*Cl), 53.2 (CO_2CH_3).
Analytical data are in accordance with those previously reported for this compound.[5]

Methyl 3-(3-methoxy-3-oxoprop-1-yn-1-yl)-2,3-dihydrobenzofuran-3-carboxylate (21)

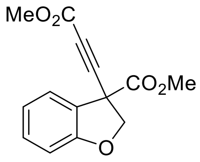

21

To a stirred solution of ester **17** (261 mg, 1.46 mmol) in THF (13 mL) at –78 °C was added LiHMDS (1.75 mL, 1.75 mmol, 1 M in THF). After 10 min chloroacetylene **20** (260 mg, 2.19 mmol) in THF (500 µL) was added dropwise and the reaction mixture was stirred at –78 °C for 1 h. Sat. aq. NH_4Cl (3 mL) was added and the mixture extracted with Et_2O (3 × 20 mL). The combined organic phase was washed with sat. aq. NaCl (20 mL), dried over $MgSO_4$ and concentrated *in vacuo*. Purification by flash column chromatography (SiO_2, Pentane:Et_2O, 85:15) afforded the title compound as a colourless oil (357 mg, 94%).

***R*f** = 0.3 (Pentane:Et_2O, 70:30).
^{1}H NMR ($CDCl_3$, 400 MHz) δ = 7.46 (1H, dd, *J* = 7.6, 1.4 Hz, Ar*H*), 7.29 – 7.23 (1H, m, Ar*H*), 6.96 (1H, td, *J* = 7.6, 1.0 Hz, Ar*H*), 6.86 (1H, d, *J* = 8.2 Hz, Ar*H*), 5.22 (1H, d, *J* = 9.2 Hz, CH_AH_B), 4.71 (1H, d, *J* = 9.2 Hz, CH_AH_B), 3.83 (3H, s, CO_2CH_3), 3.77 (3H, s, CO_2CH_3).
^{13}C NMR ($CDCl_3$, 126 MHz) δ = 168.4 (CO_2CH_3), 159.4 (C_{Ar}), 153.6 (CO_2CH_3), 131.0 (C_{Ar}H), 125.2 (C_{Ar}H), 125.2 (C_{Ar}), 121.7 (C_{Ar}H), 110.9 (C_{Ar}H), 84.4 (*C*≡CCO_2CH_3), 79.1 (CH_AH_B), 75.8 (C≡*C*CO_2CH_3), 54.2 ($C_{Ar}C$), 53.1 (CO_2CH_3), 51.1 (C≡CCO_2CH_3).
IR (film) ν_{max}/cm^{-1} 2956, 2243, 1746, 1717, 1597, 1480, 1281, 1236, 750.
HRMS: (APCI$^+$) Found $[M + H]^+$ = 261.0756, $C_{14}H_{13}O_5$ requires 261.0758.

Methyl 6-(3-methoxy-3-oxoprop-1-yn-1-yl)-6,7-dihydro-[1,3]dioxolo[4,5-*g*]benzofuran-6-carboxylate (22)

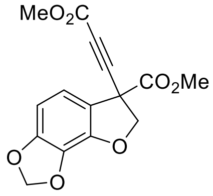

22

To a stirred solution of ester **59*** (67 mg, 302 µmol) in THF (2.1 mL) at –78 °C was added LiHMDS (360 µL, 360 µmol, 1 M in THF). After 10 min, chloroacetylene **20** (54 mg, 450 µmol) in THF (500 µL) was added dropwise and the reaction mixture was stirred at –78 °C for 1 h. Sat. aq. NH_4Cl (3 mL) was added and the mixture extracted with Et_2O (3 × 20 mL). The combined organic phase was washed with sat. aq. NaCl (20 mL), dried over $MgSO_4$ and concentrated *in vacuo*. Purification by flash column chromatography (SiO_2, Pentane:Et_2O, 80:20) afforded the title compound as a colourless oil (53 mg, 68%).

R R_f = 0.3 (Pentane:Et_2O, 70:30).

^{1}H NMR ($CDCl_3$, 400 MHz) δ = 6.95 (1H, d, *J* = 8.1 Hz, Ar*H*), 6.49 (1H, d, *J* = 8.1 Hz, Ar*H*), 5.98 (1H, d, *J* = 1.5 Hz, OCH_AH_BO), 5.96 (1H, d, *J* = 1.4 Hz, OCH_AH_BO), 5.26 (1H, d, *J* = 9.2 Hz, CH_AH_B), 4.76 (1H, d, *J* = 9.2 Hz, CH_AH_B), 3.82 (3H, s, CO_2CH_3), 3.77 (3H, s, CO_2CH_3).

^{13}C NMR ($CDCl_3$, 101 MHz) δ = 168.3 (CO_2CH_3), 153.6 (CO_2CH_3), 151.1 (C_{Ar}), 142.2 (C_{Ar}), 130.8 (C_{Ar}), 121.6 (C_{Ar}), 117.8 (C_{Ar}H), 102.6 (C_{Ar}H), 102.2 (OCH_AH_BO), 84.1 (C≡CCO_2CH_3), 80.9 (CH_AH_B), 75.8 (C≡CCO_2CH_3), 54.2 ($C_{Ar}C$), 53.1 (CO_2CH_3), 50.8 (CO_2CH_3).

IR (film) ν_{max}/cm^{-1} 3032, 1747, 1717, 1653, 1472, 1250, 1068.

HRMS: (APCI$^+$) Found $[M + H]^+$ = 306.0659, $C_{15}H_{13}O_7$ requires 305.0656.

*The starting material used to perform this alkynylation reaction was synthesised according to the procedures detailed below:

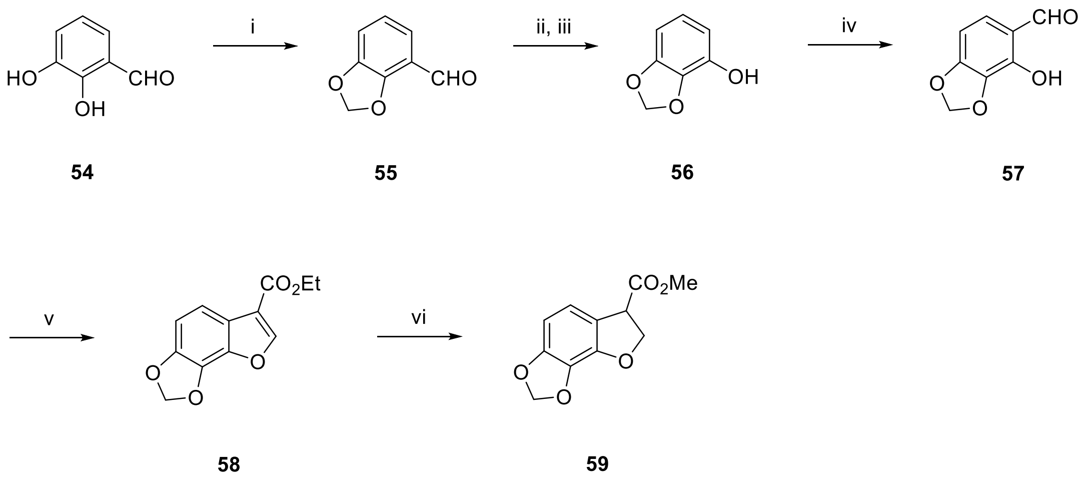

Scheme 1. Reagents and conditions: i) CH_2Br_2, K_2CO_3, 10 mol% CuO, DMF, 165 °C, 3 h, 100%, ii) MCPBA, CH_2Cl_2, 40 °C, 16 h, iii) KOH, EtOH, rt, 2 h, 64% over 2 steps, iv) paraformaldehyde, $MgCl_2$, Et_3N, THF, 85 °C, 6 h, 95%, v) Ethyl diazoacetate, $HBF_4{\cdot}OEt_2$, rt, 30 min, then H_2SO_4, rt, 30 min, 90%, vi) Mg, MeOH, rt, 1 h 82%

Benzo[*d*][1,3]dioxole-4-carbaldehyde (55)[14]

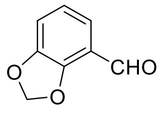

55

A solution of 2,3-dihydroxybenzaldehyde **54** (5.00 g, 36.2 mmol), CuO (288 mg, 3.62 mmol), K_2CO_3 (5.50 g, 39.8 mmol) and CH_2Br_2 (3.05 mL, 43.4 mmol) in DMF (40 mL) was heated at 165 °C for 3 h. The reaction mixture was filtered through Celite® and washed with EtOAc (150 mL). The filtrate was washed with sat. aq. LiCl (100 mL), sat. aq. K_2CO_3 (100 mL), dried over $MgSO_4$ and concentrated *in vacuo*. Purification by flash column chromatography (SiO_2, Pentane:Et_2O, 93:7 to 80:20) afforded the title compound as a pale yellow oil (5.43 g, 100%).

$\boldsymbol{R_f}$ = 0.2 (Pentane:Et_2O, 93:7).

^{1}H NMR ($CDCl_3$, 400 MHz) δ = 10.11 (1H, s, C*H*O), 7.27 (1H, dt, *J* = 8.0, 0.9 Hz, Ar*H*), 7.02 (1H, dt, *J* = 7.8, 1.0 Hz, Ar*H*), 6.93 (1H, t, *J* = 7.9 Hz, Ar*H*), 6.13 (2H, s, C*H*$_2$).

^{13}C NMR ($CDCl_3$, 101 MHz) δ = 188.1 (*C*HO), 149.3 (C_{Ar}), 149.0 (C_{Ar}), 121.8 (C_{Ar}H), 121.2 (C_{Ar}H), 119.4 (C_{Ar}), 113.5 (C_{Ar}H), 102.6 (*C*H_2).

Analytical data are in accordance with those previously reported for this compound.[14]

Benzo[*d*][1,3]dioxol-4-ol (56)[14]

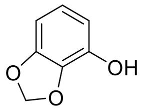

56

To a solution of aldehyde **55** (5.34 g, 5.6 mmol) in CH_2Cl_2 (120 mL) was added MCPBA (10.0 g, 40.7 mmol, 70 wt. %). The reaction mixture was heated at reflux for 16 h, filtered through Celite® and washed with CH_2Cl_2 (200 mL). The filtrate was washed with sat. aq. $NaHCO_3$ (3 × 100 mL), brine (100 mL), dried over $MgSO_4$ and concentrated *in vacuo*. The residue was dissolved in ethanolic KOH solution (5 wt.%, 100 mL) and stirred at rt for 2 h. The reaction mixture was acidified to pH 2 with HCl (1 M aq.), diluted with H_2O (100 mL) and extracted with CH_2Cl_2 (3 × 50 mL). The organic layer was washed with brine (50 mL), dried over MgSO4 and concentrated *in vacuo*. Purification by flash column chromatography (SiO_2, Pentane:Et_2O, 80:20) afforded the title compound as a white solid (3.15 g, 64%).

***R*$_f$** = 0.1 (Pentane:Et_2O, 90:10).
m.p. 61 °C.
^{1}H NMR ($CDCl_3$, 400 MHz) δ = 6.73 (1H, t, *J* = 8.1 Hz, Ar*H*), 6.49 – 6.43 (2H, m, Ar*H* × 2), 5.96 (2H, s, CH_2), 4.98 (1H, s, O*H*).
^{13}C NMR ($CDCl_3$, 101 MHz) δ = 148.8 (C_{Ar}), 139.6 (C_{Ar}), 134.1 (C_{Ar}), 122.4 (C_{Ar}H), 111.0 (C_{Ar}H), 102.1 (C_{Ar}H), 101.3 (C_{Ar}H).
Analytical data are in accordance with those previously reported for this compound.[14]

4-Hydroxybenzo[*d*][1,3]dioxole-5-carbaldehyde (57)[15]

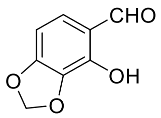

57

To a solution of phenol **56** (1.97 g, 14.3 mmol) in THF (140 mL) was added $MgCl_2$ (2.72 g, 28.5 mmol), Et_3N (3.98 mL, 28.5 mmol) and paraformaldehyde (1.28 g, 42.8 mmol). The reaction mixture was heated at 85 °C for 6 h and HCl (1 M aq., 100 mL) was added. The solution was extracted with Et_2O (3 × 100 mL) and the organic layer was washed with brine (50 mL), dried over $MgSO_4$ and concentrated *in vacuo*. Purification by recrystallisation from EtOH afforded the title compound as a white solid (2.24 g, 95%).

***R*f** = 0.1 (Pentane:Et_2O, 90:10).

m.p. 112 °C.

^{1}H NMR ($CDCl_3$, 400 MHz) δ = 10.99 (1H, s, C*H*O), 9.73 (1H, s, O*H*), 7.16 (1H, d, *J* = 8.2 Hz, Ar*H*), 6.58 (1H, d, *J* = 8.2 Hz, Ar*H*), 6.11 (2H, s, C*H*2).

^{13}C NMR ($CDCl_3$, 101 MHz) δ = 195.2 (*C*HO), 155.3 (*C*Ar), 145.6 (*C*Ar), 134.3 (*C*Ar), 130.6 (*C*ArH), 118.4 (*C*Ar), 103.0 (*C*H2), 102.1 (*C*ArH).

Analytical data are in accordance with those previously reported for this compound.[15]

Ethyl [1,3]dioxolo[4,5-*g*]benzofuran-6-carboxylate (58)[3]

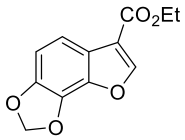

58

To a solution of aldehyde **57** (100 mg, 600 µmol) and $HBF_4{\cdot}OEt_2$ (8 µL, 60 µmol) in CH_2Cl_2 (5 mL) at 0 °C was added dropwise a solution of ethyl diazoacetate (120 µL, 960 µmol) in CH_2Cl_2 (3 mL) over a period of 5 min. The reaction mixture was stirred at room temperature for 30 min and then concentrated *in vacuo* after nitrogen evolution had ceased. THF (3 mL) and conc. H_2SO_4 (30 µL) was added and the reaction mixture was stirred at room temperature for 30 min. CH_2Cl_2 (10 mL) was added followed by solid $NaHCO_3$ until gas evolution had ceased. The solution was washed successively with water (10 mL), 1 M aq. NaOH (10 mL) and sat. aq. NaCl (10 mL), dried over $MgSO_4$ and concentrated *in vacuo*. Purification by flash column chromatography (SiO_2, Pentane:Et_2O, 90:10) afforded the title compound as a colourless oil (126 mg, 90%).

***R*f** = 0.2 (Pentane:Et_2O, 95:5).

^{1}H NMR ($CDCl_3$, 400 MHz) δ = 8.16 (1H, s, Ar*H*), 7.55 (1H, d, *J* = 8.3 Hz, Ar*H*), 6.96 (1H, d, *J* = 8.3 Hz, Ar*H*), 6.09 (2H, s, C*H*2), 4.39 (2H, q, *J* = 7.1 Hz, C*H*2CH3), 1.41 (3H, t, *J* = 7.1 Hz, CH2C*H*3).

^{13}C NMR ($CDCl_3$, 101 MHz) δ = 163.4 (*C*O2CH2CH3), 151.0 (*C*ArH), 146.9 (*C*Ar), 139.3 (*C*Ar), 131.1 (*C*Ar), 122.0 (*C*Ar), 115.4 (*C*Ar), 114.3 (*C*ArH), 106.7 (*C*ArH), 102.2 (*C*H2), 60.8 (*C*H2CH3), 14.5 (CH2*C*H3).

IR (film) ν_{max}/cm^{-1} 2926, 1717, 1487, 1271, 1086, 801.

HRMS: (EI^+) Found [M^+] = 234.0530, $C_{12}H_{10}O_5$ requires 234.0523.

Methyl 6,7-dihydro-[1,3]dioxolo[4,5-*g*]benzofuran-6-carboxylate (59)[4]

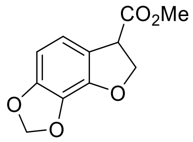

59

Mg turnings (110 mg, 4.54 mmol) were added to a solution of benzofuran **58** (200 mg, 908 μmol) in MeOH (6 mL). After 1 h the reaction mixture was evaporated to dryness and re–dissolved in CH_2Cl_2 (20 mL). The solution was washed successively with sat. aq. NH_4Cl (20 mL), sat. aq. NaCl (20 mL), dried over $MgSO_4$ and concentrated *in vacuo*. Purification by flash column chromatography (SiO_2, Pentane:Et_2O, 85:15) afforded the title compound as a colourless oil (166 mg, 82%).

***R*f** = 0.3 (Pentane:Et_2O, 80:20).
^{1}H NMR ($CDCl_3$, 400 MHz) δ = 6.85 (1H, dd, *J* = 7.9, 1.2 Hz, Ar*H*), 6.43 (1H, d, *J* = 7.9 Hz, Ar*H*), 6.05 – 5.86 (2H, m, CH_2), 5.01 (1H, dd, *J* = 9.3, 6.3 Hz, C*H*), 4.73 (1H, t, *J* = 9.3 Hz, CH_AH_B), 4.28 (1H, ddd, *J* = 9.4, 6.2, 1.2 Hz, CH_AH_B), 3.77 (3H, s, CO_2CH_3).
^{13}C NMR ($CDCl_3$, 101 MHz) δ = 171.7 (CO_2CH_3), 150.1 (C_{Ar}), 142.6 (C_{Ar}), 130.5 (C_{Ar}), 120.7 (C_{Ar}), 117.7 (C_{Ar}H), 101.8 (CH_2), 101.6 (C_{Ar}H), 74.7 (CH_AH_B), 52.8 (CO_2CH_3), 47.0 (*C*H).
IR (film) ν_{max}/cm^{-1} 2900, 1735, 1470, 1250, 1192, 1069, 1025.
HRMS: ($APCI^+$) Found $[M + H]^+$ = 223.0600, $C_{11}H_{11}O_5$ requires 223.0601.

Ethyl 3-(3-methoxy-3-oxoprop-1-yn-1-yl)-4-(trimethylsilyl)-2,3-dihydrobenzofuran-3-carboxylate (23)

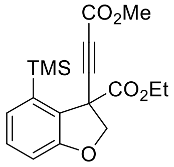

23

To a stirred solution of ester **62*** (310 mg, 1.17 mmol) in THF (10 mL) at –78 °C was added LiHMDS (1.41 mL, 1.41 mmol, 1 M in THF). After 10 min chloroacetylene **20** (208 mg, 1.76 mmol) in THF (0.5 mL) was added dropwise and the reaction mixture was stirred at –78 °C for 1 h. Sat. aq. NH_4Cl (5 mL) was added and the mixture extracted with Et_2O

(3 × 20 mL). The combined organic phase was washed with sat. aq. NaCl (20 mL), dried over $MgSO_4$ and concentrated *in vacuo*. Purification by flash column chromatography (SiO_2, Pentane:Et_2O, 85:15) afforded the title compound as a colourless oil (280 mg, 69%).

***R*f** = 0.2 (Pentane:Et_2O, 90:10).
^{1}H NMR ($CDCl_3$, 400 MHz) δ = 7.04 (1H, t, *J* = 3.8 Hz, Ar*H*), 6.93 (1H, dd, *J* = 7.5, 1.2 Hz, Ar*H*), 6.66 (1H, dd, *J* = 7.9, 1.2 Hz, Ar*H*), 4.55 (2H, s, C$H_A$$H_B$ and C$H_A$$H_B$), 4.09 – 3.98 (2H, m, $CO_2CH_2CH_3$), 3.55 (3H, s, CO_2CH_3), 1.07 (3H, t, *J* = 7.1 Hz, $CO_2CH_2CH_3$), 0.11 (9H, s, TMS-Si(CH_3)$_3$).
^{13}C NMR ($CDCl_3$, 101 MHz) δ = 169.7 ($CO_2CH_2CH_3$), 159.8 (C_{Ar}), 153.7 (CO_2CH_3), 138.4 (C_{Ar}), 129.9 (C_{Ar}H), 129.8 (C_{Ar}), 128.6 (C_{Ar}H), 111.6 (C_{Ar}H), 85.6 (CH_AH_B), 81.1 (*C*≡CCO_2CH_3), 78.2 (C≡*C*CO_2CH_3), 63.1 ($CO_2CH_2CH_3$), 53.0 (CO_2CH_3), 52.8 ($C_{Ar}C$), 14.1 ($CO_2CH_2CH_3$), 0.5 (TMS-Si(CH_3)$_3$).
IR (film) ν_{max}/cm^{-1} 2956, 2244, 1742, 1719, 1283, 1249, 839.
HRMS: (ESI$^+$) Found [M + Na]$^+$ = 369.1129, $C_{18}H_{22}O_5NaSi$ requires 369.1129.

*The starting material used to perform this alkynylation reaction was synthesised according to the procedures detailed below:

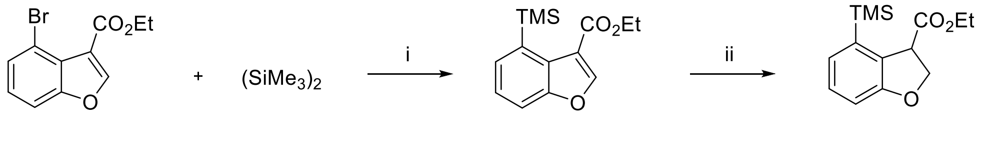

Scheme 2. Reagents and conditions: i) 1.2 eq. **60**, 1.5 mol% $Pd_2(dba)_3$, 9 mol% JohnPhos, 5 eq. KF, 2 eq. H_2O, DMPU, 100 °C, 16 h, 58%, ii) Mg, MeOH, rt, 1 h, 100%

Ethyl 4-(trimethylsilyl)benzofuran-3-carboxylate (61)[16]

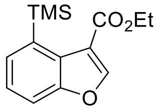

61

To a solution of bromide **37** (140 mg, 500 μmol) in degassed DMPU (1.6 mL) was added tris(dibenzylideneacetone)dipalladium(0) (7.0 mg, 7.5 μmol), JohnPhos (13 mg, 45 μmol) and KF (150 mg, 2.5 mmol). The reaction mixture was stirred for 5 min at rt and water (18 μL, 1.0 mmol) and hexamethyldisilane **60** (120 μL, 600 μmol) were added successively.

The reaction mixture was heated at 100 °C for 16 h and hexane (40 mL) was added. The organic layer was washed with sat. aq. LiCl (3 × 20 mL), dried over $MgSO_4$ and concentrated *in vacuo*. Purification by flash column chromatography (SiO_2, Pentane:Et_2O, 99:1) afforded the title compound as a pale yellow oil (76 mg, 58%).

***R*f** = 0.8 (Pentane: Et_2O, 90:10).
^{1}H NMR ($CDCl_3$, 400 MHz) δ = 8.26 (1H, s, Ar*H*), 7.59 (1H, dd, *J* = 7.2, 1.1 Hz, Ar*H*), 7.54 (1H, dd, *J* = 8.3, 1.1 Hz, Ar*H*), 7.34 (1H, dd, *J* = 8.3, 7.3 Hz, Ar*H*), 4.38 (2H, q, *J* = 7.1 Hz, $CO_2CH_2CH_3$), 1.39 (3H, t, *J* = 7.1 Hz, $CO_2CH_2CH_3$), 0.40 (9H, s, TMS-Si(CH_3)$_3$).
^{13}C NMR ($CDCl_3$, 126 MHz) δ = 163.5 ($CO_2CH_2CH_3$), 155.6 (C_{Ar}), 151.4 (C_{Ar}H), 135.1 (C_{Ar}), 131.7 (C_{Ar}H), 128.0 (C_{Ar}), 124.6 (C_{Ar}H), 116.6 (C_{Ar}), 112.8 (C_{Ar}H), 60.7 ($CO_2CH_2CH_3$), 14.6 ($CO_2CH_2CH_3$), 1.3 (TMS-Si(CH_3)$_3$).
IR (film) ν_{max}/cm^{-1} 2981, 2958, 1732, 1556, 1401, 1144, 842, 764.
HRMS: (APCI$^+$) Found [M + H]$^+$ = 263.1090, $C_{14}H_{19}O_3Si$ requires 263.1098.

Ethyl 4-(trimethylsilyl)-2,3-dihydrobenzofuran-3-carboxylate (62)[4]

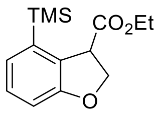

62

To a solution of benzofuran **61** (70 mg, 270 μmol) in MeOH (2.0 mL) was added freshly ground Mg turnings (32 mg, 1.30 mmol). The reaction was stirred at rt for 1 h before sat. aq. NH_4Cl (5 mL) was added followed by water (20 mL) and Et_2O (30 mL). The layers were separated and the organic layer was washed with brine (20 mL), dried over $MgSO_4$ and concentrated *in vacuo*. Purification by flash column chromatography (SiO_2, Pentane:Et_2O, 95:5) afforded the title compound as a white solid (70 mg, 100%).

***R*f** = 0.4 (Pentane:Et_2O, 90:10).
^{1}H NMR ($CDCl_3$, 400 MHz) δ = 7.20 (1H, t, *J* = 7.8 Hz, Ar*H*), 7.05 (1H, d, *J* = 8.5 Hz, Ar*H*), 6.86 (1H, d, *J* = 8.0 Hz, Ar*H*), 4.74 (1H, dt, *J* = 9.3, 2.5 Hz, CH_AH_B), 4.60 (1H, t, *J* = 9.2 Hz, CH_AH_B), 4.25 (1H, dt, *J* = 9.4, 2.5 Hz, C*H*), 4.17 (2H, q, *J* = 7.2 Hz, $CO_2CH_2CH_3$), 1.25 (3H, t, *J* = 7.1 Hz, $CO_2CH_2CH_3$), 0.29 (9H, s, TMS-Si(CH_3)$_3$).

^{13}C NMR ($CDCl_3$, 126 MHz) δ = 172.8 ($CO_2CH_2CH_3$), 159.8 (C_{Ar}), 137.9 (C_{Ar}), 129.4 (C_{Ar}), 129.0 (C_{Ar}H), 127.0 (C_{Ar}H), 110.9 (C_{Ar}H), 74.0 (CH_AH_B), 61.5 ($CO_2CH_2CH_3$), 48.5 (*C*H), 14.3 ($CO_2CH_2CH_3$), –0.3 (TMS-Si(CH_3)$_3$).
IR (film) ν_{max}/cm^{-1} 2956, 2899, 1732, 1571, 1422, 1249,
HRMS: (APCI$^+$) Found [M + H]$^+$ = 265.1258, $C_{14}H_{21}O_3Si$ requires 265.1255.

2-(Trimethylsilyl)ethyl 3-(3-methoxy-3-oxoprop-1-yn-1-yl)-2,3-dihydrobenzofuran-3-carboxylate (24)

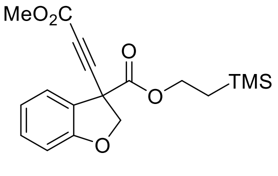

24

To a stirred solution of ester **63*** (529 mg, 2.00 mmol) in THF (15 mL) at –78 °C was added LiHMDS (2.40 mL, 2.40 mmol, 1 M in THF). After 10 min chloroacetylene **20** (356 mg, 3.00 mmol) in THF (0.5 mL) was added dropwise and the reaction mixture was stirred at –78 °C for 1 h. Sat. aq. NH_4Cl (5 mL) was added and the mixture extracted with Et_2O (3 × 50 mL). The combined organic phase was washed with sat. aq. NaCl (50 mL), dried over $MgSO_4$ and concentrated *in vacuo*. Purification by flash column chromatography (SiO_2, Pentane:Et_2O, 95:5) afforded the title compound as a colourless oil (667 mg, 96%).

***R*f** = 0.6 (Pentane:Et_2O, 75:25).
^{1}H NMR ($CDCl_3$, 400 MHz) δ = 7.47 (1H, dd, *J* = 7.6, 1.4 Hz, Ar*H*), 7.28 – 7.23 (1H, m, Ar*H*), 6.96 (1H, td, *J* = 7.6, 1.0 Hz, Ar*H*), 6.85 (1H, d, *J* = 8.1 Hz, Ar*H*), 5.21 (1H, d, *J* = 9.2 Hz, CH_AH_B), 4.70 (1H, d, *J* = 9.1 Hz, CH_AH_B), 4.33 – 4.26 (2H, m, CO_2CH_2), 3.77 (3H, s, CO_2CH_3), 1.12 – 0.97 (2H, m, $CO_2CH_2CH_2$), 0.04 (9H, s, TMS–(CH_3)$_3$).
^{13}C NMR ($CDCl_3$, 110 MHz) δ = 167.9 (CO_2CH_3), 159.5 (CO_2CH_2), 153.7 (C_{Ar}), 130.9 (C_{Ar}H), 125.4 (C_{Ar}), 125.2 (C_{Ar}H), 121.7 (C_{Ar}H), 110.8 (C_{Ar}H), 84.8 ($C{\equiv}CCO_2CH_3$), 79.1 (CH_AH_B), 75.8 ($C{\equiv}CCO_2CH_3$), 65.9 (CO_2CH_2), 53.0 (CO_2CH_3), 51.2 ($C_{Ar}C$), 17.4 ($CO_2CH_2CH_2$), –1.4 (TMS–(CH_3)$_3$).
IR (film) ν_{max}/cm^{-1} 2954, 2243, 1739, 1718, 1480, 1278, 1235, 1174, 858, 836, 750.
HRMS: (ESI$^+$) Found [M + Na]$^+$ = 369.1130, $C_{18}H_{22}O_5NaSi$ requires 369.1129.

*The starting material used to perform this alkynylation reaction was synthesised according to the procedure detailed below:

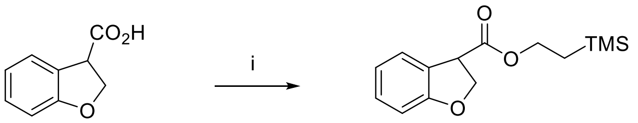

48 63

Scheme 3. Reagents and conditions: i) EDC·HCl, DMAP, 2-(trimethylsilyl)ethanol, Et_3N, CH_2Cl_2, rt, 16 h, 88%

2-(Trimethylsilyl)ethyl 2,3-dihydrobenzofuran-3-carboxylate (63)[17]

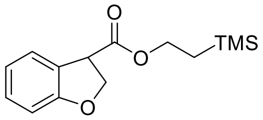

63

To a solution of acid **48** (1.0 g, 6.1 mmol) in CH_2Cl_2 (30 mL) was added *N*-(3-dimethylaminopropyl)-*N*'-ethylcarbodiimide hydrochloride (1.8 g, 9.1 mmol), 4-(dimethylamino)pyridine (74 mg, 610 μmol) and 2-(trimethylsilyl)ethanol (1.8 mL, 12 mmol). The reaction mixture was stirred at rt for 16 h, diluted with CH_2Cl_2 (30 mL) and water (50 mL) and the layers separated. The aqueous layer was extracted with CH_2Cl_2 (3 × 30 mL) and the combined organic phase was washed with sat. aq. NaCl (30 mL), dried over $MgSO_4$ and concentrated *in vacuo*. Purification by flash column chromatography (SiO_2, Pentane:Et_2O, 97:3) afforded the title compound as a colourless oil (1.4 g, 88%).

***R*f** = 0.4 (Pentane:Et_2O, 90:10).

^{1}H NMR ($CDCl_3$, 400 MHz) δ = 7.39 (1H, d, *J* = 7.5 Hz, Ar*H*), 7.21 – 7.15 (1H, m, Ar*H*), 6.89 (1H, td, *J* = 7.4, 1.0 Hz, Ar*H*), 6.82 (1H, d, *J* = 7.8 Hz, Ar*H*), 4.93 (1H, dd, *J* = 9.2, 6.7 Hz, C$H_A$$H_B$), 4.66 (1H, t, *J* = 9.8 Hz, C$H_A$$H_B$), 4.31 (1H, dd, *J* = 9.5, 6.2 Hz, CO_2CH_2), 4.25 (2H, td, *J* = 8.8, 0.9 Hz, C*H*), 1.08 – 0.99 (2H, m, $CO_2CH_2CH_3$), 0.05 (9H, s, TMS–$(CH_3)_3$).

^{13}C NMR ($CDCl_3$, 110 MHz) δ = 171.4 (CO_2CH_2), 159.9 (C_{Ar}), 129.5 (C_{Ar}H), 125.4 (C_{Ar}H), 124.5 (C_{Ar}), 120.7 (C_{Ar}H), 110.0 (C_{Ar}H), 72.6 (CH_AH_B), 64.0 (C*H*), 47.4 (CO_2CH_2), 17.6 ($CO_2CH_2CH_3$), –1.4 (TMS–$(CH_3)_3$).

IR (film) ν_{max}/cm^{-1} 2954, 1732, 1597, 1482, 1235, 1172, 857, 749.

HRMS: (ESI$^+$) Found $[M + Na]^+$ = 207.1074, $C_{14}H_{20}O_3SiNa$ requires 207.1074.

Methyl 3-(3-(tert-butoxy)-3-oxoprop-1-yn-1-yl)-2,3-dihydrobenzofuran-3-carboxylate (25)

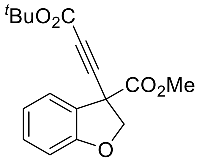

25

To a stirred solution of ester **17** (248 mg, 1.39 mmol) in THF (11 mL) at –78 °C was added LiHMDS (1.67 mL, 1.67 mmol, 1 M in THF). After 10 min chloroacetylene **30** (335 mg, 2.09 mmol) in THF (1 mL) was added dropwise and the reaction mixture was stirred at –78 °C for 1 h. Sat. aq. $NaHCO_3$ (2 mL) was added and the mixture extracted with Et_2O (3 × 30 mL). The combined organic phase was washed with sat. aq. NaCl (30 mL), dried over $MgSO_4$ and concentrated *in vacuo*. Purification by flash column chromatography (SiO_2, Pentane:Et_2O, 90:10) afforded the title compound as a colourless oil (420 mg, 100%).

***R*f** = 0.3 (Pentane:EtOAc, 90:10).
^{1}H NMR ($CDCl_3$, 500 MHz) δ = 7.48 (1H, dd, *J* = 7.6, 1.2 Hz, Ar*H*), 7.27 – 7.23 (1H, m, Ar*H*), 6.96 (1H, td, *J* = 7.6, 1.0 Hz, Ar*H*), 6.85 (1H, d, *J* = 8.1 Hz, Ar*H*), 5.21 (1H, d, *J* = 9.2 Hz, C$H_A$$H_B$), 4.70 (1H, d, *J* = 9.2 Hz, C$H_A$$H_B$), 3.82 (3H, s, CO_2CH_3), 1.48 (9H, s, $CO_2C(CH_3)_3$).
^{13}C NMR ($CDCl_3$, 126 MHz) δ = 168.7 (CO_2CH_3), 159.4 ($CO_2C(CH_3)_3$), 152.3 (C_{Ar}), 130.9 (C_{Ar}H), 125.5 (C_{Ar}), 125.4 (C_{Ar}H), 121.7 (C_{Ar}H), 110.8 (C_{Ar}H), 84.1 (C≡$C$$CO_2C(CH_3)_3$), 81.5 ($CO_2C(CH_3)_3$), 79.2 ($CH_AH_B$), 77.4 ($C$≡$CCO_2C(CH_3)_3$), 54.1 ($CO_2CH_3$), 51.0 ($C_{Ar}C$), 28.1 ($CO_2C(CH_3)_3$).
IR (film) ν_{max}/cm^{-1} 2981, 2240, 1747, 1707, 1480, 1291, 1251, 1153, 837, 752.
HRMS: (ESI$^+$) Found [M + Na]$^+$ = 325.1047, $C_{17}H_{18}O_5Na$ requires 325.1057.

***tert*-Butyl 3-(3-(methoxy(methyl)carbamoyl)-2,3-dihydrobenzofuran-3-yl)propiolate (26)**

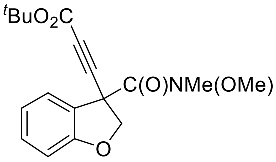

26

To a stirred solution of amide **64*** (207 mg, 1.00 mmol) in THF (8 mL) at –78 °C was added LiHMDS (1.20 mL, 1.20 mmol, 1 M in THF). After 10 min chloroacetylene **30** (241 mg, 1.50 mmol) in THF (0.6 mL) was added dropwise and the reaction mixture was stirred at

–78 °C for 1 h. Sat. aq. $NaHCO_3$ (2 mL) was added and the mixture extracted with Et_2O (3 × 30 mL). The combined organic phase was washed with sat. aq. NaCl (30 mL), dried over $MgSO_4$ and concentrated *in vacuo*. Purification by flash column chromatography (SiO_2, Pentane:Et_2O, 75:25) afforded the title compound as a colourless oil (282 mg, 85%).

***R*$_f$** = 0.7 (Pentane:EtOAc, 50:50).
^{1}H NMR ($CDCl_3$, 400 MHz) δ = 7.54 (1H, dd, *J* = 7.5, 1.1 Hz, Ar*H*), 7.25 – 7.19 (1H, m, Ar*H*), 6.95 (1H, td, *J* = 7.5, 1.0 Hz, Ar*H*), 6.83 (1H, d, *J* = 8.2 Hz, Ar*H*), 5.09 (1H, d, *J* = 9.3 Hz, CH_AH_B), 4.76 (1H, d, *J* = 9.2 Hz, CH_AH_B), 3.85 (3H, s, $C(O)N(CH_3)OCH_3$), 3.24 (3H, s, $C(O)N(CH_3)OCH_3$), 1.47 (9H, s, $CO_2C(CH_3)_3$).
^{13}C NMR ($CDCl_3$, 101 MHz) δ = 167.8 ($C(O)N(CH_3)OCH_3$), 159.8 ($CO_2C(CH_3)_3$), 152.6 (C_{Ar}), 130.4 ($C_{Ar}H$), 127.1 ($C_{Ar}H$), 125.8 (C_{Ar}), 121.4 ($C_{Ar}H$), 110.5 ($C_{Ar}H$), 83.9 ($C{\equiv}CCO_2C(CH_3)_3$), 83.5 ($C{\equiv}CCO_2C(CH_3)_3$), 79.7 (CH_AH_B), 77.4 ($C_{Ar}C$), 61.1 ($C(O)N(CH_3)OCH_3$), 50.6 ($CO_2C(CH_3)_3$), 33.6 ($C(O)N(CH_3)OCH_3$), 28.1 ($CO_2C(CH_3)_3$).
IR (film) ν_{max}/cm^{-1} 2980, 2940, 2234, 1704, 1671, 1479, 1461, 1395, 1287, 1240, 1152, 752.
HRMS: (ESI^+) Found $[M + H]^+$ = 332.1493, $C_{18}H_{25}O_5N$ requires 332.1493.

*The starting material used to perform this alkynylation reaction was synthesised according to the procedure detailed below:

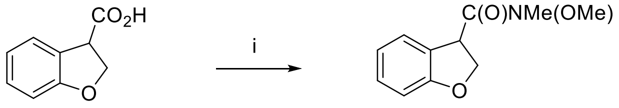

48 **64**

Scheme 4. Reagents and conditions: i) EDC·HCl, DMAP, HNMe(OMe)·HCl, Et_3N, CH_2Cl_2, rt, 16 h, 100%

N-Methoxy-*N*-methyl-2,3-dihydrobenzofuran-3-carboxamide (64)[17]

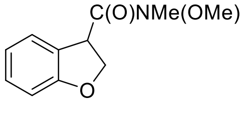

64

To a solution of acid **48** (240 mg, 1.46 mmol) in CH_2Cl_2 (12 mL) at rt was added *N*-*O*-dimethylhydroxylamine hydrochloride (178 mg, 1.82 mmol), *N*-(3-dimethylaminopropyl)-*N*'-ethylcarbodiimide hydrochloride (450 mg, 2.35 mmol), 4-(dimethylamino)pyridine (25 mg,

200 μmol) and Et_3N (254 μL, 1.82 mmol). The reaction mixture was stirred at rt for 16 h, diluted with CH_2Cl_2 (50 mL) and water (50 mL) and the layers separated. The aqueous layer was extracted with CH_2Cl_2 (3 × 30 mL) and the combined organic phase was washed with sat. aq. NaCl (30 mL), dried over $MgSO_4$ and concentrated *in vacuo*. Purification by flash column chromatography (SiO_2, Pentane:EtOAc, 65:35) afforded the title compound as a pale yellow oil (303 mg, 100%).

***R*f** = 0.15 (Pentane:EtOAc, 80:20).
^{1}H NMR ($CDCl_3$, 400 MHz) δ = 7.30 (1H, d, *J* = 7.1 Hz, Ar*H*), 7.15 (1H, td, *J* = 7.8, 1.4 Hz, Ar*H*), 6.85 (1H, td, *J* = 7.5, 1.1 Hz, Ar*H*), 6.82 (1H, d, *J* = 8.8 Hz, Ar*H*), 4.93 – 4.84 (1H, m, CH_AH_B), 4.74 – 4.65 (2H, m, CH_AH_B and C*H*), 3.75 (3H, s, $N(CH_3)OCH_3$), 3.27 (3H, s, $N(CH_3)OCH_3$).
^{13}C NMR ($CDCl_3$, 101 MHz) δ = 172.3 ($C(O)N(CH_3)OCH_3$), 160.3 (C_{Ar}), 129.3 ($C_{Ar}H$), 125.7 (C_{Ar}), 125.5 ($C_{Ar}H$), 120.7 ($C_{Ar}H$), 110.0 ($C_{Ar}H$), 73.2 (CH_AH_B), 61.5 ($N(CH_3)OCH_3$), 45.1 (*C*H), 32.8 ($N(CH_3)OCH_3$).
IR (film) ν_{max}/cm^{-1} 2940, 1664, 1437, 1234, 753.
HRMS: (ESI^+) Found $[M + Na]^+$ = 230.0788, $C_{11}H_{13}O_3NNa$ requires 230.0788.

Methyl 3-(3-((ethylthio)carbonyl)-2,3-dihydrobenzofuran-3-yl)propiolate (27)

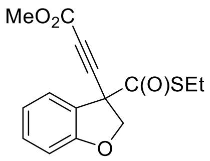

27

To a stirred solution of thioester **65*** (208 mg, 1.00 mmol) in THF (7 mL) at –78 °C was added LiHMDS (1.20 mL, 1.20 mmol, 1 M in THF). After 10 min chloroacetylene **20** (178 mg, 1.50 mmol) in THF (0.5 mL) was added dropwise and the reaction mixture was stirred at –78 °C for 1 h. Sat. aq. NH_4Cl (5 mL) was added and the mixture extracted with Et_2O (3 × 50 mL). The combined organic phase was washed with sat. aq. NaCl (50 mL), dried over $MgSO_4$ and concentrated *in vacuo*. Purification by flash column chromatography (SiO_2, Pentane:Et_2O, 93:7) afforded the title compound as a colourless oil (288 mg, 99%).

***R*f** = 0.25 (Pentane:Et_2O, 90:10).

^{1}H NMR ($CDCl_3$, 400 MHz) δ = 7.47 (1H, dd, *J* = 7.5, 1.4 Hz, Ar*H*), 7.30 – 7.26 (1H, m, Ar*H*), 6.97 (1H, td, *J* = 7.6, 1.0 Hz, Ar*H*), 6.87 (1H, d, *J* = 8.1 Hz, Ar*H*), 5.13 (1H, d, *J* = 9.2 Hz, CH_AH_B), 4.68 (1H, d, *J* = 9.2 Hz, CH_AH_B), 3.79 (3H, s, CO_2CH_3), 2.92 (2H, q, *J* = 7.4 Hz, CH_2CH_3), 1.26 (3H, t, *J* = 7.4 Hz, CH_2CH_3).

^{13}C NMR ($CDCl_3$, 110 MHz) δ = 195.5 (*C*(O)SCH_2CH_3), 160.0 (C_{Ar}), 153.5 (CO_2CH_3), 131.2 (C_{Ar}H), 125.7 (C_{Ar}), 125.2 (C_{Ar}H), 121.7 (C_{Ar}H), 111.0 (C_{Ar}H), 84.2 (*C*≡CCO_2CH_3), 79.5 (CH_AH_B), 78.0 (C≡*C*CO_2CH_3), 58.2 (C_{Ar}C), 53.1 (CO_2CH_3), 24.8 (CH_2CH_3), 14.3 (CH_2CH_3).

IR (film) ν_{max}/cm^{-1} 2954, 2239, 1716, 1681, 1478, 1273, 1235, 942, 749.

HRMS: (ESI^+) Found $[M + H]^+$ = 291.0687, $C_{15}H_{15}O_4S$ requires 291.0686.

*The starting material used to perform this alkynylation reaction was synthesised according to the procedure detailed below:

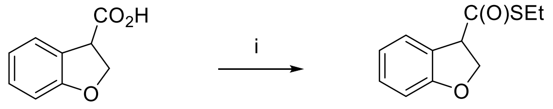

Scheme 5. Reagents and conditions: i) EDC·HCl, DMAP, thioethanol, CH_2Cl_2, 16 h, 92%

S-Ethyl 2,3-dihydrobenzofuran-3-carbothioate (65)[17]

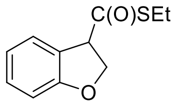

65

To a solution of acid **48** (1.0 g, 6.1 mmol) in CH_2Cl_2 (30 mL) was added *N*-(3-dimethylaminopropyl)-*N*'-ethylcarbodiimide hydrochloride (1.8 g, 9.1 mmol), 4-(dimethylamino)pyridine (74 mg, 610 µmol) and thioethanol (1.8 mL, 24 mmol). The reaction mixture was stirred at rt for 16 h, diluted with CH_2Cl_2 (30 mL) and water (50 mL) and the layers separated. The aqueous layer was extracted with CH_2Cl_2 (3 × 30 mL) and the combined organic phase was washed with sat. aq. NaCl (30 mL), dried over $MgSO_4$ and concentrated *in vacuo*. Purification by flash column chromatography (SiO_2, Pentane:Et_2O, 97:3) afforded the title compound as a colourless oil (1.2 g, 92%).

***R*f** = 0.7 (Pentane:Et_2O, 70:30).

^{1}H NMR ($CDCl_3$, 400 MHz) δ = 7.40 (1H, d, *J* = 7.6 Hz, Ar*H*), 7.26 – 7.20 (1H, m, Ar*H*), 6.93 (1H, td, *J* = 7.5, 1.0 Hz, Ar*H*), 6.87 (1H, d, *J* = 7.9 Hz, Ar*H*), 4.92 (1H, dd, *J* = 9.4, 5.6 Hz, $C\mathit{H}_AH_B$), 4.68 (1H, t, *J* = 9.4 Hz, $CH_A\mathit{H}_B$), 4.47 (1H, dd, *J* = 9.2, 5.7 Hz, C*H*), 2.95 (2H, q, *J* = 7.4 Hz, $C\mathit{H}_2CH_3$), 1.29 (3H, t, *J* = 7.4 Hz, $CH_2C\mathit{H}_3$).

^{13}C NMR ($CDCl_3$, 110 MHz) δ = 198.5 (*C*(O)), 160.4 (C_{Ar}), 129.9 ($\mathit{C}_{Ar}H$), 125.7 ($\mathit{C}_{Ar}H$), 124.4 (C_{Ar}), 120.8 ($\mathit{C}_{Ar}H$), 110.3 ($\mathit{C}_{Ar}H$), 73.2 ($\mathit{C}H_AH_B$), 55.9 (*C*H), 23.9 ($\mathit{C}H_2CH_3$), 14.7 ($CH_2\mathit{C}H_3$).

IR (film) ν_{max}/cm^{-1} 2981, 1678, 1480, 1460, 1232, 971, 748.

HRMS: (ESI$^+$) Found $[M + Na]^+$ = 209.0633, $C_{11}H_{13}O_2SNa$ requires 209.0631.

Methyl 3-(3-(4-methyl-2,6,7-trithiabicyclo[2.2.2]octane-1-carbonyl)-2,3-dihydrobenzofuran-3-yl)propiolate (28)

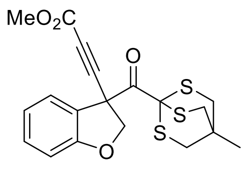

28

To a stirred solution of ketone **70*** (200 mg, 618 μmol) in THF (5 mL) at –78 °C was added LiHMDS (928 μL, 928 μmol, 1 M in THF). After 10 min chloroacetylene **20** (88 mg, 743 μmol) in THF (0.5 mL) was added dropwise and the reaction mixture was stirred at –78 °C for 30 min. Sat. aq. NH_4Cl (5 mL) and water (20 mL) were added and the mixture extracted with Et_2O (3 × 30 mL). The combined organic phase was washed with sat. aq. NaCl (20 mL), dried over $MgSO_4$ and concentrated *in vacuo*. Purification by flash column chromatography (SiO_2, Pentane:Et_2O, 65:35) afforded the title compound as a colourless oil (136 mg, 54%).

***R*f** = 0.2 (Pentane:Et_2O, 60:40).

^{1}H NMR ($CDCl_3$, 400 MHz) δ = 7.51 (1H, dd, *J* = 7.8, 1.4 Hz, Ar*H*), 7.28 – 7.24 (2H, m, Ar*H* × 2), 6.96 (1H, td, *J* = 7.6, 1.0 Hz, Ar*H*), 6.85 (1H, d, *J* = 8.0 Hz, Ar*H*), 5.52 (1H, d, *J* = 9.7 Hz, $C\mathit{H}_AH_B$), 4.80 (1H, d, *J* = 9.6 Hz, $CH_A\mathit{H}_B$), 3.77 (3H, s, $CO_2C\mathit{H}_3$), 3.06 (6H, s, $C\mathit{H}_2$ × 3), 1.26 (3H, s, $C\mathit{H}_3$).

^{13}C NMR ($CDCl_3$, 110 MHz) δ = 194.5 (*C*(O)), 160.0 ($\mathit{C}O_2CH_3$), 153.6 (C_{Ar}), 131.0 ($\mathit{C}_{Ar}H$), 126.8 ($\mathit{C}_{Ar}H$), 125.4 (C_{Ar}), 121.7 ($\mathit{C}_{Ar}H$), 110.7 ($\mathit{C}_{Ar}H$), 83.9 ($\mathit{C}{\equiv}CCO_2CH_3$), 79.8 ($C{\equiv}\mathit{C}CO_2CH_3$), 79.6 ($\mathit{C}H_AH_B$), 64.4 ($C_{Ar}\mathit{C}$), 56.5 (C(O)*C*), 53.0 ($CO_2\mathit{C}H_3$), 36.5 ($\mathit{C}H_2$ × 3), 29.7 ($\mathit{C}H_3$), 29.5 ($CH_3\mathit{C}$).

IR (film) ν_{max}/cm^{-1} 2955, 1722, 1596, 1479, 1463, 1174, 1020, 749.

HRMS: (ESI$^+$) Found $[M + H]^+$ = 407.0440, $C_{19}H_{19}O_4S_3$ requires 407.0439.

*The starting material used to perform this alkynylation reaction was synthesised according to the procedure detailed below:

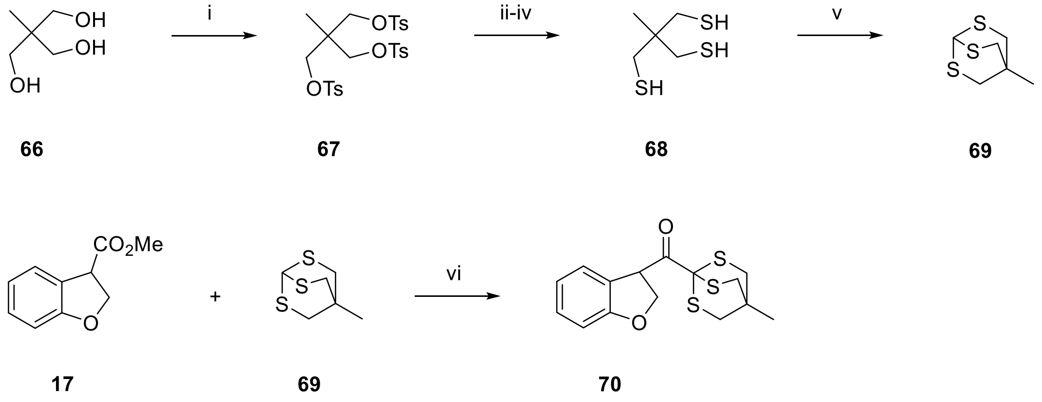

Scheme 6. Reagents and conditions: i) TsCl, py, 0 °C – rt, 16 h, 100%, ii) NaSH·9H_2O, S, DMF, 80 °C, 1 h, then **71**, 100 °C, 4 h, iii) Cu, toluene, 115 °C, 16 h, iv) Zn, HCl, EtOH, rt, 24 h, 94% over 3 steps, v) trimethylorthoformate, PTSA, toluene, 115 °C, 24 h, 54%, vi) **69**, ^{n}BuLi, THF, –78 °C, 10 min, then **17**, 30 min, 76%,

2-Methyl-2-((tosyloxy)methyl)propane-1,3-diyl bis(4-methylbenzenesulfonate) (67)[18]

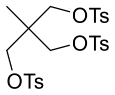

67

To a solution of 1,1,1-tris(hydroxymethyl)ethanol **66** (4.00 g, 3.3 mmol) in pyridine (40 mL) at 0 °C was added *p*-toluenesulfonyl chloride (25.4 g, 133 mmol). The reaction mixture was stirred for 16 h then Et_2O (200 mL) was added followed by HCl (6 M aq., 400 mL). The two layers were separated and the organic layer was washed with sat. aq. NaCl (100 mL), dried over $MgSO_4$ and concentrated *in vacuo* to afford the title compound (19.4 g, 100 %) as a white solid with no further purification necessary.

***R*f** = 0.5 (Pentane:Et_2O, 70:30).

m.p. 107 °C.

^{1}H NMR ($CDCl_3$, 400 MHz) δ = 7.70 (6H, d, J = 8.4 Hz, ArH × 6), 7.35 (6H, d, J = 8.1 Hz, ArH × 6), 3.76 (6H, s, CH_2 × 3), 2.46 (9H, s, CH_3 × 3), 0.88 (3H, s, CH_3).

^{13}C NMR ($CDCl_3$, 110 MHz) δ = 145.5 (C_{Ar} × 3), 132.0 (C_{Ar} × 3), 130.2 ($C_{Ar}H$ × 6), 128.0 ($C_{Ar}H$ × 6), 69.9 (CH_2 × 3), 39.5 (CH_3C), 21.8 (CH_3 × 3), 16.2 (CH_3).
Analytical data are in accordance with those previously reported for this compound.[18]

2-(Mercaptomethyl)-2-methylpropane-1,3-dithiol (68)[18]

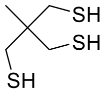

68

A solution of $NaSH\cdot 9H_2O$ (3.88 g, 47.1 mmol) and S (2.63 g, 82.2 mmol) in DMF (50 mL) was heated at 80 °C for 1 h. Tosylate **67** (6.40 g, 11.0 mmol) was added and the reaction mixture was heated at 100 °C for 4 h. The reaction was cooled and the solvent removed by distillation under reduced pressure. Toluene (50 mL) and Cu (4.73 g, 74.4 mmol) were added and the mixture was heated at 115 °C for 16 h. The reaction mixture was filtered through Celite® and washed with toluene (70 mL). The solvent was reduced *in vacuo* to approximately 50 mL then EtOH (50 mL) and Zn (10.6 g, 162 mmol) were added followed by conc. HCl (40 mL) dropwise. The reaction mixture was stirred at rt for 24 h. Water (100 mL) was added and the layers separated. The aqueous layer was washed with EtOAc (3 × 50 mL) and the combined organic phase was washed with sat. aq. NaCl (50 mL), dried over $MgSO_4$ and concentrated *in vacuo* to afford the title compound (1.74 g, 94 %) as a pale yellow oil with no further purification necessary.

R_f = 0.4 (Pentane:EtOAc, 50:50).
^{1}H NMR ($CDCl_3$, 400 MHz) δ = 2.60 (6H, d, J = 8.7 Hz, CH_2 × 3), 1.22 (3H, t, J = 8.8 Hz, SH × 3), 1.00 (3H, s, CH_3).
^{13}C NMR ($CDCl_3$, 110 MHz) δ = 39.2 (CH_3C), 31.8 (CH_2 × 3), 21.5 (CH_3).
Analytical data are in accordance with those previously reported for this compound.[18]

4-Methyl-2,6,7-trithiabicyclo[2.2.2]octane (69)[18]

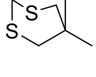

69

A solution of thiol **68** (1.70 g, 10.1 mmol), trimethylorthoformate (1.87 mL, 17.1 mmol) and PTSA (85 mg, 500 μmol) in toluene (50 mL) was heated at 115 °C for 24 h. Sat. aq. $NaHCO_3$

(50 mL) was added and the two layers separated. The aqueous layer was washed with Et_2O (3 × 50 mL) and the combined organic phase was washed with sat. aq. NaCl (50 mL), dried over $MgSO_4$ and concentrated *in vacuo*. Purification by flash column chromatography (SiO_2, Pentane:Et_2O, 95:5) afforded the title compound (1.04 g, 54 %) as a white solid.

***R*f** = 0.3 (Pentane:Et_2O, 90:10).
m.p. 130 °C.
^{1}H NMR ($CDCl_3$, 400 MHz) δ = 4.60 (1H, s, C*H*), 3.03 (6H, s, CH_2 × 3), 1.22 (3H, s, CH_3).
^{13}C NMR ($CDCl_3$, 110 MHz) δ = 38.2 (*C*H), 34.3 (*C*H_2 × 3), 30.9 (*C*H_3), 28.4 (CH_3*C*).
Analytical data are in accordance with those previously reported for this compound.[18]

(2,3-Dihydrobenzofuran-3-yl)(4-methyl-2,6,7-trithiabicyclo[2.2.2]octan-1-yl)methanone (70)

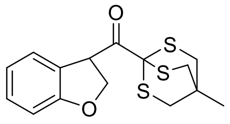

70

To a solution of thioether **69** (750 mg, 4.21 mmol) in THF (15 mL) at –78 °C was added dropwise ^{n}BuLi (1.85 mL, 740 µmol, 2.5 M hexanes) and the reaction mixture stirred for 10 min. A solution of ester **17** (600 mg, 3.36 mmol) in THF (2 mL) was added and the reaction mixture stirred for a further 20 min at –78 °C. Sat. aq. NH_4Cl (10 mL) was added followed by water (50 mL) and the mixture extracted with Et_2O (3 × 50 mL). The combined organic phase was washed with sat. aq. NaCl (20 mL), dried over $MgSO_4$ and concentrated *in vacuo*. Purification by flash column chromatography (SiO_2, Pentane:Et_2O, 80:20) afforded the title compound as a pale yellow oil (824 mg, 76%).

m.p. 130 °C.
***R*f** = 0.2 (Pentane:Et_2O, 80:20).
^{1}H NMR ($CDCl_3$, 400 MHz) δ = 7.29 (1H, dt, *J* = 7.8, 1.3 Hz, Ar*H*), 7.21 – 7.14 (1H, m, Ar*H*), 6.91 – 6.80 (2H, m, Ar*H* × 2), 5.41 – 5.30 (1H, m, C*H*), 4.79 (1H, dd, *J* = 9.7, 8.9 Hz, CH_AH_B), 4.58 (1H, dd, *J* = 8.9, 7.2 Hz, CH_AH_B), 3.17 – 3.06 (6H, m, CH_2 × 3), 1.31 (3H, s, CH_3).
^{13}C NMR ($CDCl_3$, 110 MHz) δ = 200.7 (*C*(O)), 160.8 (C_{Ar}), 129.6 (C_{Ar}H), 126.3 (C_{Ar}), 125.7 (C_{Ar}H), 121.0 (C_{Ar}H), 110.1 (C_{Ar}H), 74.9 (*C*H_AH_B), 65.7 (C(O)*C*), 50.5 (*C*H), 35.9 (*C*H_2 × 3), 30.2 (*C*H_3), 29.7 (CH_3*C*).

IR (film) ν_{max}/cm^{-1} 2957, 2914, 1715, 1481, 1459, 1233, 1119, 983, 752.

HRMS: (ESI$^+$) Found [M + H]$^+$ = 325.0386, $C_{15}H_{17}O_2S_3$ requires 325.0385.

Methyl 3-(3-(2-cyano-2-(triphenyl-λ^5-phosphaneylidene)acetyl)-2,3-dihydrobenzofuran-3-yl)propiolate (29)

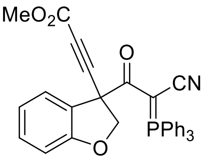

29

To a stirred solution of cyanoketophosphorane **73*** (180 mg, 400 µmol) in DMF (8 mL) at –40 °C was added LiHMDS (480 µL, 480 µmol, 1 M in THF). After 10 min chloroacetylene **20** (72 mg, 600 µmol) in DMF (0.5 mL) was added dropwise and the reaction mixture was stirred at –40 °C for 2 h. Sat. aq. NH_4Cl (5 mL) was added and the mixture extracted with EtOAc (3 × 30 mL). The combined organic phase was washed with sat. aq. LiCl (20 mL), dried over $MgSO_4$ and concentrated *in vacuo*. Purification by flash column chromatography (SiO_2, Pentane:EtOAc, 70:30) afforded the title compound as a pale yellow solid (190 mg, 90%).

$\boldsymbol{R_f}$ = 0.65 (Pentane:EtOAc, 40:60).

m.p. 91 °C

^{1}H NMR ($CDCl_3$, 400 MHz) δ = 7.79 (1H, dd, *J* = 7.6, 1.4 Hz, Ar*H*), 7.65 – 7.44 (15H, m, Ar*H* × 15), 7.25 – 7.18 (1H, m, Ar*H*), 7.01 – 6.92 (1H, m, Ar*H*), 6.81 (1H, d, *J* = 8.4 Hz, Ar*H*), 5.27 (1H, d, *J* = 9.0 Hz, CH_AH_B), 4.82 (1H, d, *J* = 8.9 Hz, CH_AH_B), 3.76 (3H, s, CO_2CH_3).

^{13}C NMR ($CDCl_3$, 126 MHz) δ = 187.3 (d, *J* = 3.9 Hz, *C*(O)C=P), 159.6 (C_{Ar}), 154.1 (CO_2CH_3), 133.6 (d, *J* = 9.8 Hz, C_{Ar}H × 6), 133.4 (d, *J* = 2.9 Hz, C_{Ar}H × 3), 130.0 (C_{Ar}H), 129.3 (d, *J* = 13.1 Hz, C_{Ar}H × 6), 126.9 (*C*≡N), 126.6 (C_{Ar}H), 122.6 (d, *J* = 93.5 Hz, C_{Ar} × 3), 121.3 (C_{Ar}H), 120.5 (d, *J* = 14.3 Hz, C_{Ar}), 110.3 (C_{Ar}H), 86.2 (*C*≡CCO_2CH_3), 79.2 (CH_AH_B), 77.3 (C≡CCO_2CH_3), 54.8 (d, *J* = 6.9 Hz, $C_{Ar}C$), 52.9 (CO_2CH_3), 47.7 (d, *J* = 125.0 Hz, C(O)*C*=P).

^{31}P NMR (162 MHz, $CDCl_3$) δ = 22.8.

IR (film) ν_{max}/cm^{-1} 3010, 2235, 2179, 1714, 1653, 1460, 1276, 1236, 750.

HRMS: (ESI$^+$) Found [M + H]$^+$ = 530.1516, $C_{33}H_{25}O_4NP$ requires 530.1516.

*The starting material used to perform this alkynylation reaction was synthesised according to the procedure detailed below:

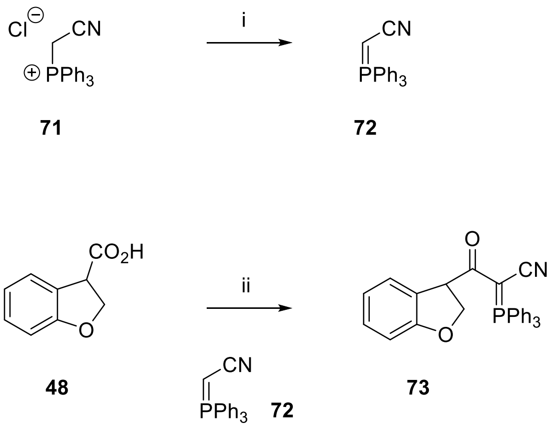

Scheme 7. Reagents and conditions: i) Et_3N, CH_2Cl_2, rt, 30 min, 84%, ii) EDC·HCl, DMAP, CH_2Cl_2, 16 h, 56%

2-(Triphenyl-λ^5-phosphaneylidene)acetonitrile (72)[19]

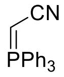

72

To a solution of (cyanomethyl)triphenylphosphonium chloride **71** (4.00 g, 11.8 mmol) in CH_2Cl_2 (60 mL) was added dropwise Et_3N (4.13 mL, 29.6 mmol). The reaction mixture was stirred for 30 min then water (30 mL) was added. The two layers were separated and the organic layer was washed with water (30 mL) once more. The organic layer was dried over $MgSO_4$ and concentrated *in vacuo*. Purification by recrystallisation ($CHCl_3$/pentane) afforded the title compound as a beige solid (3.00 g, 84%).

***R*f** = 0.1 ($CHCl_3$:MeOH, 95:5).

m.p. 195 °C

^{1}H NMR ($CDCl_3$, 400 MHz) δ = 7.63 – 7.38 (15H, m, Ar*H* × 15), 1.55 (1H, s, C*H*).

^{13}C NMR ($CDCl_3$, 126 MHz) δ = 132.6 (d, *J* = 10.0 Hz, C_{Ar}H × 6), 132.5 (d, *J* = 2.9 Hz, C_{Ar}H × 3), 129.0 (d, *J* = 12.6 Hz, C_{Ar}H × 6), 127.2 (d, *J* = 91.6 Hz, (C_{Ar} × 3), –2.2 (d, *J* = 135.9 Hz, *C*H).

^{31}P NMR (162 MHz, $CDCl_3$) δ = 23.1.

Analytical data are in accordance with those previously reported for this compound.[19]

3-(2,3-Dihydrobenzofuran-3-yl)-3-oxo-2-(triphenyl-λ^5-phosphaneylidene)propanenitrile (73)[17]

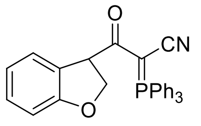

73

To a solution of acid **48** (34 mg, 210 μmol) in CH_2Cl_2 (1 mL) was added *N*-(3-dimethylaminopropyl)-*N*'-ethylcarbodiimide hydrochloride (60 mg, 310 μmol), 4-(dimethylamino)pyridine (3.0 mg, 21 μmol) and ylide **72** (75 mg, 250 μmol). The reaction mixture was stirred at rt for 16 h, diluted with CH_2Cl_2 (50 mL) and water (50 mL) and the layers separated. The aqueous layer was extracted with CH_2Cl_2 (3 × 30 mL) and the combined organic phase was washed with sat. aq. NaCl (30 mL), dried over $MgSO_4$ and concentrated *in vacuo*. Purification by flash column chromatography (SiO_2, Pentane:EtOAc, 60:40) afforded the title compound as a white solid (52 mg, 56%).

***R*f** = 0.6 (Pentane:EtOAc, 40:60).
m.p. 224 °C
^{1}H NMR ($CDCl_3$, 400 MHz) δ = 7.65 – 7.45 (15H, m, Ar*H* × 15), 7.14 (1H, t, *J* = 7.7 Hz, Ar*H*), 6.90 (1H, t, *J* = 7.5 Hz, Ar*H*), 6.77 (1H, d, *J* = 7.9 Hz, Ar*H*), 5.08 (1H, dd, *J* = 9.3, 6.1 Hz, C*H*), 4.91 (1H, dd, *J* = 8.7, 6.1 Hz, C$H_A$$H_B$), 4.66 (1H, t, *J* = 9.1 Hz, C$H_A$$H_B$).
^{13}C NMR ($CDCl_3$, 126 MHz) δ = 193.4 (d, *J* = 3.9 Hz, *C*(O)C=P), 160.2 (C_{Ar}), 133.6 (d, *J* = 10.1 Hz, C_{Ar}H × 6), 133.4 (d, *J* = 3.1 Hz, C_{Ar}H × 3), 129.3 (d, *J* = 12.8 Hz, C_{Ar}H × 6), 128.8 (C_{Ar}H), 127.3 (*C*≡N), 125.1 (C_{Ar}H), 122.9 (d, *J* = 93.6 Hz, C_{Ar} × 3), 122.7 (d, *J* = 16.0 Hz, C_{Ar}), 120.3 (C_{Ar}H), 109.8 (C_{Ar}H), 73.0 (CH_AH_B), 50.7 (d, *J* = 7.3 Hz, *C*H), 48.2 (d, *J* = 125.6 Hz, C(O)*C*=P).
^{31}P NMR (162 MHz, $CDCl_3$) δ = 20.5.
IR (film) ν_{max}/cm^{-1} 3003, 2173, 1595, 1481, 1108, 747, 666.
HRMS: (ESI$^+$) Found [M + H]$^+$ = 448.1457, $C_{29}H_{23}O_2NP$ requires 448.1461.

***tert*-Butyl 3-chloropropiolate (30)[1]**

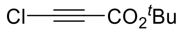

30

To a solution of tert-butyl propiolate (1.26 g, 10.0 mmol) and ^{t}BuOCl (1.09 g, 10.0 mmol) in ^{t}BuOH (5 mL) was added portionwise ^{t}BuOK (112 mg, 1.00 mmol). The reaction mixture was

stirred at rt for 1 h, then filtered through Celite® and the solvent removed *in vacuo*. Purification by flash column chromatography (SiO_2, Pentane) afforded the title compound as a colourless oil (768 mg, 48%).

***R*f** = 0.7 (Pentane:Et_2O, 90:10).
^{1}H NMR ($CDCl_3$, 400 MHz) δ = 1.48 (9H, s, $CO_2C(CH_3)_3$).
^{13}C NMR ($CDCl_3$, 101 MHz) δ = 151.4 (*C*$O_2C(CH_3)_3$), 84.3 (CO_2*C*$(CH_3)_3$), 66.1 (*C*≡CCl), 63.2 (C≡*C*Cl), 28.0 ($CO_2C($*C*$H_3)_3$).
IR (film) ν_{max}/cm^{-1} 2982, 2927, 2218, 1714, 1274, 1258, 1157.
HRMS: Ion not found

Methyl (*Z*)-3-(4-methoxy-4-oxobut-2-en-2-yl)-2,3-dihydrobenzofuran-3-carboxylate (31)[6]

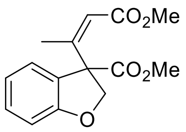

31

To a solution of CuI (143 mg, 753 μmol) in THF (2 mL) at –78 °C was added MeLi (940 μL, 1.51 mmol, 1.6 M in Et_2O). The mixture was stirred vigorously at 0 °C for 1 h before being cooled to –78 °C. A solution of alkyne **21** (98 mg, 377 μmol) in THF (1.5 mL + 0.5 mL) was added dropwise and the reaction mixture stirred at –78 °C for 3 h. Sat. aq. NH_4Cl (3 mL) was added and the mixture extracted with Et_2O (3 × 20 mL). The combined organic phase was washed with sat. aq. NaCl (20 mL), dried over $MgSO_4$ and concentrated *in vacuo*. Purification by flash column chromatography (SiO_2, Pentane:Et_2O, 80:20) afforded the title compound as a colourless oil and a single diastereomer (96 mg, 92%).

***R*f** = 0.2 (Pentane:Et_2O, 80:20).
^{1}H NMR ($CDCl_3$, 400 MHz) δ = 7.20 (2H, ddd, *J* = 15.8, 7.7, 1.3 Hz, Ar*H* × 2), 6.90 – 6.85 (1H, m, Ar*H*), 6.86 – 6.82 (1H, m, Ar*H*), 5.93 (1H, q, *J* = 1.5 Hz, C=C*H*), 5.58 (1H, d, *J* = 9.7 Hz, CH_AH_B), 4.42 (1H, d, *J* = 9.7 Hz, CH_AH_B), 3.69 (3H, s, CO_2CH_3), 3.68 (3H, s, CO_2CH_3), 1.77 (3H, d, *J* = 1.6 Hz, C*H*$_3$).
^{13}C NMR ($CDCl_3$, 101 MHz) δ = 171.5 (*C*O_2CH_3), 166.5 (*C*O_2CH_3), 160.5 (*C*=CH), 159.0 (C_{Ar}), 130.3 (C_{Ar}H), 126.6 (C_{Ar}), 125.9 (C_{Ar}H), 120.5 (C_{Ar}H), 117.9 (C=*C*H), 110.6 (C_{Ar}H), 81.9 (*C*H_AH_B), 61.5 (C_{Ar}*C*), 52.9 (CO_2*C*H_3), 51.5 (CO_2*C*H_3), 25.7 (*C*H_3).

IR (film) ν_{max}/cm^{-1} 2952, 1732, 1717, 1481, 1230, 1154, 753.
HRMS: (APCI$^+$) Found $[M + H]^+$ = 277.1069, $C_{15}H_{17}O_5$ requires 277.1071.

Methyl (*Z*)-3-(1-methoxy-1-oxohept-2-en-3-yl)-2,3-dihydrobenzofuran-3-carboxylate (32)[6]

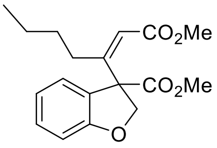

32

To a solution of CuI (76 mg, 400 µmol) in THF (1.5 mL) at –78 °C was added ^{n}BuLi (320 µL, 800 µmol, 2.5 M in hexane). The mixture was stirred vigorously at 0 °C for 1 h before being cooled to –78 °C. A solution of alkyne **21** (52 mg, 200 µmol) in THF (0.75 mL + 0.25 mL) was added dropwise and the reaction mixture stirred at –78 °C for 3 h. Sat. aq. NH_4Cl (2 mL) was added and the mixture extracted with Et_2O (3 × 20 mL). The combined organic phase was washed with sat. aq. NaCl (20 mL), dried over $MgSO_4$ and concentrated *in vacuo*. Purification by flash column chromatography (SiO_2, Pentane:Et_2O, 85:15) afforded the title compound as a colourless oil and a single diastereomer (57 mg, 89%).

***R*$_f$** = 0.3 (Pentane:Et_2O, 70:30).
^{1}H NMR ($CDCl_3$, 500 MHz) δ = 7.22 (1H, td, *J* = 7.7, 1.4 Hz, Ar*H*), 7.18 (1H, dd, *J* = 7.6, 1.3 Hz, Ar*H*), 6.87 (1H, td, *J* = 7.6, 1.1 Hz, Ar*H*), 6.84 (1H, d, *J* = 8.2 Hz, Ar*H*), 5.86 (1H, t, *J* = 1.4 Hz, C=C*H*), 5.60 (1H, d, *J* = 9.8 Hz, CH_AH_B), 4.41 (1H, d, *J* = 9.8 Hz, CH_AH_B), 3.68 (6H, s, CO_2CH_3 × 2), 2.16 – 2.07 (1H, m, C=CCH_AH_B), 2.01 – 1.93 (1H, m, C=CCH_AH_B), 1.33 – 1.09 (4H, m, CH_2 × 2), 0.73 (3H, t, *J* = 7.1 Hz, CH_3).
^{13}C NMR ($CDCl_3$, 126 MHz) δ = 171.8 (CO_2CH_3), 166.9 (CO_2CH_3), 163.1 (*C*=CH), 160.9 (C_{Ar}), 130.3 (C_{Ar}H), 126.3 (C_{Ar}), 125.9 (C_{Ar}H), 120.5 (C_{Ar}H), 116.8 (C=*C*H), 110.5 (C_{Ar}H), 82.4 (CH_AH_B), 61.8 ($C_{Ar}C$), 52.9 (CO_2CH_3), 51.5 (CO_2CH_3), 37.2 (C=CCH_AH_B), 30.0 (CH_2), 22.3 (CH_2), 13.8 (CH_3).
IR (film) ν_{max}/cm^{-1} 2955, 1734, 1715, 1481, 1228, 974, 753.
HRMS: (EI$^+$) Found [M^+] = 318.1462, $C_{18}H_{22}O_5$ requires 318.1462.

Methyl (Z)-3-(1-methoxy-5-methyl-1-oxohex-2-en-3-yl)-2,3-dihydrobenzofuran-3-carboxylate (33)[6]

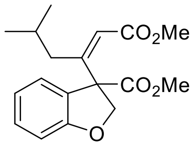

33

To a solution of CuI (76 mg, 400 μmol) in THF (1.5 mL) at –78 °C was added ^{i}BuLi (470 μL, 800 μmol, 1.7 M in hexane). The mixture was stirred vigorously at 0 °C for 1 h before being cooled to –78 °C. A solution of alkyne **21** (52 mg, 200 μmol) in THF (0.75 mL + 0.25 mL) was added dropwise and the reaction mixture stirred at –78 °C for 3 h. Sat. aq. NH_4Cl (2 mL) was added and the mixture extracted with Et_2O (3 × 20 mL). The combined organic phase was washed with sat. aq. NaCl (20 mL), dried over $MgSO_4$ and concentrated *in vacuo*. Purification by flash column chromatography (SiO_2, Pentane:Et_2O, 90:10) afforded the title compound as a colourless oil and a single diastereomer (58 mg, 91%).

$\boldsymbol{R_f}$ = 0.3 (Pentane:Et_2O, 70:30).
^{1}H NMR ($CDCl_3$, 500 MHz) δ = 7.24 – 7.16 (2H, m, Ar*H* × 2), 6.86 (1H, td, J = 7.6, 1.1 Hz, Ar*H*), 6.82 (1H, d, J = 8.1 Hz, Ar*H*), 5.83 (1H, s, C=C*H*), 5.60 (1H, d, J = 9.8 Hz, CH_AH_B), 4.40 (1H, d, J = 9.8 Hz, CH_AH_B), 3.68 (3H, s, CO_2CH_3), 3.68 (3H, s, CO_2CH_3), 2.15 (1H, ddd, J = 14.2, 5.1, 1.2 Hz, C=CCH_AH_B), 1.83 (1H, ddd, J = 14.2, 8.6, 1.1 Hz, C=CCH_AH_B), 1.36 – 1.22 (1H, m, C*H*$(CH_3)_A(CH_3)_B$), 0.76 (3H, d, J = 6.6 Hz, $CH(CH_3)_A(CH_3)_B$), 0.61 (3H, d, J = 6.6 Hz, $CH(CH_3)_A(CH_3)_B$).
^{13}C NMR ($CDCl_3$, 126 MHz) δ = 171.8 (CO_2CH_3), 166.6 (CO_2CH_3), 161.6 (*C*=CH), 160.9 (C_{Ar}), 130.3 (C_{Ar}H), 126.4 (C_{Ar}), 126.0 (C_{Ar}H), 120.4 (C_{Ar}H), 118.3 (C=*C*H), 110.5 (C_{Ar}H), 82.4 (CH_AH_B), 61.6 ($C_{Ar}C$), 52.9 (CO_2CH_3), 51.5 (CO_2CH_3), 47.1 (C=CCH_AH_B), 26.0 ($CH(CH_3)_A(CH_3)_B$), 22.8 ($CH(CH_3)_A(CH_3)_B$), 21.6 ($CH(CH_3)_A(CH_3)_B$).
IR (film) ν_{max}/cm^{-1} 2955, 1735, 1715, 1481, 1234, 975, 753.
HRMS: (ESI$^+$) Found $[M + H]^+$ = 319.1534, $C_{18}H_{23}O_5$ requires 319.1540.

Methyl (*E*)-3-(1-methoxy-1-oxoocta-2,7-dien-3-yl)-2,3-dihydrobenzofuran-3-carboxylate (74)[6]

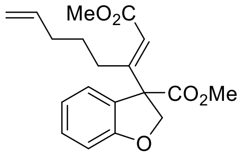

74

To a solution of iodide **44** (157 mg, 800 µmol) in pentane (3 mL) and Et_2O (2 mL) at –78 °C was added ^{t}BuLi (940 µL, 1.6 mmol, 1.7 M in pentane). After 10 min CuI (76 mg, 400 µmol) was added and the mixture was stirred vigorously at 0 °C for 1 h before being cooled to –78 °C. A solution of alkyne **21** (52 mg, 200 µmol) in Et_2O (0.75 mL + 0.25 mL) was added dropwise and the reaction mixture stirred at –78 °C for 30 min. Sat. aq. NH_4Cl (1 mL) was added and the mixture partitioned between water (15 mL) and Et_2O (20 mL). The layers were separated and the aqueous layer was extracted with Et_2O (3 × 20 mL). The combined organic phase was washed with sat. aq. NaCl (20 mL), dried over $MgSO_4$ and concentrated *in vacuo*. Purification by flash column chromatography (SiO_2, Pentane:Et_2O, 92:8) afforded the title compound (13 mg, 19%) as a colourless oil, in addition to alkene **75** (36 mg, 54%), separable by column chromatography.

Data for **74**

***R*f** = 0.7 (Pentane:Et_2O, 70:30).

^{1}H NMR ($CDCl_3$, 500 MHz) δ = 7.41 (1H, dd, *J* = 7.7, 1.4 Hz, Ar*H*), 7.27 – 7.22 (1H, m, Ar*H*), 6.95 (1H, td, *J* = 7.5, 1.1 Hz, Ar*H*), 6.84 (1H, dt, *J* = 8.0, 0.8 Hz, Ar*H*), 5.81 – 5.68 (1H, m, CH_2=C*H*), 5.62 (1H, s, C=C*H*), 5.25 (1H, d, *J* = 9.4, CH_AH_B), 5.04 – 4.93 (2H, m, CH_2=CH), 4.41 (1H, d, *J* = 9.4, 1.0 Hz, CH_AH_B), 3.78 (3H, s, CO_2CH_3), 3.66 (3H, s, CO_2CH_3), 2.59 – 2.51 (2H, m, C=CCH_2), 2.13 – 2.04 (2H, m, CH_2=CHCH_2), 1.56 – 1.40 (2H, m, CH_2=CHCH_2CH_2).

^{13}C NMR ($CDCl_3$, 126 MHz) δ = 171.3 (CO_2CH_3), 166.4 (CO_2CH_3), 160.8 (*C*=CH), 160.5 (C_{Ar}), 138.1 (CH_2=*C*H), 130.5 (C_{Ar}H), 127.3 (C_{Ar}H), 124.9 (C_{Ar}), 121.1 (C_{Ar}H), 118.5 (C=*C*H), 115.2 (*C*H_2=CH), 110.6 (C_{Ar}H), 78.3 (CH_AH_B), 65.1 ($C_{Ar}C$), 531 (CO_2CH_3), 51.4 (CO_2CH_3), 34.4 (CH_2=CH*C*H_2), 30.8 (C=CCH_AH_B), 28.7 (CH_2=CH$CH_2$$CH_AH_B$).

IR (film) ν_{max}/cm^{-1} 2980, 1737, 1722, 1640, 1481, 1225, 1171, 754.

HRMS: (ESI$^+$) Found $[M + H]^+$ = 331.1541; $C_{19}H_{23}O_5$ requires 331.1540.

Methyl (*Z*)-3-(1-methoxy-1-oxoocta-2,7-dien-3-yl)-2,3-dihydrobenzofuran-3-carboxylate (75)[57]

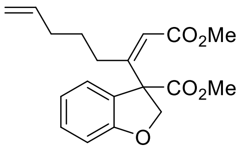

75

This compound was prepared according to the above procedure for alkene **74**.

***R*f** = 0.3 (Pentane:Et_2O, 70:30).

^{1}H NMR ($CDCl_3$, 500 MHz) δ = 7.24 – 7.14 (2H, m, Ar*H* × 2), 6.93 – 6.79 (2H, m, Ar*H* × 2), 5.87 (1H, t, *J* = 1.4 Hz, C=C*H*), 5.60 (1H, d, *J* = 9.8 Hz, CH_AH_B), 5.57 – 5.43 (1H, m, CH_2=C*H*), 4.95 – 4.80 (2H, m, CH_2=CH), 4.41 (1H, d, *J* = 9.8 Hz, CH_AH_B), 3.68 (6H, s, CO_2CH_3 × 2), 2.16 – 1.96 (2H, m, C=CCH_AH_B and C=CCH_AH_B), 1.95 – 1.79 (2H, m, CH_2=CHCH_2), 1.39 – 1.24 (2H, m, CH_2=CH$CH_2$$CH_AH_B$ and CH_2=CH$CH_2$$CH_AH_B$).

^{13}C NMR ($CDCl_3$, 126 MHz) δ = 171.7 (CO_2CH_3), 166.8 (CO_2CH_3), 162.9 (*C*=CH), 160.8 (C_{Ar}), 137.8 (CH_2=*C*H), 130.4 (C_{Ar}H), 126.3 (C_{Ar}), 125.9 (C_{Ar}H), 120.5 (C_{Ar}H), 117.0 (C=*C*H), 115.3 (CH_2=CH), 110.6 (C_{Ar}H), 82.5 (CH_AH_B), 61.7 ($C_{Ar}C$), 52.9 (CO_2CH_3), 51.5 (CO_2CH_3), 36.9 (C=CCH_AH_B), 33.2 (CH_2=CHCH_2), 27.1 (CH_2=CH$CH_2$$CH_AH_B$).

IR (film) ν_{max}/cm^{-1} 2980, 2360, 2341, 1734, 1717, 1481, 1231, 754.

HRMS: (ESI$^+$) Found $[M + Na]^+$ = 353.1360; $C_{19}H_{22}O_5Na$ requires 353.1359.

2-Bromo-6-hydroxybenzaldehyde (36)[7]

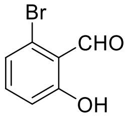

36

To a suspension of NaH (1.20 g, 30.0 mmol, 60 wt% in mineral oil) in THF (30 mL) at rt was added dropwise 3-bromophenol (2.60 g, 15.0 mmol) in THF (5 mL). The solution was stirred for 15 min and diethylcarbamoyl chloride (2.76 mL, 30.0 mL) was added in THF (3 mL). The reaction mixture was stirred for 16 h and water (30 mL) was added at 0 °C. After warming to rt, Et_2O (30 mL) was added and the layers separated. The aqueous layer was extracted with Et_2O (3 × 20 mL) and the combined organic phase was washed with sat. aq. NaCl (20 mL), dried over $MgSO_4$ and concentrated *in vacuo*. Separately, diisopropylamine (3.15 mL,

22.5 mmol) in THF (30 mL) was cooled to 0 °C and ^{n}BuLi (9.00 mL, 22.5 mmol, 2.5 M hexanes) was added dropwise and the solution stirred for 30 min at 0 °C, then cooled to –78 °C. The residue from the previous step was dissolved in THF (5 mL) and added dropwise to the solution. The reaction mixture was stirred at –78 °C for 30 min, then DMF (3.48 mL, 45.0 mmol) was added dropwise. The mixture was stirred for a further 30 min at –78 °C, then 30 min at rt. The reaction mixture was cooled to 0 °C and HCl (15 mL, 30 mmol, 2 M aq.) was added. The solution was stirred at rt for 20 min and KOH (20 mL, 40 mmol, 2 M aq.) was added followed by Et_2O (100 mL). The two layers were separated and HCl (25 mL, 50 mmol, 2 M aq.) was added to the aqueous layer followed by Et_2O (100 mL). The layers were again separated and the aqueous layer extracted with Et_2O (3 × 100 mL) and the combined organic phase was washed with sat. aq. NaCl (50 mL), dried over $MgSO_4$ and concentrated *in vacuo* to afford the title compound (2.07 g, 69%) with no further purification necessary.

***R*f** = 0.7 (Pentane:Et_2O, 80:20).
m.p. 55 °C
^{1}H NMR ($CDCl_3$, 400 MHz) δ = 12.00 (1H, s, C*H*O), 10.35 (1H, d, *J* = 0.6 Hz, O*H*), 7.35 (1H, dd, *J* = 8.5, 7.8 Hz, Ar*H*), 7.18 (1H, dd, *J* = 7.8, 1.0 Hz, Ar*H*), 6.96 (1H, dt, *J* = 8.5, 0.8 Hz, Ar*H*).
^{13}C NMR ($CDCl_3$, 101 MHz) δ = 198.0 (*C*HO), 164.1 (C_{Ar}), 137.7 (C_{Ar}H), 127.6 (C_{Ar}), 124.6 (C_{Ar}H), 118.0 (C_{Ar}H).
Analytical data are in accordance with those previously reported for this compound.[7]

Ethyl 4-bromobenzofuran-3-carboxylate (37)[3]

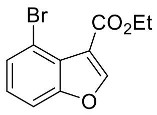

37

To a solution of aldehyde **36** (2.00 g, 9.94 mmol) and $HBF_4{\cdot}OEt_2$ (137 µL, 995 µmol) in CH_2Cl_2 (10 mL) at rt was added dropwise a solution of ethyl diazoacetate (1.97 mL, 15.9 mmol) in CH_2Cl_2 (20 mL) over a period of 10 min. The reaction mixture was stirred at room temperature for 30 min and then concentrated *in vacuo* after nitrogen evolution had ceased. conc. H_2SO_4 (2.00 mL) was added and the reaction mixture was stirred at room temperature for 30 min. CH_2Cl_2 (30 mL) was added followed by solid $NaHCO_3$ until gas evolution had ceased. The solution was washed successively with water (30 mL), 1 M aq. NaOH (20 mL) and sat. aq.

NaCl (50 mL), dried over $MgSO_4$ and concentrated *in vacuo*. Purification by flash column chromatography (SiO_2, Pentane:Et_2O, 90:10) afforded the title compound as a colourless oil (2.26 g, 84%).

***R*f** = 0.7 (Pentane:Et_2O, 60:40).
m.p. 45 °C
^{1}H NMR ($CDCl_3$, 400 MHz) δ = 8.21 (1H, s, Ar*H*), 7.55 (1H, dd, *J* = 7.8, 0.9 Hz, Ar*H*), 7.49 (1H, dd, *J* = 8.3, 0.9 Hz, Ar*H*), 7.20 (1H, t, *J* = 8.1 Hz, Ar*H*), 4.40 (2H, q, *J* = 7.1 Hz, $CO_2CH_2CH_3$), 1.41 (3H, t, *J* = 7.2 Hz, $CO_2CH_2CH_3$).
^{13}C NMR ($CDCl_3$, 101 MHz) δ = 162.4 ($CO_2CH_2CH_3$), 156.3 (C_{Ar}), 151.4 (C_{Ar}H), 129.5 (C_{Ar}H), 126.3 (C_{Ar}H), 124.6 (C_{Ar}), 115.8 (C_{Ar}), 114.3 (C_{Ar}), 111.2 (C_{Ar}H), 61.3 ($CO_2CH_2CH_3$), 14.4 ($CO_2CH_2CH_3$).
IR (film) ν_{max}/cm^{-1} 2982, 1734, 1559, 1126, 897, 761.
HRMS: (APCI$^+$) Found $[M]^+$ = 268.9811, $C_{11}H_{10}O_3Br$ requires 268.9808.

Ethyl 4-(tributylstannyl)benzofuran-3-carboxylate (40)[8]

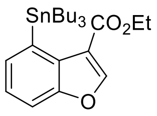

40

To a solution of benzofuran **37** (81 mg, 300 μmol) in dioxane (2 mL) in a microwave vial was added successively $Pd_2(dba)_3$ (7.0 mg, 7.5 μmol), JohnPhos (13 mg, 45 μmol) and bis(tributyltin) (230 μL, 450 μmol). The reaction mixture was heated at 110 °C for 16 h, filtered through Celite® and concentrated *in vacuo*. Purification by flash column chromatography (SiO_2, Pentane:Et_3N, 99:1) afforded the title compound as a colourless oil (93 mg, 65%).

***R*f** = 0.8 (Pentane:Et_2O, 95:5).
^{1}H NMR ($CDCl_3$, 400 MHz) δ = 8.23 (1H, s, Ar*H*), 7.52 (1H, dd, *J* = 7.1, 1.0 Hz, Ar*H*), 7.48 (1H, dd, *J* = 8.3, 1.0 Hz, Ar*H*), 7.31 (1H, dd, *J* = 8.3, 7.0 Hz, Ar*H*), 4.36 (2H, q, *J* = 7.1 Hz, $CO_2CH_2CH_3$), 1.58 – 1.46 (6H, m, $SnCH_2CH_2CH_2CH_3$ × 3), 1.39 (3H, t, *J* = 7.1 Hz, $CO_2CH_2CH_3$), 1.32 (6H, q, *J* = 7.3 Hz, $SnCH_2CH_2CH_2CH_3$ × 3), 1.17 – 1.02 (6H, m, $SnCH_2CH_2CH_2CH_3$ × 3), 0.87 (9H, t, *J* = 7.3 Hz, $SnCH_2CH_2CH_2CH_3$ × 3).
^{13}C NMR ($CDCl_3$, 101 MHz) δ = 163.5 ($CO_2CH_2CH_3$), 155.2 (C_{Ar}), 150.9 (C_{Ar}H), 137.1 (C_{Ar}), 133.6 (C_{Ar}H), 130.9 (C_{Ar}), 124.6 (C_{Ar}H), 115.8 (C_{Ar}), 111.6 (C_{Ar}H), 60.5 ($CO_2CH_2CH_3$), 29.3

($SnCH_2CH_2CH_2CH_3$ × 3), 27.6 ($SnCH_2CH_2CH_2CH_3$ × 3), 14.6 ($CO_2CH_2CH_3$), 13.9 ($SnCH_2CH_2CH_2CH_3$ × 3), 12.6 ($SnCH_2CH_2CH_2CH_3$ × 3).
IR (film) ν_{max}/cm^{-1} 2955, 2922, 2871, 2853, 1720, 1405, 1280, 1139, 766.
HRMS: (APCI$^-$) Found $[M - H]^-$ = 479.1609, $C_{23}H_{35}O_3Sn$ requires 479.1614.

Methyl 4-(tributylstannyl)-2,3-dihydrobenzofuran-3-carboxylate (41)[4]

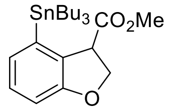

41

Mg turnings (170 mg, 6.99 mmol) were added to a solution of benzofuran **40** (670 mg, 1.40 mmol) in MeOH (9 mL) at rt. After 4 h the reaction was heated at 70 °C for 1 h. CH_2Cl_2 (100 mL) and water (100 mL) were added and the layers separated. The aqueous layer was extracted with CH_2Cl_2 (2 × 100 mL) and the combined organic phase was washed with sat. aq. NaCl (50 mL), dried over $MgSO_4$ and concentrated *in vacuo*. Purification by flash column chromatography (SiO_2, Pentane:Et_2O:Et_3N, 95:4.5:0.5) afforded the title compound as a colourless oil (621 mg, 92%).

***R*f** = 0.3 (Pentane:Et_2O, 95:5).
^{1}H NMR ($CDCl_3$, 400 MHz) δ = 7.15 (1H, t, *J* = 7.8 Hz, Ar*H*), 7.03 – 6.90 (1H, m, Ar*H*), 6.79 (1H, dd, *J* = 8.0, 1.1 Hz, Ar*H*), 4.85 (1H, dd, *J* = 9.2, 3.2 Hz, CH_AH_B), 4.57 (1H, t, *J* = 9.2 Hz, CH_AH_B), 4.13 (1H, dd, *J* = 9.1, 3.1 Hz, C*H*), 3.70 (3H, s, CO_2CH_3), 1.55 – 1.43 (6H, m, $SnCH_2CH_2CH_2CH_3$ × 3), 1.39 – 1.28 (6H, m, $SnCH_2CH_2CH_2CH_3$ × 3), 1.16 – 0.98 (6H, m, $SnCH_2CH_2CH_2CH_3$ × 3), 0.89 (9H, t, *J* = 7.3 Hz, $SnCH_2CH_2CH_2CH_3$ × 3).
^{13}C NMR ($CDCl_3$, 101 MHz) δ = 172.6 (CO_2CH_3), 159.3 (C_{Ar}), 140.0 (C_{Ar}), 131.6 (C_{Ar}), 129.4 ($C_{Ar}H$), 128.8 ($C_{Ar}H$), 110.1 ($C_{Ar}H$), 73.1 (CH_AH_B), 52.7 (CO_2CH_3), 49.2 (*C*H), 29.3 ($SnCH_2CH_2CH_2CH_3$ × 3), 27.6 ($SnCH_2CH_2CH_2CH_3$ × 3), 13.8 ($SnCH_2CH_2CH_2CH_3$ × 3), 10.4 ($SnCH_2CH_2CH_2CH_3$ × 3).
IR (film) ν_{max}/cm^{-1} 2955, 2922, 2871, 2852, 1735, 1569, 1421, 1246, 1213, 982, 775.
HRMS: (ESI$^+$) Found $[M + Na]^+$ = 491.1583, $C_{22}H_{36}O_3NaSn$ requires 491.1579.

Methyl 3-(3-methoxy-3-oxoprop-1-yn-1-yl)-4-(tributylstannyl)-2,3-dihydrobenzofuran-3-carboxylate (42)

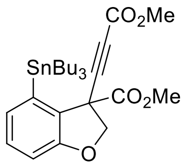

42

To a stirred solution of ester **41** (657 mg, 1.41 mmol) in THF (12 mL) at –78 °C was added LiHMDS (1.69 mL, 1.69 mmol, 1 M in THF). After 10 min chloroacetylene **20** (250 mg, 2.11 mmol) in THF (0.5 mL) was added dropwise and the reaction mixture was stirred at –78 °C for 1 h. Sat. aq. NH_4Cl (5 mL) was added and the mixture extracted with Et_2O (3 × 50 mL). The combined organic phase was washed with sat. aq. NaCl (50 mL), dried over $MgSO_4$ and concentrated *in vacuo*. Purification by flash column chromatography (SiO_2, Pentane:Et_2O:Et_3N, 93:6.5:0.5) afforded the title compound as a colourless oil (368 mg, 48%).

$\boldsymbol{R_f}$ = 0.2 (Pentane:Et_2O, 95:5).

^{1}H NMR ($CDCl_3$, 400 MHz) δ = 7.20 (1H, t, *J* = 7.2 Hz, Ar*H*), 7.06 (1H, dd, *J* = 7.3, 1.1 Hz, Ar*H*), 6.81 (1H, dd, *J* = 8.0, 1.2 Hz, Ar*H*), 4.89 (1H, d, *J* = 9.1 Hz, CH_AH_B), 4.72 (1H, d, *J* = 9.1 Hz, CH_AH_B), 3.79 (3H, s, CO_2CH_3), 3.77 (3H, s, CO_2CH_3), 1.58 – 1.43 (6H, m, $SnCH_2CH_2CH_2CH_3$ × 3), 1.36 – 1.28 (6H, m, $SnCH_2CH_2CH_2CH_3$ × 3), 1.21 – 1.04 (6H, m, $SnCH_2CH_2CH_2CH_3$ × 3), 0.89 (9H, t, *J* = 7.3 Hz, $SnCH_2CH_2CH_2CH_3$ × 3).

^{13}C NMR ($CDCl_3$, 101 MHz) δ = 169.6 ($CO_2CH_2CH_3$), 159.3 (C_{Ar}), 153.5 (CO_2CH_3), 140.5 (C_{Ar}), 131.9 (C_{Ar}), 130.5 (C_{Ar}H), 129.6 (C_{Ar}H), 110.6 (C_{Ar}H), 85.1 ($C{\equiv}CCO_2CH_3$), 80.1 (CH_AH_B), 77.5 ($C{\equiv}CCO_2CH_3$), 53.8 (CO_2CH_3), 53.2 ($C_{Ar}C$), 52.8 (CO_2CH_3), 29.2 ($SnCH_2CH_2CH_2CH_3$ × 3), 27.5 ($SnCH_2CH_2CH_2CH_3$ × 3), 13.7 ($SnCH_2CH_2CH_2CH_3$ × 3), 11.1 ($SnCH_2CH_2CH_2CH_3$ × 3).

IR (film) ν_{max}/cm^{-1} 2955, 2924, 2871, 2853, 2242, 1746,

HRMS: (ESI$^+$) Found $[M + H]^+$ = 551.1815, $C_{26}H_{39}O_5Sn$ requires 551.1814.

Methyl 4-iodo-3-(3-methoxy-3-oxoprop-1-yn-1-yl)-2,3-dihydrobenzofuran-3-carboxylate (43)

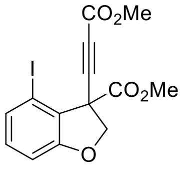

43

To a solution of stannane **42** (100 mg, 190 μmol) in $CHCl_3$ (10 mL) was added dropwise iodine (9.5 mL, 228 μmol, 0.024 M in $CHCl_3$). The reaction mixture was stirred for 1 h and sat. aq. $Na_2S_2O_3$ (10 mL) was added. The mixture was extracted with $CHCl_3$ (3 × 30 mL). The combined organic phase was washed with sat. aq. NaCl (30 mL), dried over $MgSO_4$ and concentrated *in vacuo*. Purification by flash column chromatography (SiO_2, Pentane:Et_2O, 80:20) afforded the title compound as a colourless oil (64 mg, 87%).

$\boldsymbol{R_f}$ = 0.3 (Pentane:Et_2O, 70:30).
^{1}H NMR ($CDCl_3$, 400 MHz) δ = 7.35 (1H, dd, J = 7.8, 1.0 Hz, ArH), 6.96 (1H, t, J = 8.0 Hz, ArH), 6.87 (1H, dd, J = 8.2, 1.1 Hz, ArH), 4.89 (1H, d, J = 9.1 Hz, C$H_A$$H_B$), 4.85 (2H, d, J = 9.1 Hz, C$H_A$$H_B$), 3.86 (3H, s, $CO_2C\mathit{H}_3$), 3.79 (3H, s, $CO_2C\mathit{H}_3$).
^{13}C NMR ($CDCl_3$, 126 MHz) δ = 168.7 ($\mathit{C}O_2CH_3$), 160.1 ($\mathit{C}O_2CH_3$), 153.7 (C_{Ar}), 132.2 (C_{Ar}H), 132.1 (C_{Ar}H), 129.4 (C_{Ar}), 110.9 (C_{Ar}H), 91.5 (C_{Ar}), 82.7 ($\mathit{C}{\equiv}CCO_2CH_3$), 80.6 ($\mathit{C}H_AH_B$), 78.3 ($C{\equiv}\mathit{C}CO_2CH_3$), 55.3 ($C_{Ar}\mathit{C}$), 54.1 ($CO_2\mathit{C}H_3$), 53.1 ($CO_2\mathit{C}H_3$).
IR (film) ν_{max}/cm^{-1} 2955, 1745, 1716, 1436, 1287, 1247, 872.
HRMS: (ESI$^+$) Found $[M + Na]^+$ = 408.9543, $C_{14}H_{11}O_5INa$ requires 408.9543.

5-Iodopent-1-ene (44)[9]

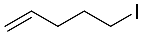

44

To a solution of 4-penten-1-ol (1.03 mL, 10.0 mmol) in CH_2Cl_2 (30 mL) was added PPh_3 (3.41 g, 13.0 mmol) and imidazole (885 mg, 13.0 mmol). The mixture was cooled to 0 °C and iodine (3.05 g, 12.0 mmol) was added. The reaction mixture was stirred for 1 h at 0 °C and then concentrated *in vacuo*. Pentane (150 mL) was added and the mixture was filtered through Celite®. Distillation of the residue (bp 50 °C, 30 mbar) afforded the title compound as a colourless oil (1.30 g, 67%).

***R*f** = 0.7 (Pentane:Et_2O, 90:10).

^{1}H NMR ($CDCl_3$, 400 MHz) δ = 5.75 (1H, ddt, *J* = 17.0, 10.1, 6.7 Hz, C*H*=CH_AH_B), 5.08 (1H, dq, *J* = 17.1, 1.7 Hz, CH=C$H_A$$H_B$), 5.02 (1H, ddt, *J* = 10.2, 2.0, 1.2 Hz, CH=$CH_A$$H_B$,), 3.19 (2H, t, *J* = 6.9 Hz, CH_2I), 2.21 – 2.11 (2H, m, CH_2CH=CH_AH_B), 1.91 (2H, p, *J* = 6.9 Hz, C$H_2$$CH_2$I).

^{13}C NMR ($CDCl_3$, 126 MHz) δ = 136.7 (*C*H=CH_AH_B), 116.1 (CH=*C*H_AH_B), 34.4 (*C*H_2CH=CH_AH_B), 32.6 (*C*$H_2$$CH_2$I), 6.5 (*C*$H_2$I).

Analytical data are in accordance with those previously reported for this compound.[9]

Methyl (*Z*)-4-iodo-3-(1-methoxy-1-oxoocta-2,7-dien-3-yl)-2,3-dihydrobenzofuran-3-carboxylate (45)[6]

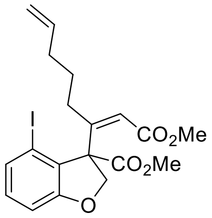

45

To a solution of iodide **44** (282 mg, 1.44 mmol) in pentane (5.3 mL) and Et_2O (3.7 mL) at –78 °C was added *^{t}*BuLi (1.69 mL, 1.6 mmol, 1.7 M in pentane). After 10 min CuI (137 mg, 720 µmol) was added and the mixture was stirred vigorously at 0 °C for 1 h before being cooled to –78 °C. A solution of alkyne **43** (139 mg, 360 µmol) in Et_2O (0. 5 mL + 0.2 mL) was added dropwise and the reaction mixture stirred at –78 °C for 1.5 h. Methanol (0.5 mL) was added and the mixture partitioned between water (15 mL) and Et_2O (20 mL). The layers were separated and the aqueous layer was extracted with Et_2O (3 × 20 mL). The combined organic phase was washed with sat. aq. NaCl (20 mL), dried over $MgSO_4$ and concentrated *in vacuo*. Purification by flash column chromatography (SiO_2, Pentane:Et_2O, 85:15) afforded the title compound as a colourless oil and a single diastereomer (74 mg, 45%). The analytical data for this sample is presented below:

***R*f** = 0.5 (Pentane:Et_2O, 60:40).

^{1}H NMR ($CDCl_3$, 400 MHz) δ = 7.26 (1H, dd, *J* = 7.5, 1.3 Hz, Ar*H*), 6.86 – 6.75 (2H, m, Ar*H* × 2), 5.90 (1H, t, *J* = 1.4 Hz, C=C*H*), 5.55 – 5.47 (1H, m, CH_AH_B=C*H*), 5.44 (1H, d, *J* = 9.7 Hz, C$H_A$$H_B$), 4.86 – 4.77 (2H, m, C$H_2$=CH), 4.45 (1H, d, *J* = 9.7 Hz, $CH_A$$H_B$), 3.62 (3H, s,

CO_2CH_3), 3.61 (3H, s, CO_2CH_3), 2.32 – 1.92 (2H, m, CH_2C=CH), 1.92 – 1.74 (2H, m, CH_2=CHCH_2), 1.44 – 1.20 (2H, m, CH_2=CHCH_2CH_2).

^{13}C NMR ($CDCl_3$, 126 MHz) δ = 170.5 (CO_2CH_3), 167.1 (CO_2CH_3), 161.9 (*C*=CH), 159.7 (C_{Ar}), 137.7 (CH_2=*C*H), 132.7 (C_{Ar}H), 131.4 (C_{Ar}H), 130.4, 118.5 (C=*C*H), 115.3 (CH_2=CH), 110.6 (C_{Ar}H), 93.2 (C_{Ar}), 84.1 (CH_AH_B), 64.3 ($C_{Ar}C$), 52.5 (CO_2CH_3), 51.5 (CO_2CH_3), 36.5 (C=CCH_2), 33.2 (CH_2=CHCH_2), 27.5 (CH_2=CHCH_2CH_2).

IR (film) ν_{max}/cm^{-1} 2949, 1735, 1714, 1634, 1594, 1434, 1253, 1230, 870.

HRMS: (ESI$^+$) Found [M + Na]$^+$ = 479.0326, $C_{19}H_{21}O_5INa$ requires 479.0326.

A small sample of the title compound was purified further using fine silica gel (0.015-0.040 mm) eluting with pentane:Et_2O, 90:10 to obtain an analytical sample. The spectroscopic data for this sample is presented below:

^{1}H NMR ($CDCl_3$, 400 MHz) δ = 7.33 (1H, dd, J = 7.5, 1.3 Hz, Ar*H*), 6.94 – 6.80 (2H, m, Ar*H* × 2), 5.97 (1H, t, J = 1.5 Hz, C=C*H*), 5.64 – 5.50 (1H, m, CH_AH_B=C*H*), 5.51 (1H, d, J = 9.7 Hz, CH_AH_B), 4.92 – 4.85 (2H, m, CH_2=CH), 4.52 (1H, d, J = 9.7 Hz, CH_AH_B), 3.69 (3H, s, CO_2CH_3), 3.68 (3H, s, CO_2CH_3), 2.39 – 2.00 (2H, m, CH_2C=CH), 1.99 – 1.78 (2H, m, CH_2=CHCH_2), 1.51 – 1.30 (2H, m, CH_2=CHCH_2CH_2).

^{13}C NMR ($CDCl_3$, 151 MHz) δ = 170.5 (CO_2CH_3), 167.2 (CO_2CH_3), 162.0 (*C*=CH), 159.8 (C_{Ar}), 137.8 (CH_2=*C*H), 132.8 (C_{Ar}H), 131.5 (C_{Ar}H), 130.4, 118.5 (C=*C*H), 115.4 (CH_2=CH), 110.7 (C_{Ar}H), 93.2 (C_{Ar}), 84.1 (CH_AH_B), 64.3 ($C_{Ar}C$), 52.6 (CO_2CH_3), 51.6 (CO_2CH_3), 36.5 (C=CCH_2), 33.2 (CH_2=CHCH_2), 27.6 (CH_2=CHCH_2CH_2).

*^{1}H and ^{13}C NMR spectra of both samples are shown on pages S59 and S60.

Methyl (*Z*)-10-(2-methoxy-2-oxoethylidene)-6-methylene-7,8,9,10-tetrahydro-1*H*-cycloocta[*cd*]benzofuran-10a(6*H*)-carboxylate (46)[10]

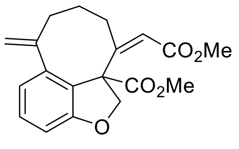

46

To a solution of alkene **45** (24 mg, 53 μmol) in MeCN (1 mL) was added triphenylphosphine (5.5 mg, 21 μmol), $Pd(OAc)_2$ (1.2 mg, 5.3 μ mol) and Et_3N (73 μL, 530 μmol). The reaction mixture was heated at 80 °C for 2 h. EtOAc (20 mL) was added and the reaction mixture filtered

through Celite®. Purification by flash column chromatography (SiO_2, Pentane:Et_2O, 90:10) afforded the title compound as a colourless oil and a single diastereomer (14 mg, 81%).

***R*f** = 0.5 (Pentane:Et_2O, 60:40).

^{1}H NMR ($CDCl_3$, 400 MHz) δ = 7.17 (1H, t, J = 7.9 Hz, Ar*H*), 6.84 (1H, dd, J = 7.6, 1.1 Hz, Ar*H*), 6.79 (1H, dd, J = 8.1, 1.1 Hz, Ar*H*), 5.95 (1H, s, C=C*H*), 5.61 (1H, d, J = 9.7 Hz, CH_AH_B), 5.06 – 5.02 (1H, m, C=CH_AH_B), 4.96 (1H, t, J = 1.8 Hz, C=CH_AH_B), 4.30 (1H, d, J = 9.7 Hz, CH_AH_B), 3.69 (3H, s, CO_2CH_3), 3.59 (3H, s, CO_2CH_3), 3.28 – 3.18 (1H, m, CH_AH_BC=CH), 2.39 – 2.15 (3H, m, $CH_2C=CH_2$ and CH_AH_BC=CH), 1.94 – 1.78 (2H, m, $CH_2CH_2C=CH_2$).

^{13}C NMR ($CDCl_3$, 126 MHz) δ = 171.0 (CO_2CH_3), 166.5 (CO_2CH_3), 162.7 (C_{Ar}), 160.8 (*C*=CH), 148.4 (C_{Ar}), 141.3 (*C*=CH_2), 130.1 (C_{Ar}H), 123.9, 121.9 (C_{Ar}H), 119.3 (C=*C*H), 115.8 (C=CH_2), 109.7 (C_{Ar}H), 81.9 (CH_AH_B), 62.4 ($C_{Ar}C$), 52.4 (CO_2CH_3), 51.5 (CO_2CH_3), 34.6 ($CH_2C=CH_2$), 34.1 (CH_AH_BC=CH), 28.5 ($CH_2CH_2C=CH_2$).

IR (film) ν_{max}/cm^{-1} 2950, 1738, 1716, 1573, 1435, 1256, 1230, 1145.

HRMS: (ESI^+) Found $[M + Na]^+$ = 351.1203, $C_{19}H_{20}O_5Na$ requires 351.1203.

Methyl (*Z*)-10-(2-methoxy-2-oxoethylidene)-6-oxo-7,8,9,10-tetrahydro-1*H*-cycloocta[cd]benzofuran-10a(6*H*)-carboxylate (47)[11]

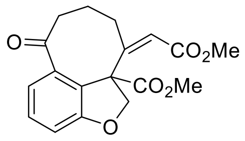

47

To a solution of alkene **46** (13 mg, 40 μmol) in THF (1.2 mL) and water (0.6 mL) was added OsO_4 (500 μg, 2.0 μmol) and $NaIO_4$ (42 mg, 200 μmol). The reaction mixture was stirred for 2 h then sat. aq. Na_2SO_3 (30 mL) was added followed by EtOAc (30 mL). The layers were separated and the aqueous layer was extracted with EtOAc (3 × 30 mL). The combined organic phase was washed with sat. aq. NaCl (20 mL), dried over $MgSO_4$ and concentrated *in vacuo*. Purification by flash column chromatography (SiO_2, Pentane:Et_2O, 70:30) afforded the title compound as a colourless oil (9 mg, 69%).

***R*f** = 0.5 (Pentane:Et_2O, 60:40).

^{1}H NMR ($CDCl_3$, 400 MHz) δ = 7.53 (1H, dd, J = 7.8, 1.2 Hz, Ar*H*), 7.31 (1H, t, J = 7.9 Hz, Ar*H*), 7.07 (1H, dd, J = 8.1, 1.2 Hz, Ar*H*), 6.05 (1H, s, C=C*H*), 5.72 (1H, d, J = 9.7 Hz,

CH_AH_B), 4.21 (1H, d, J = 9.7 Hz, CH_AH_B), 4.09 – 3.96 (1H, m, CH_AH_BC=CH), 3.71 (3H, s, CO_2CH_3), 3.63 (3H, s, CO_2CH_3), 2.68 – 2.59 (1H, m, CH_AH_BC=CH), 2.53 – 2.39 (2H, m, CH_2C=CH), 2.08 – 1.88 (2H, m, CH_2CH_2C(O)).

^{13}C NMR ($CDCl_3$, 110 MHz) δ = 201.3 (*C*(O)CH_2), 170.8 (CO_2CH_3), 166.2 (CO_2CH_3), 161.6 (C_{Ar}), 158.7 (*C*=CH), 136.9 (C_{Ar}), 130.4 ($C_{Ar}H$), 125.3 (C_{Ar}), 123.1 ($C_{Ar}H$), 120.1 (C=*C*H), 115.5 ($C_{Ar}H$), 79.4 (CH_AH_B), 63.3 ($C_{Ar}C$), 52.9 (CO_2CH_3), 51.7 (CO_2CH_3), 39.2 (CH_AH_BC=CH), 33.7 (CH_2C=CH), 25.5 (CH_2CH_2C(O)).

IR (film) ν_{max}/cm^{-1} 2952, 1733, 1716, 1673, 145, 1232, 1155.

HRMS: (ESI$^+$) Found $[M + Na]^+$ = 353.0996, $C_{18}H_{18}O_6Na$ requires 353.0996.

2,3-Dihydrobenzofuran-3-carboxylic acid (48)

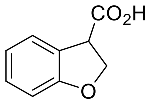

48

To a solution of ester **17** (535 mg, 3.00 mmol) in EtOH (20 mL) at rt was added a solution of Na_2CO_3 (1.00 g, 9.43 mmol) in water (10 mL). The reaction mixture was stirred for 1.5 h. HCl (5 M aq., 5 mL) was added and excess EtOH removed *in vacuo*. Et_2O (50 mL) and water (20 mL) were added and the layers separated. To the organic layer was added sat. aq. Na_2CO_3 (15 mL). the layers were separated and the aqueous layer was acidified with HCl (5 M aq.) until pH = 3. Et_2O (30 mL) was added and the layers separated. The aqueous layer was extracted with Et_2O (3 × 30 mL) and the combined organic phase was washed with sat. aq. NaCl (30 mL), dried over $MgSO_4$ and concentrated *in vacuo* to afford the title compound (266 mg, 54%) as a white solid with no further purification necessary.

$\boldsymbol{R_f}$ = 0.2 ($CHCl_3$:MeOH, 95:5).

m.p. 94 °C

^{1}H NMR ($CDCl_3$, 400 MHz) δ = 9.93 (1H, br. s, CO_2H), 7.41 (1H, d, J = 7.5 Hz, Ar*H*), 7.24 – 7.17 (1H, m, Ar*H*), 6.91 (1H, td, J = 7.5, 1.0 Hz, Ar*H*), 6.84 (1H, d, J = 7.9 Hz, Ar*H*), 4.92 (1H, dd, J = 9.3, 6.3 Hz, CH_AH_B), 4.67 (1H, t, J = 9.5 Hz, CH_AH_B), 4.37 (1H, dd, J = 9.7, 6.3 Hz, C*H*).

^{13}C NMR ($CDCl_3$, 126 MHz) δ = 177.4 (CO_2H), 159.9 (C_{Ar}), 129.9 ($C_{Ar}H$), 125.6 ($C_{Ar}H$), 123.6 (C_{Ar}), 121.0 ($C_{Ar}H$), 110.2 ($C_{Ar}H$), 72.3 (CH_AH_B), 47.1 (*C*H).

Analytical data are in accordance with those previously reported for this compound.[4]

1-(Cyanomethyl)tetrahydro-1H-thiophen-1-ium Bromide (49)[12]

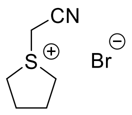

49

To a solution of tetrahydrothiophene (4.80 mL, 54.0 mmol) was added dropwise bromoacetonitrile (3.40 mL, 49.0 mmol). The reaction mixture was stirred at rt for 16 h and the resulting solid was quickly broken into smaller pieces using a spatula. The powder was triturated with Et_2O (3 × 5 mL), then collected and dried under high vacuum to afford the title compound as a white solid (8.52 g, 84%). N.B. This compound was found to be highly hydroscopic and it was necessary to prepare fresh when required. Alternatively, storage under high vacuum was found to extend the useful lifetime of this compound.

m.p. 70 °C.

^{1}H NMR (MeOD, 400 MHz) δ = 4.75 (2H, s, CH_2CN), 3.94 – 3.71 (4H, m, CH_2S), 2.57 – 2.35 (4H, m, CH_2CH$_2$S).

^{13}C NMR (MeOD, 101 MHz) δ = 118.8 (*C*N), 112.8 (*C*H$_2$CN), 46.6 (*C*H$_2$S), 30.1 (*C*H$_2$CH$_2$S).

Analytical data are in accordance with those previously reported for this compound.[12]

3-(2,3-Dihydrobenzofuran-3-yl)-3-oxo-2-(tetrahydro-1λ^4-thiophen-1-ylidene)propanenitrile (50)[12]

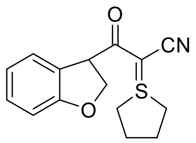

50

To a solution of acid **48** (1.77 g, 10.8 mmol) in CH_2Cl_2 (50 mL) was added *N*-(3-dimethylaminopropyl)-*N*'-ethylcarbodiimide hydrochloride (3.10 g, 16.2 mmol), 4-(dimethylamino)pyridine (132 mg, 1.08 mmol) and salt **49** (2.25 g, 10.8 mmol). The reaction mixture was stirred at rt for 16 h, diluted with CH_2Cl_2 (50 mL) and water (50 mL) and the layers separated. The aqueous layer was extracted with CH_2Cl_2 (3 × 30 mL) and the combined organic phase was washed with sat. aq. NaCl (30 mL), dried over $MgSO_4$ and concentrated *in vacuo*. Purification by flash column chromatography (SiO_2, $CHCl_3$:MeOH, 60:40) afforded the title compound as a beige solid (2.30 g, 78%).

***R*f** = 0.3 ($CHCl_3$:MeOH, 95:5).

m.p. 153 °C.

^{1}H NMR ($CDCl_3$, 400 MHz) δ = 7.36 (1H, dt, J = 7.8, 1.3 Hz, Ar*H*), 7.20 – 7.06 (1H, m, Ar*H*), 6.83 (1H, td, J = 7.5, 1.0 Hz, Ar*H*), 6.79 (1H, d, J = 8.1 Hz, Ar*H*), 4.89 (1H, dd, J = 8.5, 5.7 Hz, C$H_A$$H_B$), 4.74 (1H, dd, J = 9.3, 5.6 Hz, C*H*), 4.62 (1H, dd, J = 9.4, 8.5 Hz, C$H_A$$H_B$), 3.44 – 3.25 (4H, m, S(CH_2CH_2)$_A$(CH_2CH_2)$_B$ and S(CH_2CH_2)$_A$(CH_2CH_2)$_B$), 2.70 – 2.51 (2H, m, S(CH_2CH_2)$_A$(CH_2CH_2)$_B$), 2.09 – 1.95 (2H, m, S(CH_2CH_2)$_A$(CH_2CH_2)$_B$).

^{13}C NMR ($CDCl_3$, 110 MHz) δ = 189.1 (*C*(O)), 160.2 (C_{Ar}), 129.0 (C_{Ar}H), 127.0 (C_{Ar}), 124.9 (C_{Ar}H), 120.8 (*C*N), 120.5 (C_{Ar}H), 109.9 (C_{Ar}H), 73.2 (*C*$H_A$$H_B$), 53.4 (*C*=S), 50.2 (*C*H), 45.0 (S(*C*H_2CH_2)$_A$(CH_2CH_2)$_B$), 44.9 (S(CH_2CH_2)$_A$(*C*H_2CH_2)$_B$), 28.7 (S(CH_2*C*H_2)$_A$(CH_2CH_2)$_B$), 28.6 (S(CH_2CH_2)$_A$(CH_2*C*H_2)$_B$).

IR (film) ν_{max}/cm^{-1} 2957, 2160, 1977, 1588, 1482, 1236, 1155, 961, 766.

HRMS: (ESI$^+$) Found [M + Na]$^+$ = 296.0715, $C_{15}H_{15}O_2NNaS$ requires 296.0716.

Methyl 3-(3-(2-cyano-2-(tetrahydro-1λ^4-thiophen-1-ylidene)acetyl)-2,3-dihydrobenzofuran-3-yl)propiolate (51)

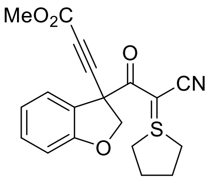

51

To a stirred solution of cyanoketothiane **50** (50 mg, 180 μmol) in THF (1 mL) at –78 °C was added LDA (220 μL, 220 μmol, 1 M in THF). After 10 min chloroacetylene **20** (32 mg, 270 μmol) in THF (0.6 mL) was added dropwise and the reaction mixture was stirred at –78 °C for 30 min. Sat. aq. NH_4Cl (5 mL) and water (20 mL) were added and the mixture extracted with EtOAc (3 × 30 mL). The combined organic phase was washed with sat. aq. NaCl (20 mL), dried over $MgSO_4$ and concentrated *in vacuo*. Purification by flash column chromatography (SiO_2, $CHCl_3$:MeOH, 99:1) afforded the title compound as a colourless oil (38 mg, 60%).

***R*f** = 0.2 ($CHCl_3$:MeOH, 98.5:1.5).

^{1}H NMR ($CDCl_3$, 400 MHz) δ = 7.69 (1H, dd, J = 7.6, 1.3 Hz, Ar*H*), 7.24 – 7.18 (1H, m, Ar*H*), 6.95 (1H, td, J = 7.5, 1.0 Hz, Ar*H*), 6.82 (1H, d, J = 8.1 Hz, Ar*H*), 5.25 (1H, d, J = 8.9 Hz, C$H_A$$H_B$), 4.72 (1H, d, J = 8.9 Hz, C$H_A$$H_B$), 3.77 (3H, s, CO_2CH_3), 3.43 – 3.26 (4H, m,

S(CH_2CH$_2$)$_A$(CH$_2$CH$_2$)$_B$ and S(CH$_2$CH$_2$)$_A$(CH_2CH$_2$)$_B$), 2.65 – 2.47 (2H, m, S(CH$_2$CH_2)$_A$(CH$_2$CH$_2$)$_B$), 2.11 – 2.02 (2H, m, S(CH$_2$CH$_2$)$_A$(CH$_2$CH_2)$_B$).

^{13}C NMR (CDCl$_3$, 110 MHz) δ = 183.3 (*C*(O)), 159.6 (*C*$_{Ar}$), 154.0 (*C*O$_2$CH$_3$), 130.3 (*C*$_{Ar}$H), 126.8 (*C*$_{Ar}$), 126.1 (*C*$_{Ar}$H), 121.5 (*C*$_{Ar}$H), 119.1 (*C*N), 110.6 (*C*$_{Ar}$H), 85.8 (*C*≡CCO$_2$CH$_3$), 79.6 (*C*H$_A$H$_B$), 77.5 (C≡*C*CO$_2$CH$_3$), 54.5 (C$_{Ar}$*C*), 54.0 (*C*=S), 53.0 (CO$_2$*C*H$_3$), 45.0 (S(*C*H$_2$CH$_2$)$_A$(CH$_2$CH$_2$)$_B$), 44.8 (S(CH$_2$CH$_2$)$_A$(*C*H$_2$CH$_2$)$_B$), 28.6 (S(CH$_2$*C*H$_2$)$_A$(CH$_2$CH$_2$)$_B$), 28.6 (S(CH$_2$CH$_2$)$_A$(CH$_2$*C*H$_2$)$_B$).

IR (film) ν_{max}/cm^{-1} 2955, 2235, 2170, 1714, 1596, 1480, 1274, 1236, 1154, 752.

HRMS: (ESI$^+$) Found [M + H]$^+$ = 356.0953, $C_{19}H_{18}O_4NS$ requires 356.0951.

Methyl (*Z*)-3-(3-(2-cyano-2-(tetrahydro-1λ^4-thiophen-1-ylidene)acetyl)-2,3-dihydrobenzofuran-3-yl)-5-methylhex-2-enoate (52)[6]

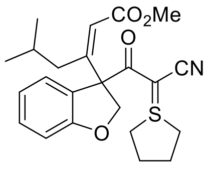

52

To a solution of CuI (32 mg, 170 µmol) in THF (0.7 mL) at –78 °C was added ^{i}BuLi (200 µL, 340 µmol, 1.7 M in hexane). The mixture was stirred vigorously at 0 °C for 1 h before being cooled to –78 °C. A solution of alkyne **51** (15 mg, 42 µmol) in THF (0.5 mL + 0.25 mL) was added dropwise and the reaction mixture stirred at –78 °C for 5 min. MeOH (1 mL) was added followed by water (20 mL) and the mixture extracted with EtOAc (3 × 20 mL). The combined organic phase was washed with sat. aq. NaCl (20 mL), dried over MgSO$_4$ and concentrated *in vacuo*. Purification by flash column chromatography (SiO$_2$, EtOAc) afforded the title compound as an orange oil and a single diastereomer (14 mg, 79%).

***R*f** = 0.2 (EtOAc).

^{1}H NMR (CDCl$_3$, 400 MHz) δ = 7.54 (1H, dd, *J* = 7.8, 1.4 Hz, Ar*H*), 7.19 – 7.12 (1H, m, Ar*H*), 6.84 (1H, td, *J* = 7.5, 1.1 Hz, Ar*H*), 6.78 (1H, d, *J* = 8.1 Hz, Ar*H*), 5.90 (1H, s, C=C*H*), 5.83 (1H, d, *J* = 9.4 Hz, CH_AH$_B$), 4.29 (1H, d, *J* = 9.5 Hz, CH$_A$$H_B$), 3.64 (3H, s, CO$_2CH_3$), 3.49 – 3.18 (4H, m, S(CH_2CH$_2$)$_A$(CH$_2$CH$_2$)$_B$ and S(CH$_2$CH$_2$)$_A$(CH_2CH$_2$)$_B$), 2.66 – 2.53 (3H, m, S(CH$_2$CH_2)$_A$(CH$_2$CH$_2$)$_B$ and C=CCH_AH$_B$), 2.05 – 1.91 (3H, m, S(CH$_2$CH$_2$)$_A$(CH$_2$CH_2)$_B$ and C=CCH$_A$$H_B$), 1.24 – 1.19 (1H, m, C*H*), 0.79 (3H, d, *J* = 6.5 Hz, CH(CH_3)$_A$(CH$_3$)$_B$), 0.56 (3H, d, *J* = 6.6 Hz, CH(CH$_3$)$_A$(CH_3)$_B$).

^{13}**C NMR** ($CDCl_3$, 126 MHz) δ = 187.7 (C(O)), 166.4 (CO_2CH_3), 162.3 (C_{Ar}), 160.8 (C=CH), 129.7 (C_{Ar}H), 127.6 (C_{Ar}), 126.4 (C_{Ar}H), 121.2 (CN), 120.2 (C_{Ar}H), 119.6 (C=CH), 110.3 (C_{Ar}H), 81.2 (CH_AH_B), 64.9 ($C_{Ar}C$), 51.3 (CO_2CH_3), 50.1 (C=S), 48.3 (CH_AH_BC=CH), 43.4 ($S(CH_2CH_2)_A(CH_2CH_2)_B$), 43.3 ($S(CH_2CH_2)_A(CH_2CH_2)_B$), 28.6 ($S(CH_2CH_2)_A(CH_2CH_2)_B$), 28.6 ($S(CH_2CH_2)_A(CH_2CH_2)_B$), 26.2 ($CH(CH_3)_A(CH_3)_B$), 23.3 ($CH(CH_3)_A(CH_3)_B$), 21.3 ($CH(CH_3)_A(CH_3)_B$).

IR (film) ν_{max}/cm^{-1} 2956, 2239, 2164, 1715, 1593, 1480, 1238, 1205, 1156, 752, 731.

HRMS: (ESI$^+$) Found [M + Na]$^+$ = 436.1544, $C_{23}H_{27}O_4NNaS$ requires 436.1553.

Methyl (*Z*)-3-(3-(2-methoxy-2-oxoacetyl)-2,3-dihydrobenzofuran-3-yl)-5-methylhex-2-enoate (53)[13]

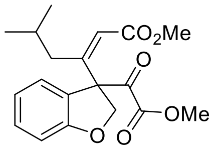

53

Ozone was bubbled through a solution of ylide **52** (7 mg, 17 µmol) in CH_2Cl_2 (1.6 mL) and MeOH (0.4 mL) at –78 °C until an intense blue colour persisted. Dimethylsulfide (500 µL, 6.8 mmol) was added and the solution stirred at rt for 1 h. The mixture was concentrated *in vacuo*. Purification by flash column chromatography (SiO_2, Pentane:EtOAc, 90:10) afforded the title compound as a pale yellow oil and a single diastereomer (2 mg, 34%).

***R*f** = 0.2 (Pentane:EtOAc, 90:10).

^{1}**H NMR** ($CDCl_3$, 500 MHz) δ = 7.41 (1H, d, J = 7.6 Hz, ArH), 7.28 – 7.21 (1H, m, ArH), 6.92 – 6.86 (2H, m, ArH × 2), 5.84 (1H, s, C=CH), 5.64 (1H, d, J = 9.8 Hz, CH_AH$_B$), 4.41 (1H, d, J = 9.9 Hz, CH$_A$$H_B$), 3.86 (3H, s, C(O)$CO_2CH_3$), 3.71 (3H, s, CO_2CH_3), 2.42 (1H, ddd, J = 14.3, 4.5, 1.1 Hz, C=CCH_AH$_B$), 1.97 (1H, ddd, J = 14.4, 9.1, 1.0 Hz, C=CCH$_A$$H_B$), 1.36 – 1.31 (1H, m, C$H$($CH_3$)$_A$($CH_3$)$_B$), 0.82 (3H, d, J = 6.6 Hz, CH(CH_3)$_A$(CH_3)$_B$), 0.65 (3H, d, J = 6.6 Hz, CH(CH_3)$_A$(CH_3)$_B$).

^{13}**C NMR** ($CDCl_3$, 126 MHz) δ = 187.7 (C(O)CO_2CH_3), 167.0 (CO_2CH_3), 163.4 (C=CH), 162.4 (C(O)CO_2CH_3), 161.5 (C_{Ar}), 130.7 (C_{Ar}H), 127.6 (C_{Ar}H), 125.1 (C_{Ar}), 120.6 (C_{Ar}H), 117.5 (C=CH), 110.7 (C_{Ar}H), 81.2 (CH_AH_B), 65.1 ($C_{Ar}C$), 53.1 (C(O)CO_2CH_3), 51.8 (CO_2CH_3), 47.3 (CH_AH_BC=CH), 26.0 ($CH(CH_3)_A(CH_3)_B$), 23.1 ($CH(CH_3)_A(CH_3)_B$), 21.5 ($CH(CH_3)_A(CH_3)_B$).

IR (film) ν_{max}/cm^{-1} 2956, 1733, 1717, 1684, 1480, 1326, 1214, 1166, 1127, 756.

HRMS: (ESI$^+$) Found [M + Na]$^+$ = 369.1309, $C_{19}H_{22}O_6Na$ requires 369.1309.

1.3 NMR Spectra

^{1}H NMR (400 MHz, $CDCl_3$) Ethyl benzofuran-3-carboxylate (16):

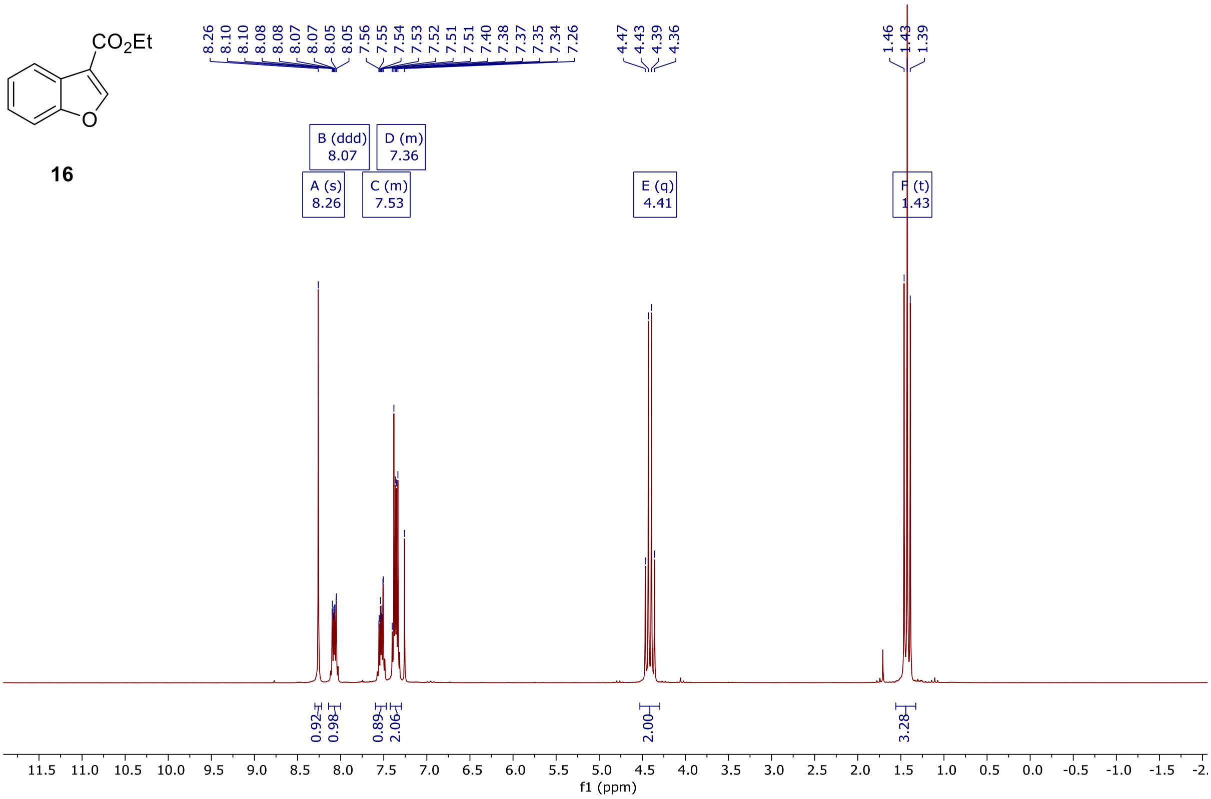

^{13}C NMR (101 MHz, $CDCl_3$):

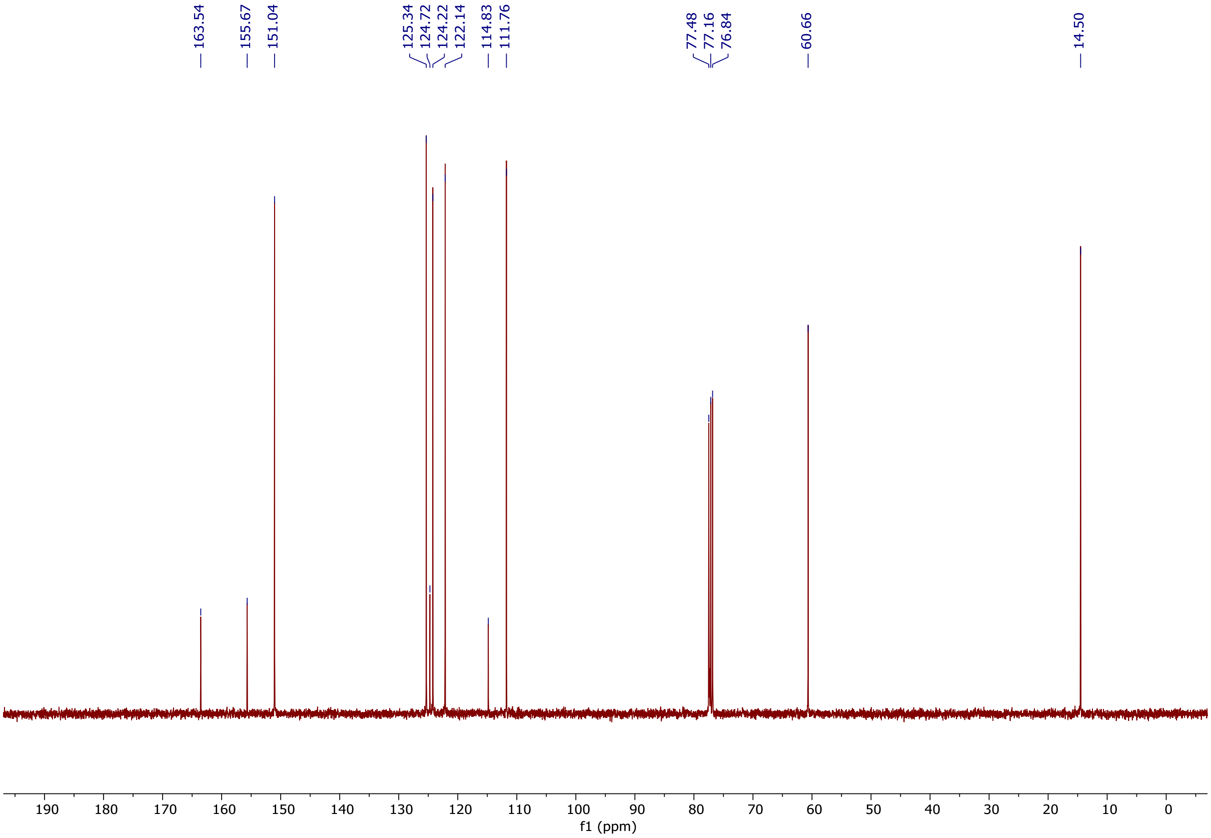

^{1}H NMR (400 MHz, $CDCl_3$) Methyl 2,3-dihydrobenzofuran-3-carboxylate (17):

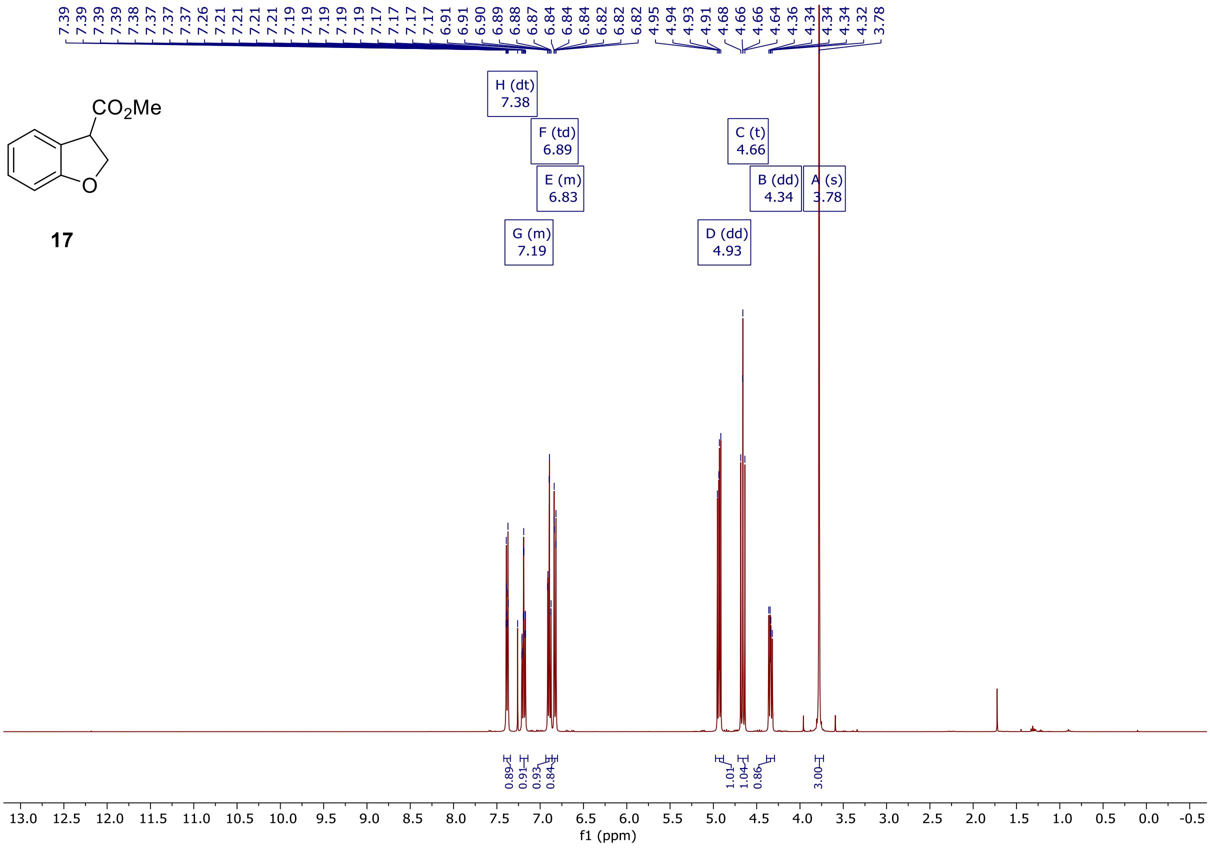

17

^{13}C NMR (101 MHz, $CDCl_3$):

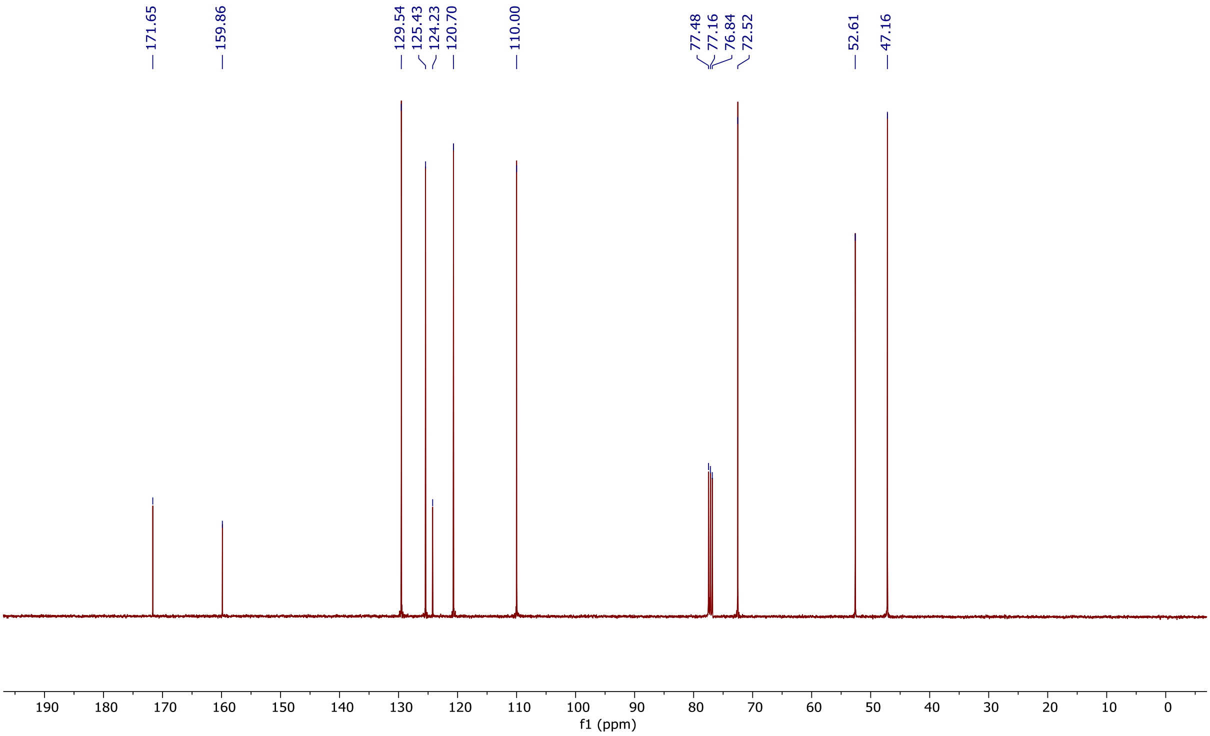

^{1}H NMR (400 MHz, $CDCl_3$) Methyl 3-bromopropiolate (19):

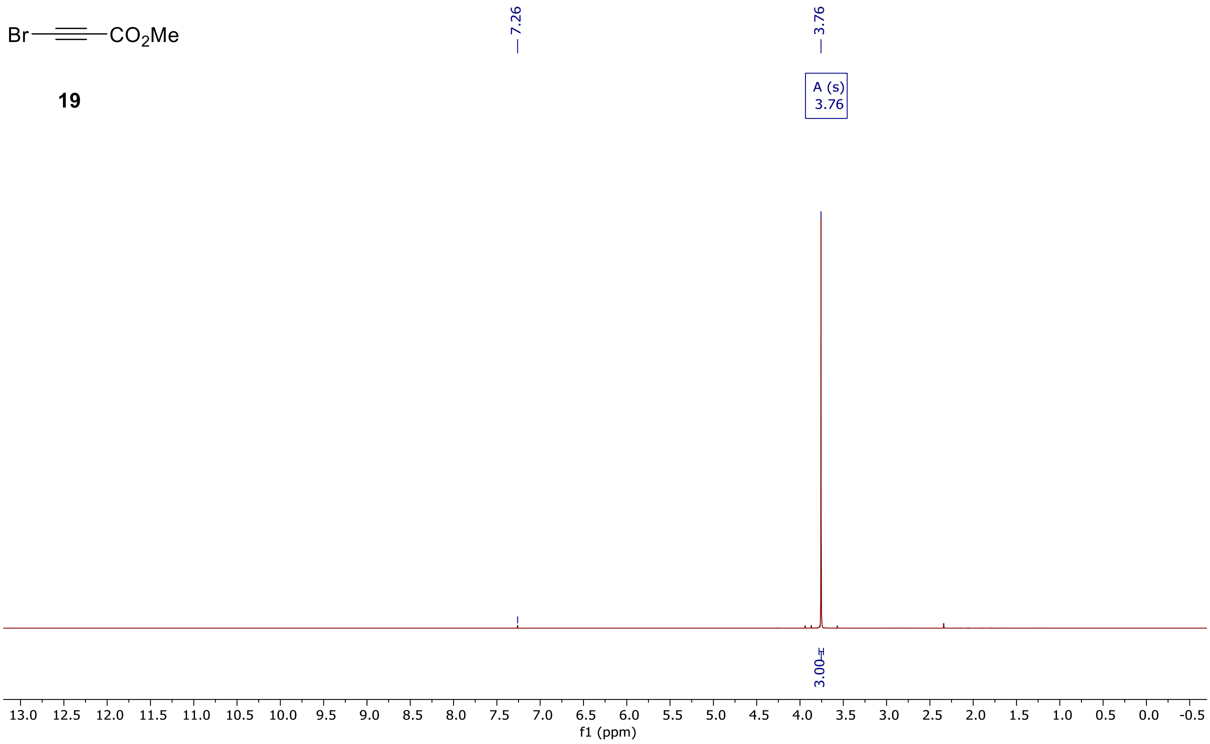

^{13}C NMR (101 MHz, $CDCl_3$):

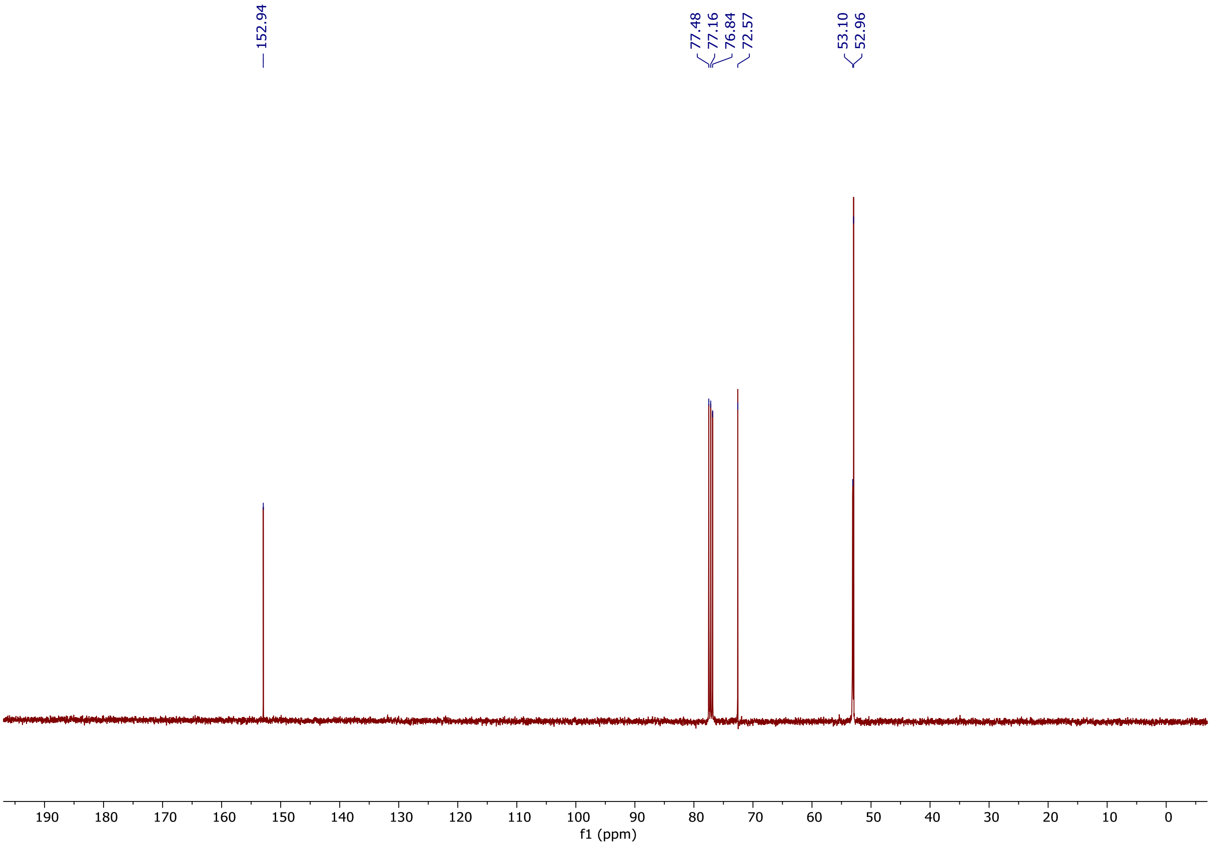

^{1}H NMR (400 MHz, $CDCl_3$) Methyl 3-chloropropiolate (20):

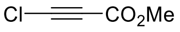

20

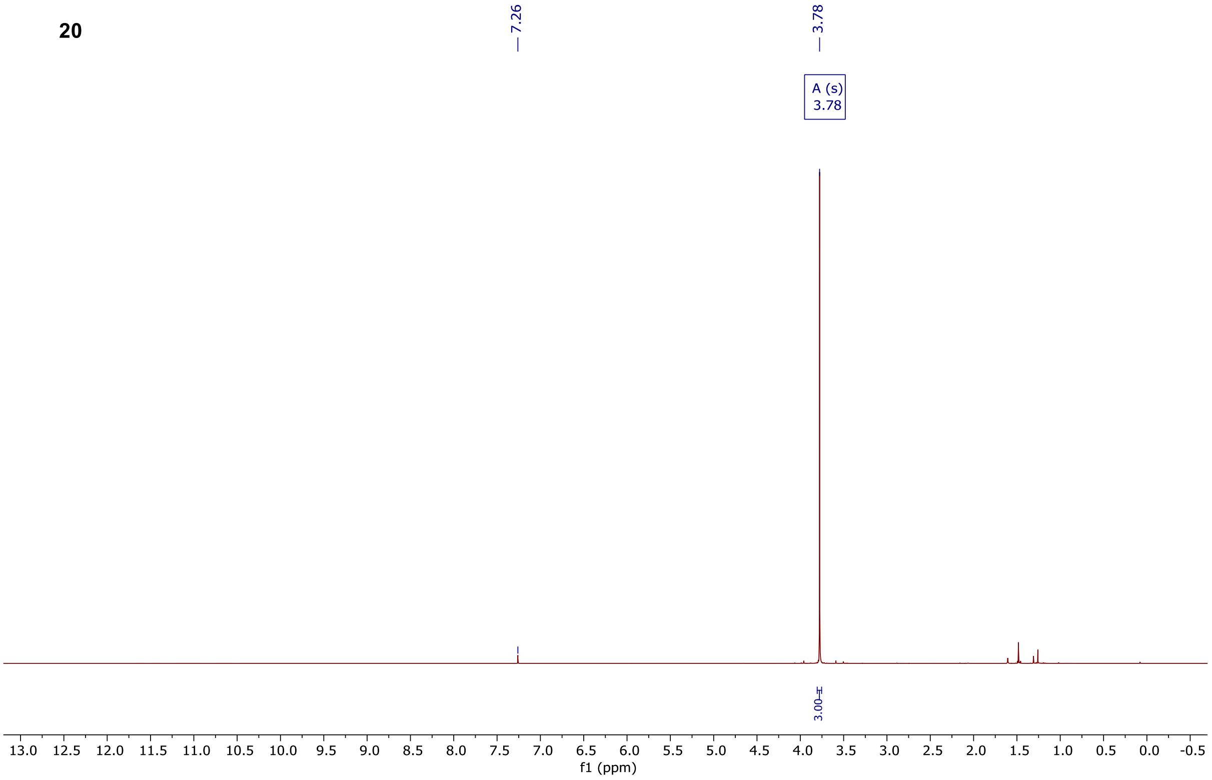

^{13}C NMR (101 MHz, $CDCl_3$):

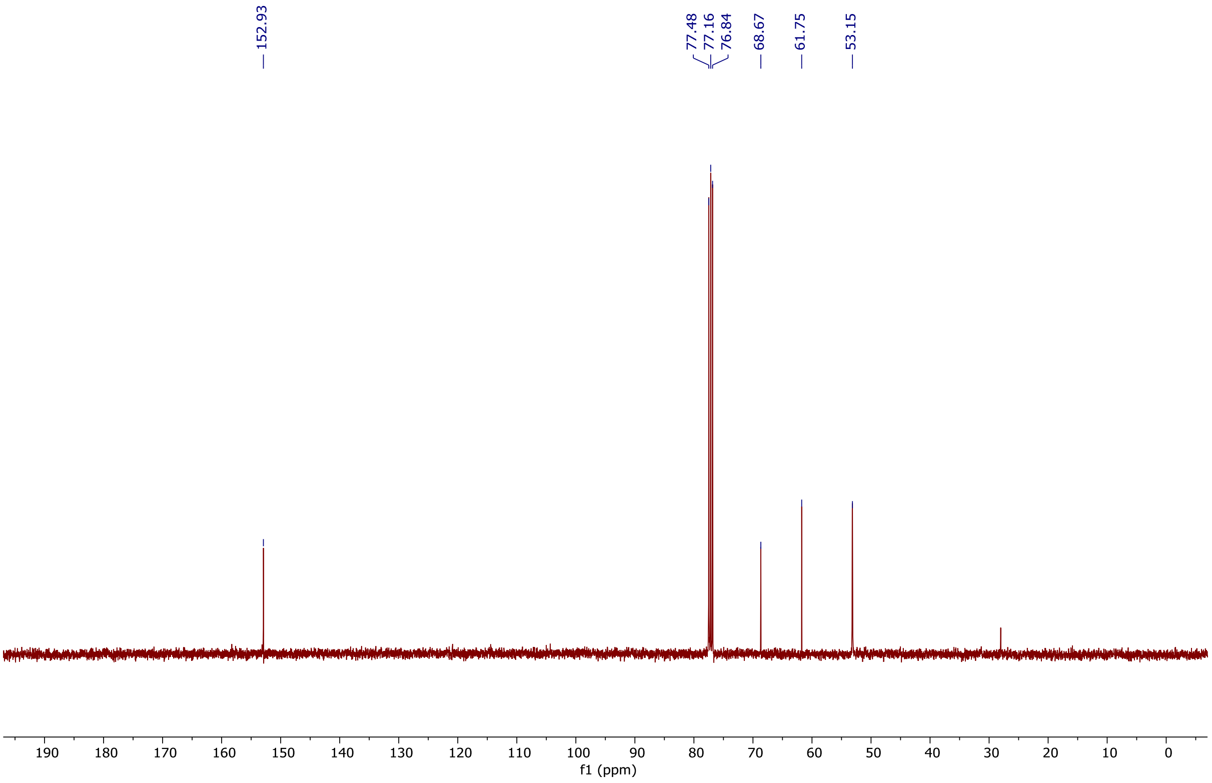

^{1}H NMR (400 MHz, $CDCl_3$) Methyl 3-(3-methoxy-3-oxoprop-1-yn-1-yl)-2,3-dihydrobenzofuran-3-carboxylate (21):

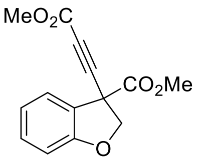

21

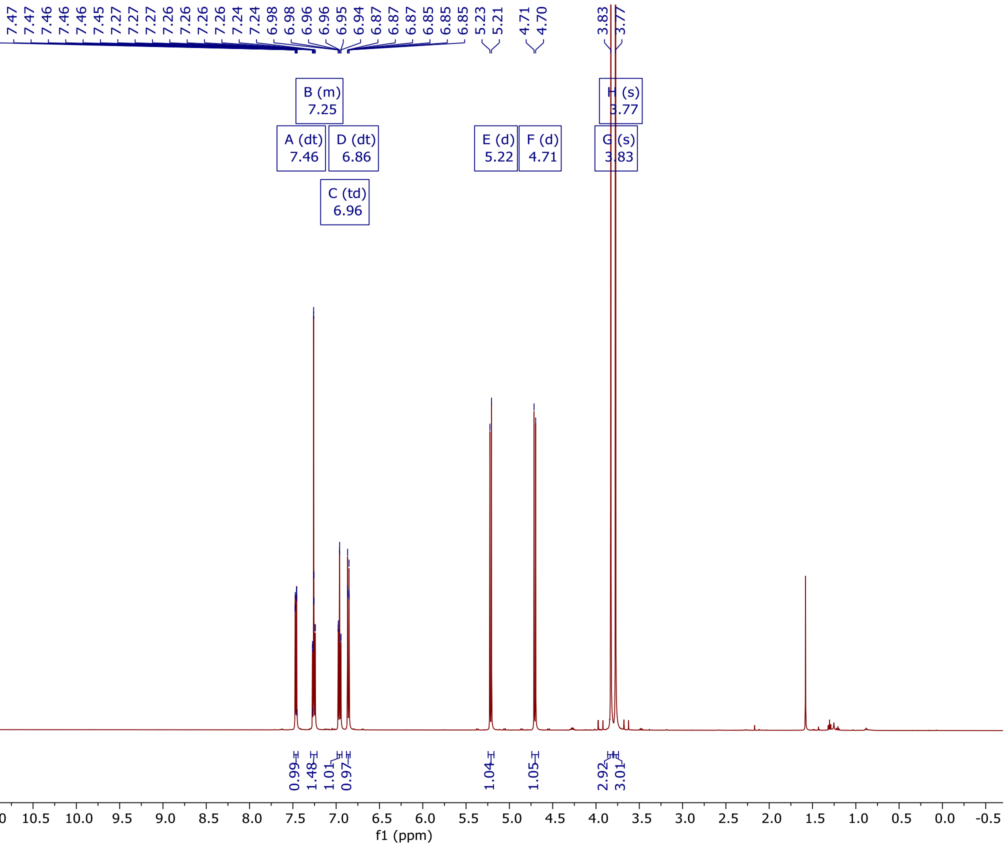

^{13}C NMR (101 MHz, $CDCl_3$):

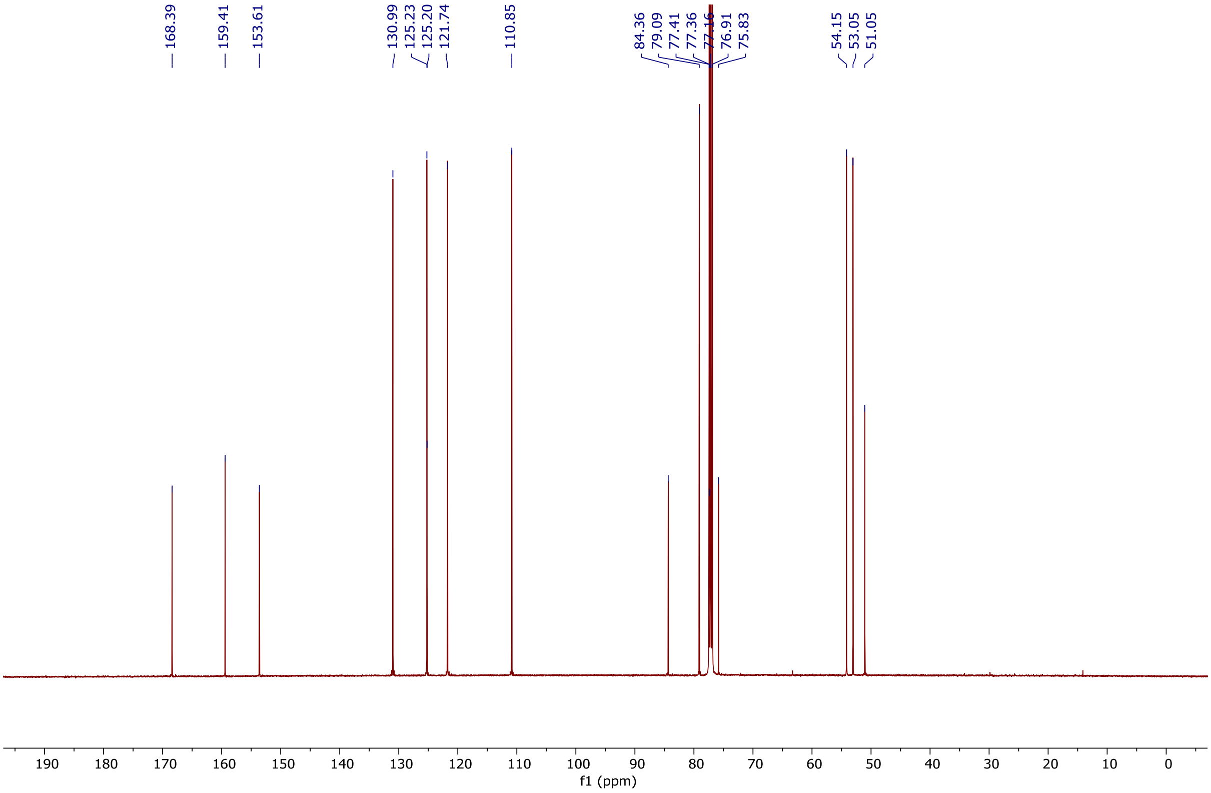

^{1}H NMR (400 MHz, CDCl3) Methyl 6-(3-methoxy-3-oxoprop-1-yn-1-yl)-6,7-dihydro-[1,3]dioxolo[4,5-*g*]benzofuran-6-carboxylate (22):

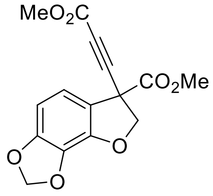

22

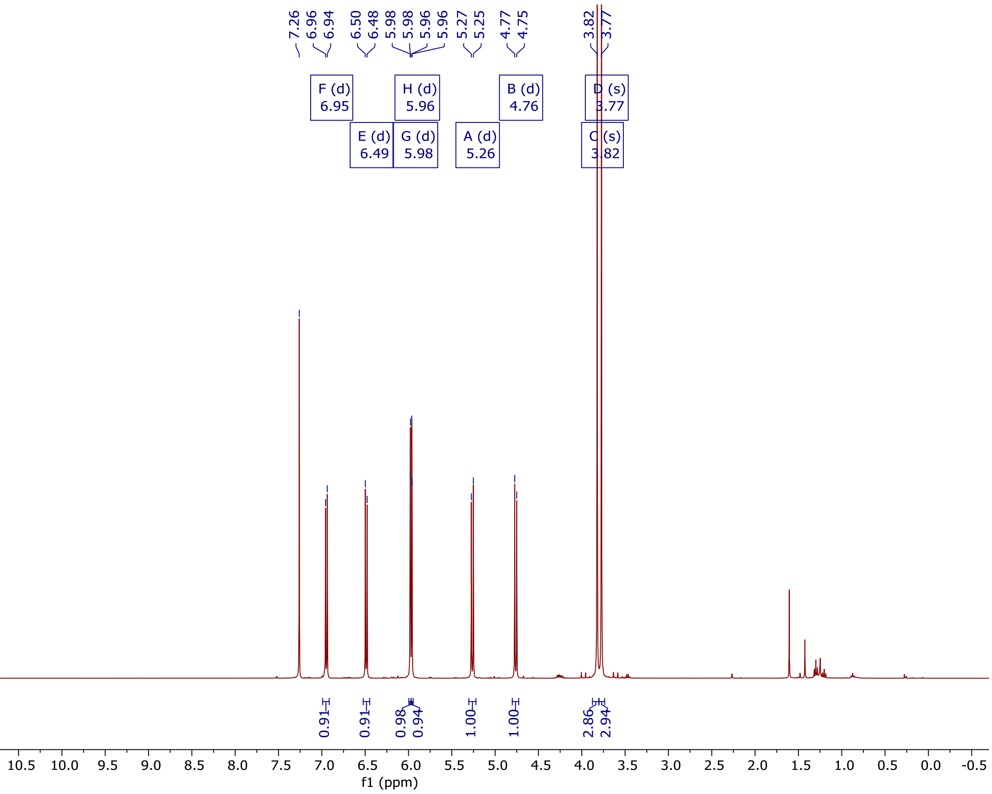

^{13}C NMR (101 MHz, CDCl3):

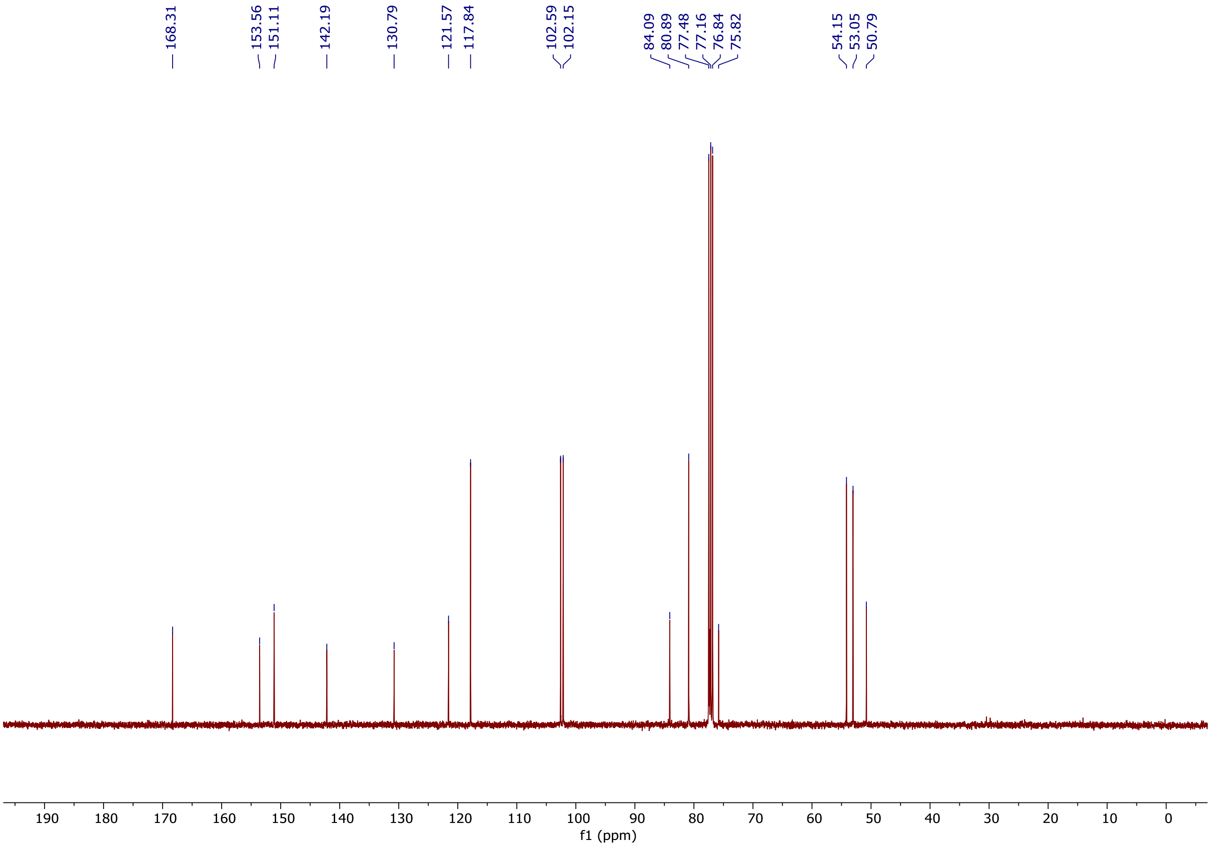

^{1}H NMR (400 MHz, $CDCl_3$) Ethyl 3-(3-methoxy-3-oxoprop-1-yn-1-yl)-4-(trimethylsilyl)-2,3-dihydrobenzofuran-3-carboxylate (23):

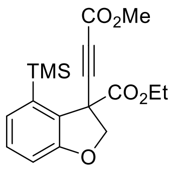

23

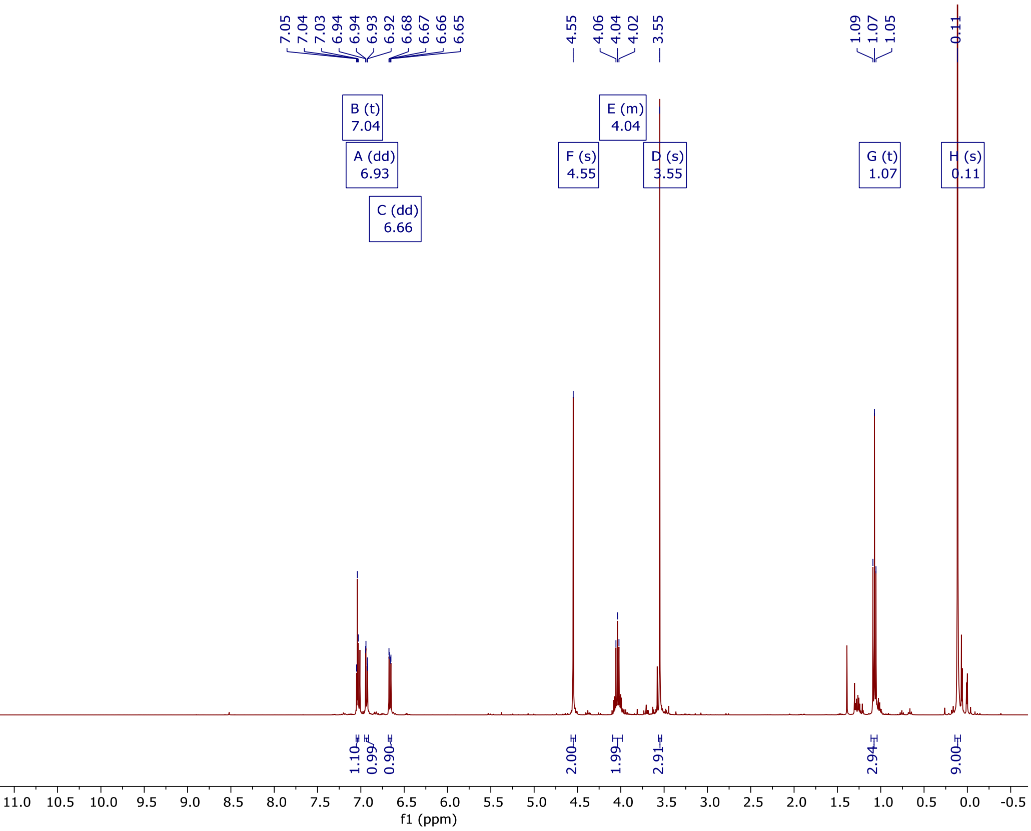

^{13}C NMR (101 MHz, $CDCl_3$):

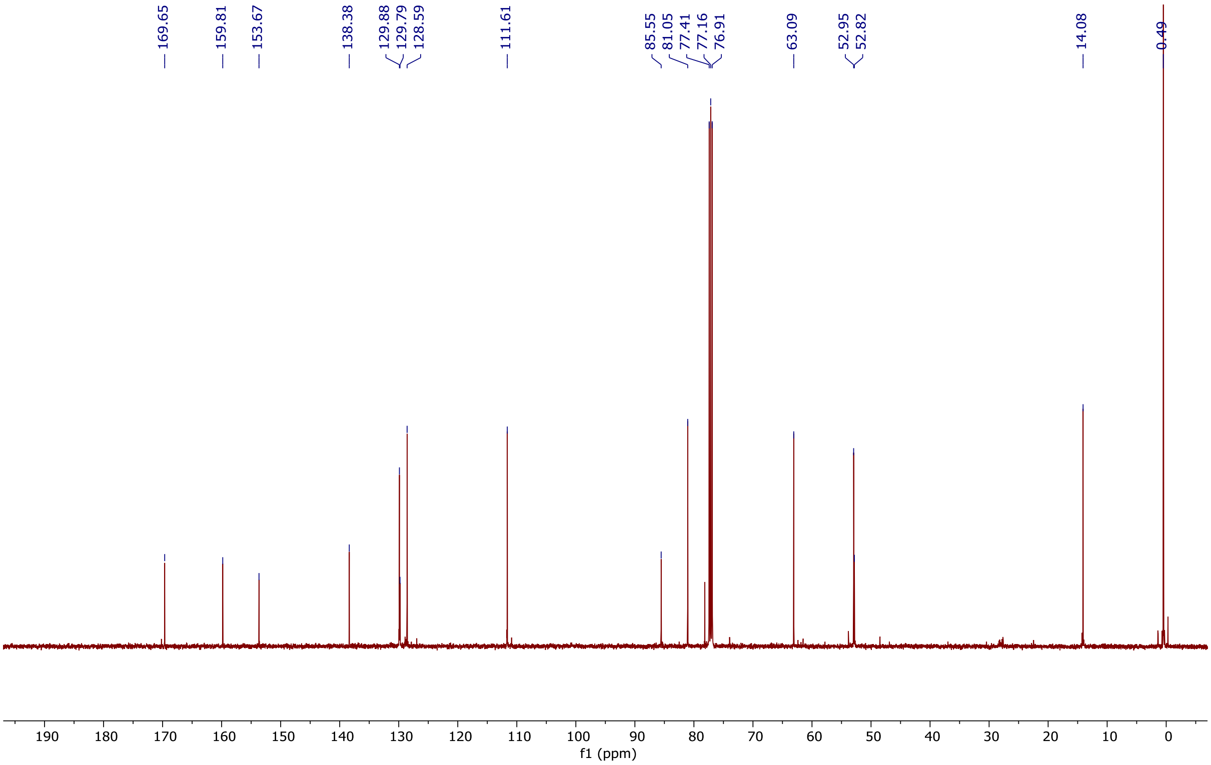

^{1}H NMR (400 MHz, $CDCl_3$) 2-(Trimethylsilyl)ethyl 3-(3-methoxy-3-oxoprop-1-yn-1-yl)-2,3-dihydrobenzofuran-3-carboxylate (24):

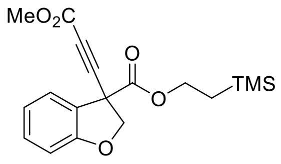

24

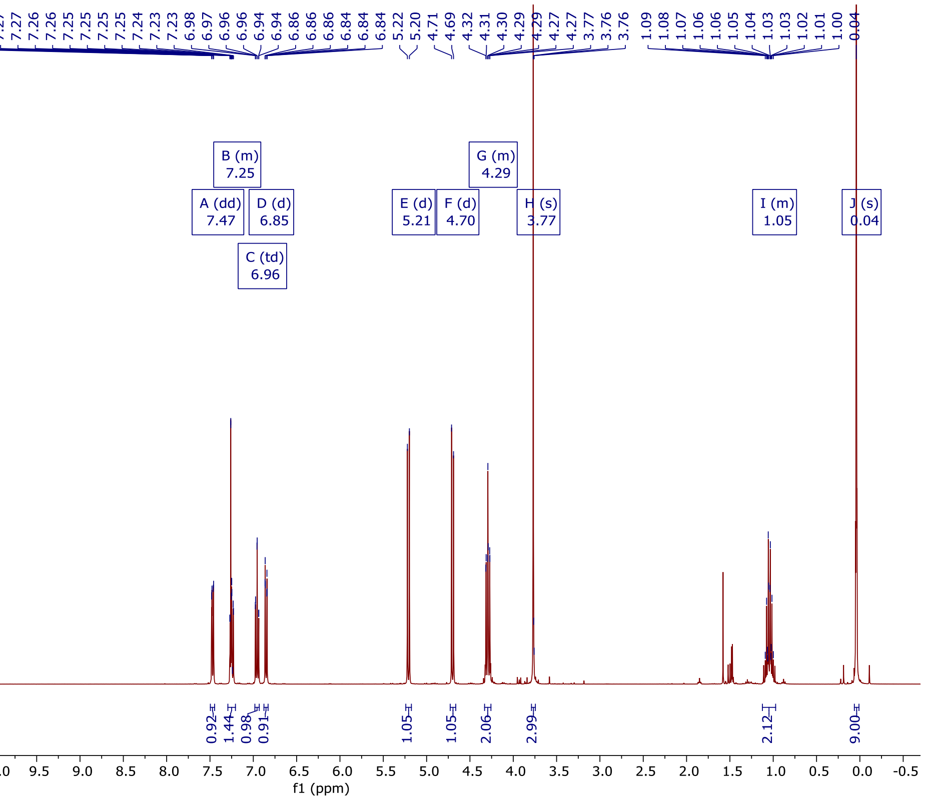

^{13}C NMR (101 MHz, $CDCl_3$):

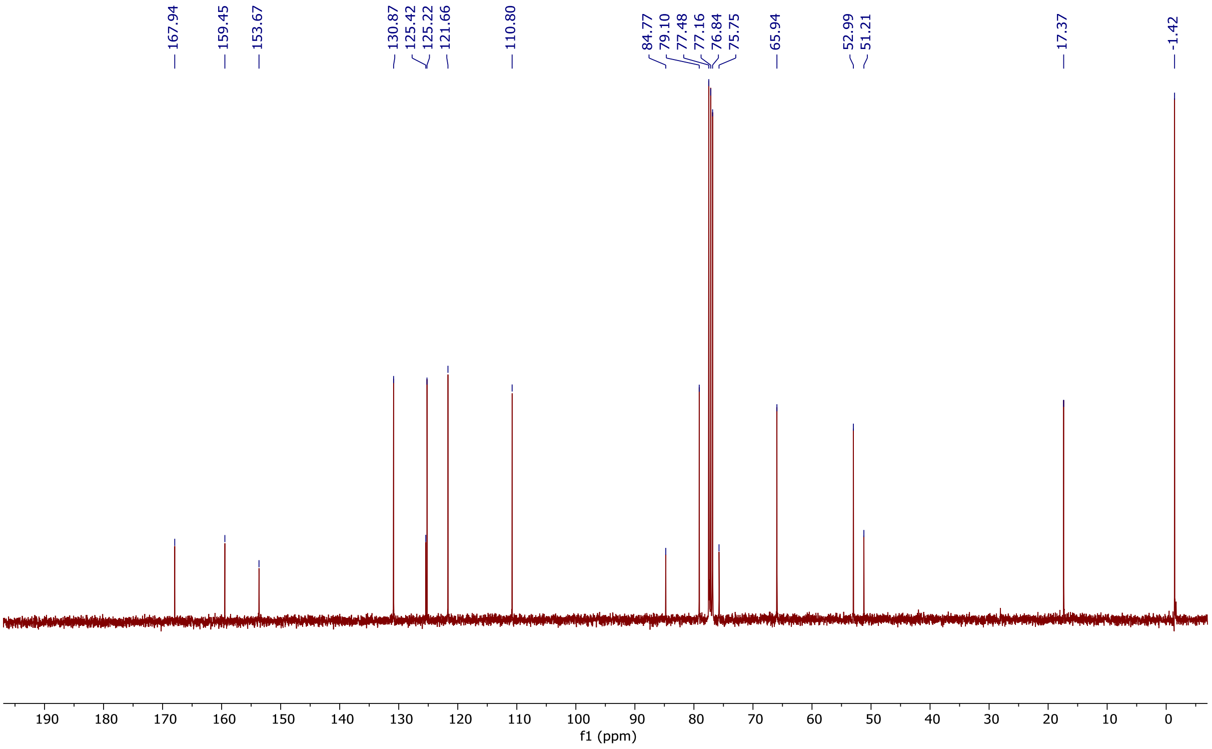

^{1}H NMR (400 MHz, $CDCl_3$) Methyl 3-(3-(tert-butoxy)-3-oxoprop-1-yn-1-yl)-2,3-dihydrobenzofuran-3-carboxylate (25):

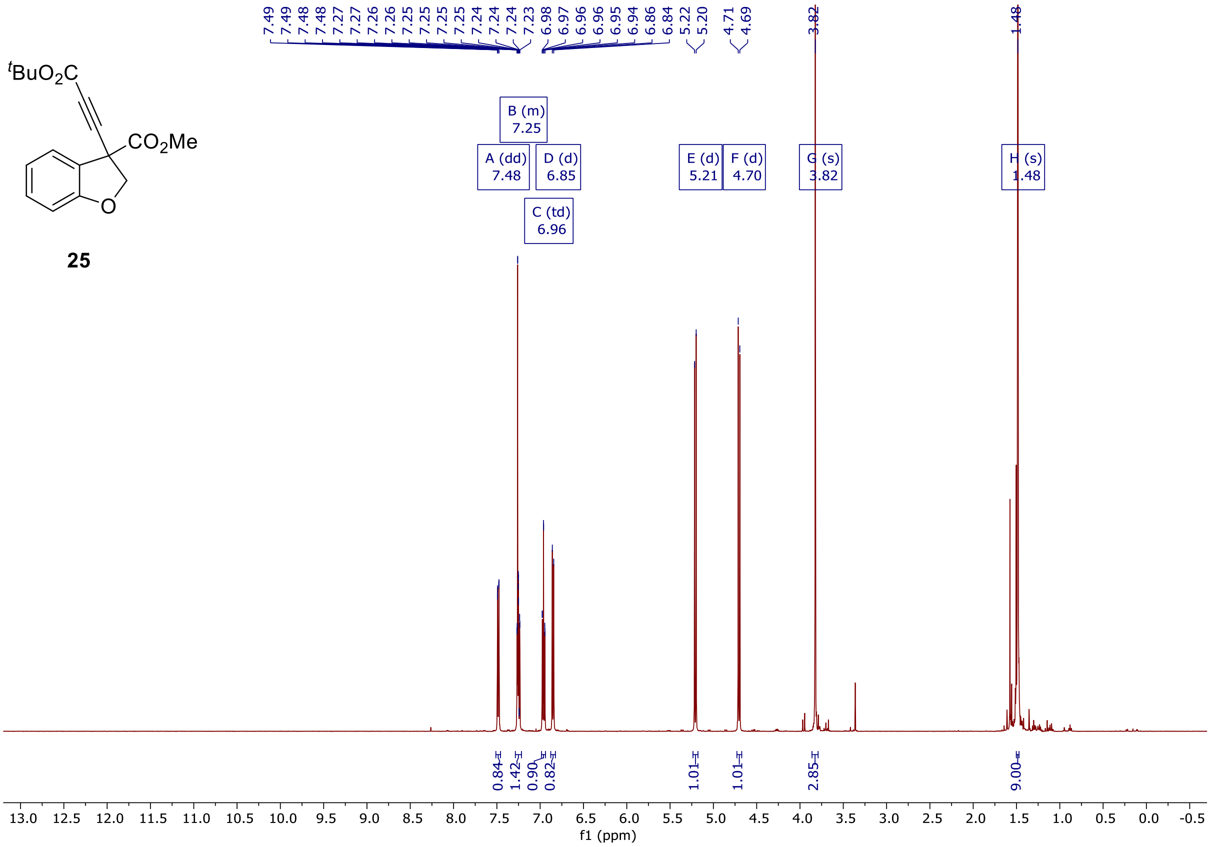

^{13}C NMR (101 MHz, $CDCl_3$):

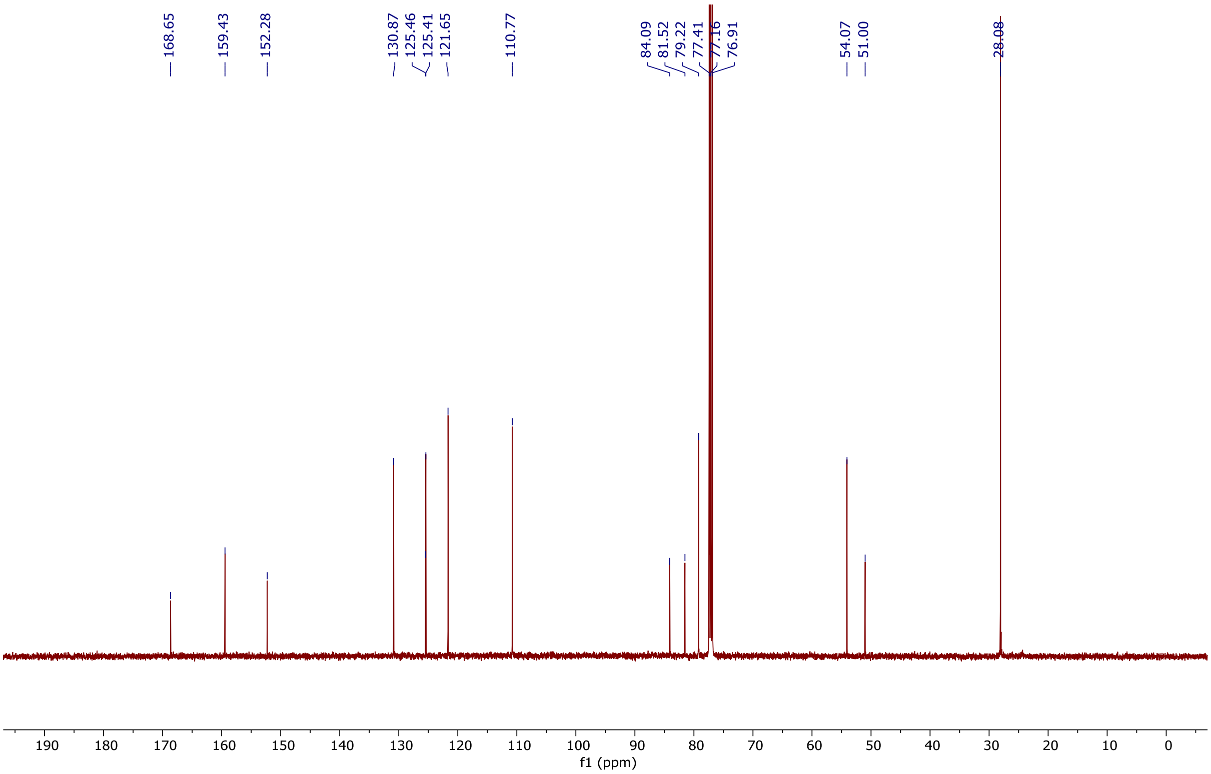

^{1}H NMR (400 MHz, $CDCl_3$) *tert*-Butyl 3-(3-(methoxy(methyl)carbamoyl)-2,3-dihydrobenzofuran-3-yl)propiolate (26):

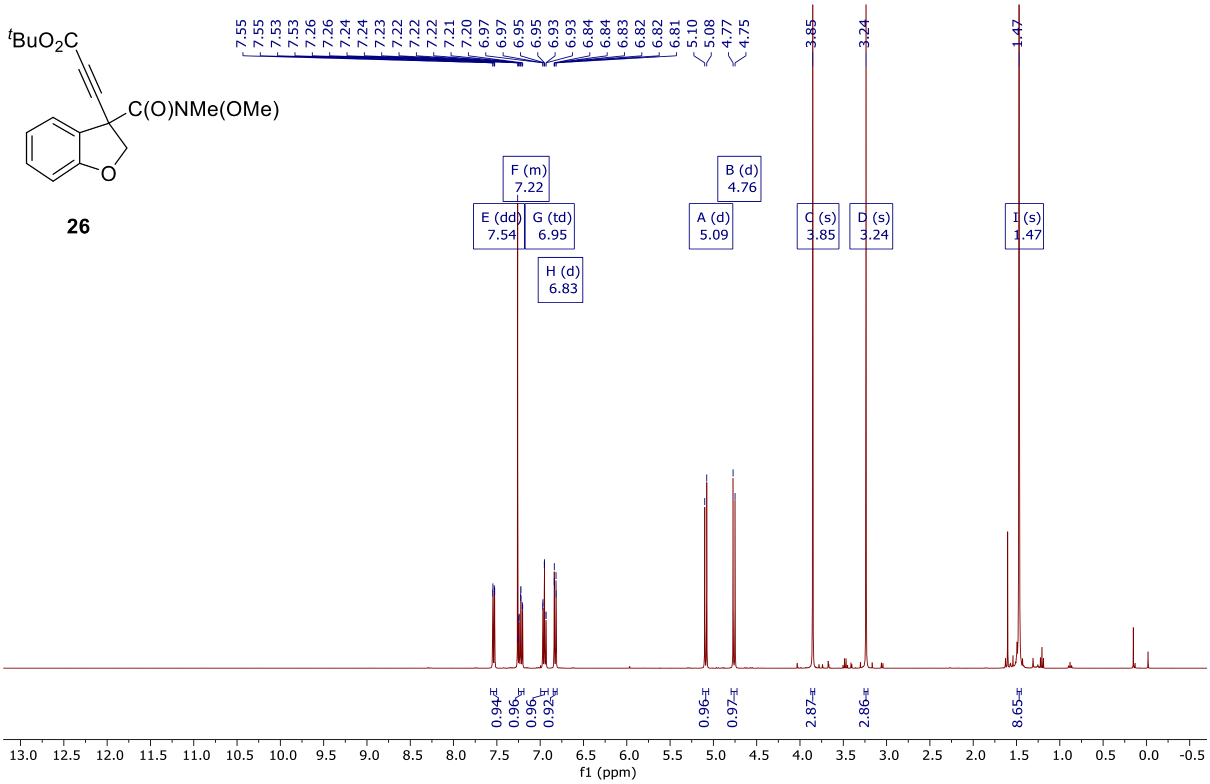

^{13}C NMR (101 MHz, $CDCl_3$):

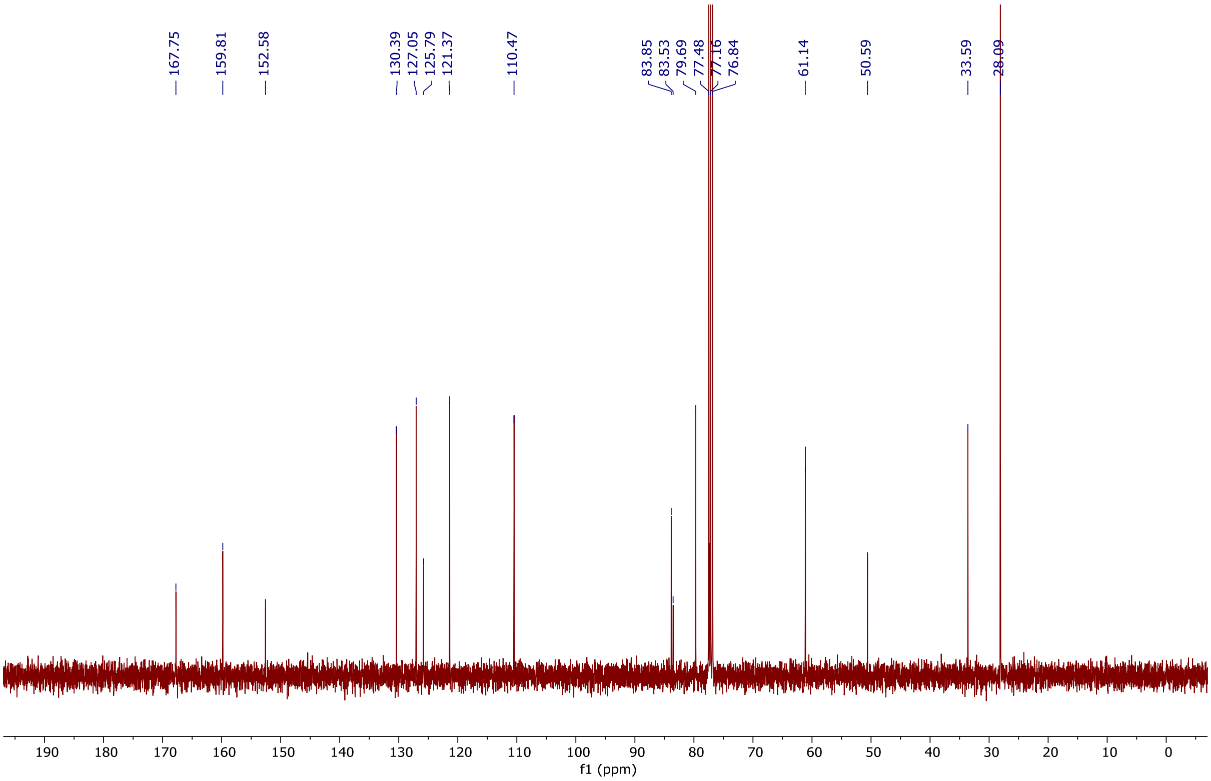

^{1}H NMR (400 MHz, $CDCl_3$) Methyl 3-(3-((ethylthio)carbonyl)-2,3-dihydrobenzofuran-3-yl)propiolate (27):

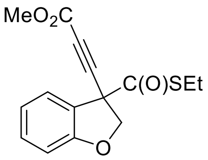

27

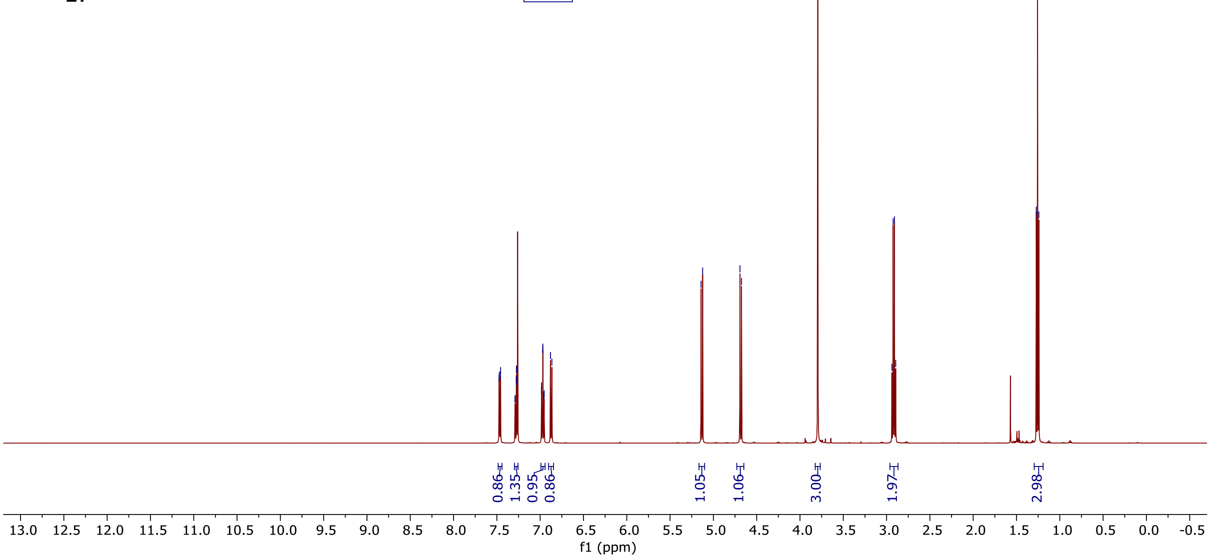

^{13}C NMR (101 MHz, $CDCl_3$):

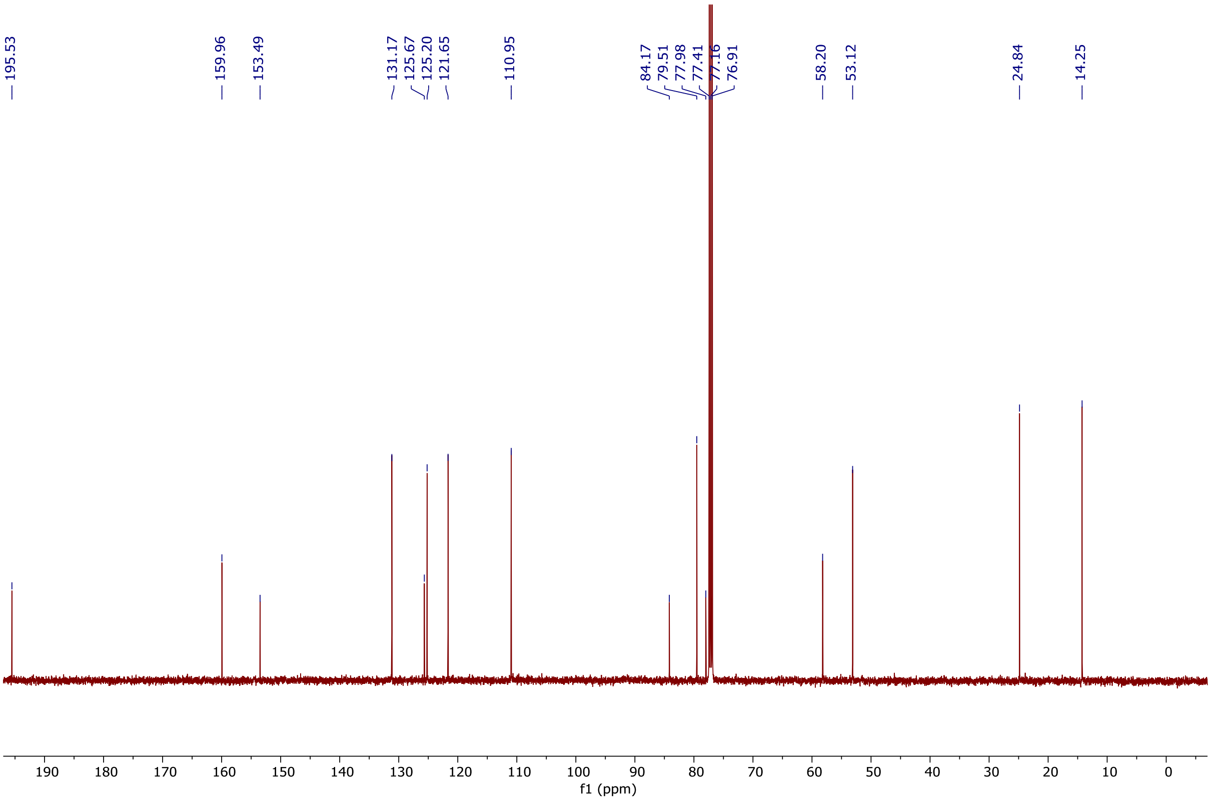

^{1}H NMR (400 MHz, $CDCl_3$) Methyl 3-(3-(4-methyl-2,6,7-trithiabicyclo[2.2.2]octane-1-carbonyl)-2,3-dihydrobenzofuran-3-yl)propiolate (28):

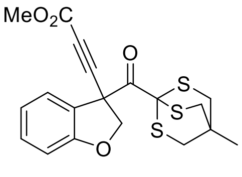

28

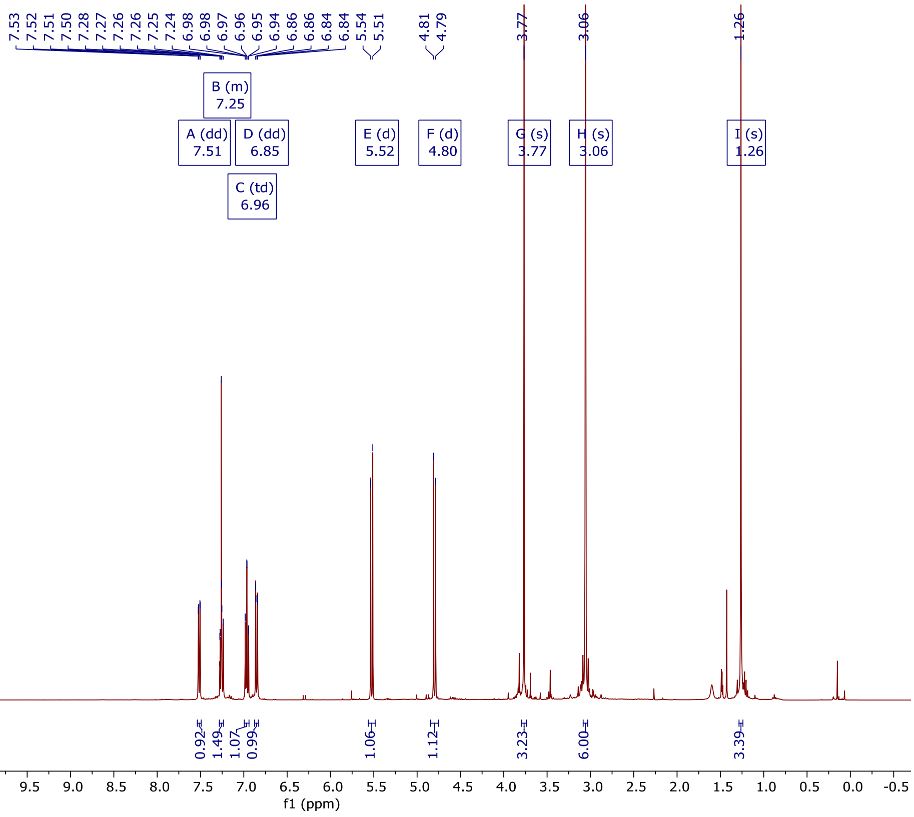

^{13}C NMR (101 MHz, $CDCl_3$):

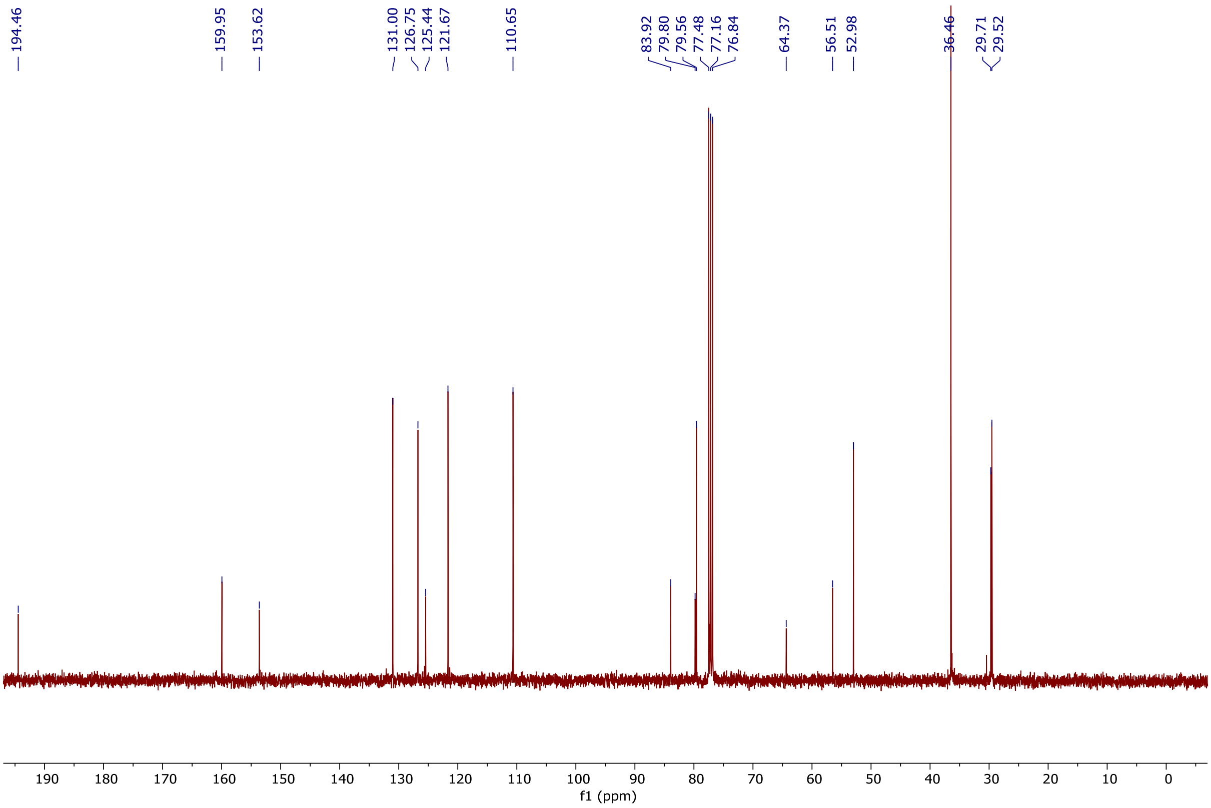

^{1}H NMR (400 MHz, $CDCl_3$) Methyl 3-(3-(2-cyano-2-(triphenyl-λ^{5}-phosphaneylidene)acetyl)-2,3-dihydrobenzofuran-3-yl)propiolate (29):

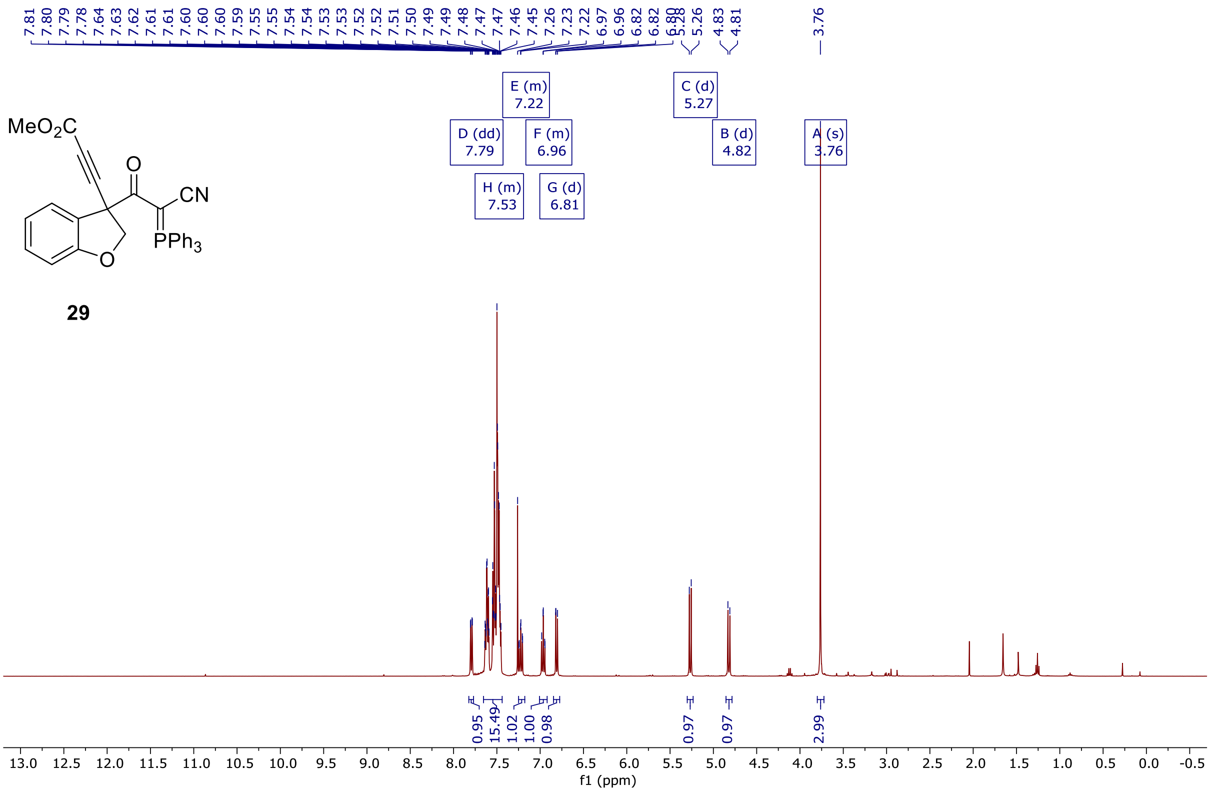

^{13}C NMR (101 MHz, $CDCl_3$):

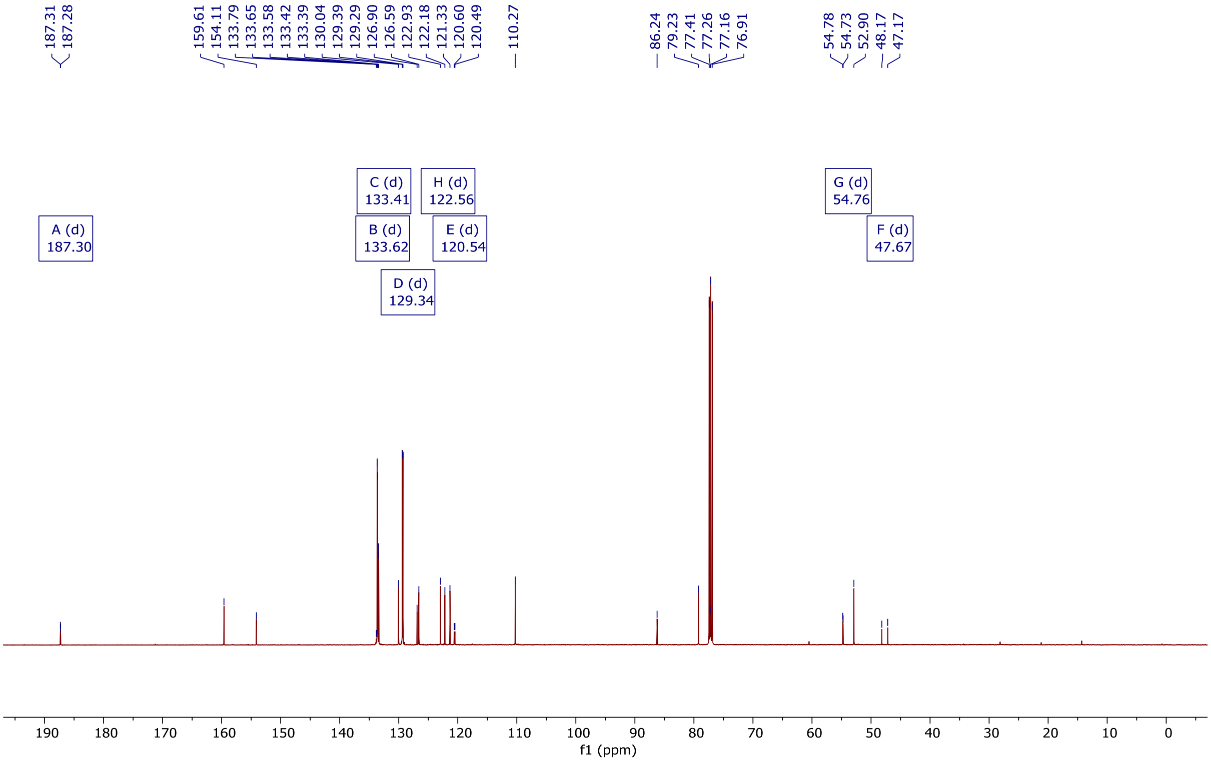

^{31}P NMR (126 MHz, $CDCl_3$):

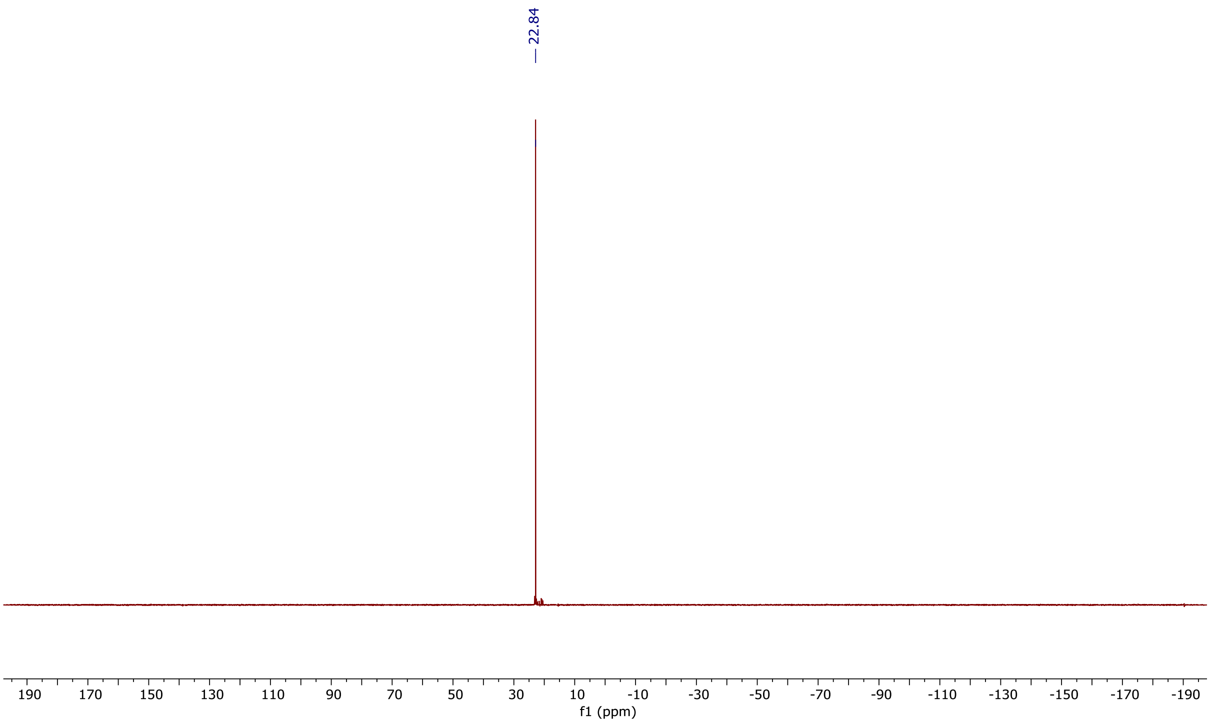

^{1}H NMR (400 MHz, $CDCl_3$) *tert*-Butyl 3-chloropropiolate (30):

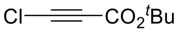

30

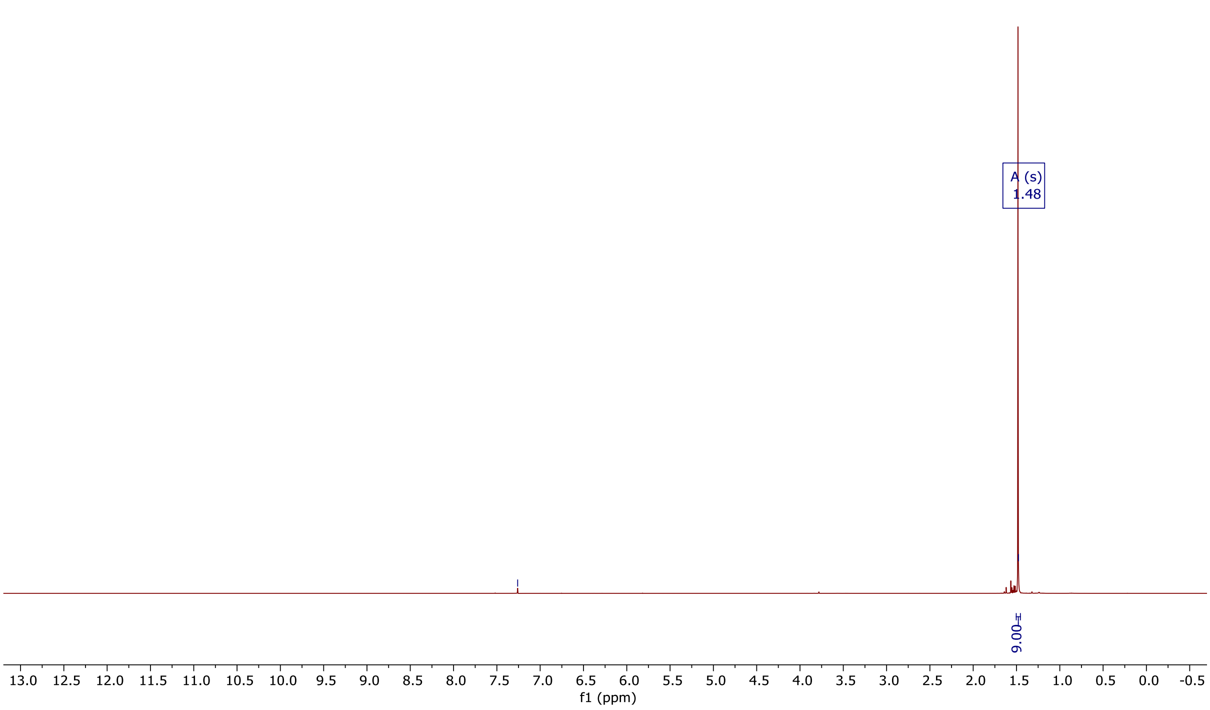

^{13}C NMR (101 MHz, $CDCl_3$):

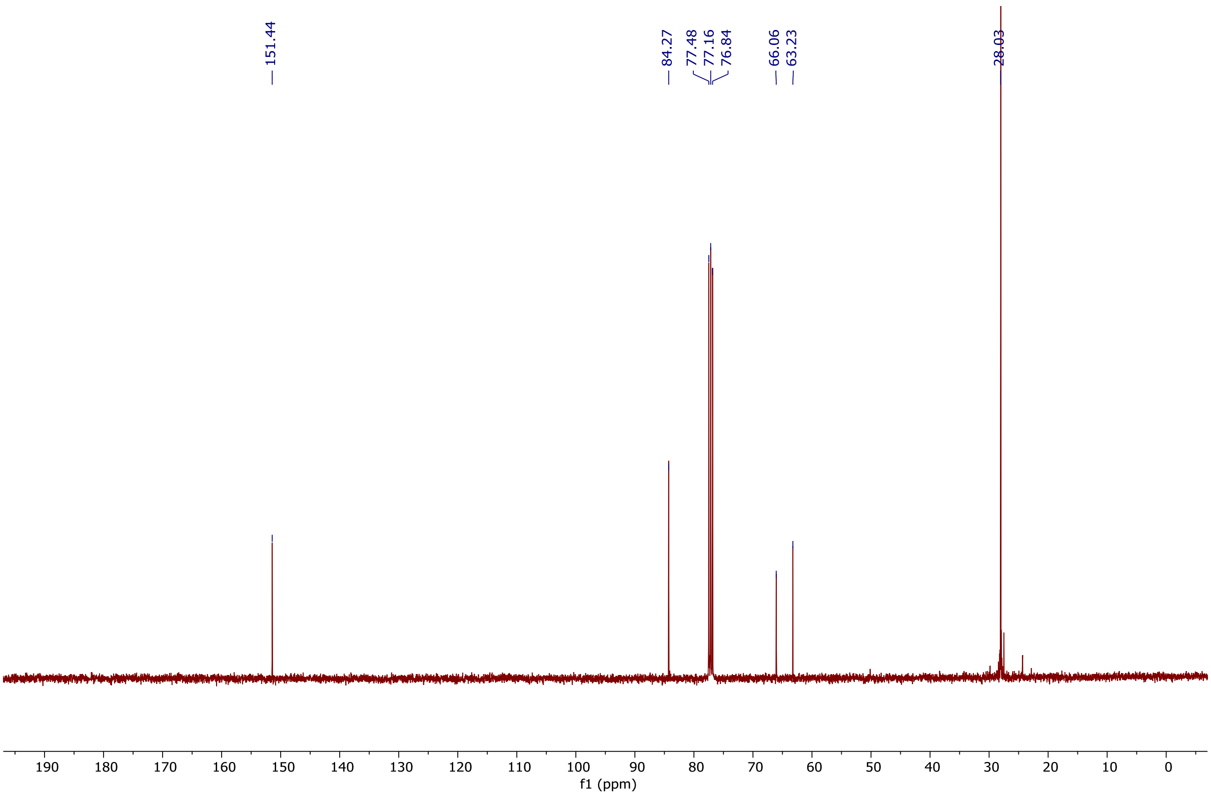

^{1}H NMR (400 MHz, $CDCl_3$) Methyl (*Z*)-3-(4-methoxy-4-oxobut-2-en-2-yl)-2,3-dihydrobenzofuran-3-carboxylate (31):

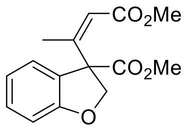

31

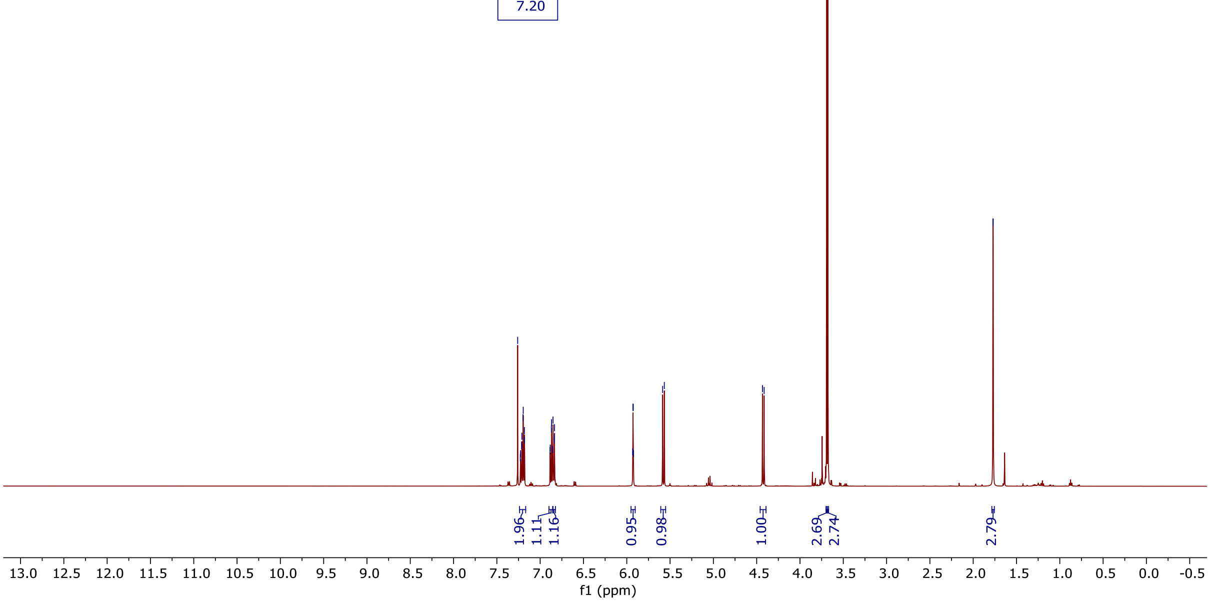

^{13}C NMR (101 MHz, $CDCl_3$):

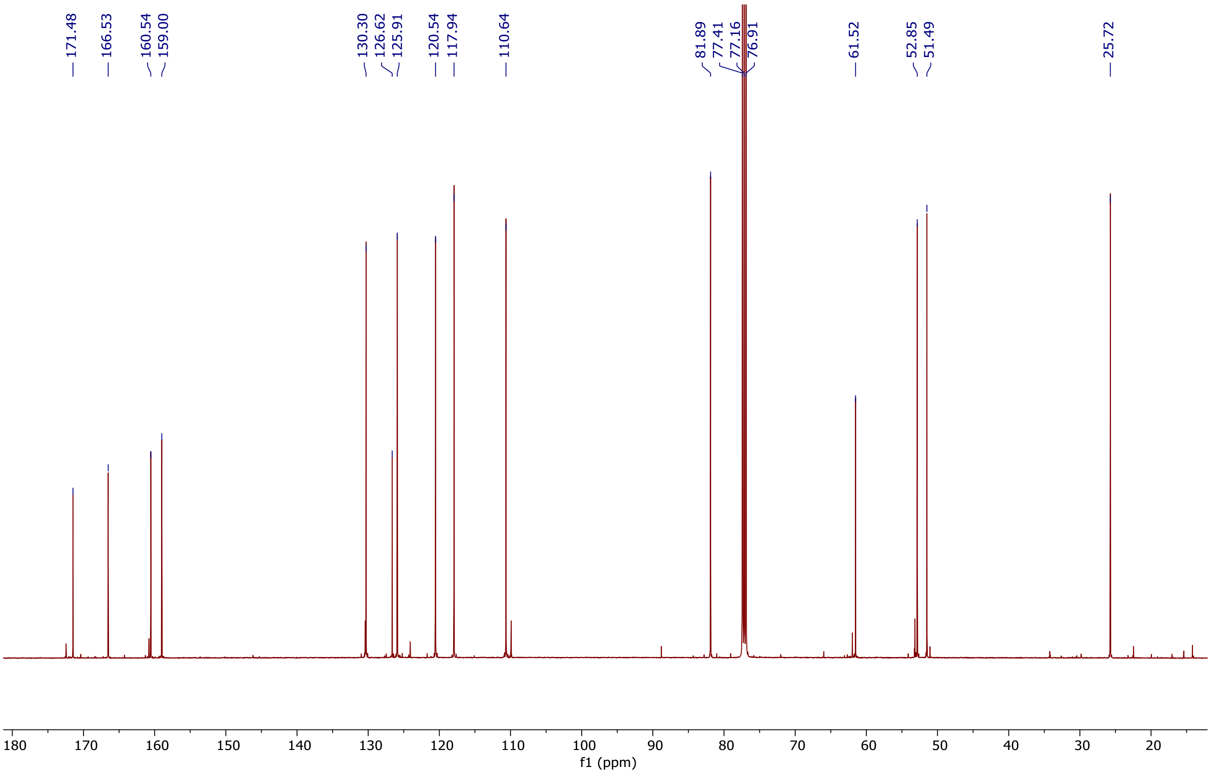

^{1}H NMR (400 MHz, CDCl$_3$) Methyl (*Z*)-3-(1-methoxy-1-oxohept-2-en-3-yl)-2,3-dihydrobenzofuran-3-carboxylate (32):

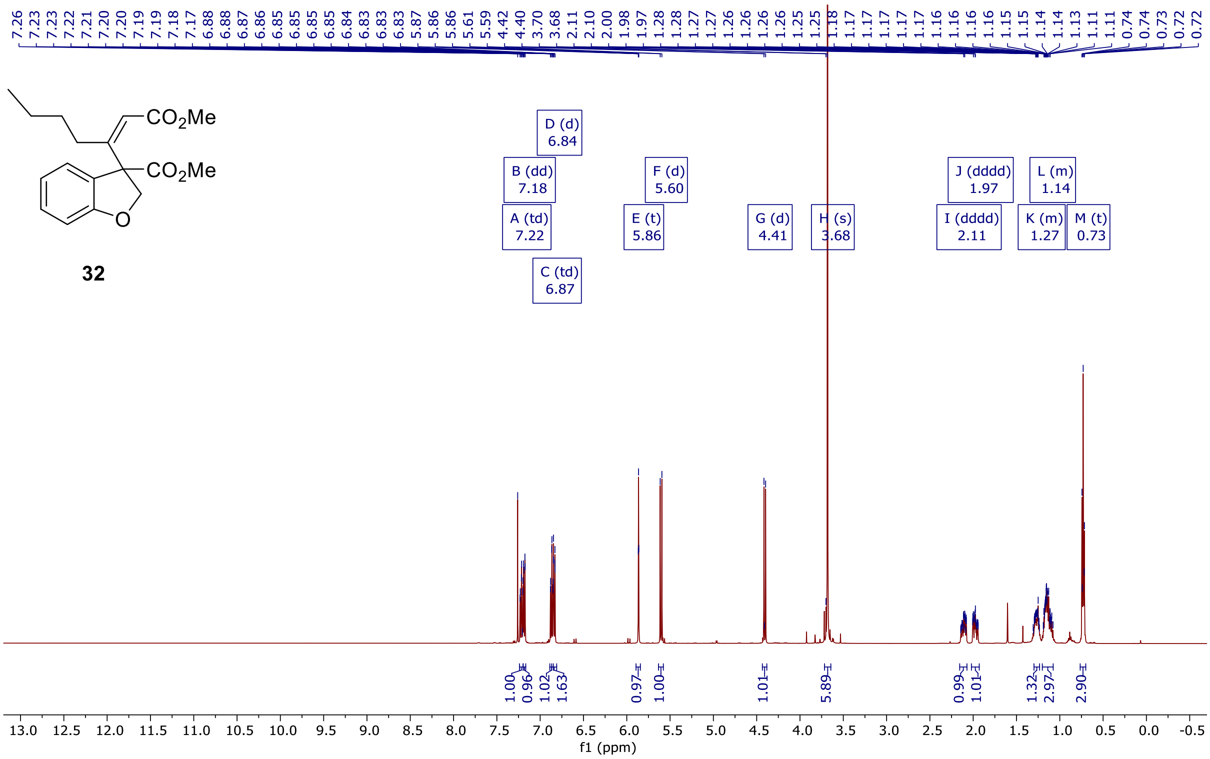

^{13}C NMR (101 MHz, CDCl$_3$):

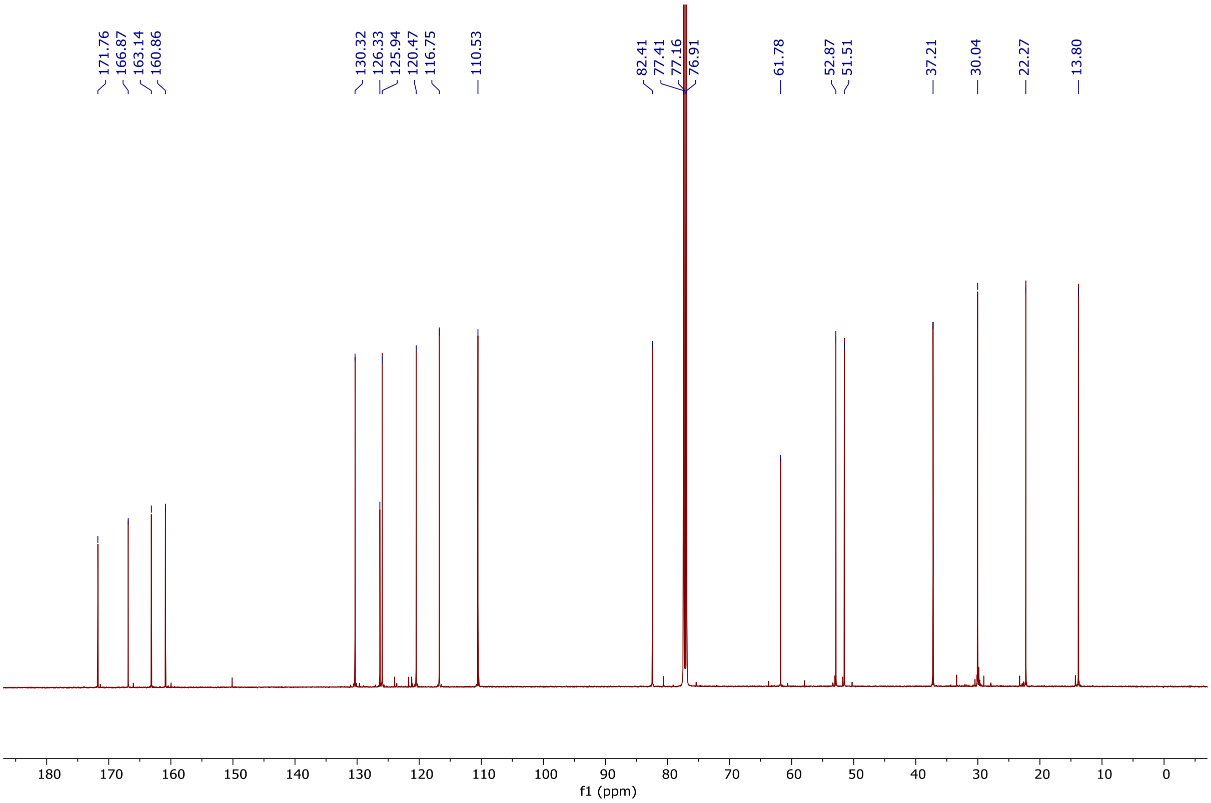

^{1}H NMR (500 MHz, $CDCl_3$) Methyl (*Z*)-3-(1-methoxy-5-methyl-1-oxohex-2-en-3-yl)-2,3-dihydrobenzofuran-3-carboxylate (33)

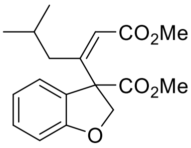

33

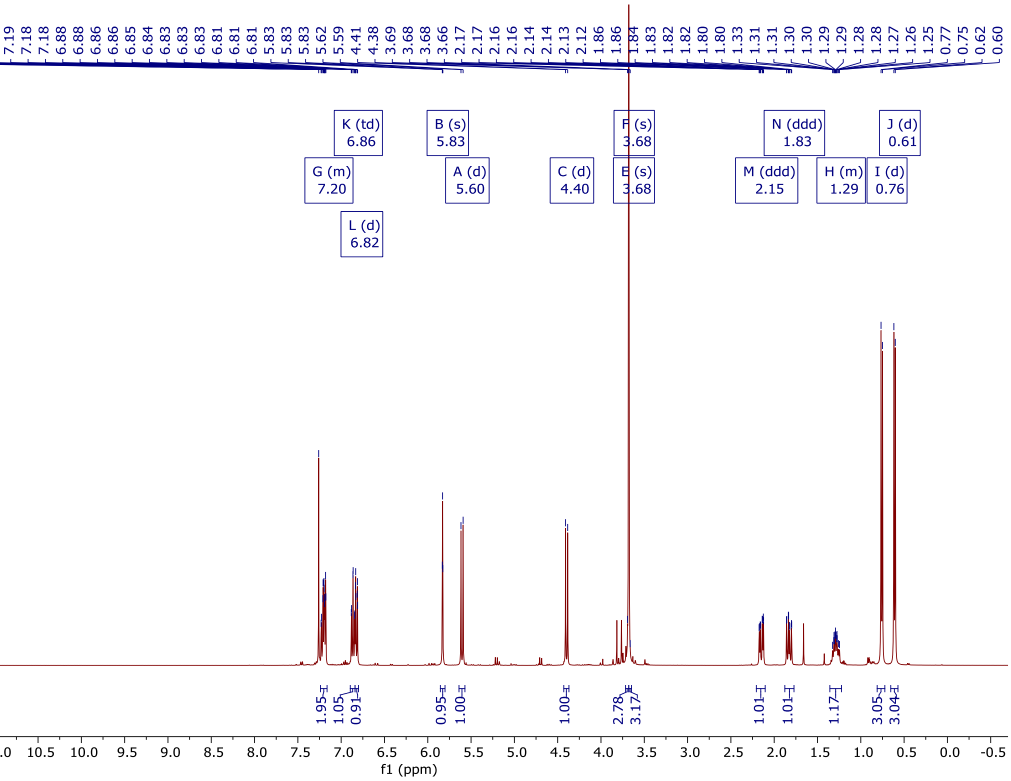

^{13}C NMR (126 MHz, $CDCl_3$):

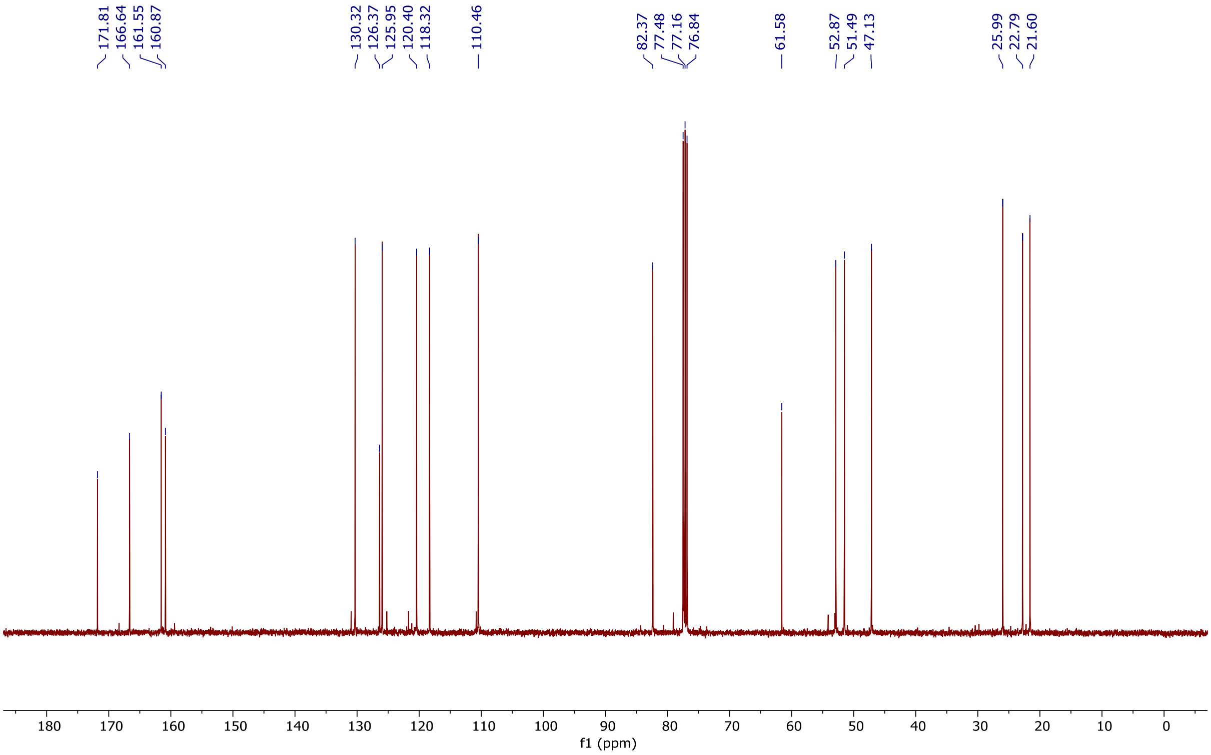

^{1}H NMR (400 MHz, $CDCl_3$) 2-Bromo-6-hydroxybenzaldehyde (36):

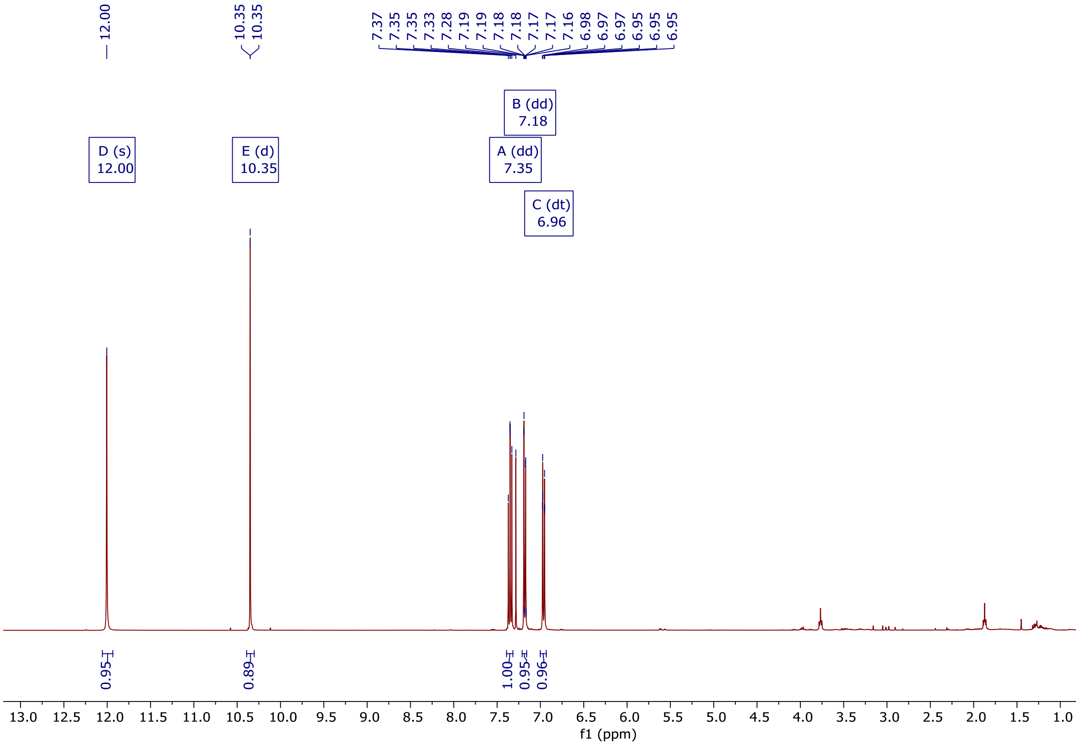

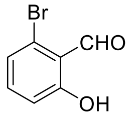

36

^{13}C NMR (101 MHz, $CDCl_3$):

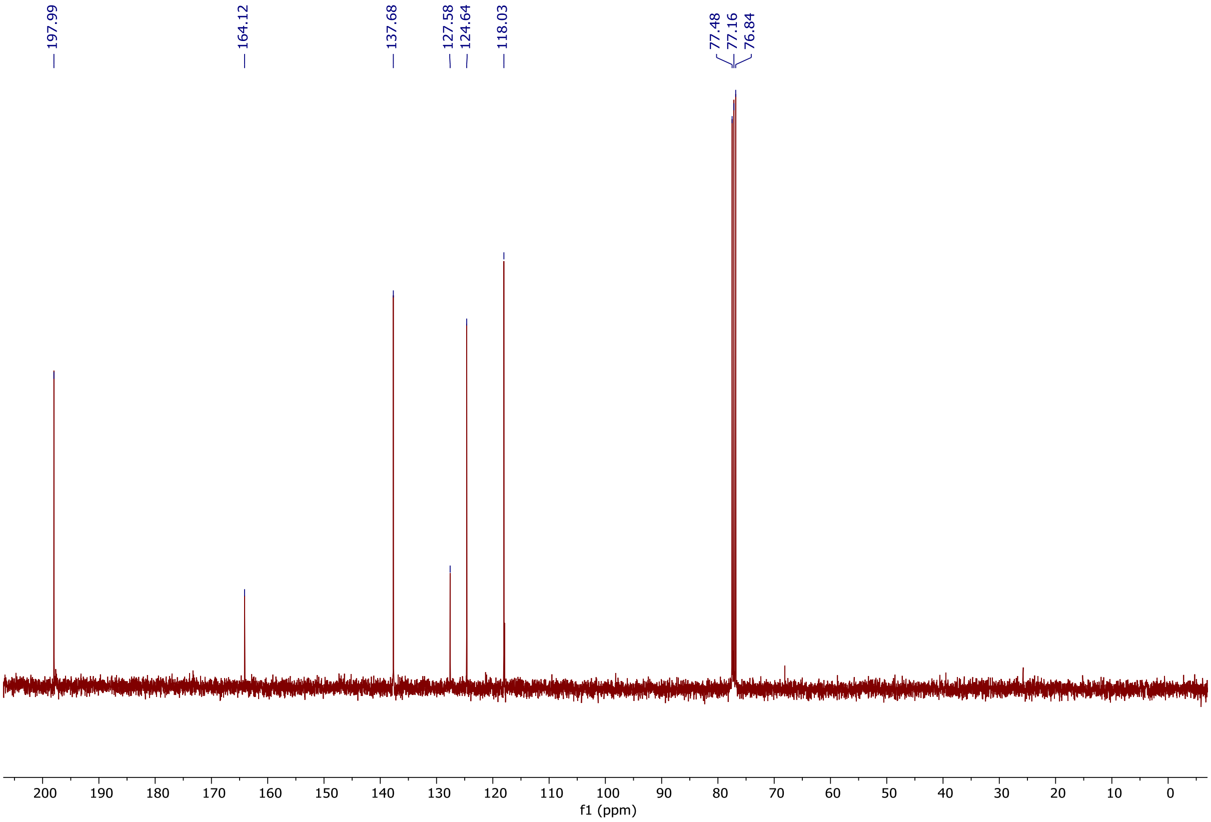

^{1}H NMR (400 MHz, $CDCl_3$) Ethyl 4-bromobenzofuran-3-carboxylate (37):

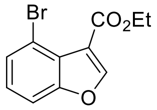

37

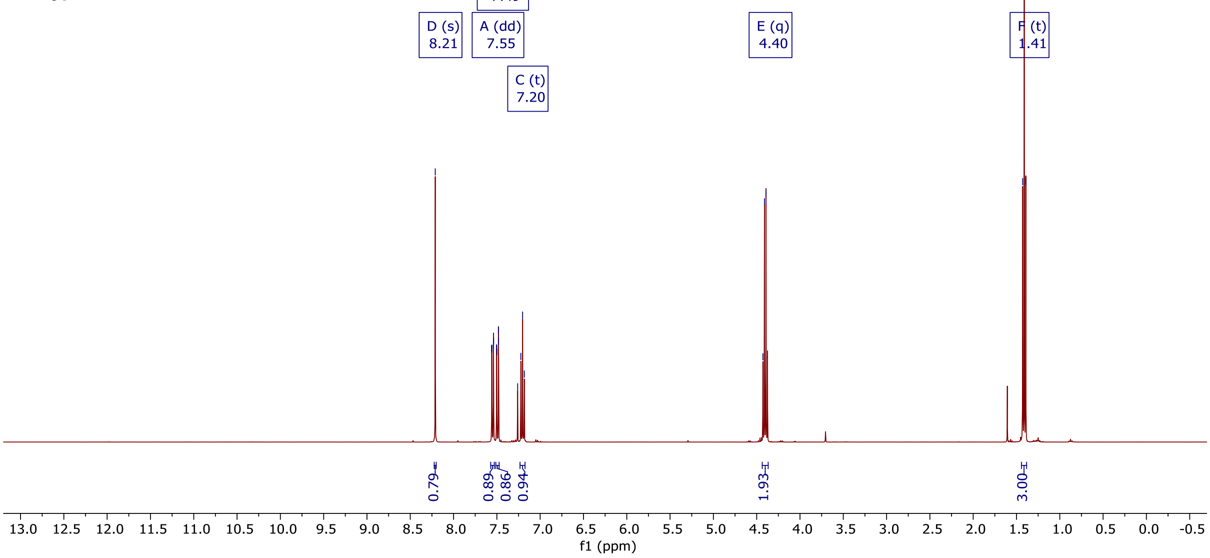

^{13}C NMR (101 MHz, $CDCl_3$):

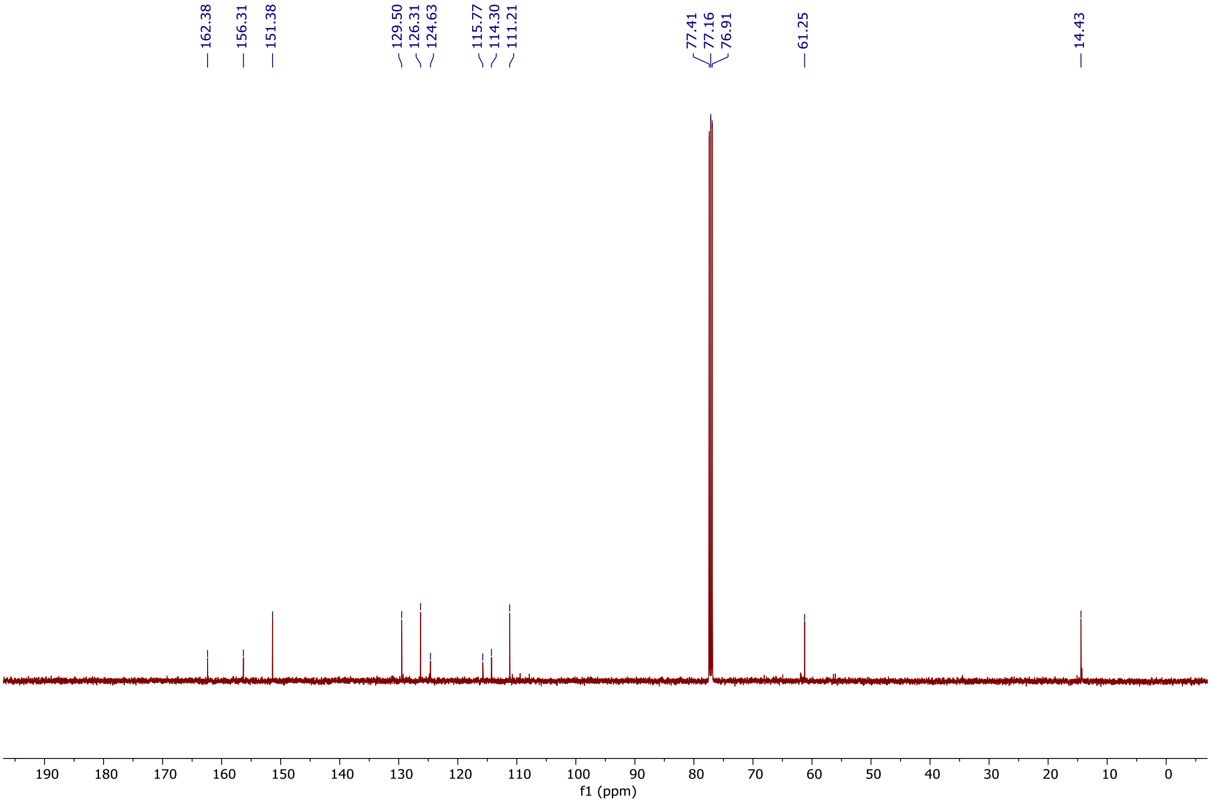

^{1}H NMR (400 MHz, $CDCl_3$) Ethyl 4-(tributylstannyl)benzofuran-3-carboxylate (40):

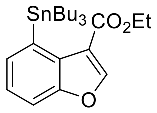

40

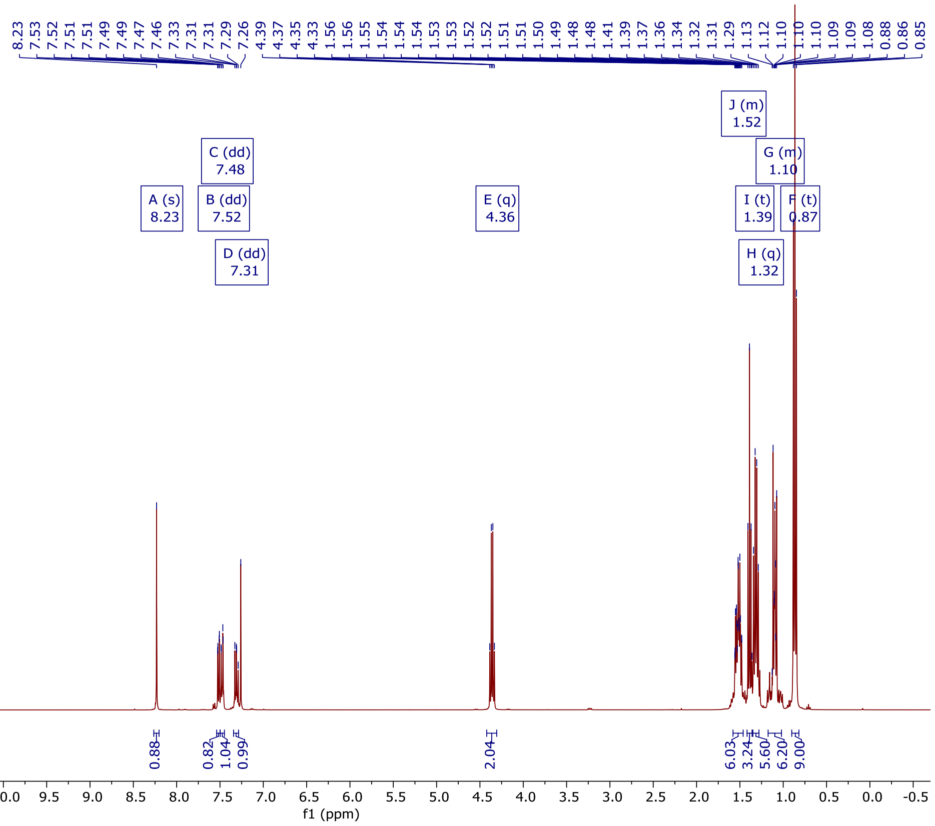

^{13}C NMR (101 MHz, $CDCl_3$):

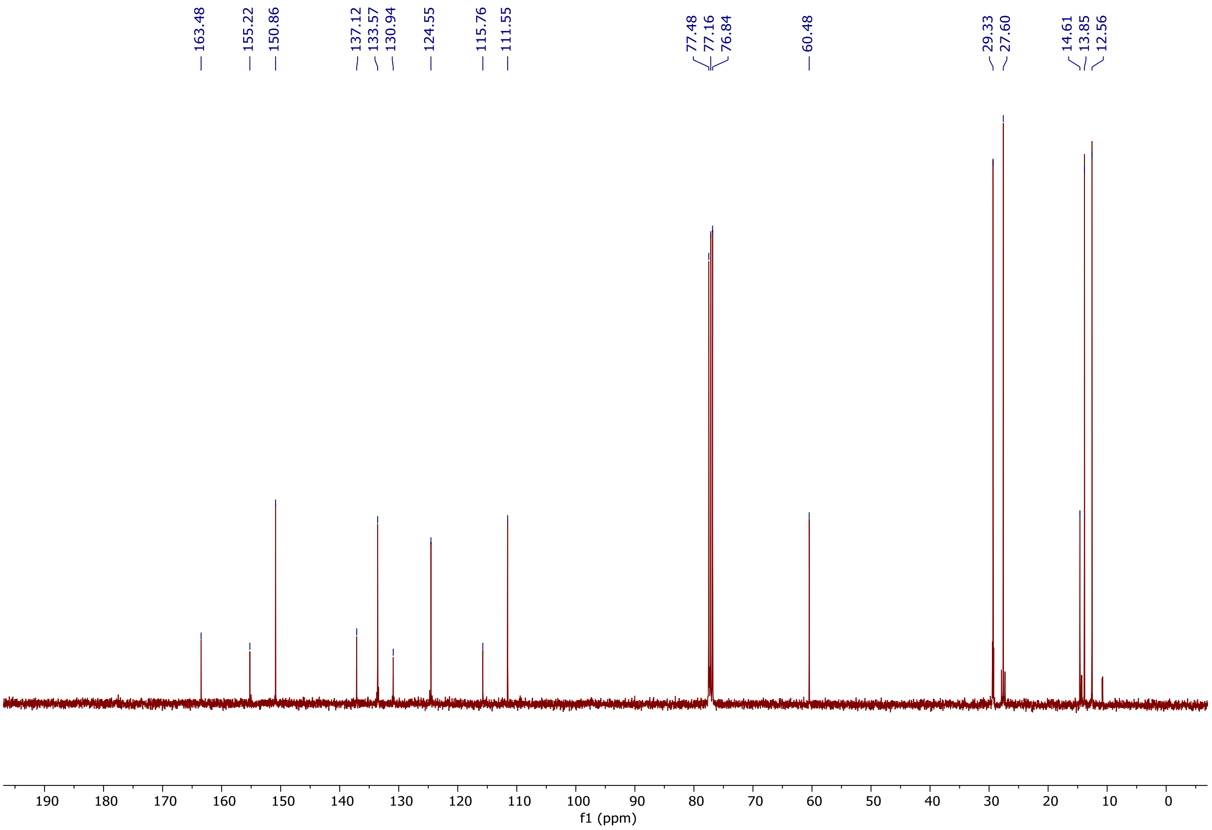

^{1}H NMR (400 MHz, $CDCl_3$) Methyl 4-(tributylstannyl)-2,3-dihydrobenzofuran-3-carboxylate (41)

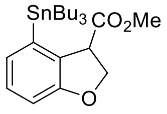

41

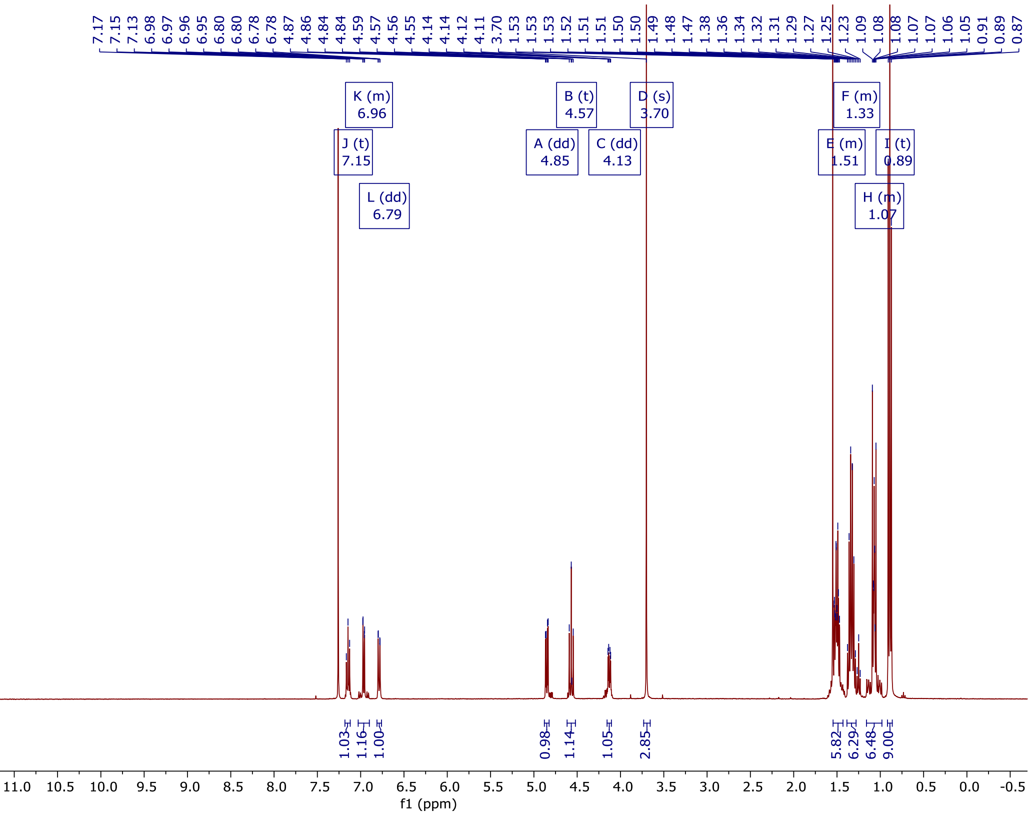

^{13}C NMR (101 MHz, $CDCl_3$):

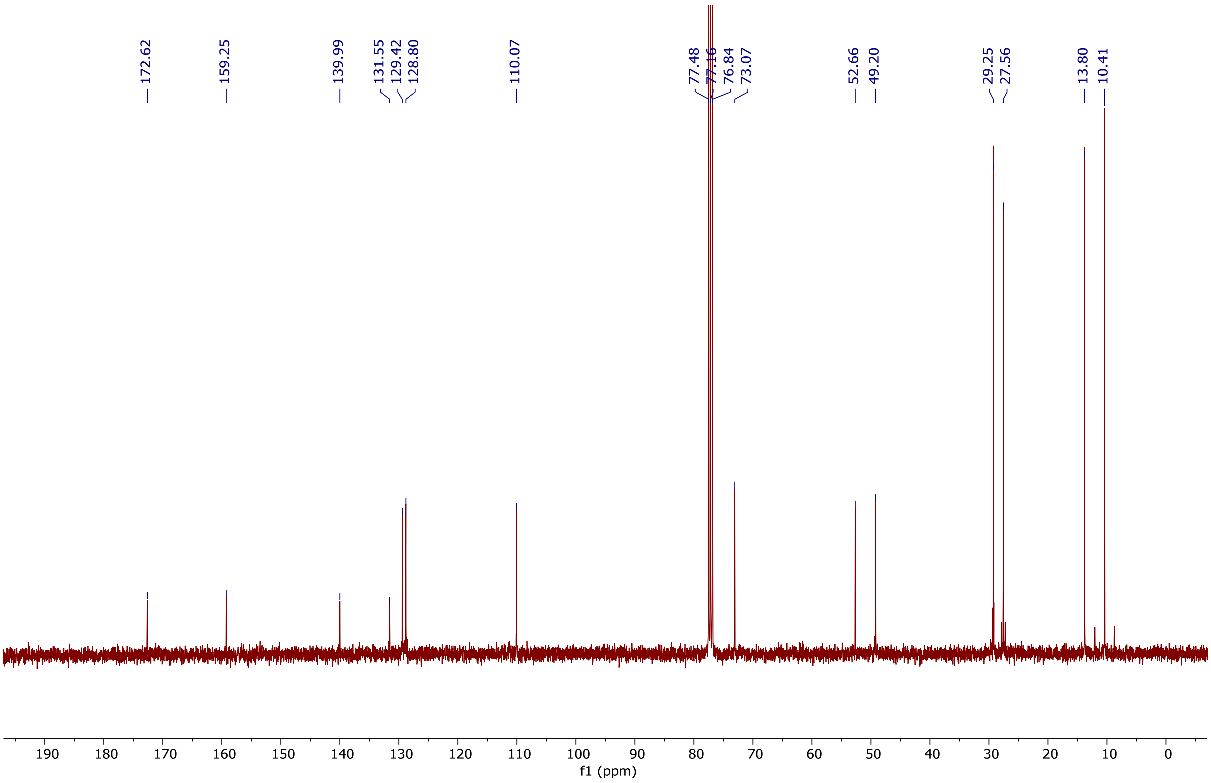

^{1}H NMR (400 MHz, CDCl$_3$) Methyl 3-(3-methoxy-3-oxoprop-1-yn-1-yl)-4-(tributylstannyl)-2,3-dihydrobenzofuran-3-carboxylate (42):

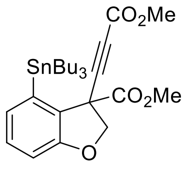

42

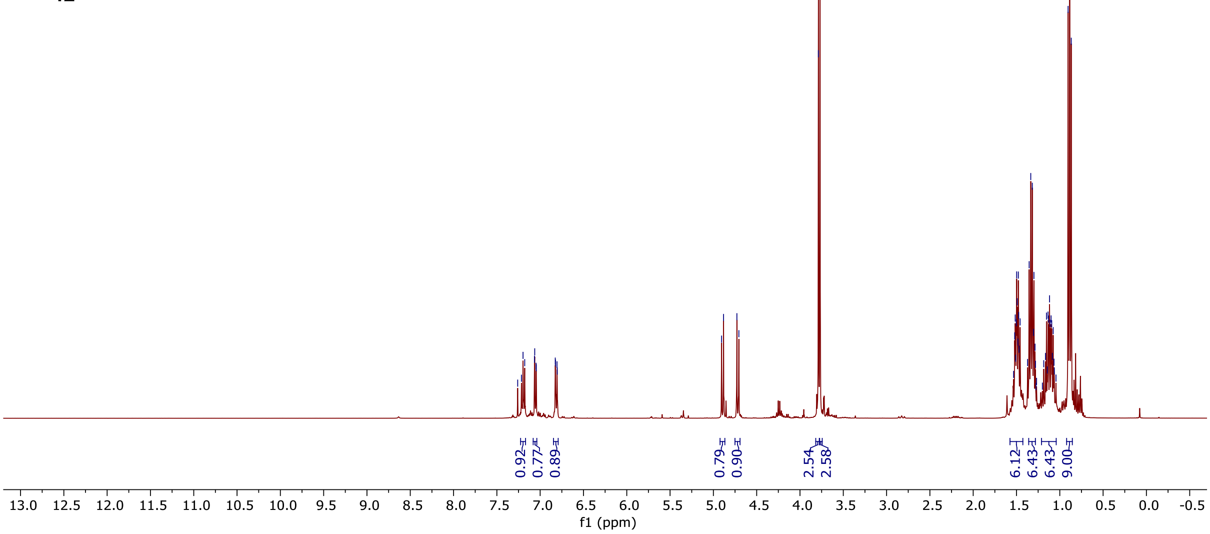

^{13}C NMR (101 MHz, CDCl$_3$):

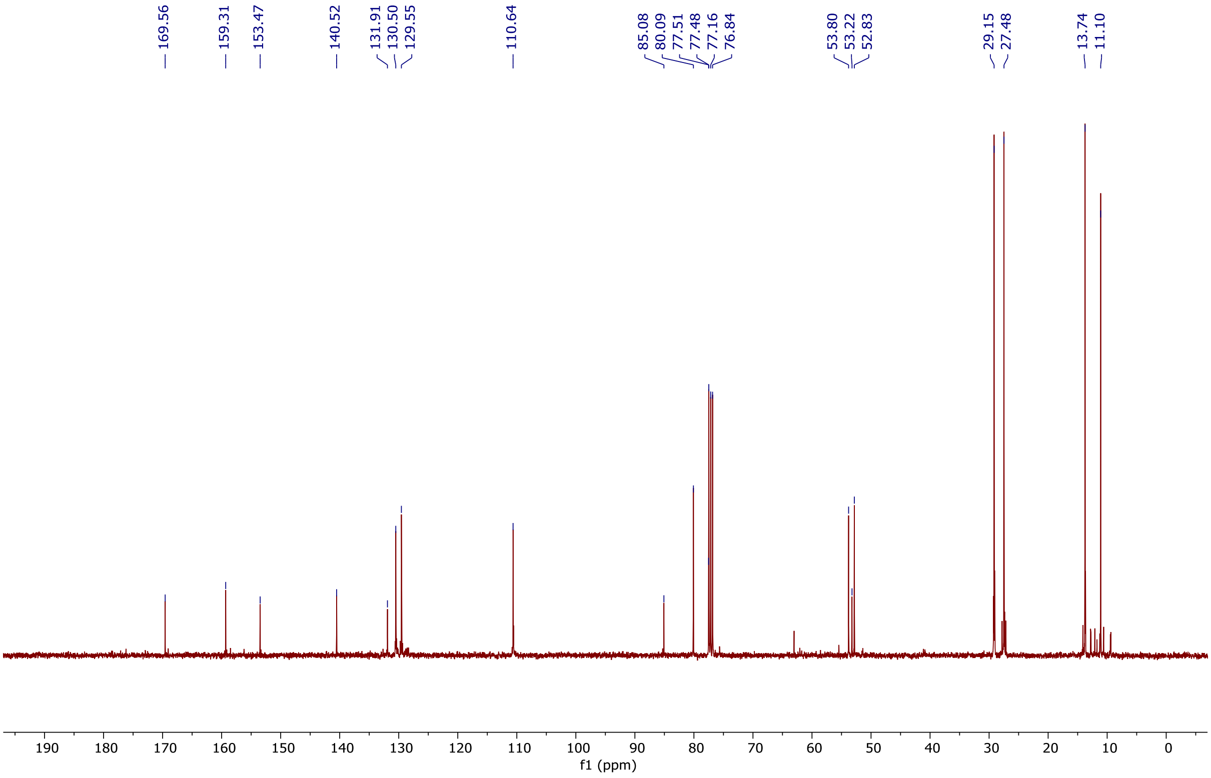

^{1}H NMR (400 MHz, $CDCl_3$) Methyl 4-iodo-3-(3-methoxy-3-oxoprop-1-yn-1-yl)-2,3-dihydrobenzofuran-3-carboxylate (43):

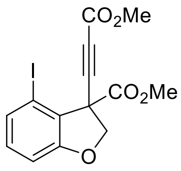

43

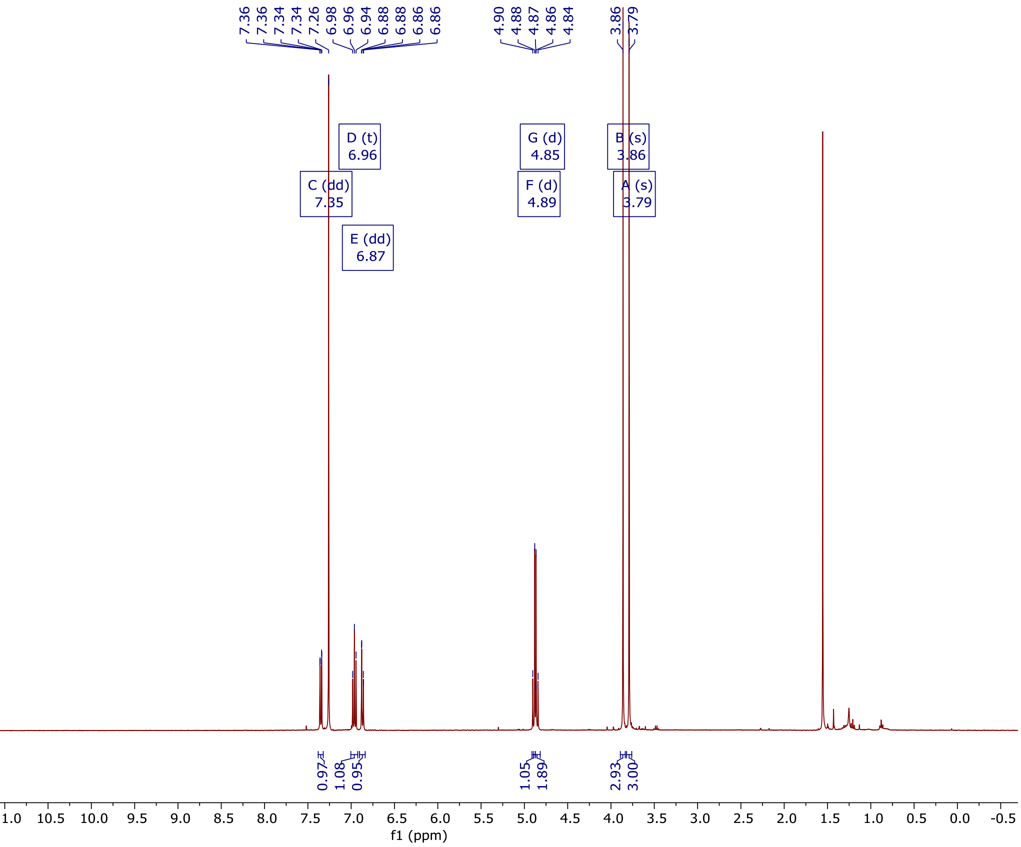

^{13}C NMR (101 MHz, $CDCl_3$):

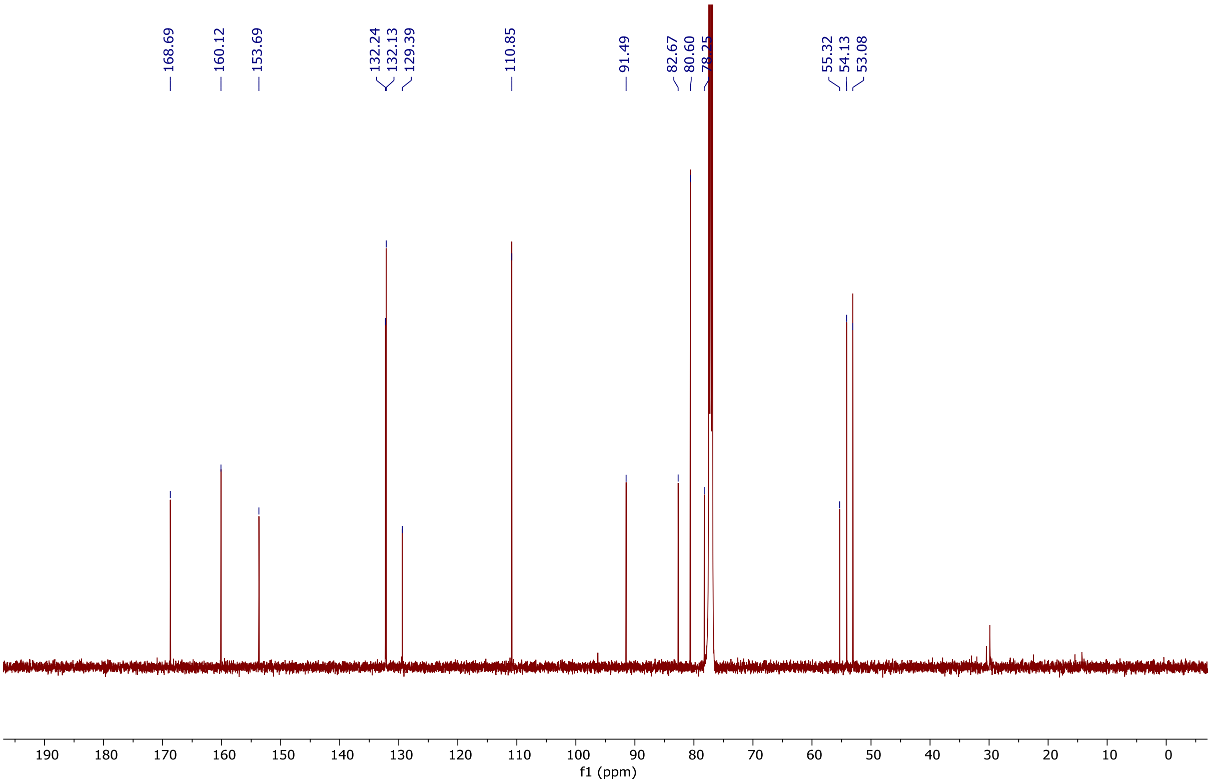

^{1}H NMR (400 MHz, $CDCl_3$) 5-Iodopent-1-ene (44):

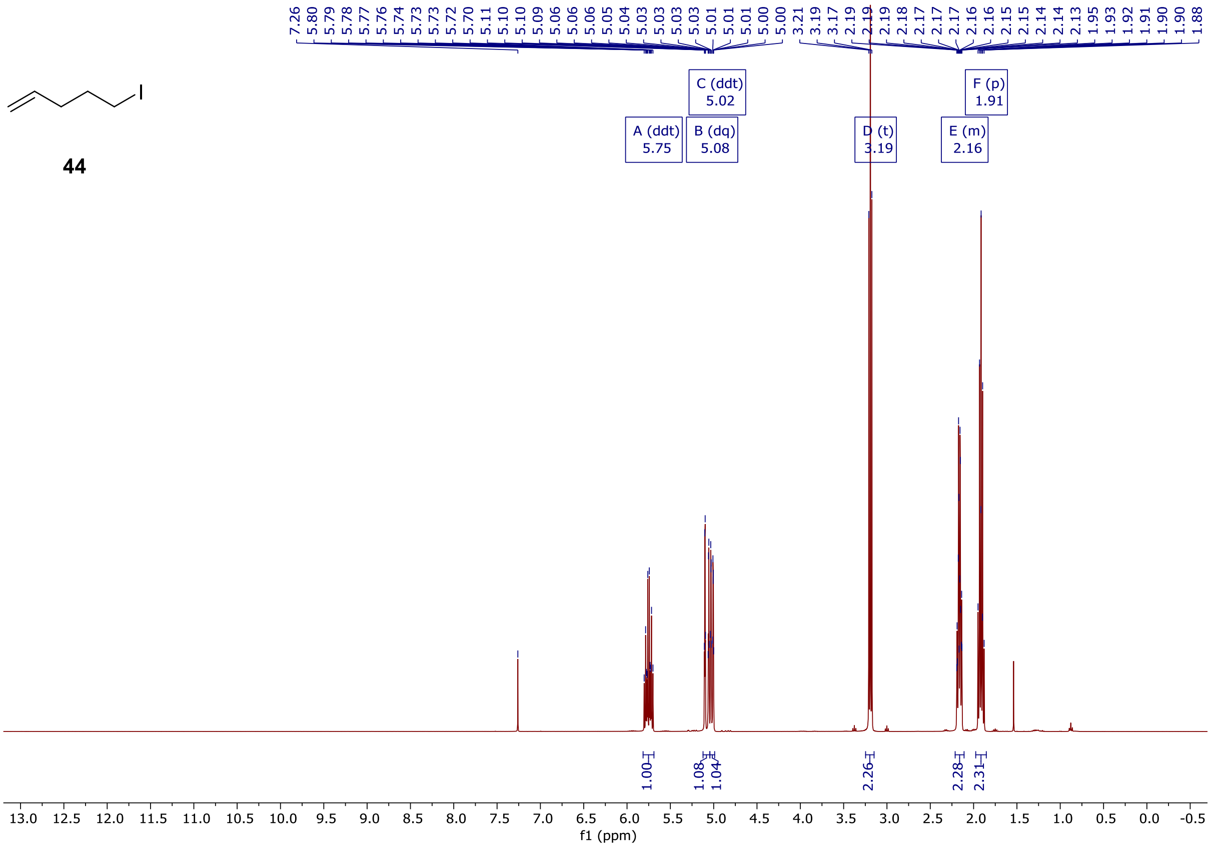

^{13}C NMR (101 MHz, $CDCl_3$):

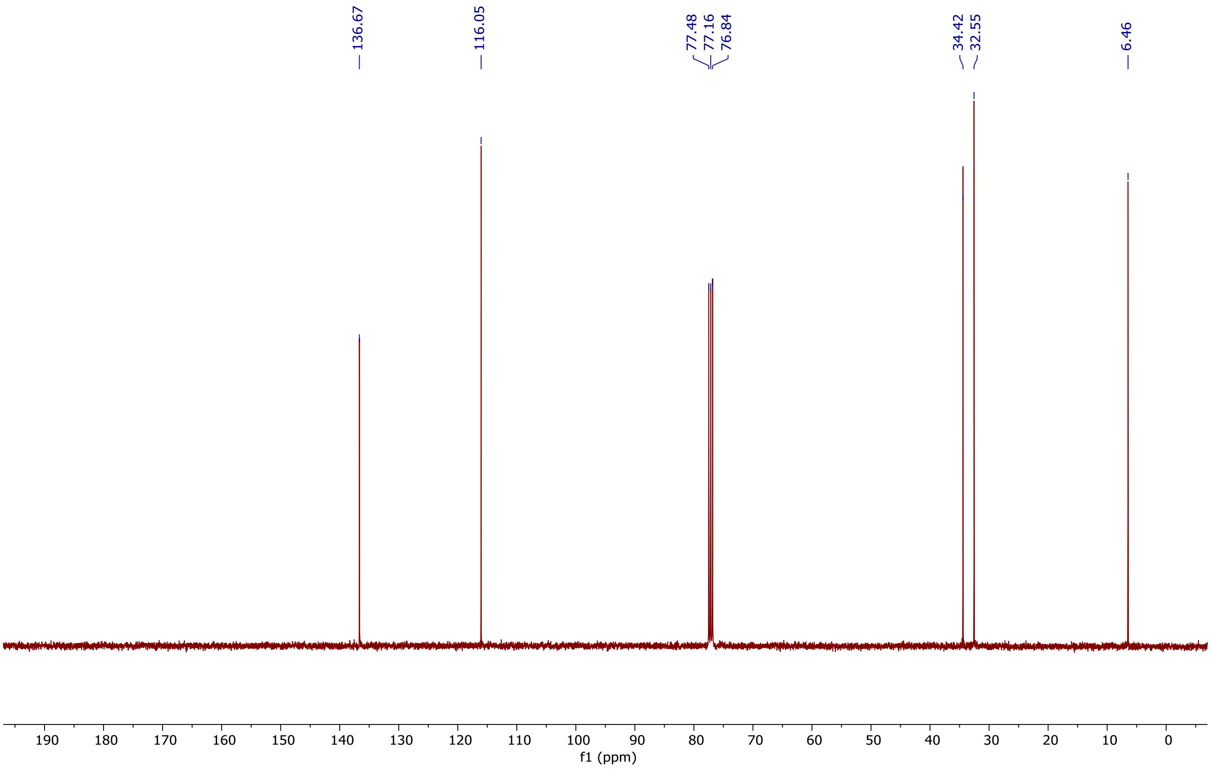

^{1}H NMR (400 MHz, $CDCl_3$) Methyl (*Z*)-4-iodo-3-(1-methoxy-1-oxoocta-2,7-dien-3-yl)-2,3-dihydrobenzofuran-3-carboxylate (45):

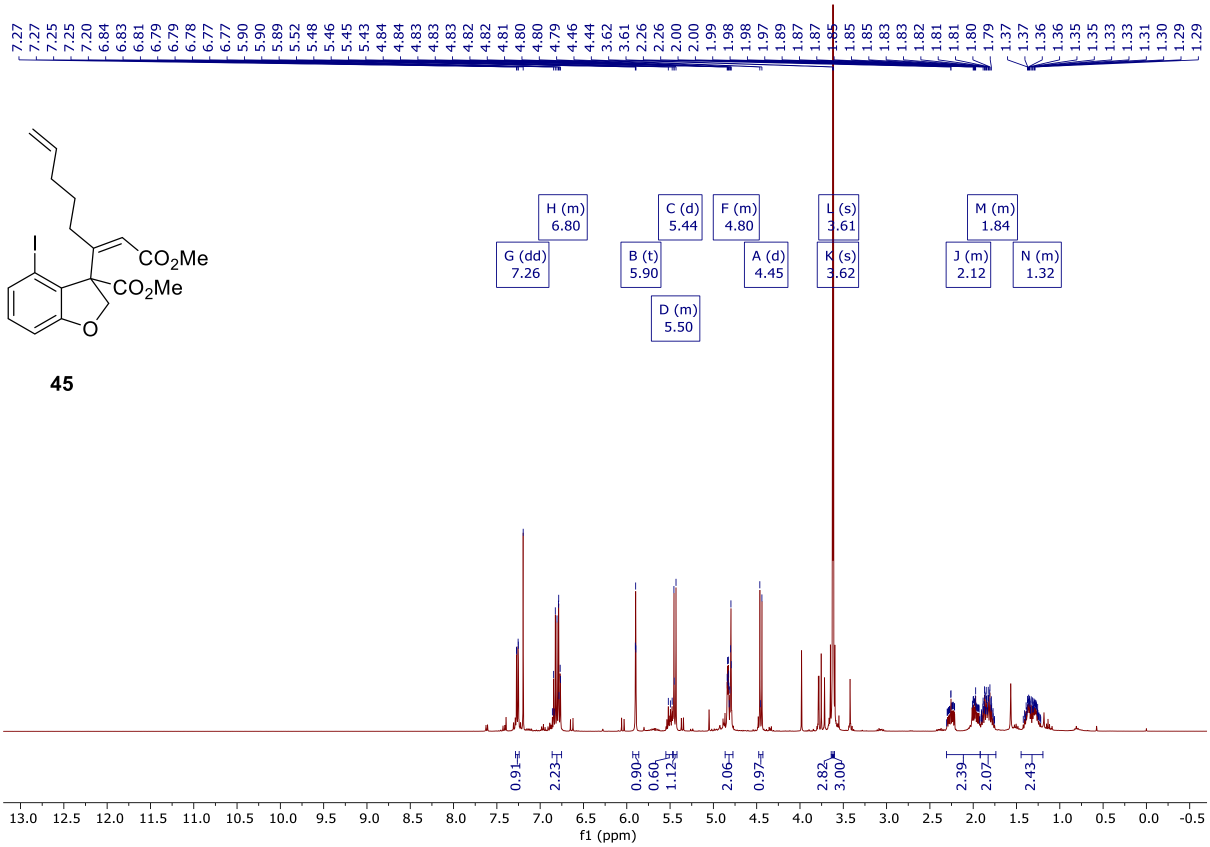

^{13}C NMR (126 MHz, $CDCl_3$):

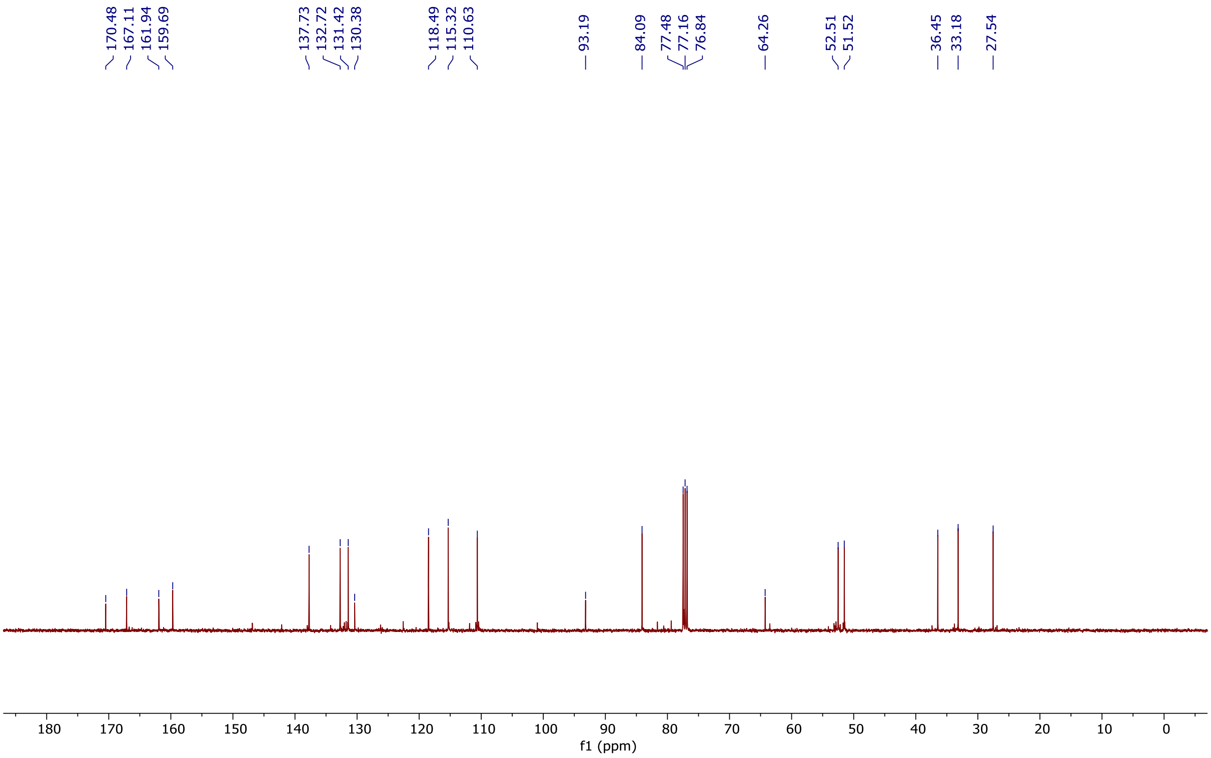

^{1}H NMR (400 MHz, CDCl₃) Methyl (*Z*)-4-iodo-3-(1-methoxy-1-oxoocta-2,7-dien-3-yl)-2,3-dihydrobenzofuran-3-carboxylate (45) - Analytical Sample:

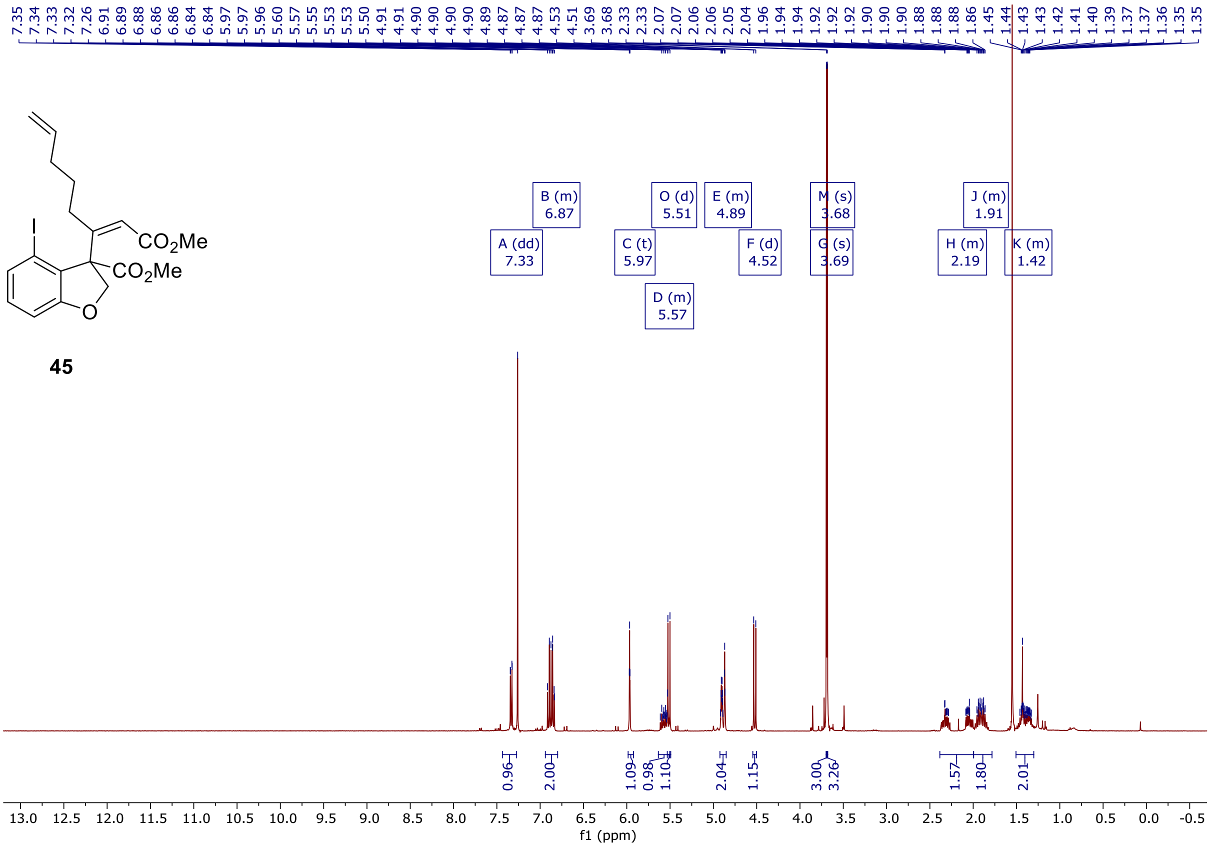

^{13}C NMR (151 MHz, CDCl₃):

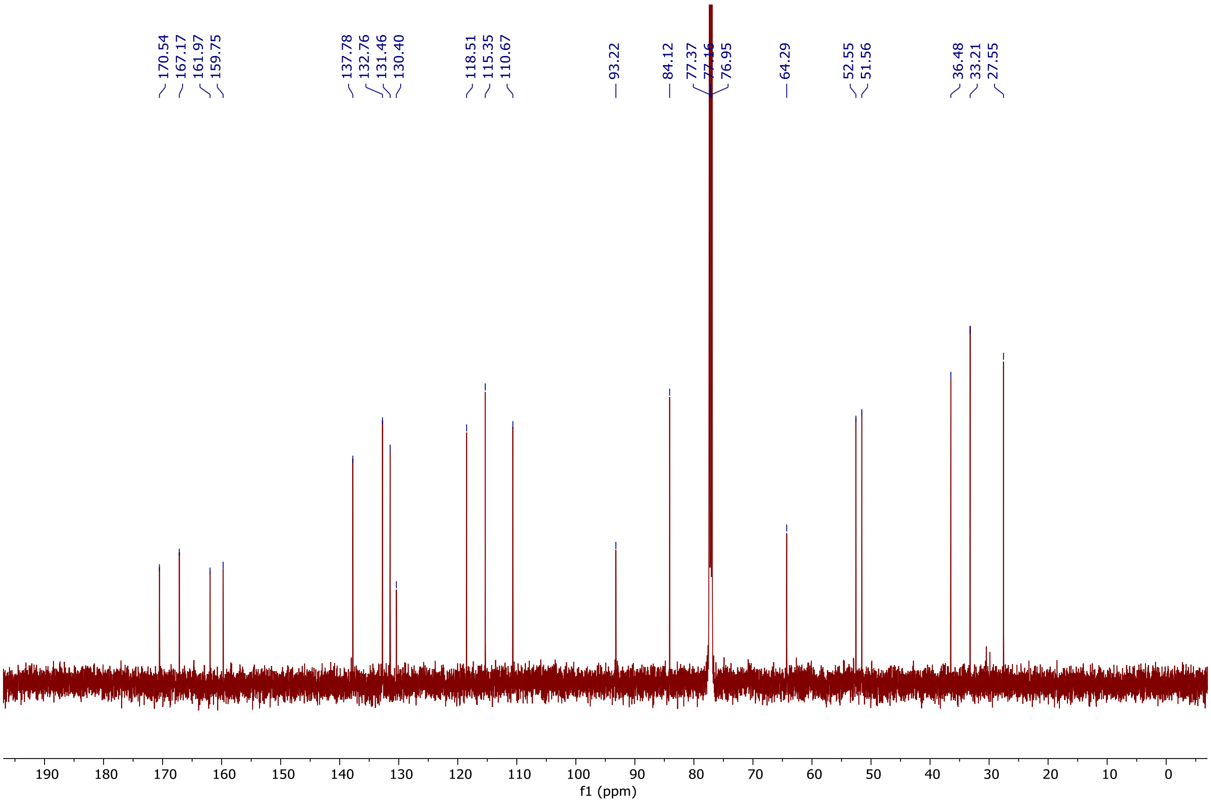

^{1}H NMR (400 MHz, $CDCl_3$) Methyl (*Z*)-10-(2-methoxy-2-oxoethylidene)-6-methylene-7,8,9,10-tetrahydro-1*H*-cycloocta[*cd*]benzofuran-10a(6*H*)-carboxylate (46):

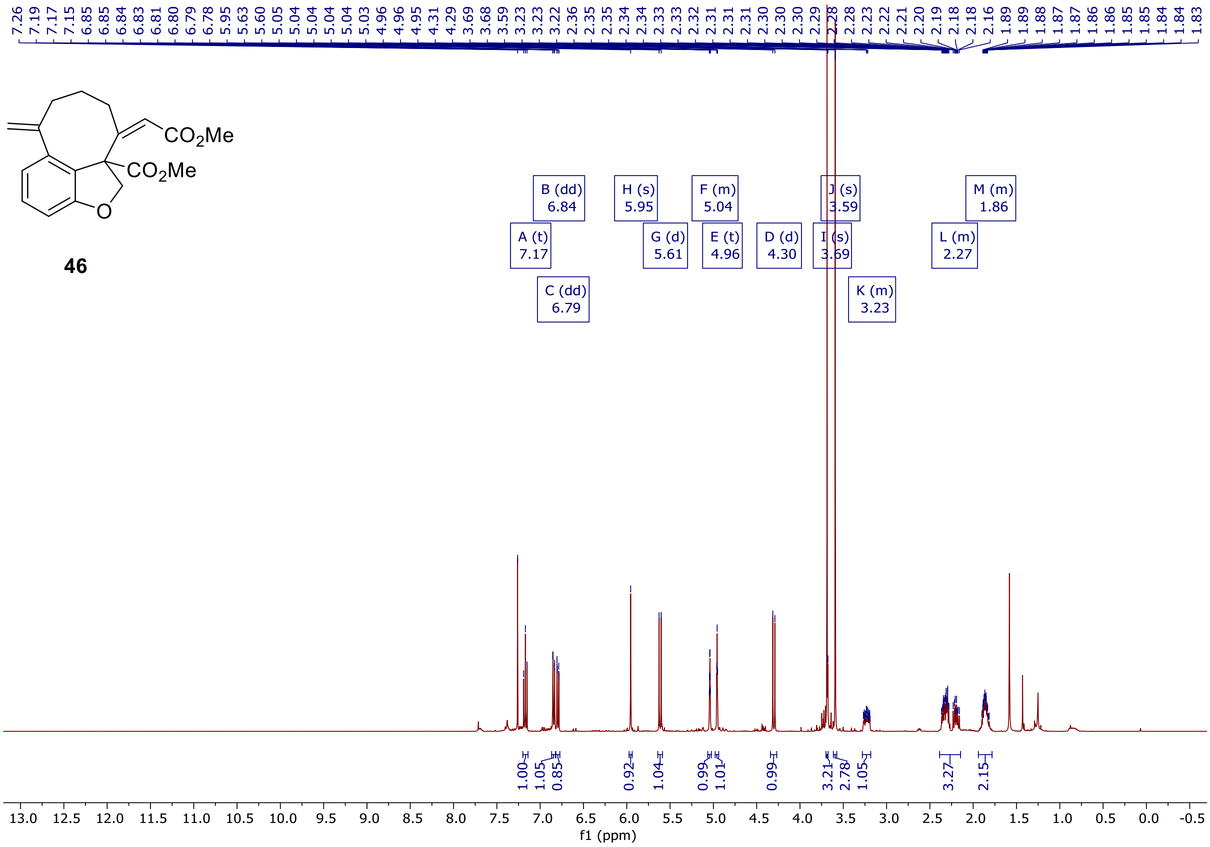

^{13}C NMR (101 MHz, $CDCl_3$):

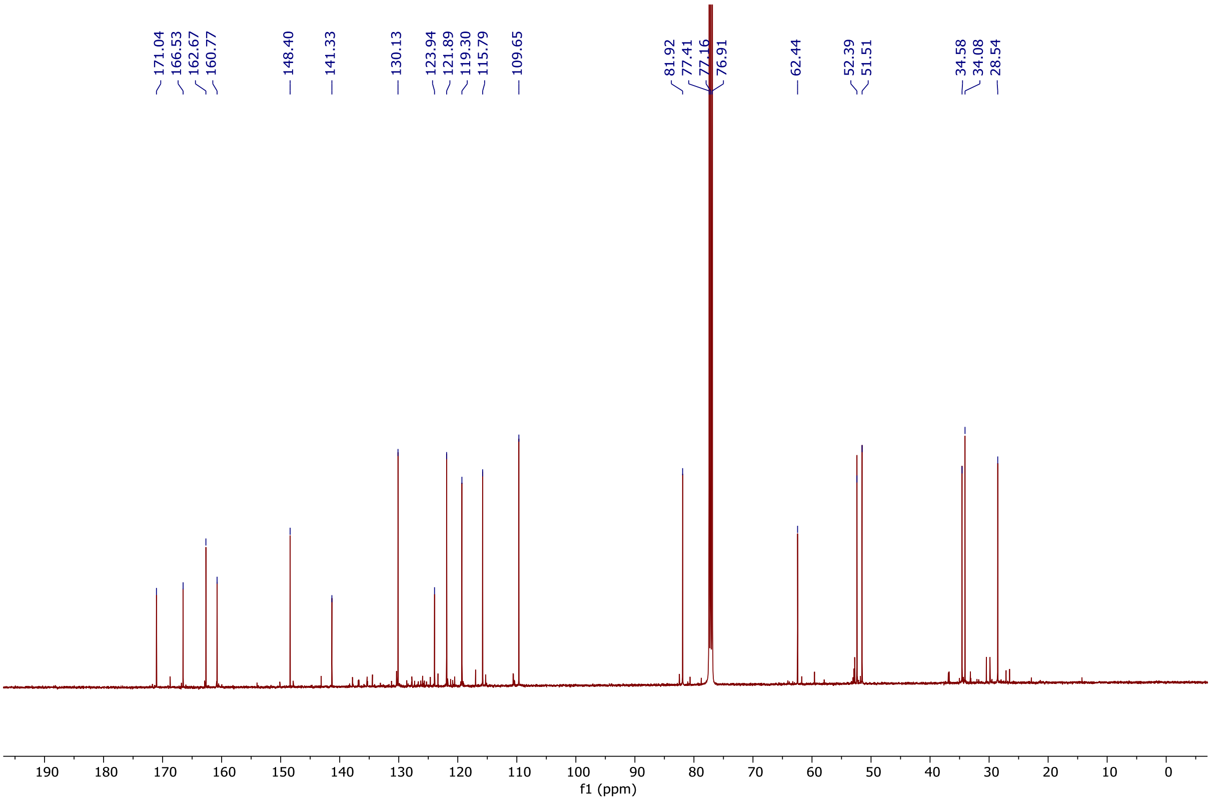

^{1}H NMR (400 MHz, $CDCl_3$) Methyl (*Z*)-10-(2-methoxy-2-oxoethylidene)-6-oxo-7,8,9,10-tetrahydro-1*H*-cycloocta[cd]benzofuran-10a(6*H*)-carboxylate (47):

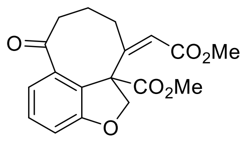

47

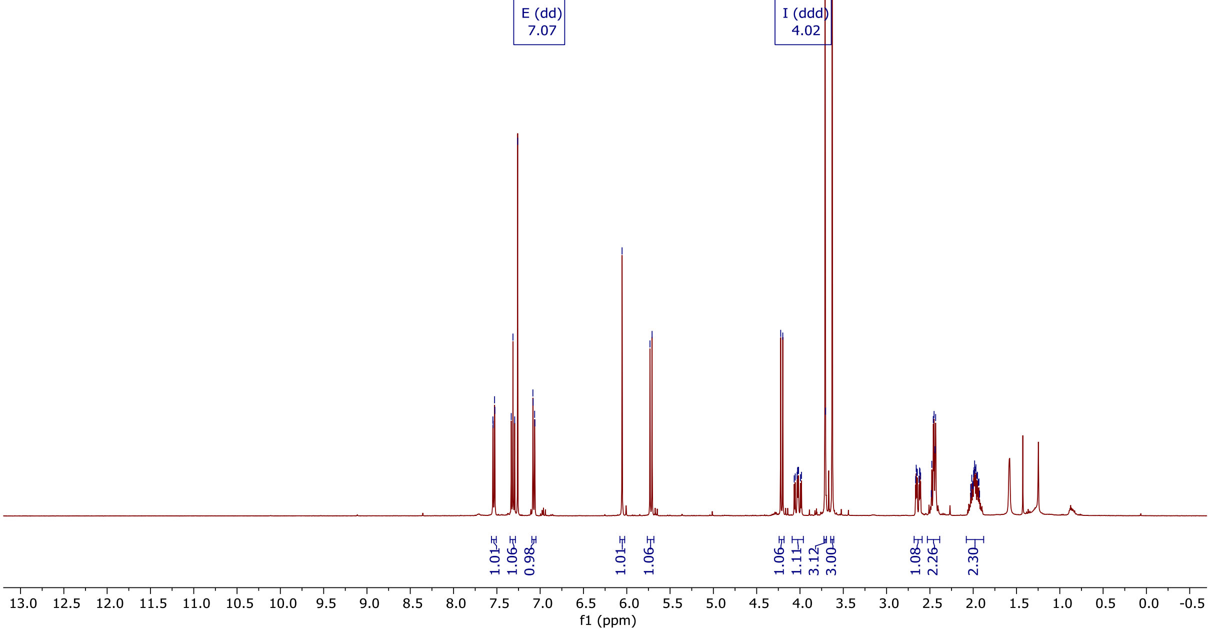

^{13}C NMR (101 MHz, $CDCl_3$):

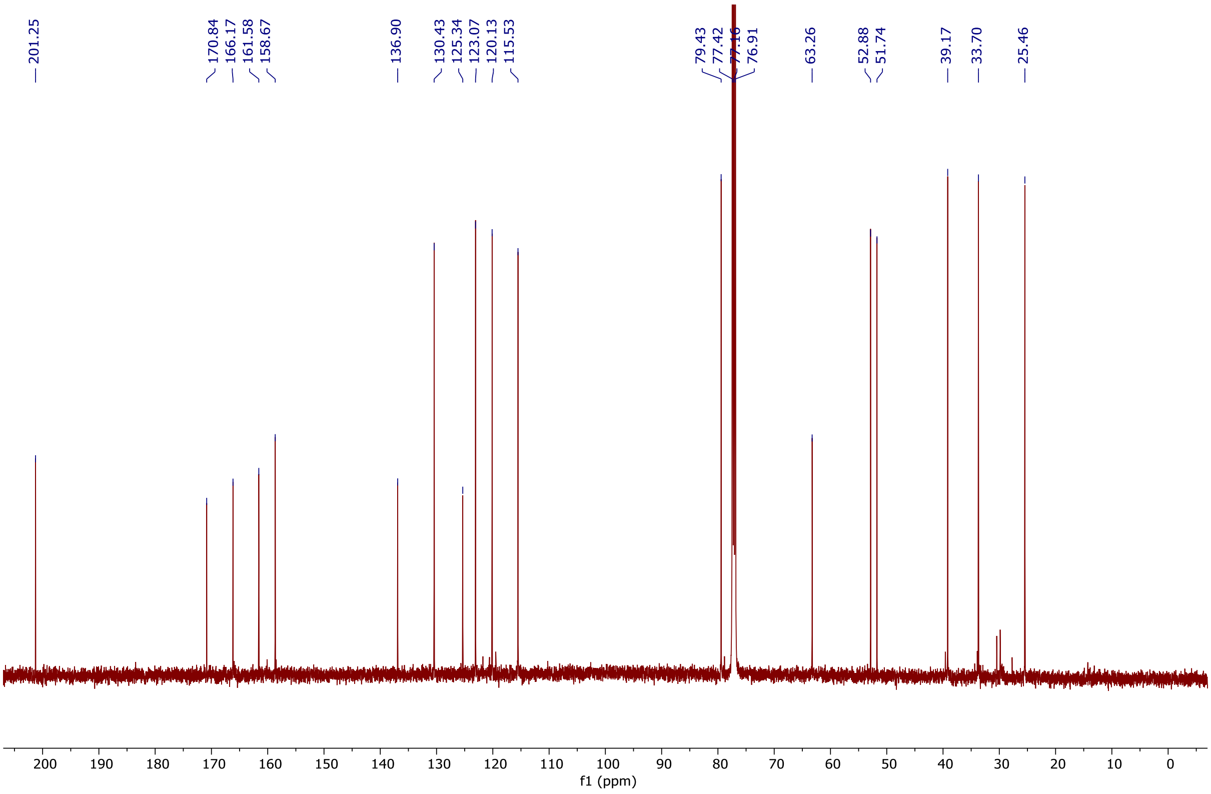

^{1}H NMR (400 MHz, $CDCl_3$) 2,3-Dihydrobenzofuran-3-carboxylic acid (48):

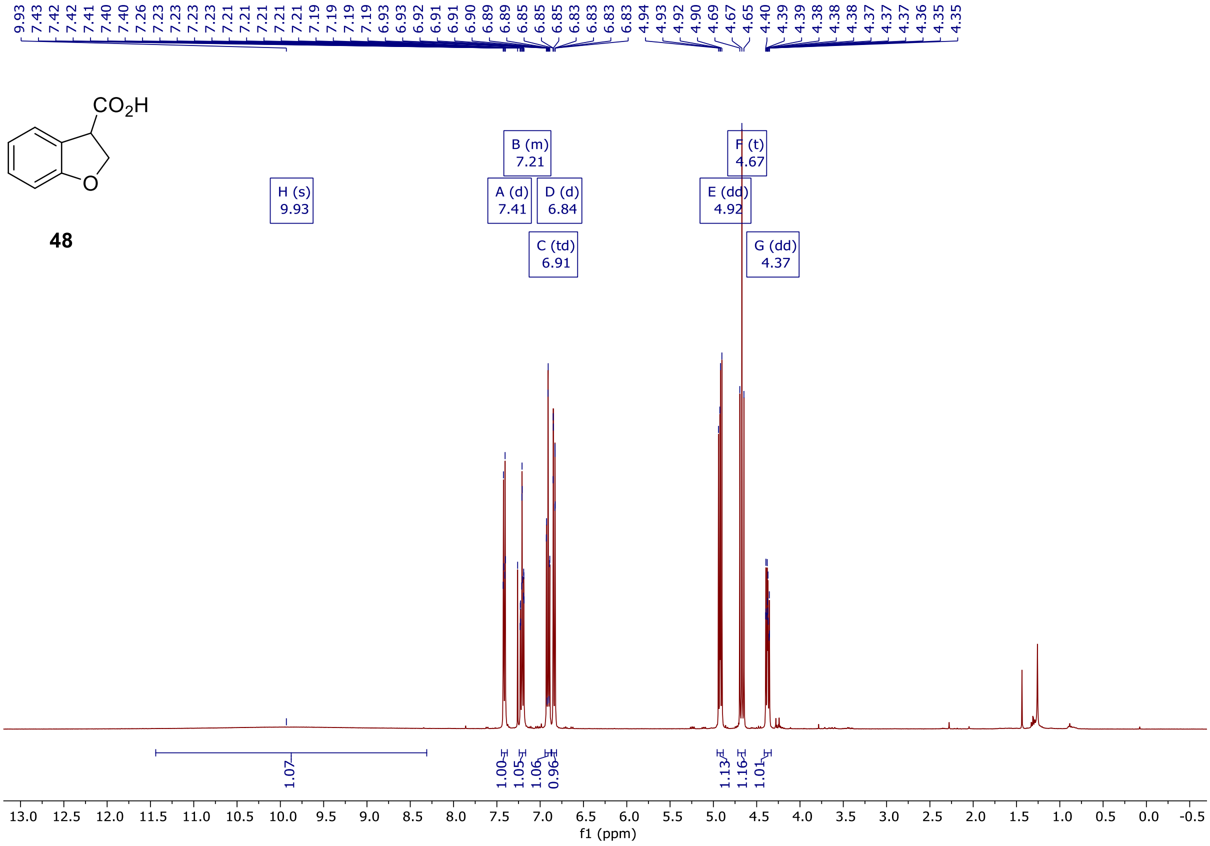

^{13}C NMR (126 MHz, $CDCl_3$):

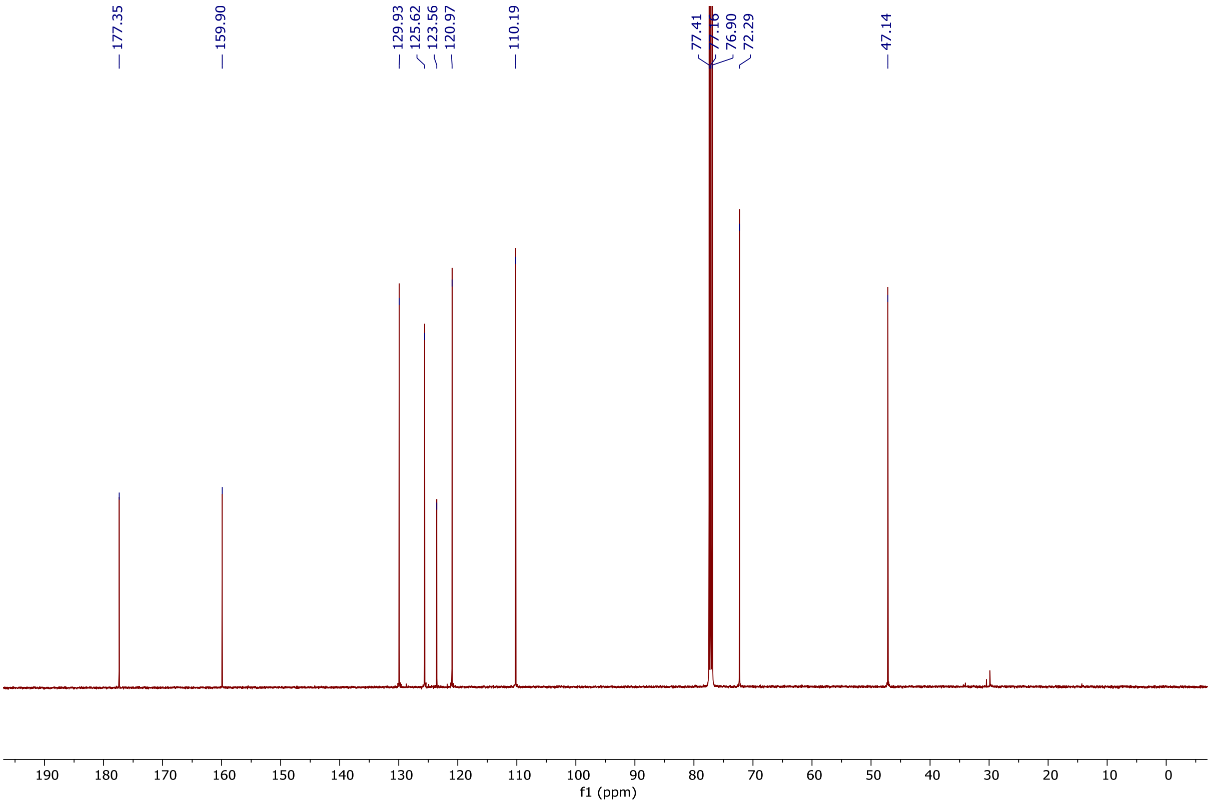

^{1}H NMR (400 MHz, MeOD) 1-(Cyanomethyl)tetrahydro-1H-thiophen-1-ium Bromide (49):

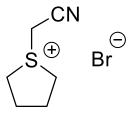

49

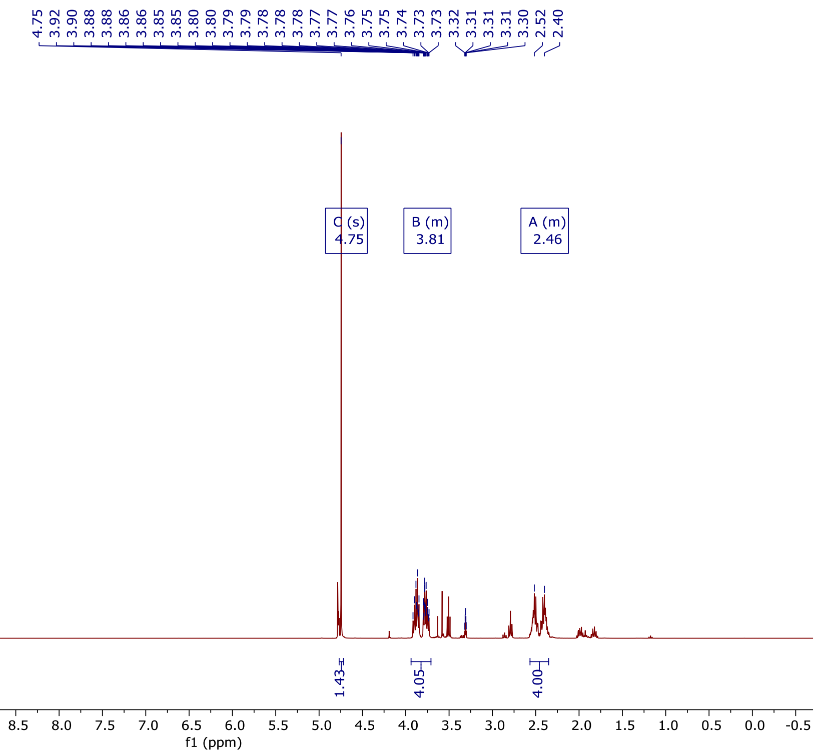

^{13}C NMR (101 MHz, MeOD):

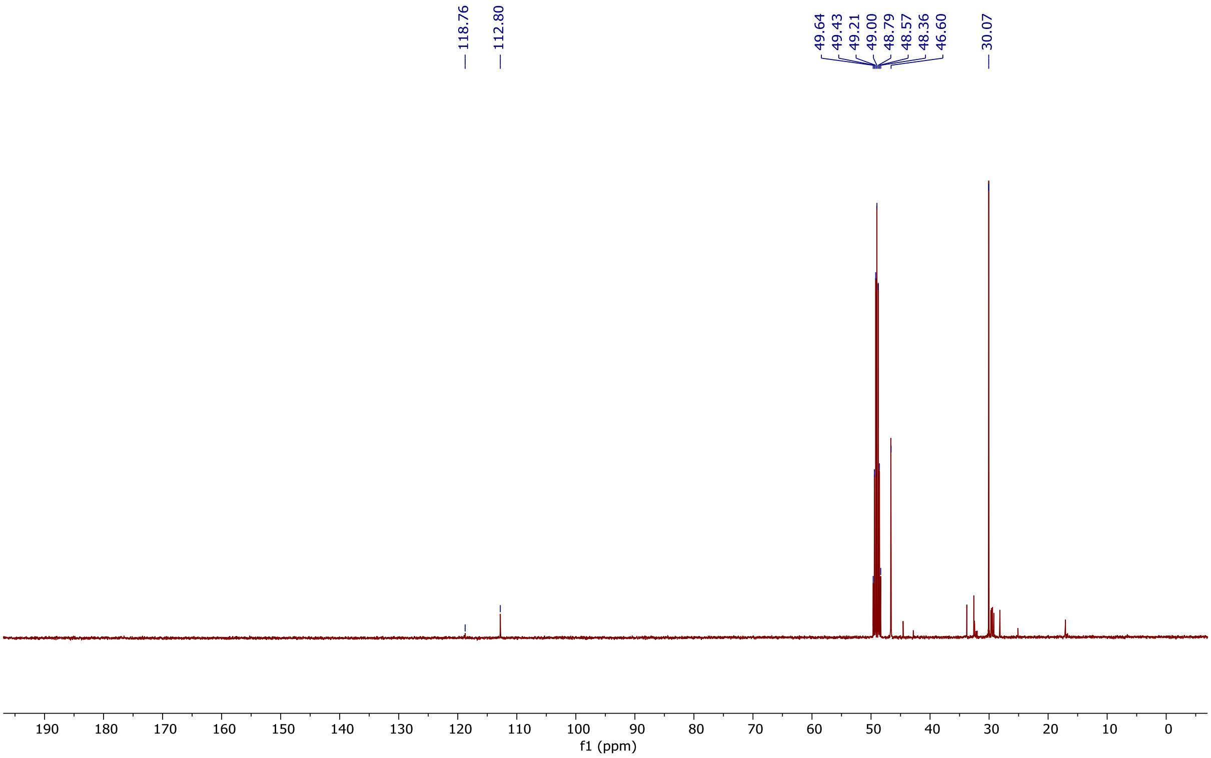

^{1}H NMR (400 MHz, $CDCl_3$) 3-(2,3-Dihydrobenzofuran-3-yl)-3-oxo-2-(tetrahydro-1λ[4]-thiophen-1-ylidene)propanenitrile (50):

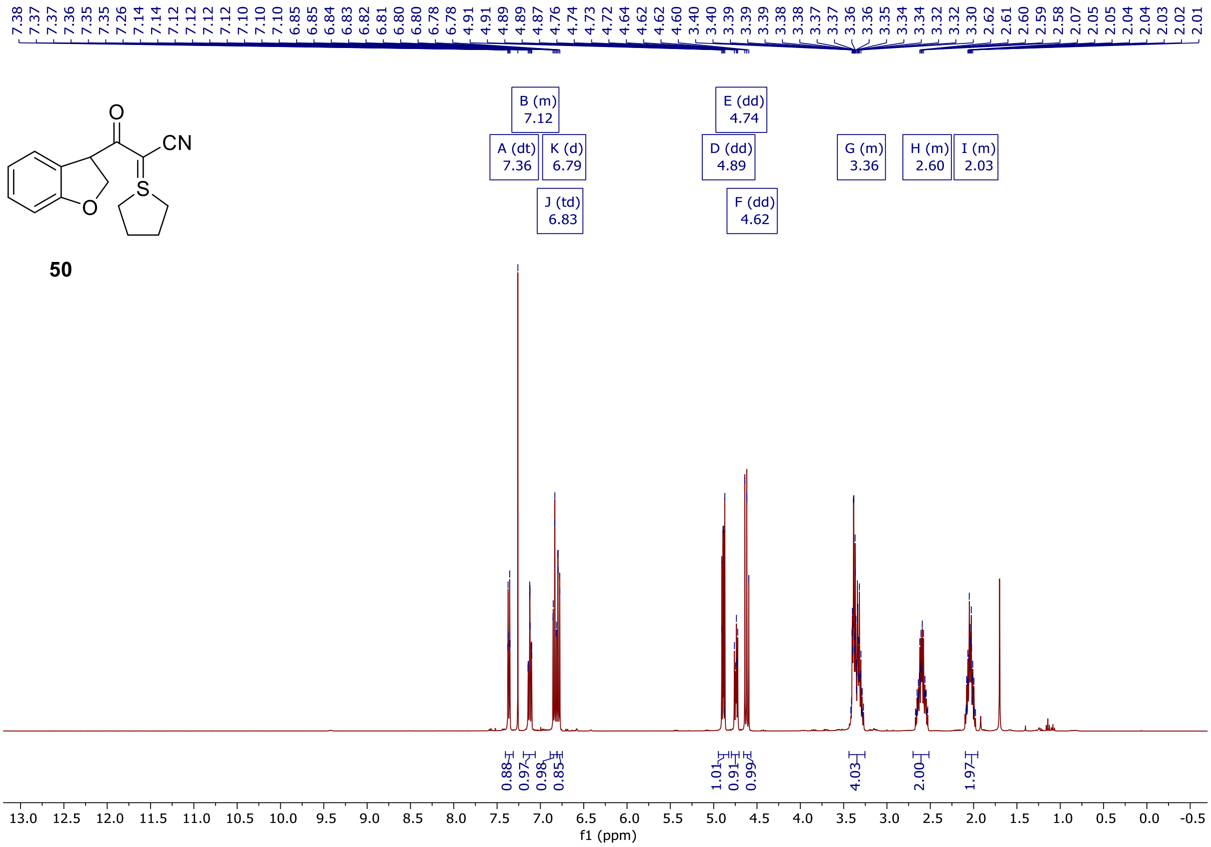

^{13}C NMR (101 MHz, $CDCl_3$):

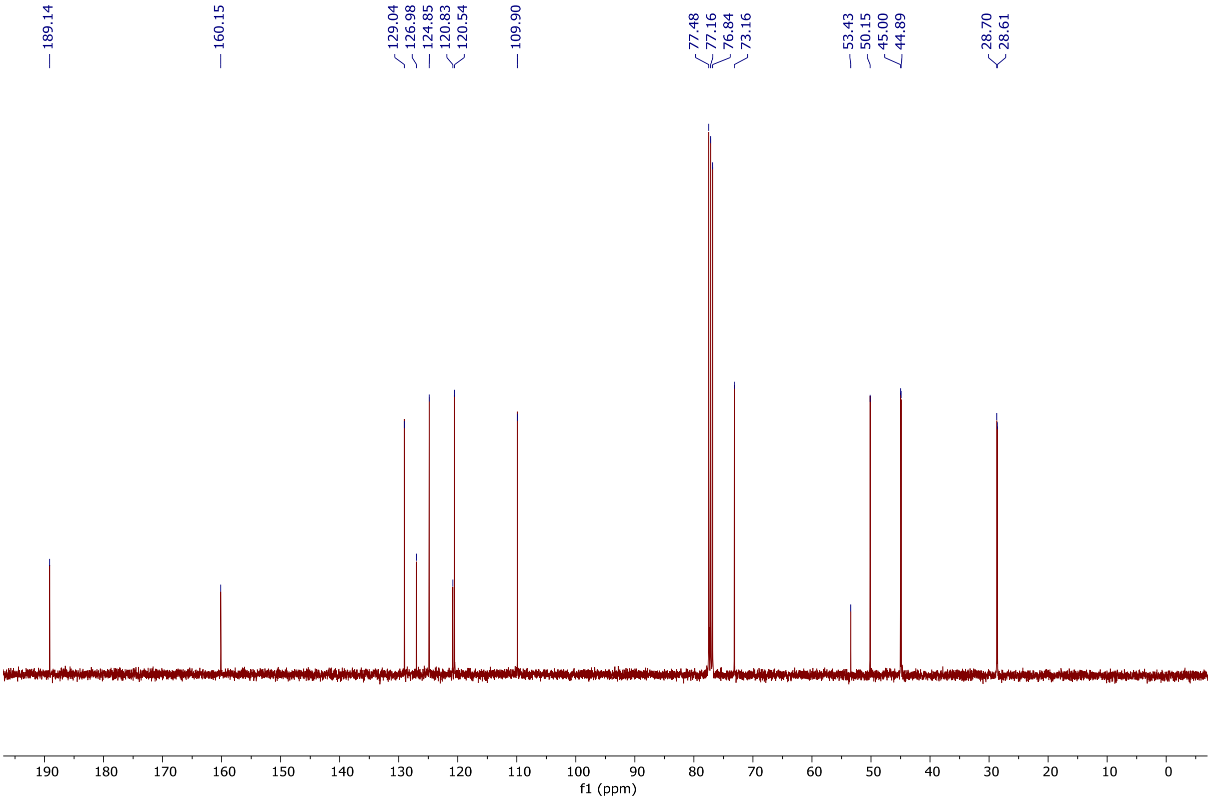

^{1}H NMR (400 MHz, $CDCl_3$) Methyl 3-(3-(2-cyano-2-(tetrahydro-1λ^4-thiophen-1-ylidene)acetyl)-2,3-dihydrobenzofuran-3-yl)propiolate (51)

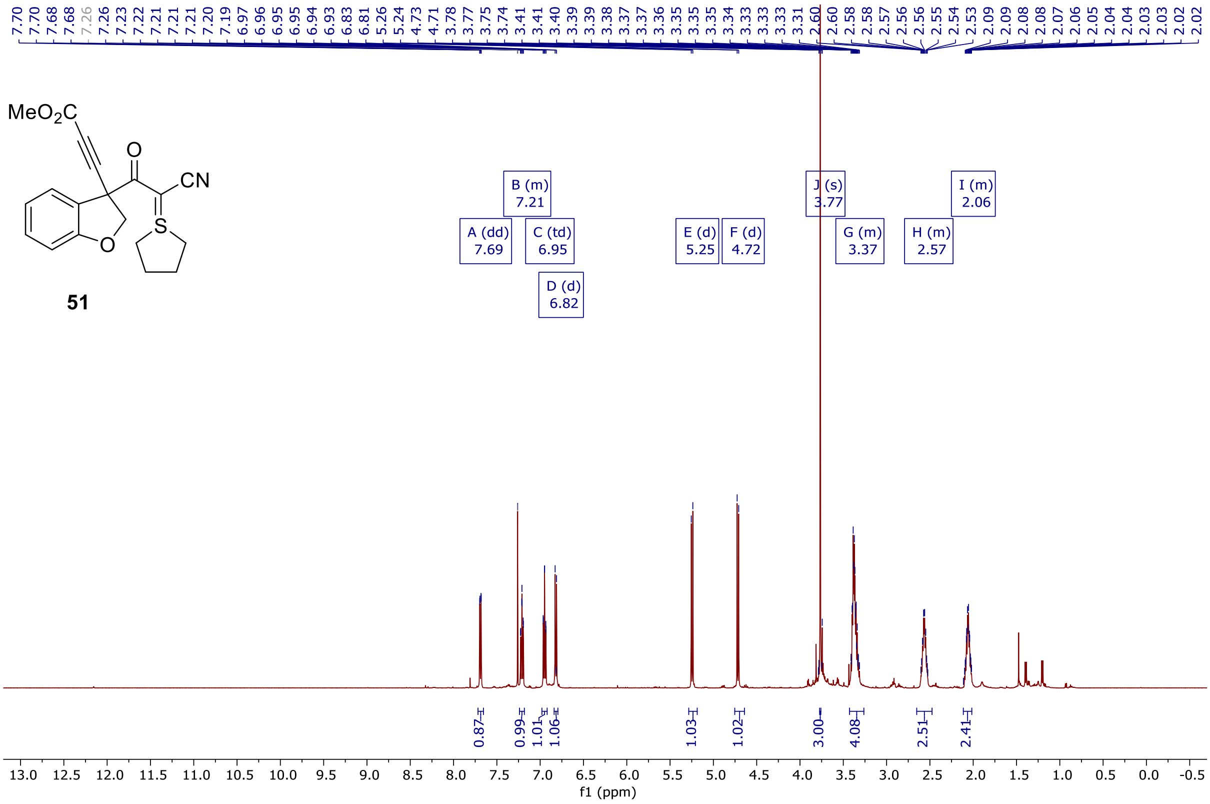

^{13}C NMR (101 MHz, $CDCl_3$):

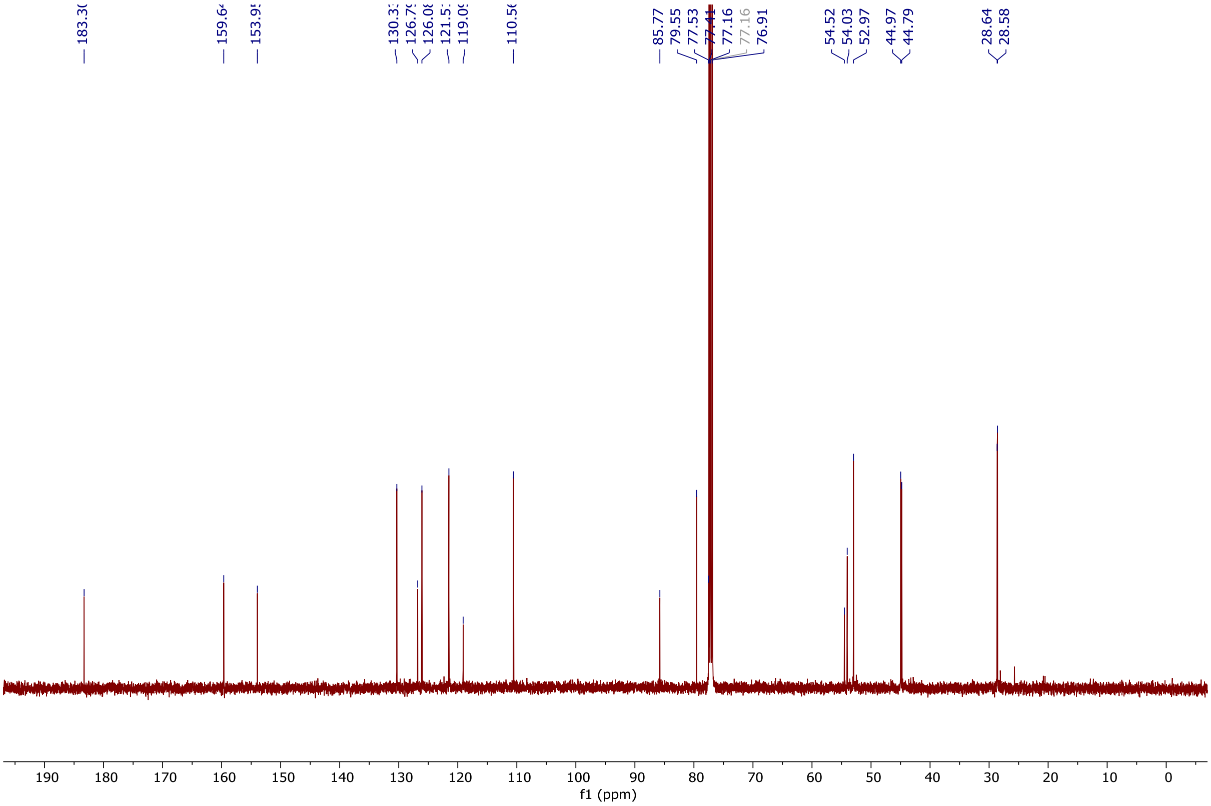

^{1}H NMR (400 MHz, CDCl$_3$) Methyl (*Z*)-3-(3-(2-cyano-2-(tetrahydro-1λ^4-thiophen-1-ylidene)acetyl)-2,3-dihydrobenzofuran-3-yl)-5-methylhex-2-enoate (52):

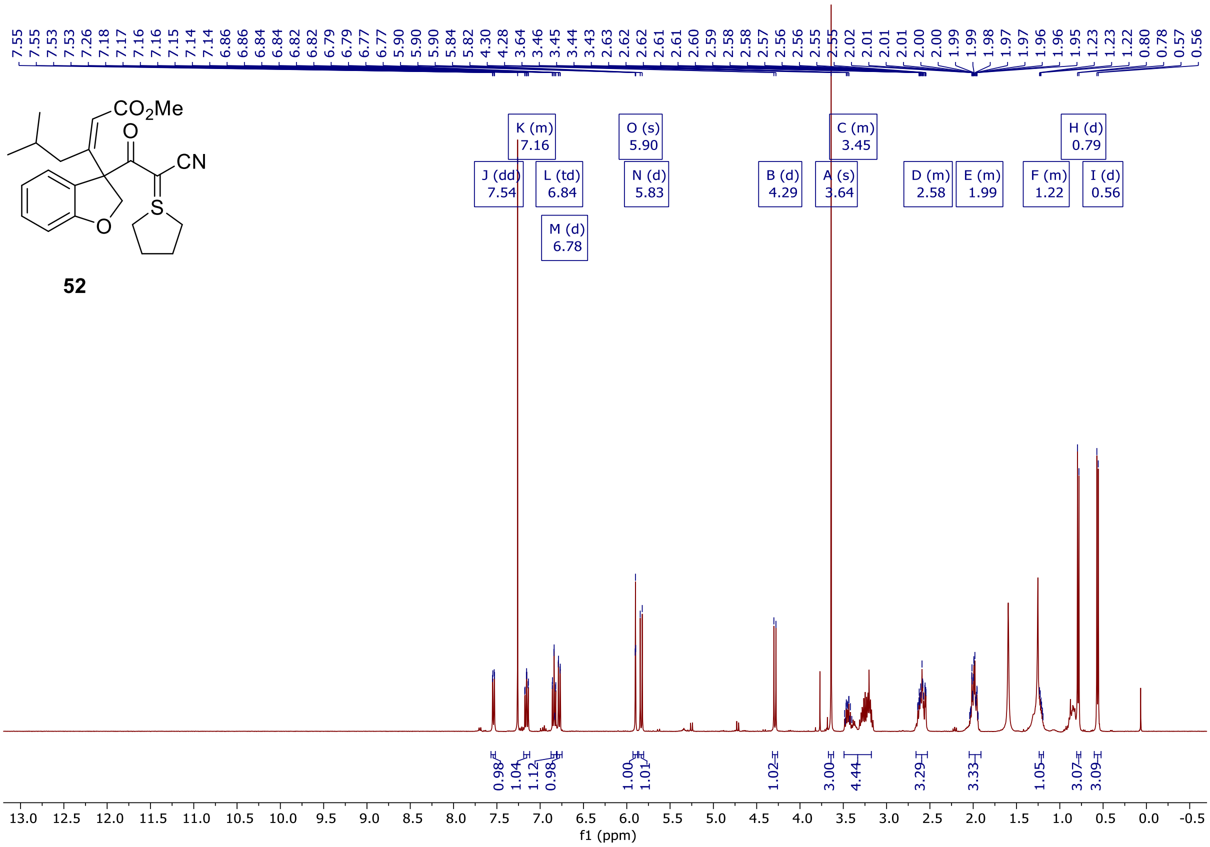

52

^{13}C NMR (126 MHz, CDCl$_3$):

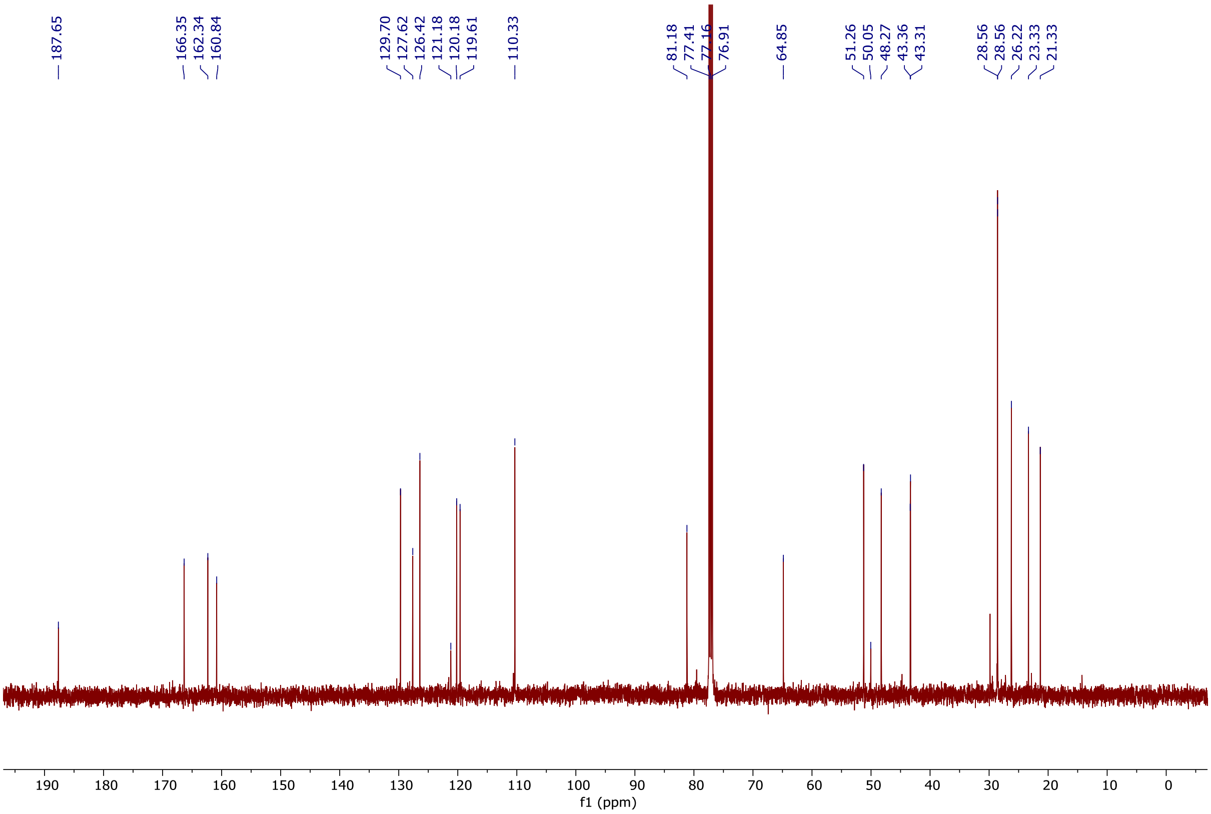

^{1}H NMR (500 MHz, CDCl$_3$) Methyl (*Z*)-3-(3-(2-methoxy-2-oxoacetyl)-2,3-dihydrobenzofuran-3-yl)-5-methylhex-2-enoate (53)

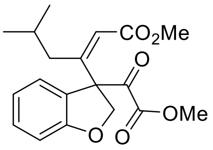

53

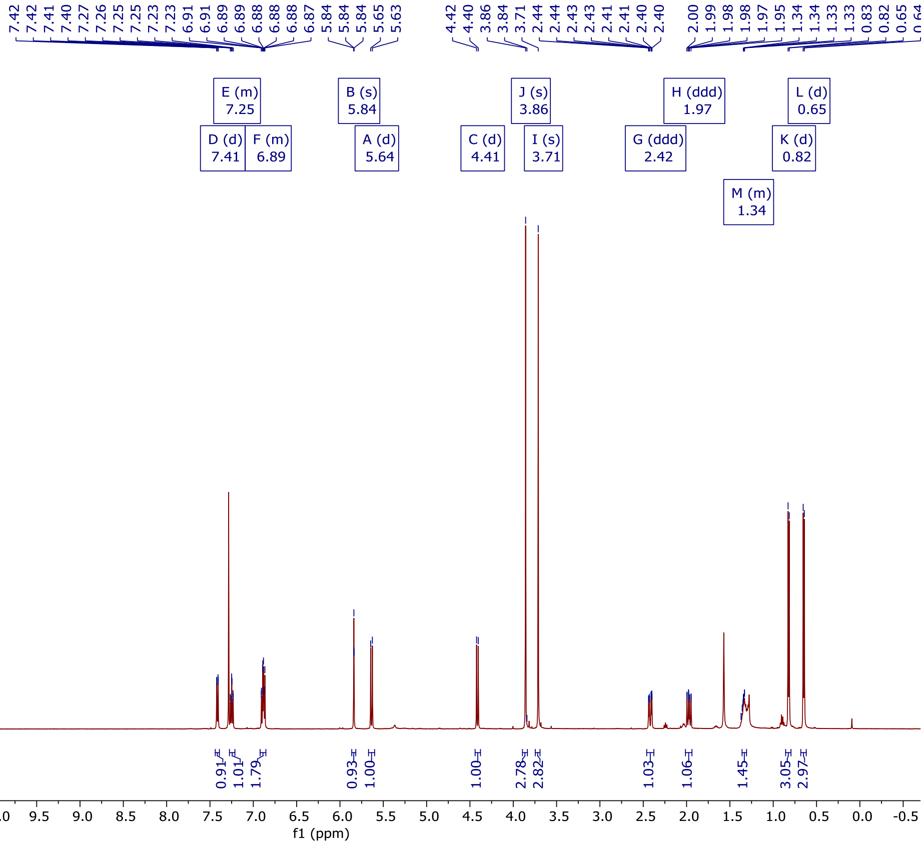

^{13}C NMR (126 MHz, CDCl$_3$):

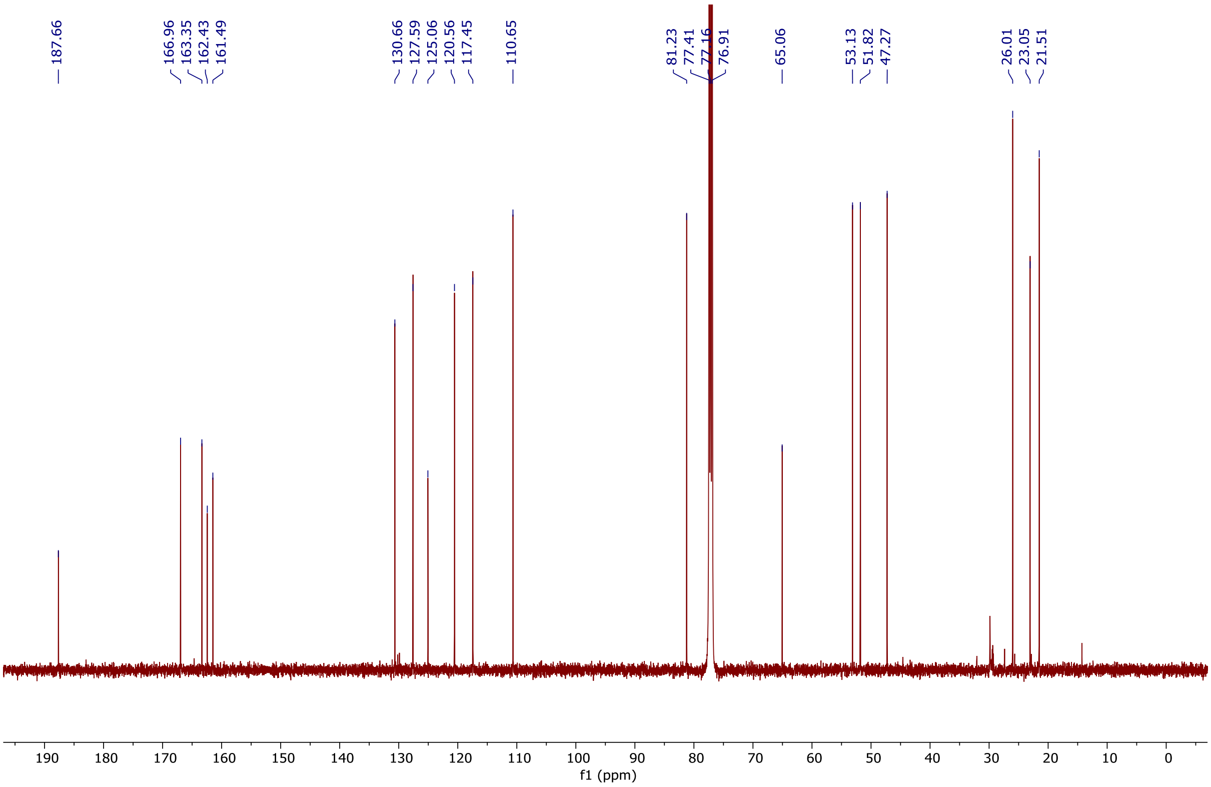

^{1}H NMR (400 MHz, CDCl$_3$) Benzo[*d*][1,3]dioxole-4-carbaldehyde (55):

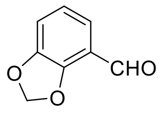

55

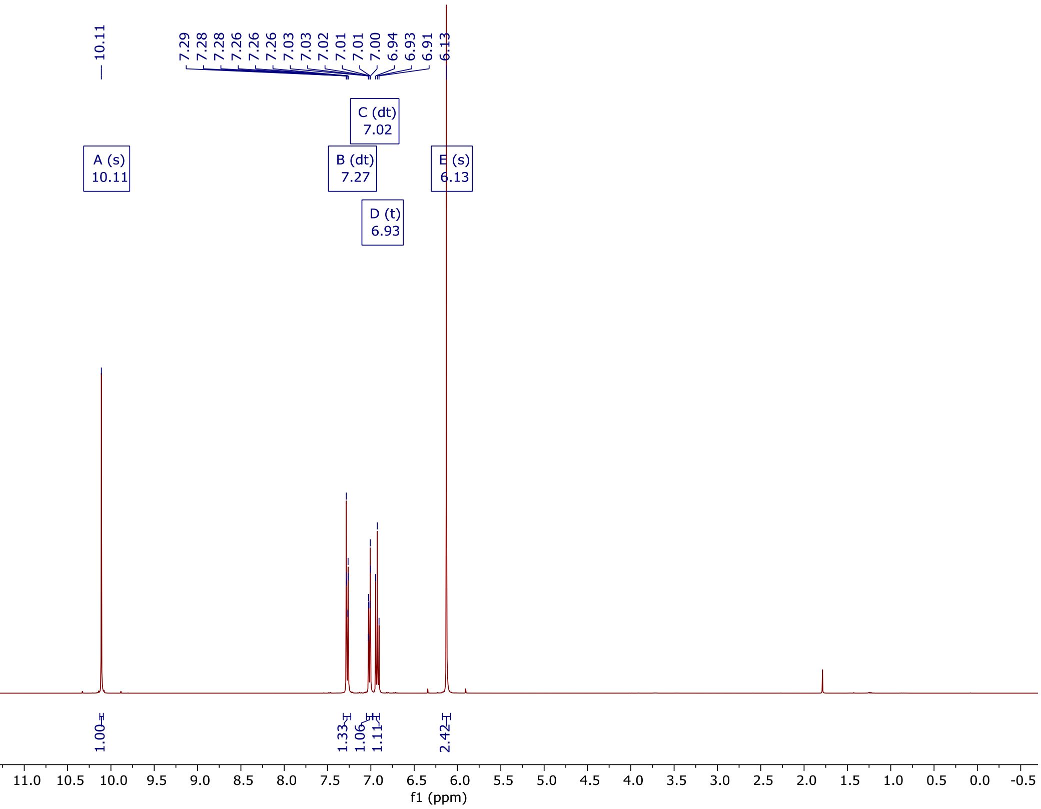

^{13}C NMR (101 MHz, CDCl$_3$):

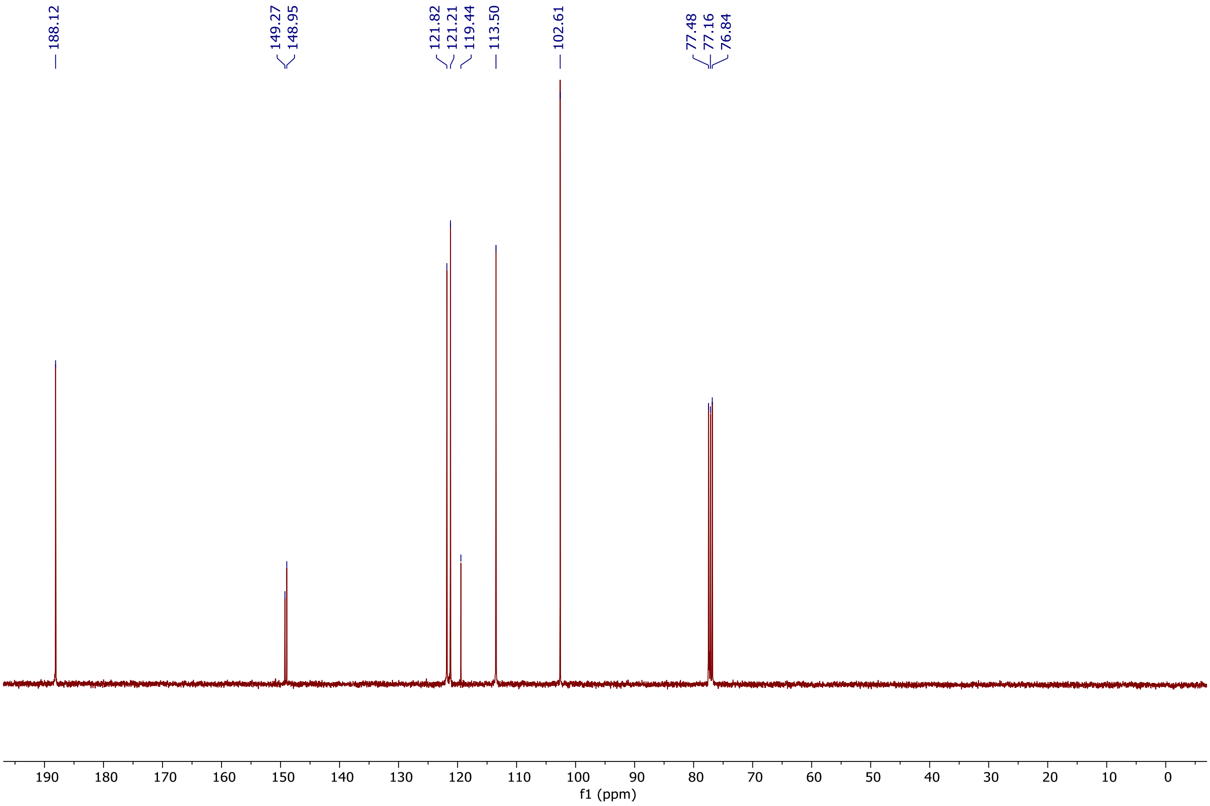

^{1}H NMR (400 MHz, $CDCl_3$) Benzo[*d*][1,3]dioxol-4-ol (56):

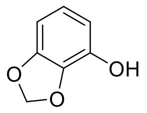

56

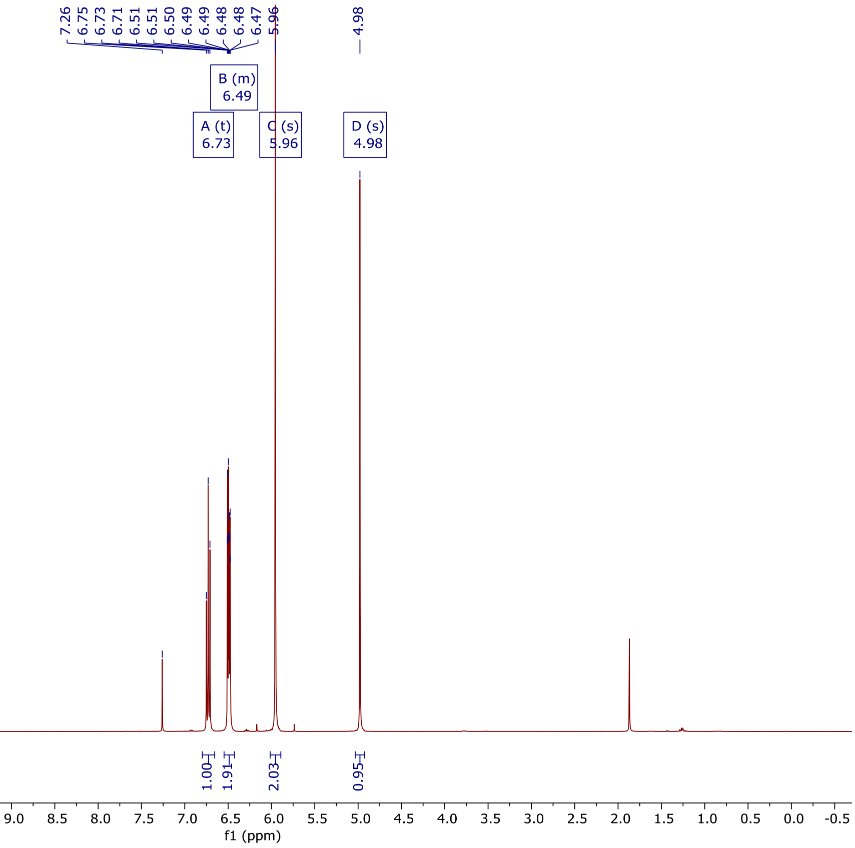

^{13}C NMR (101 MHz, $CDCl_3$):

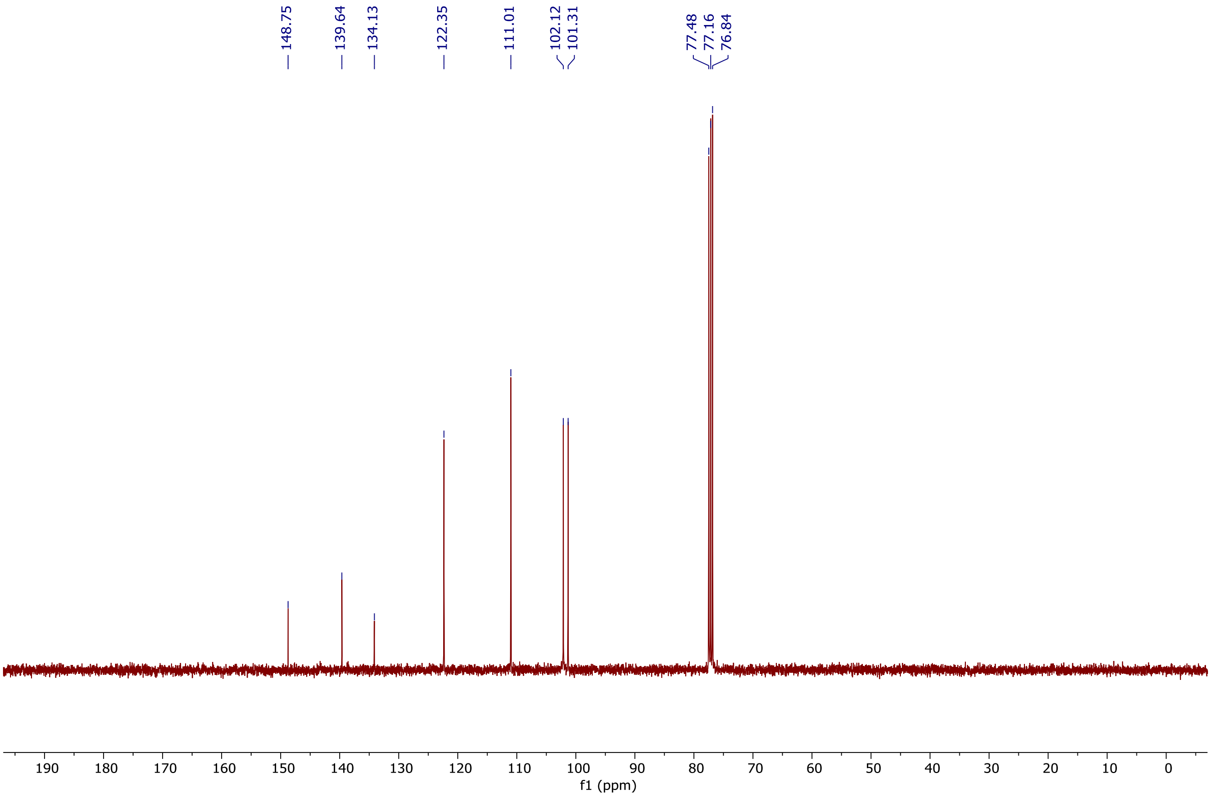

^{1}H NMR (400 MHz, $CDCl_3$) 4-Hydroxybenzo[*d*][1,3]dioxole-5-carbaldehyde (57):

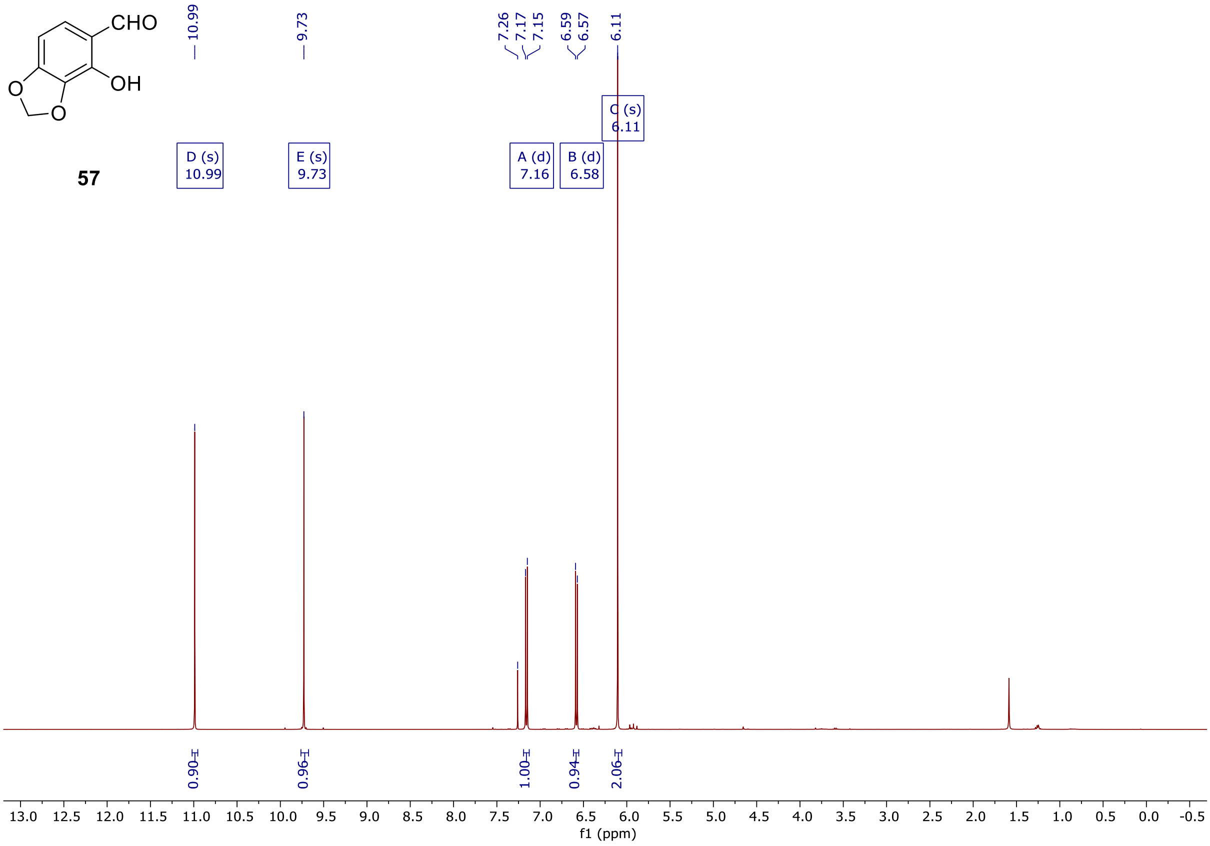

^{13}C NMR (101 MHz, $CDCl_3$):

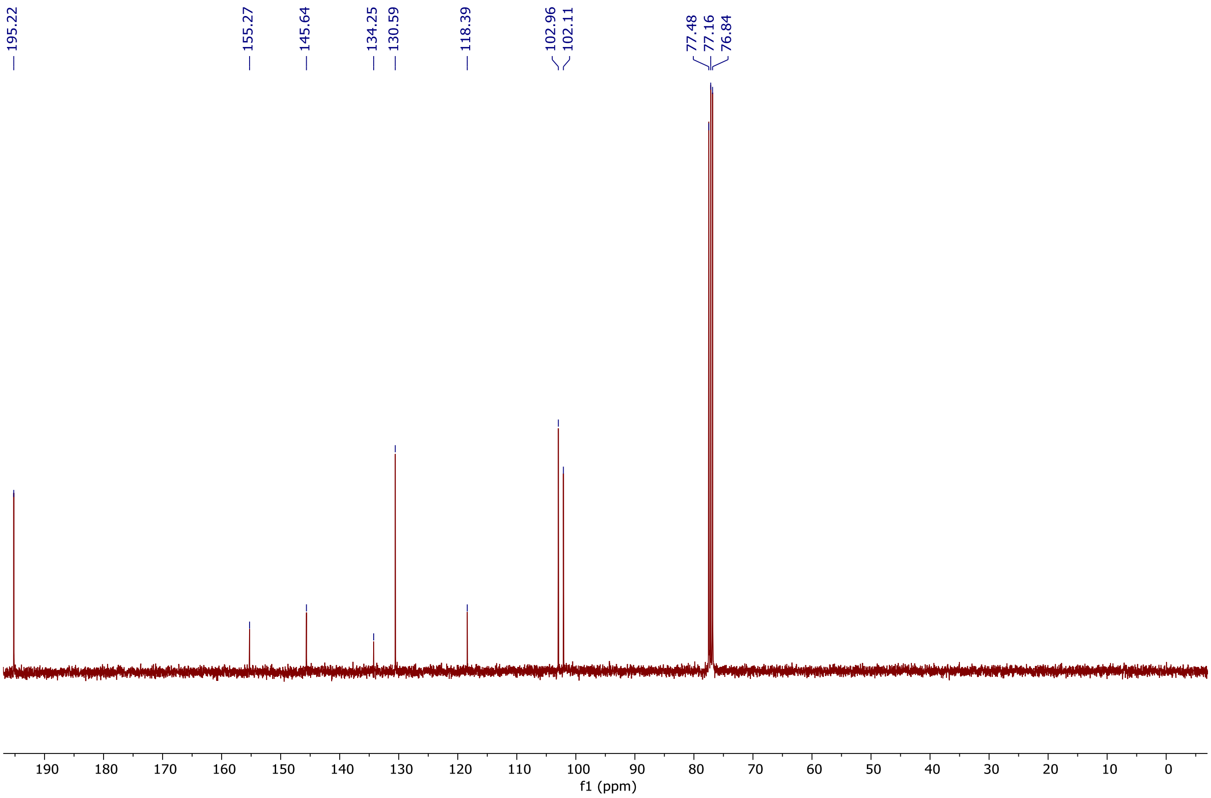

^{1}H NMR (400 MHz, $CDCl_3$) Ethyl [1,3]dioxolo[4,5-*g*]benzofuran-6-carboxylate (58):

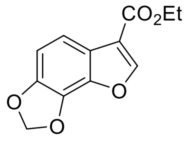

58

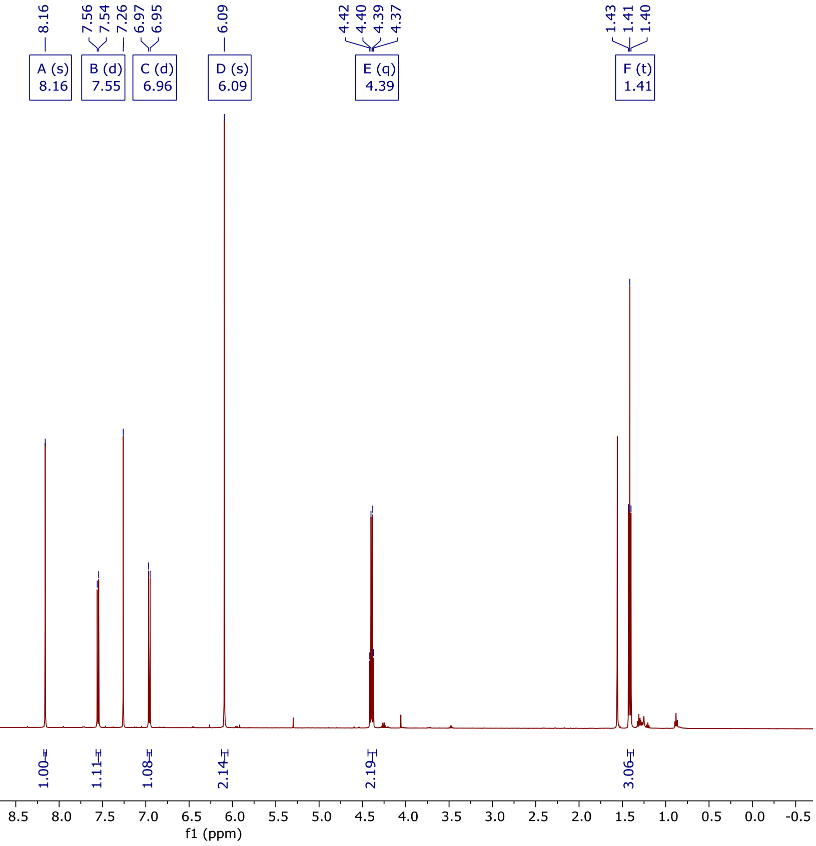

^{13}C NMR (101 MHz, $CDCl_3$):

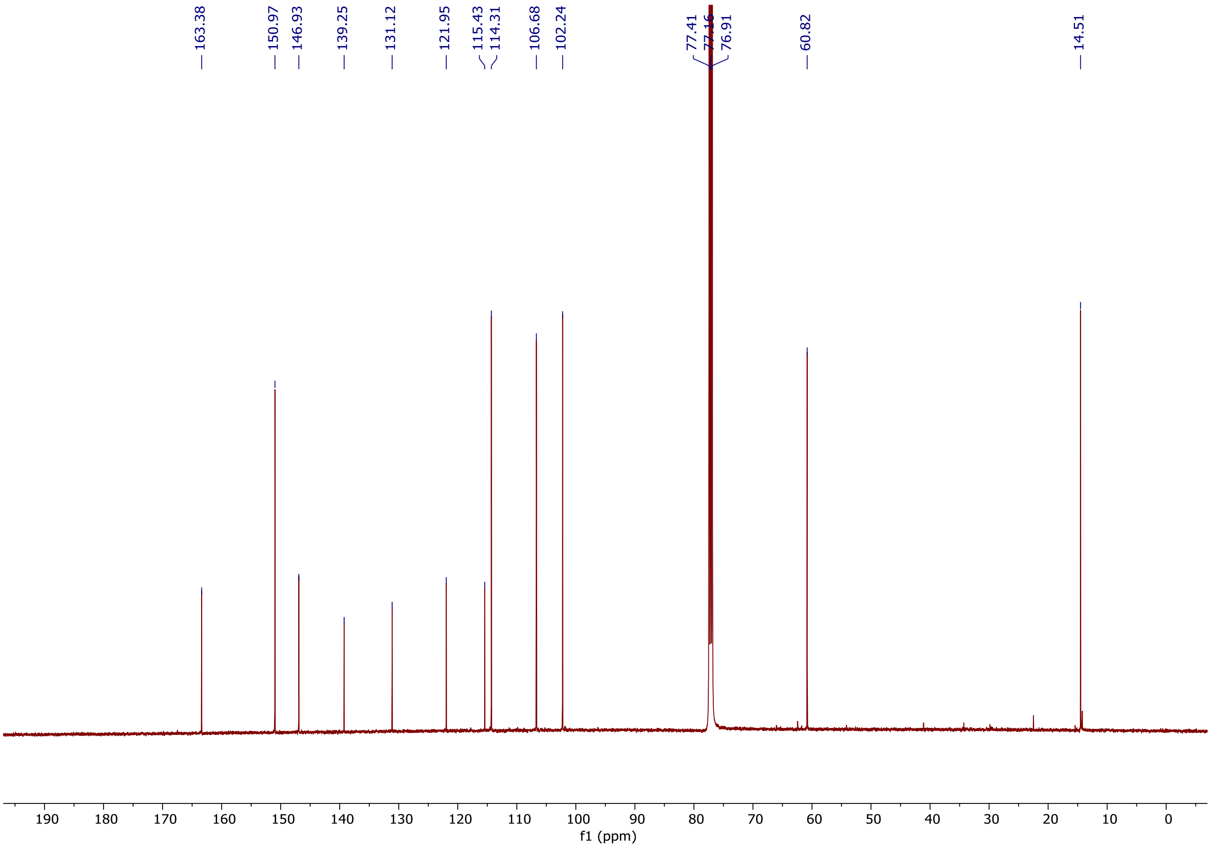

^{1}H NMR (400 MHz, $CDCl_3$) Methyl 6,7-dihydro-[1,3]dioxolo[4,5-*g*]benzofuran-6-carboxylate (59):

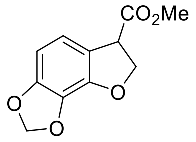

59

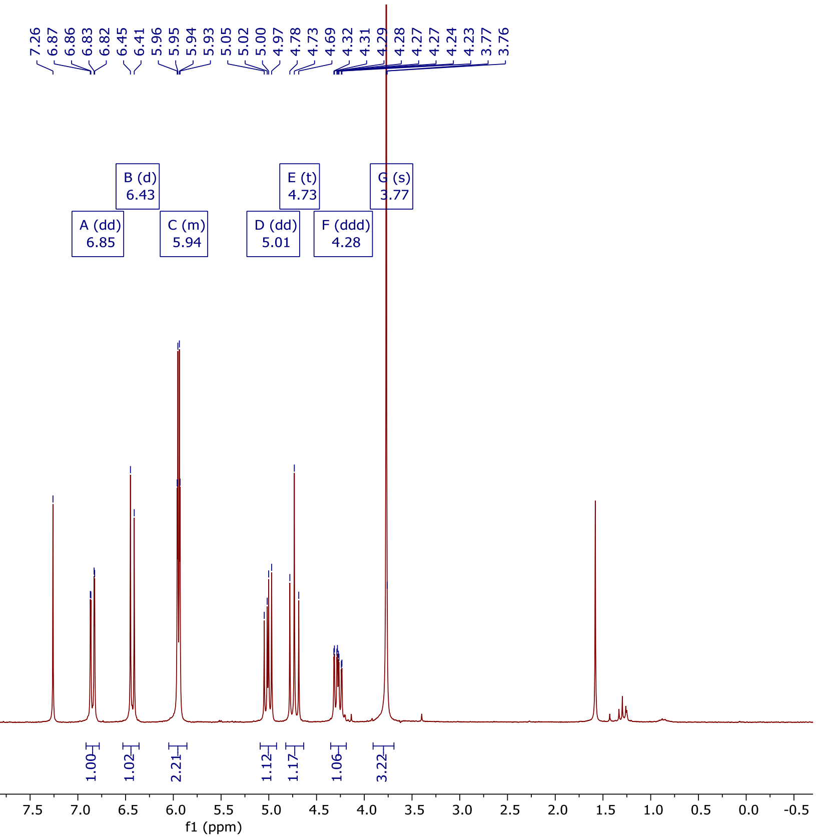

^{13}C NMR (101 MHz, $CDCl_3$):

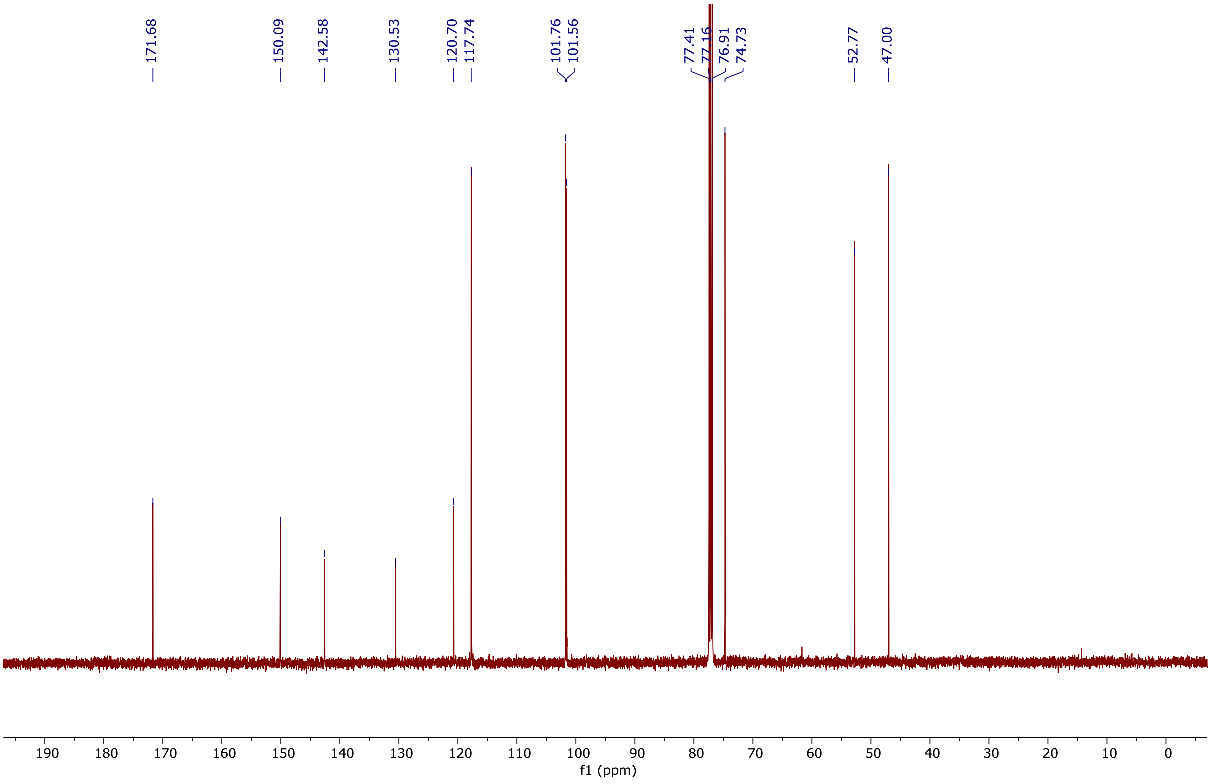

^{1}H NMR (400 MHz, $CDCl_3$) Ethyl 4-(trimethylsilyl)benzofuran-3-carboxylate (61):

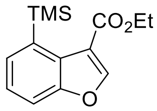

61

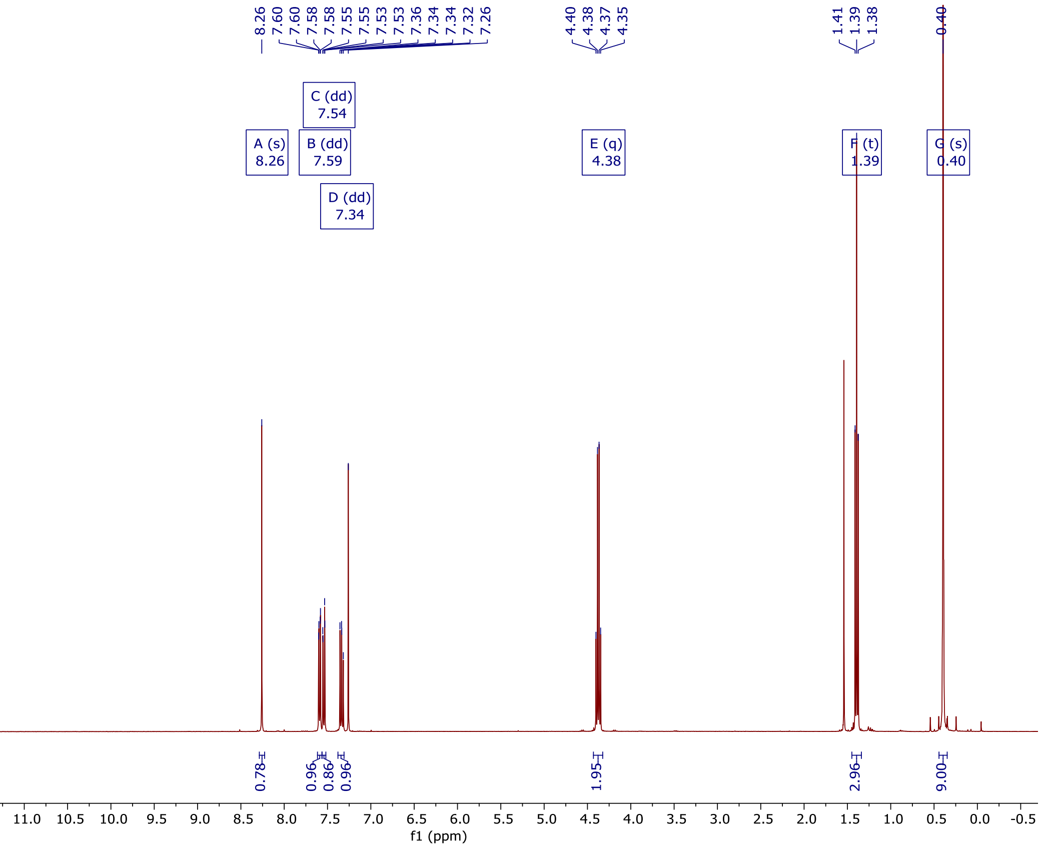

^{13}C NMR (101 MHz, $CDCl_3$):

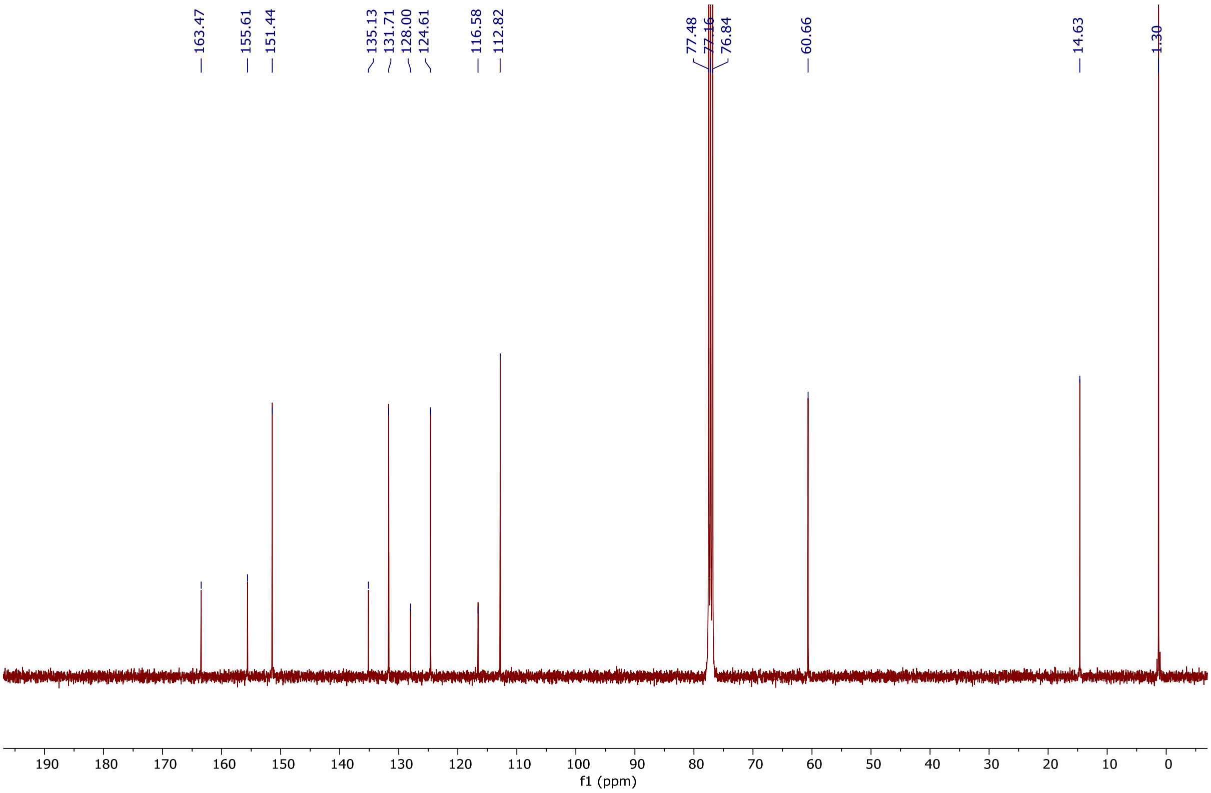

^{1}H NMR (400 MHz, CDCl₃) Ethyl 4-(trimethylsilyl)-2,3-dihydrobenzofuran-3-carboxylate (62):

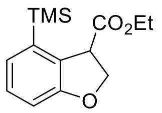

62

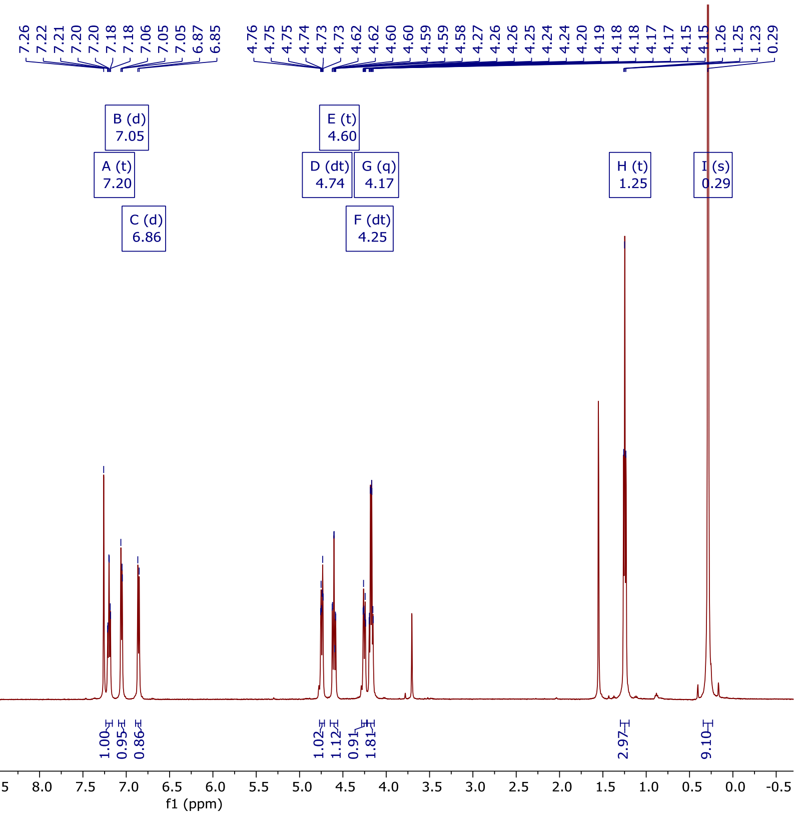

^{13}C NMR (126 MHz, CDCl₃):

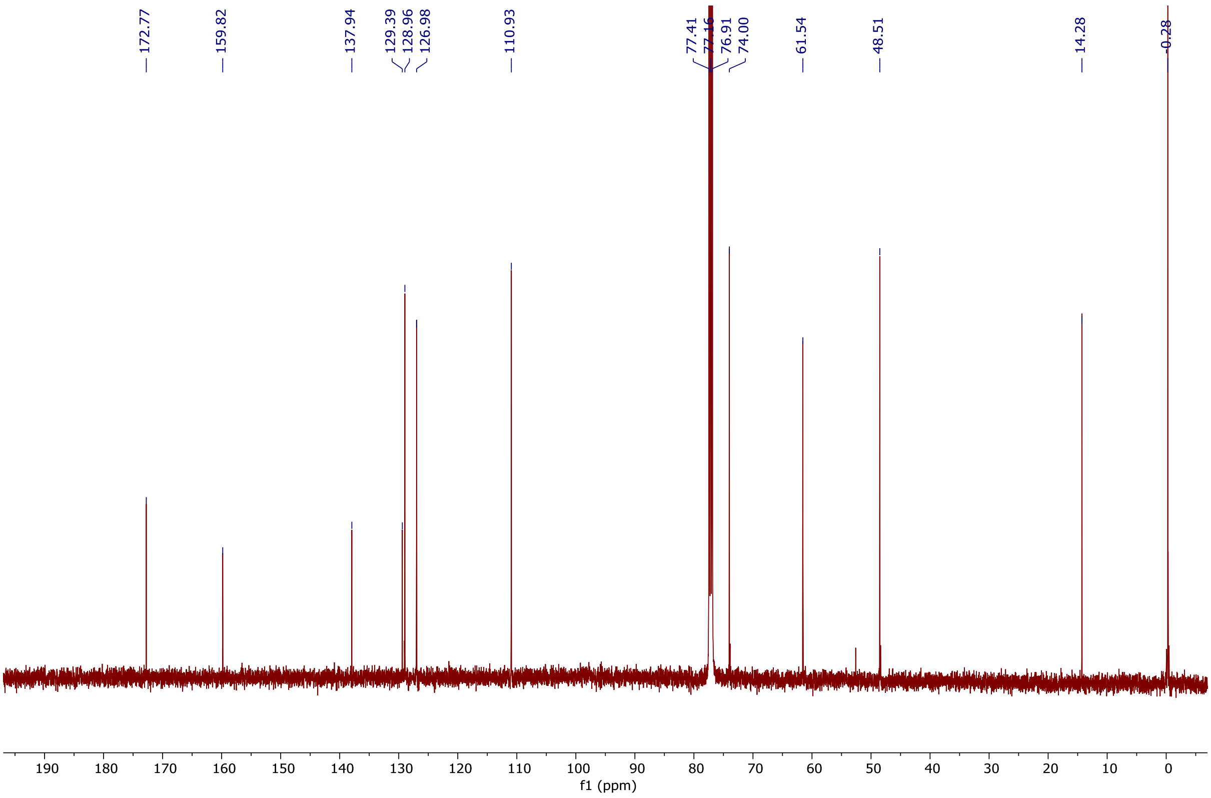

^{1}H NMR (400 MHz, $CDCl_3$) 2-(Trimethylsilyl)ethyl 2,3-dihydrobenzofuran-3-carboxylate (63):

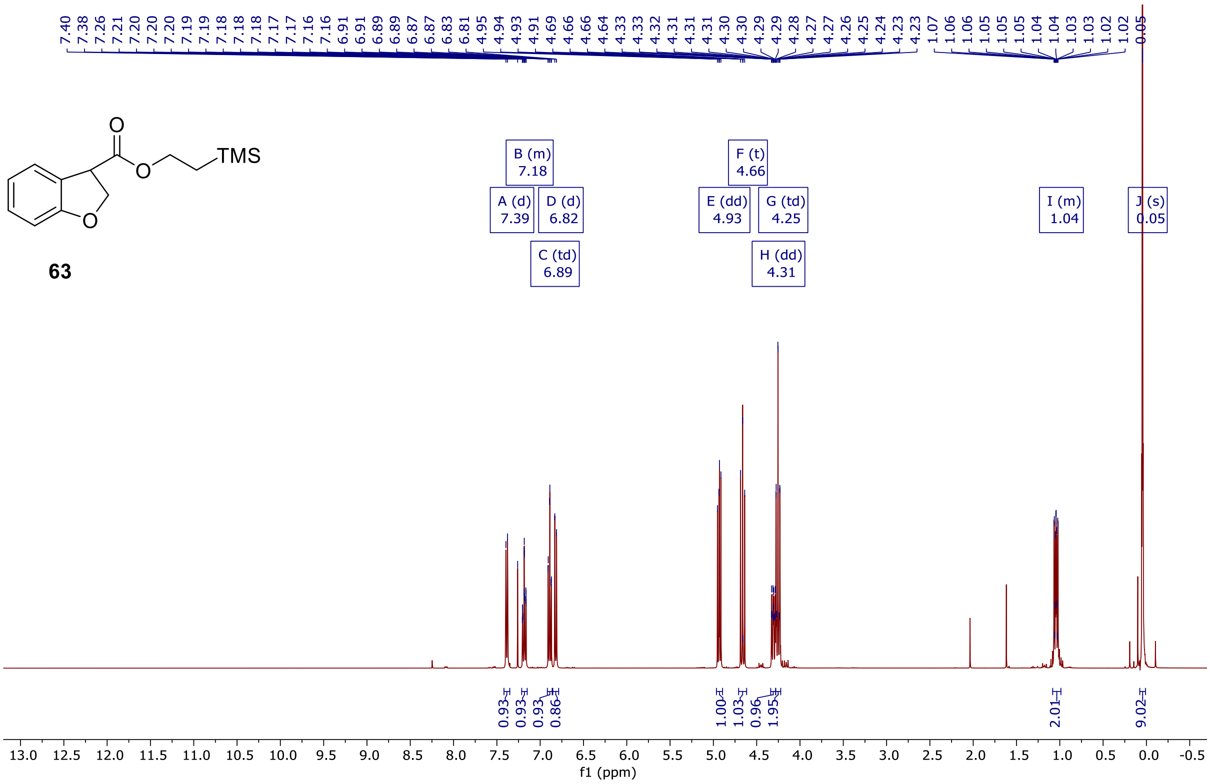

63

^{13}C NMR (101 MHz, $CDCl_3$):

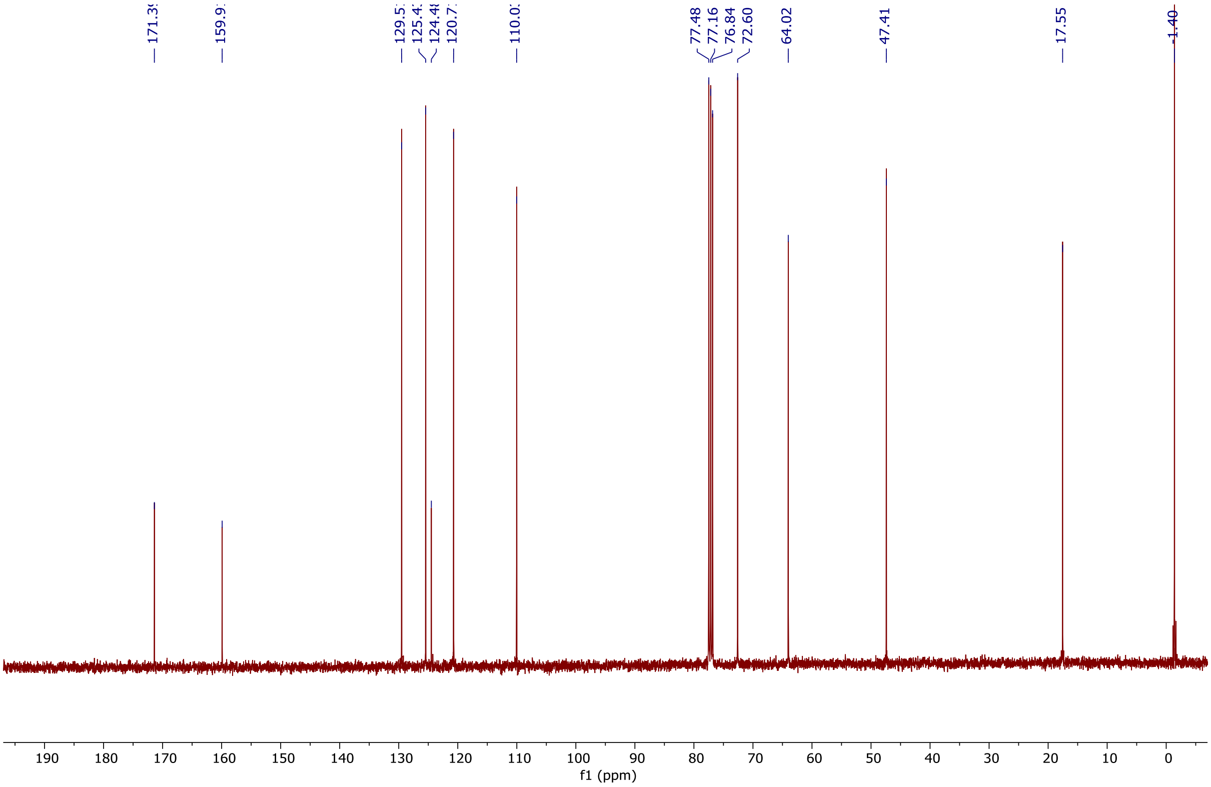

^{1}H NMR (400 MHz, $CDCl_3$) *N*-Methoxy-*N*-methyl-2,3-dihydrobenzofuran-3-carboxamide (64):

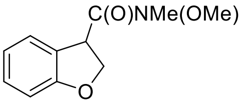

64

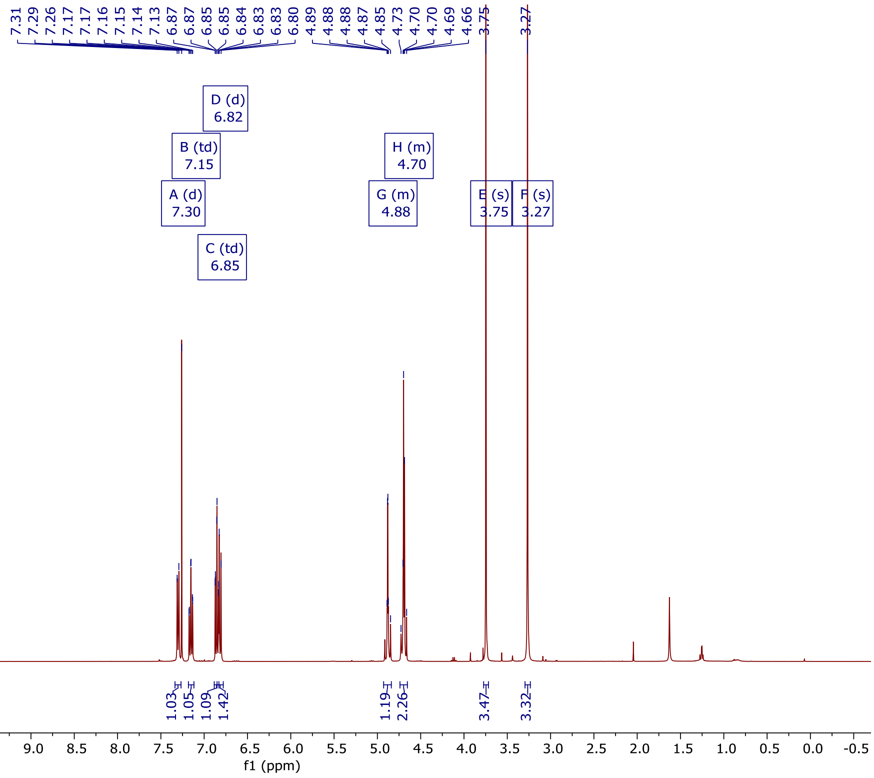

^{13}C NMR (101 MHz, $CDCl_3$):

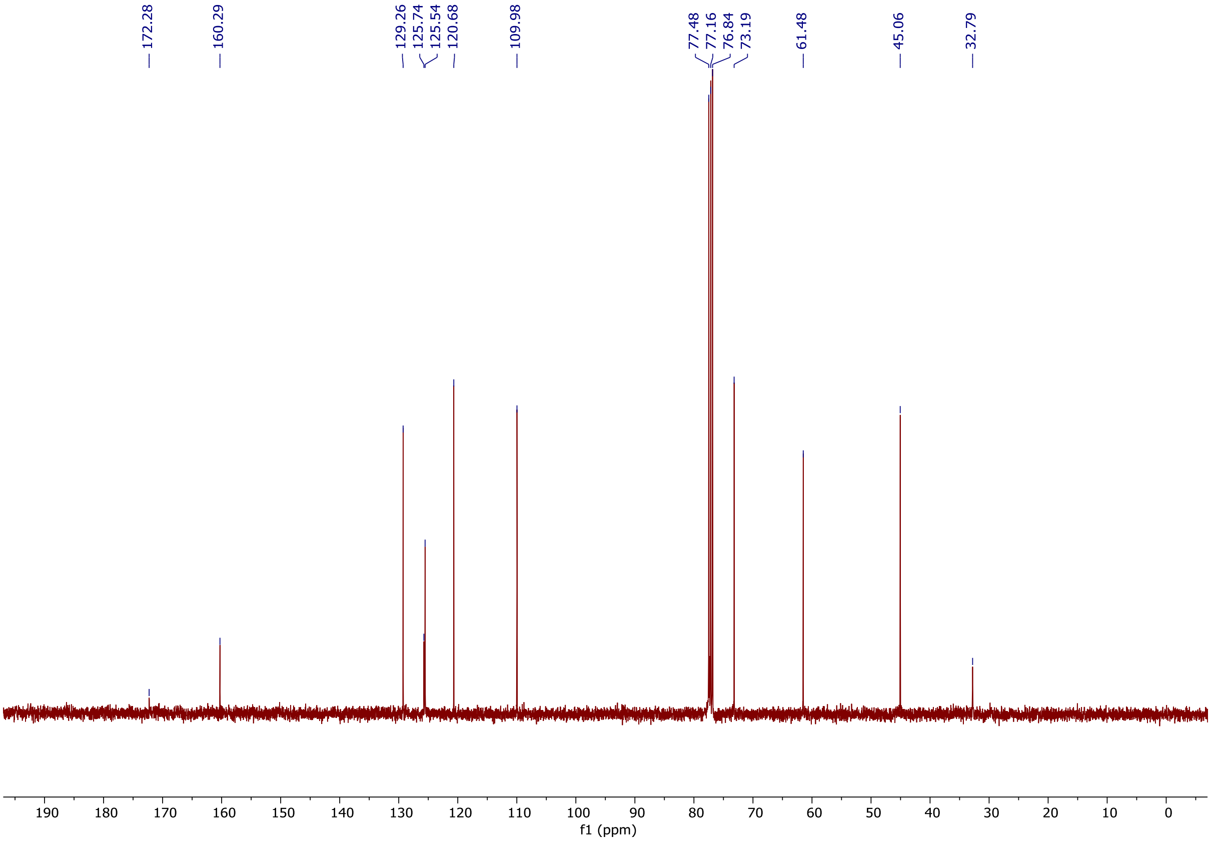

^{1}H NMR (400 MHz, $CDCl_3$) *S*-Ethyl 2,3-dihydrobenzofuran-3-carbothioate (65):

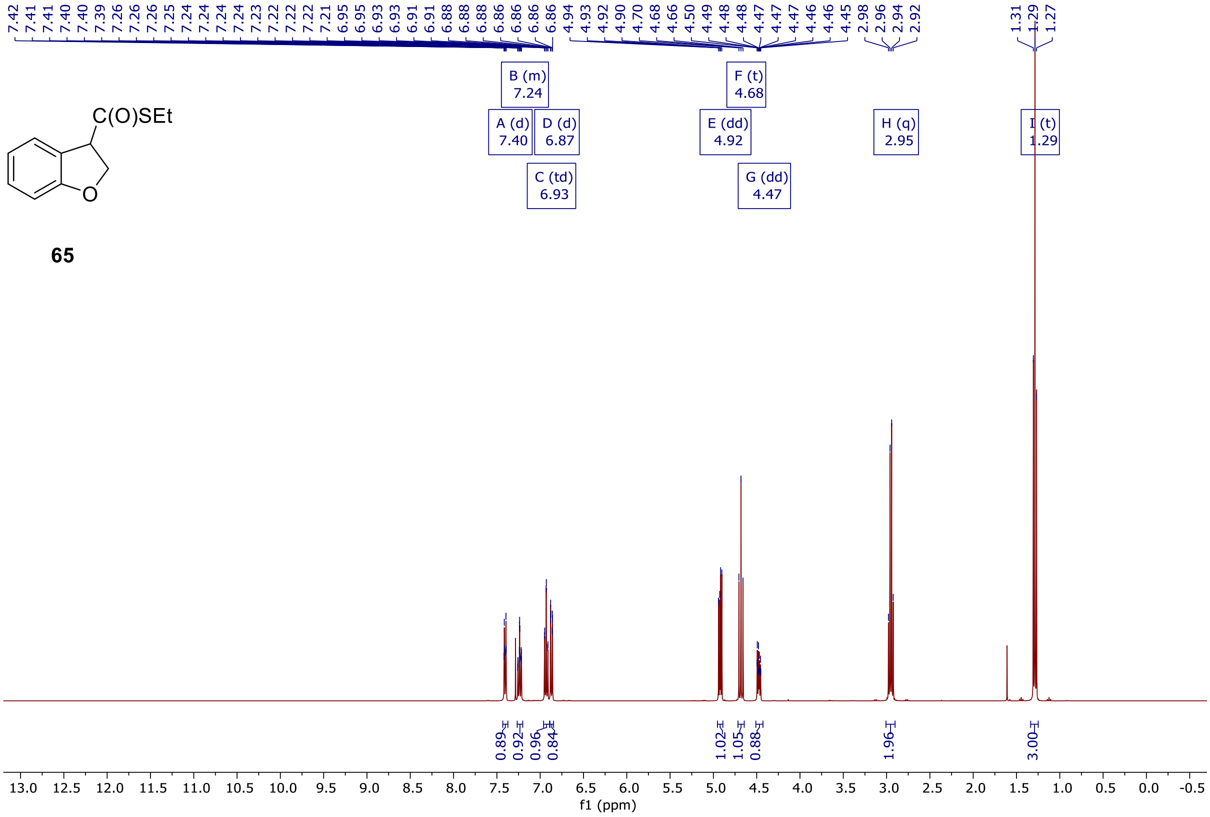

^{13}C NMR (101 MHz, $CDCl_3$):

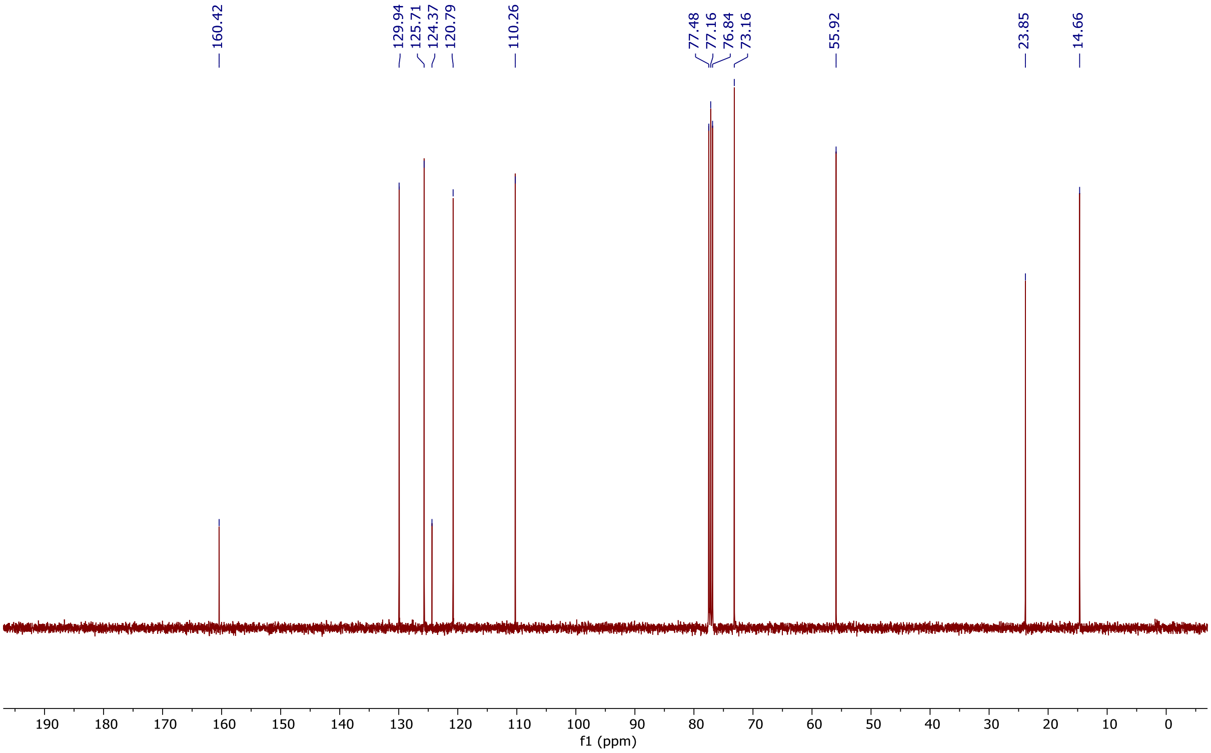

^{1}H NMR (400 MHz, CDCl$_3$) 2-Methyl-2-((tosyloxy)methyl)propane-1,3-diyl bis(4-methylbenzenesulfonate) (67):

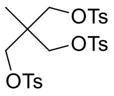

67

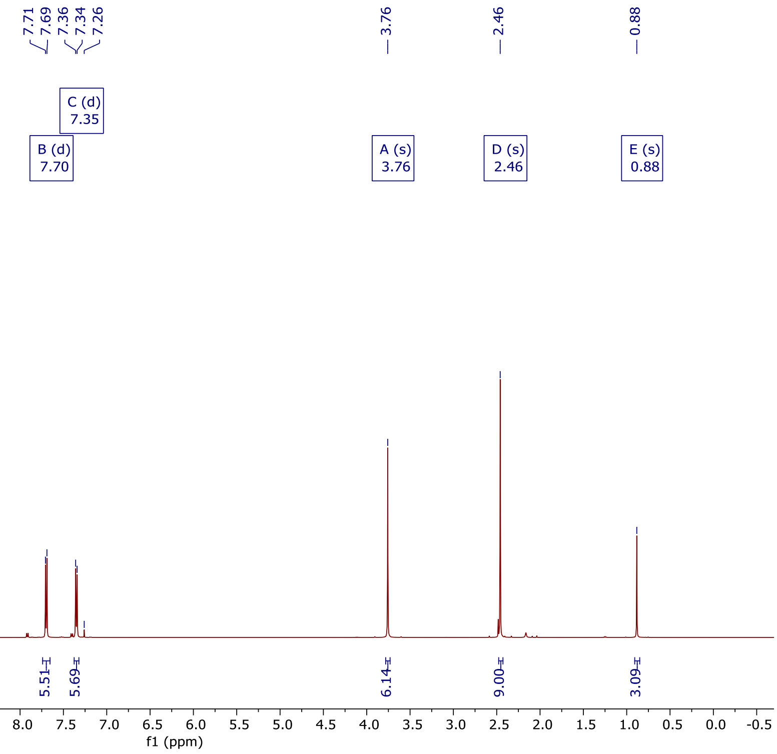

^{13}C NMR (101 MHz, CDCl$_3$):

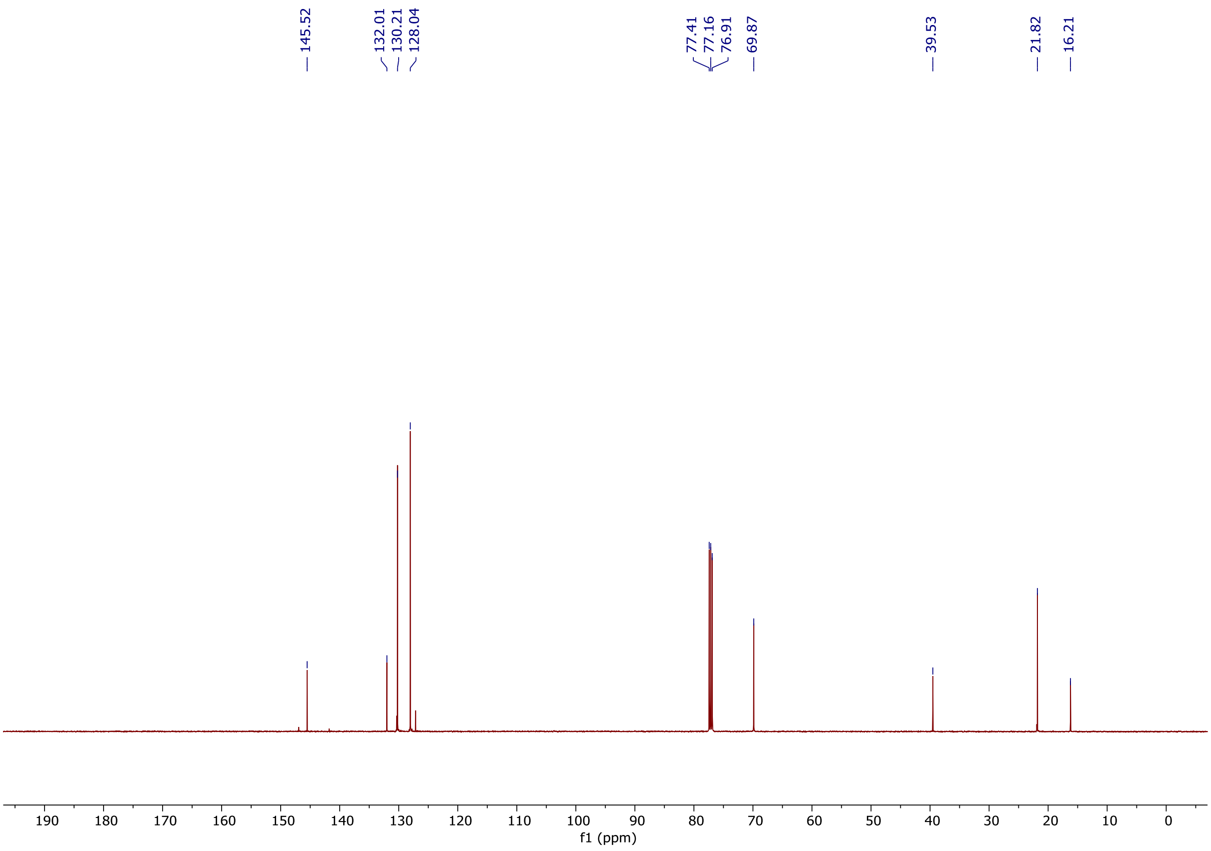

^{1}H NMR (400 MHz, $CDCl_3$) 2-(Mercaptomethyl)-2-methylpropane-1,3-dithiol (68):

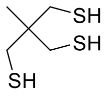

68

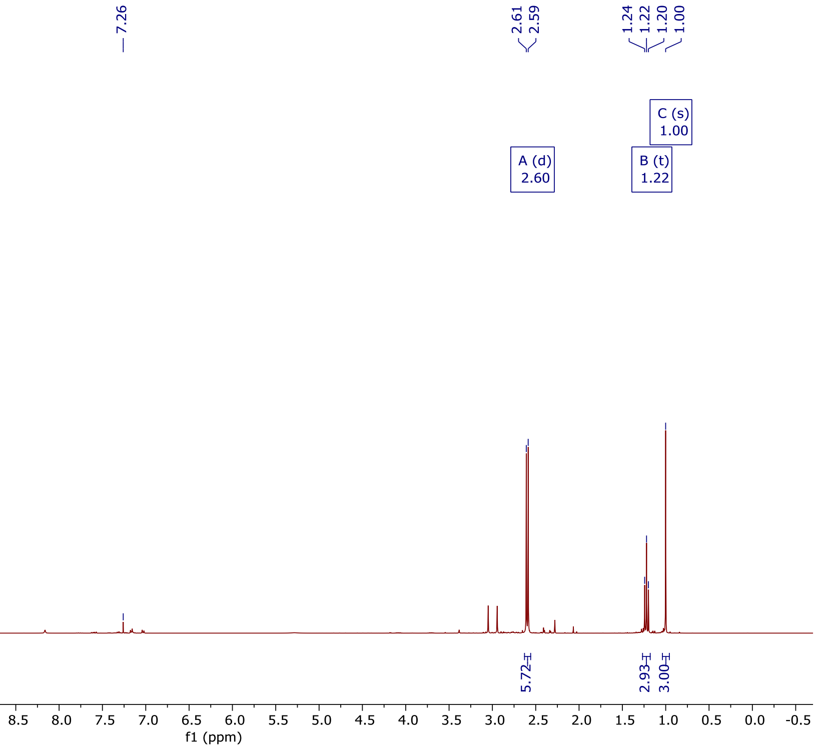

^{13}C NMR (101 MHz, $CDCl_3$):

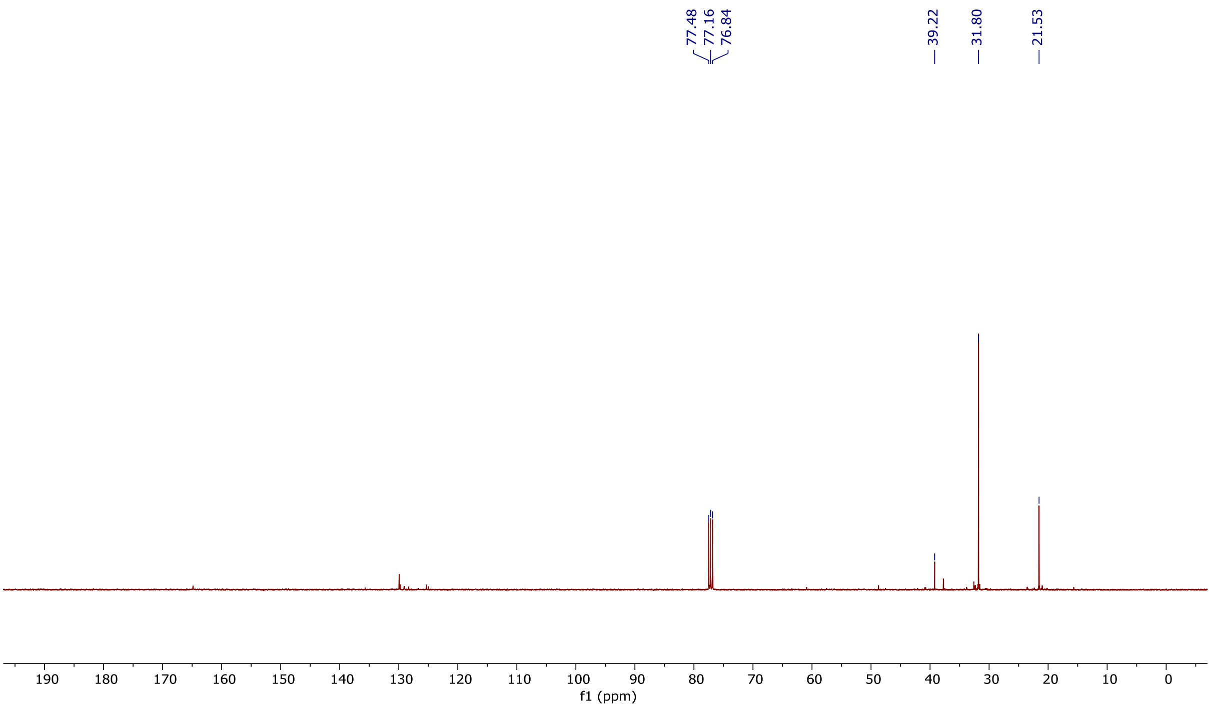

^{1}H NMR (400 MHz, $CDCl_3$) 4-Methyl-2,6,7-trithiabicyclo[2.2.2]octane (69):

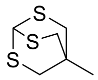

69

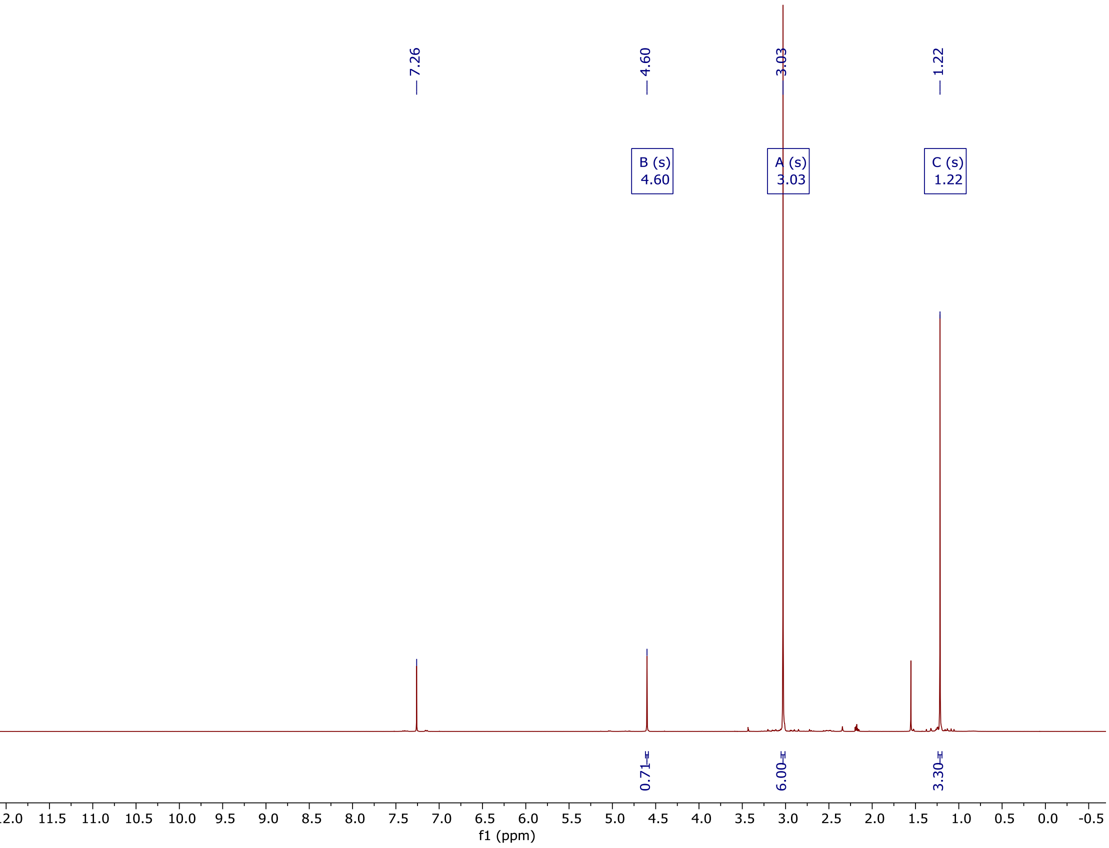

^{13}C NMR (101 MHz, $CDCl_3$):

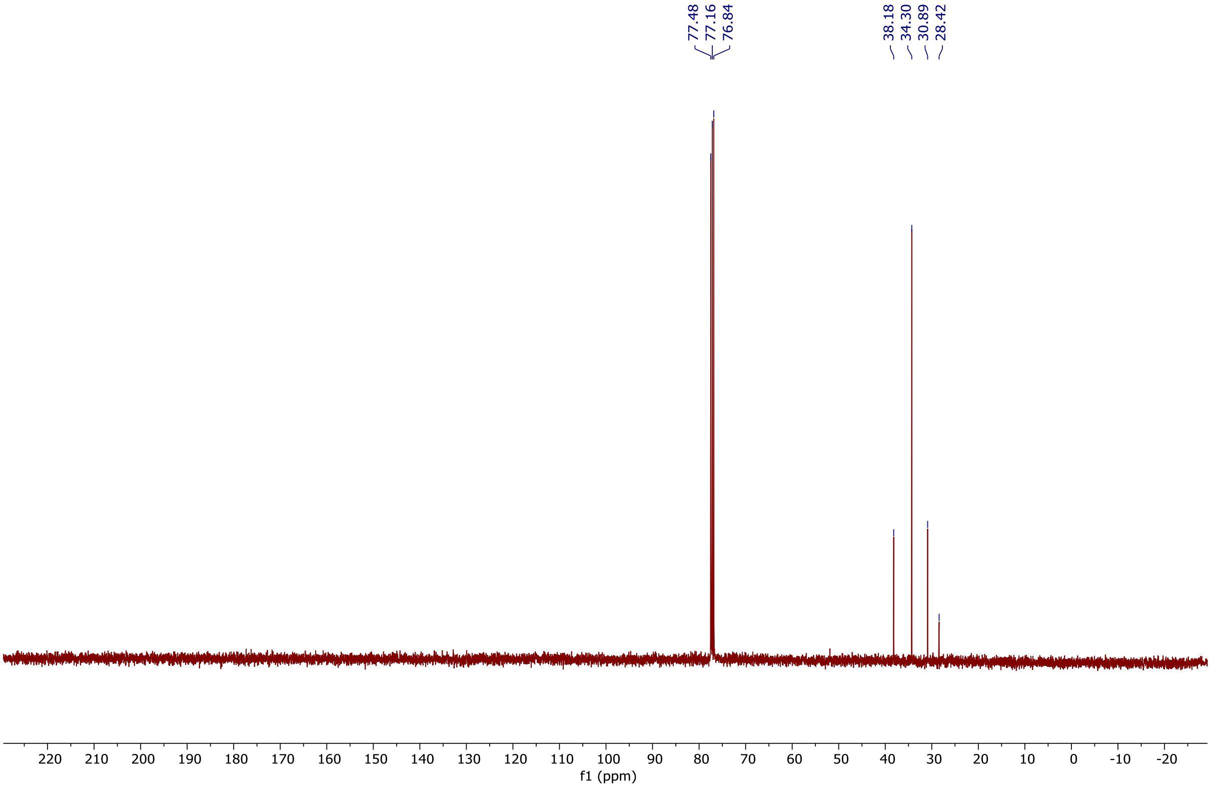

^{1}H NMR (400 MHz, $CDCl_3$) (2,3-Dihydrobenzofuran-3-yl)(4-methyl-2,6,7-trithiabicyclo[2.2.2]octan-1-yl)methanone (70):

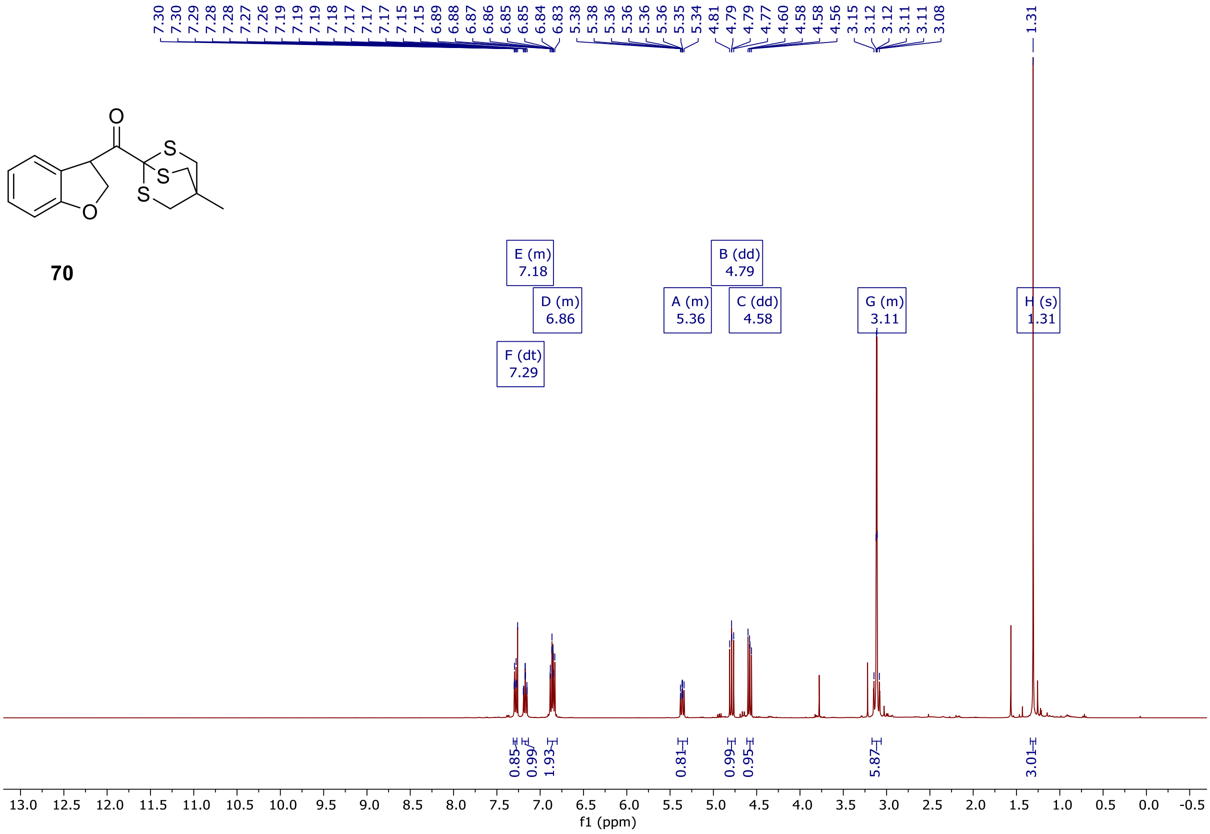

^{13}C NMR (101 MHz, $CDCl_3$):

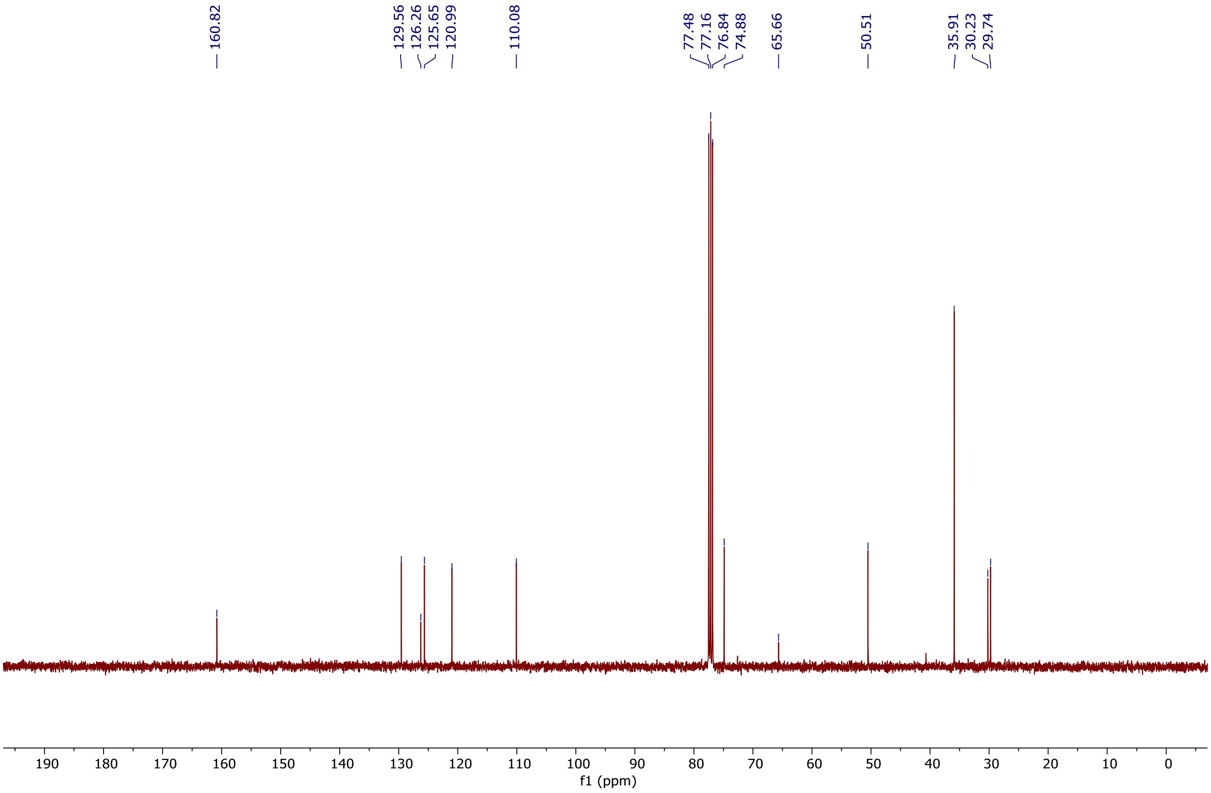

^{1}H NMR (400 MHz, $CDCl_3$) 2-(Triphenyl-λ^5-phosphaneylidene)acetonitrile (72):

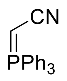

72

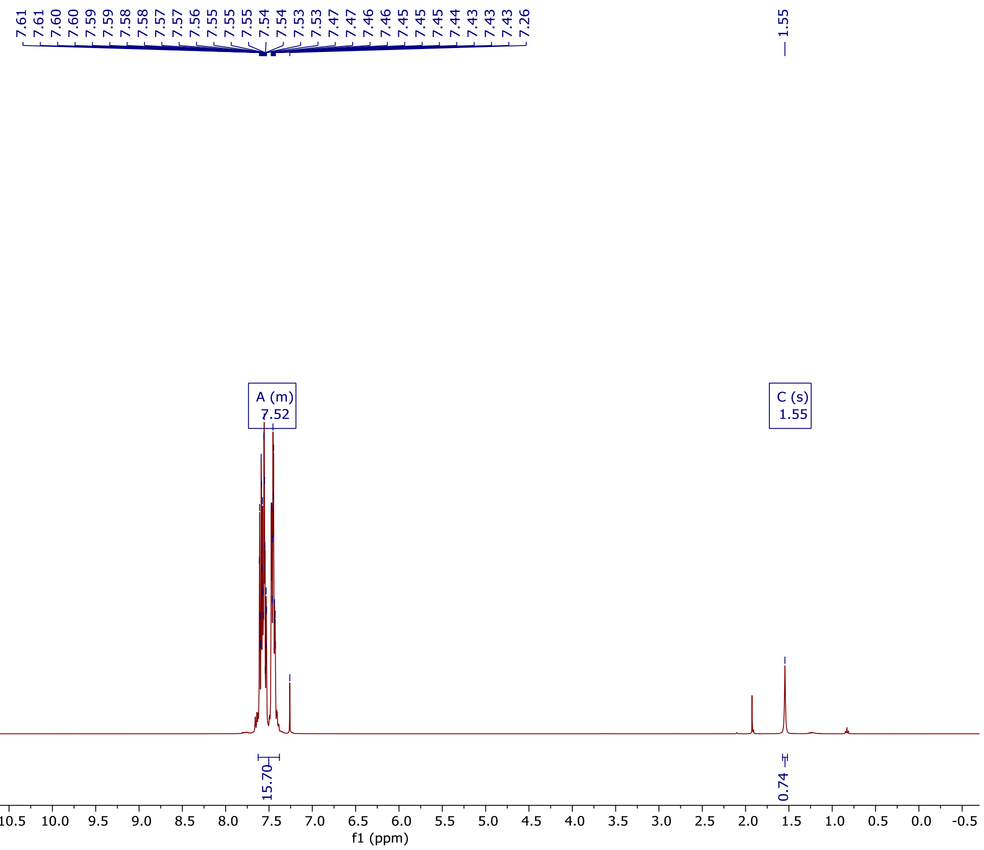

^{13}C NMR (101 MHz, $CDCl_3$):

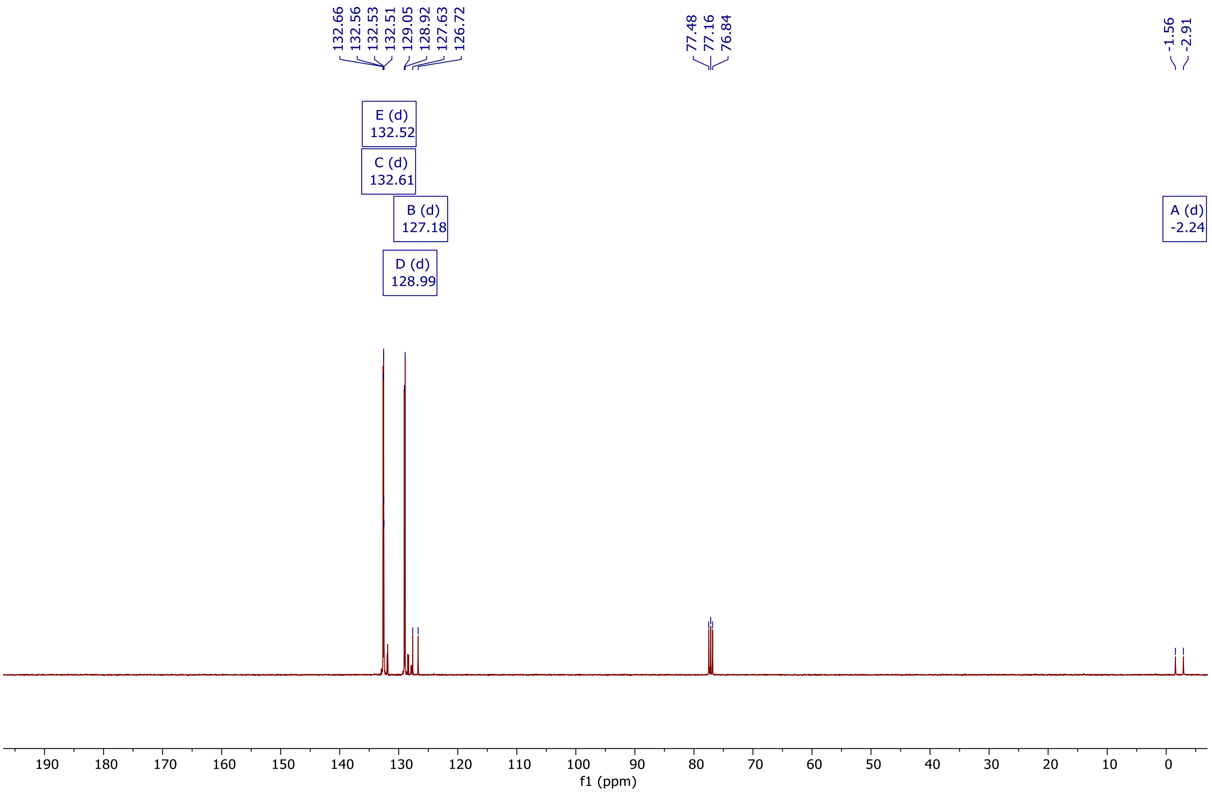

^{31}P NMR (126 MHz, $CDCl_3$):

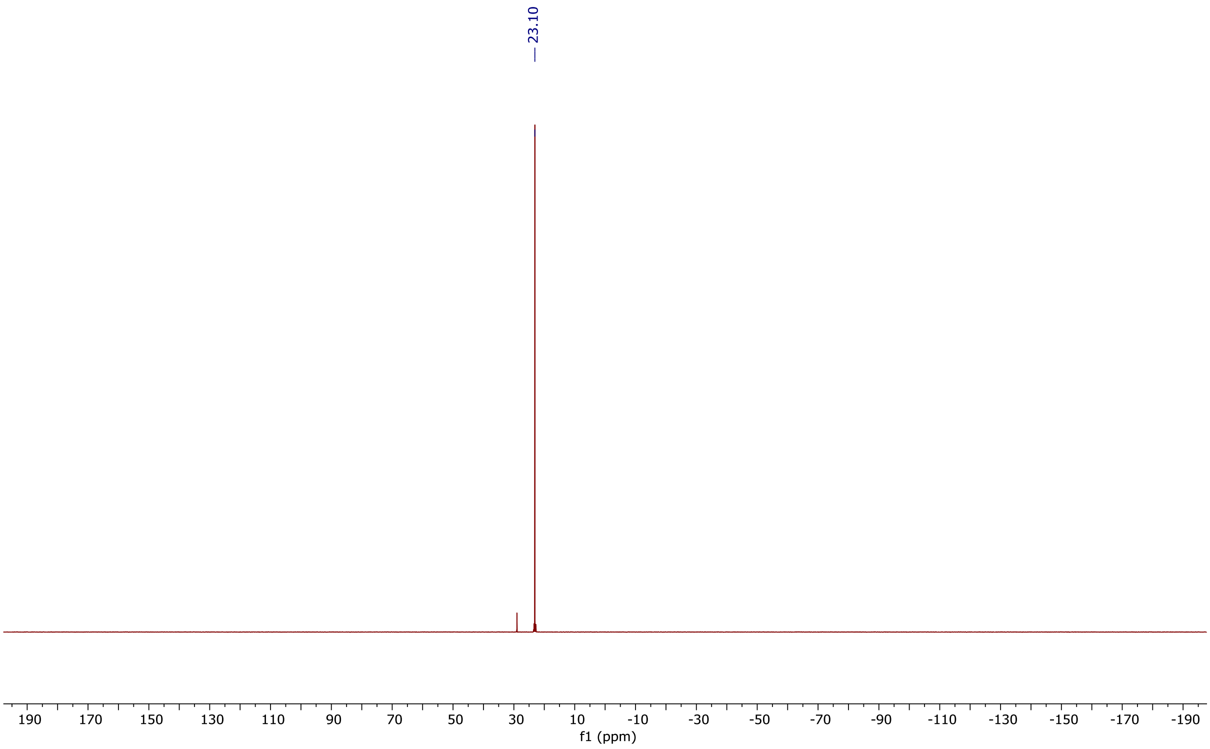

^{1}H NMR (400 MHz, $CDCl_3$) 3-(2,3-Dihydrobenzofuran-3-yl)-3-oxo-2-(triphenyl-λ^{5}-phosphaneylidene)propanenitrile (73):

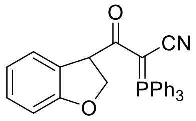

73

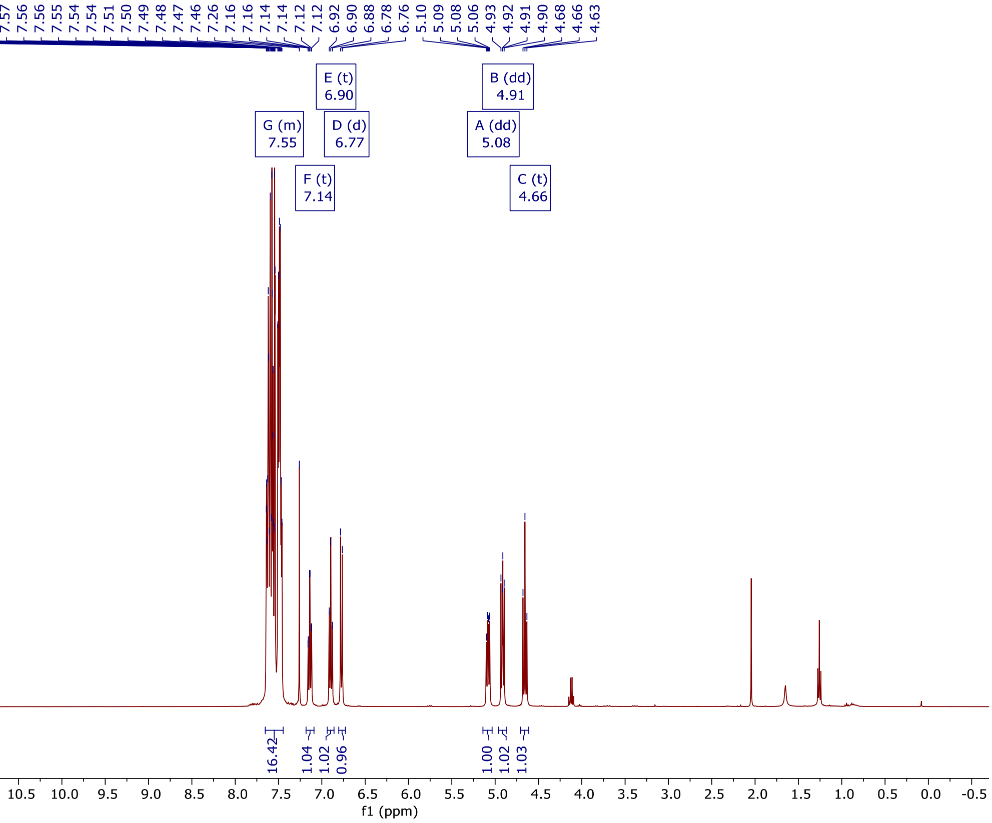

^{13}C NMR (101 MHz, $CDCl_3$):

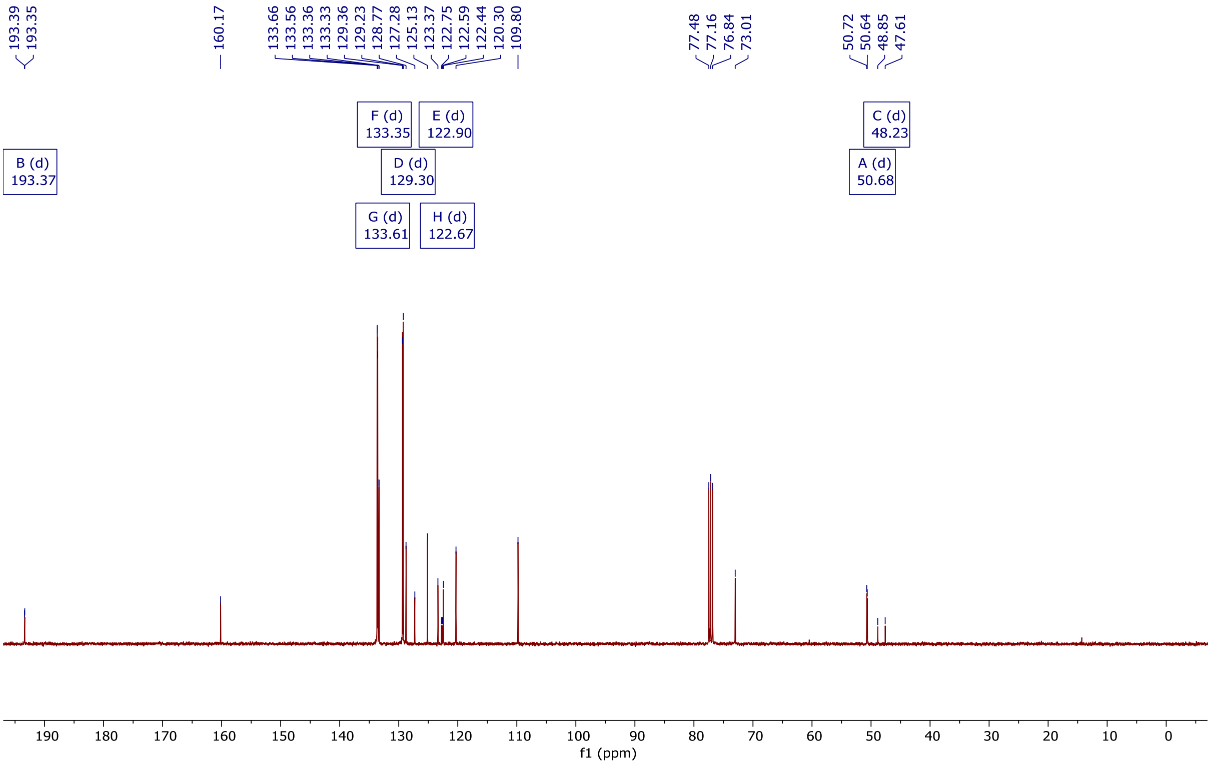

^{31}P NMR (126 MHz, $CDCl_3$):

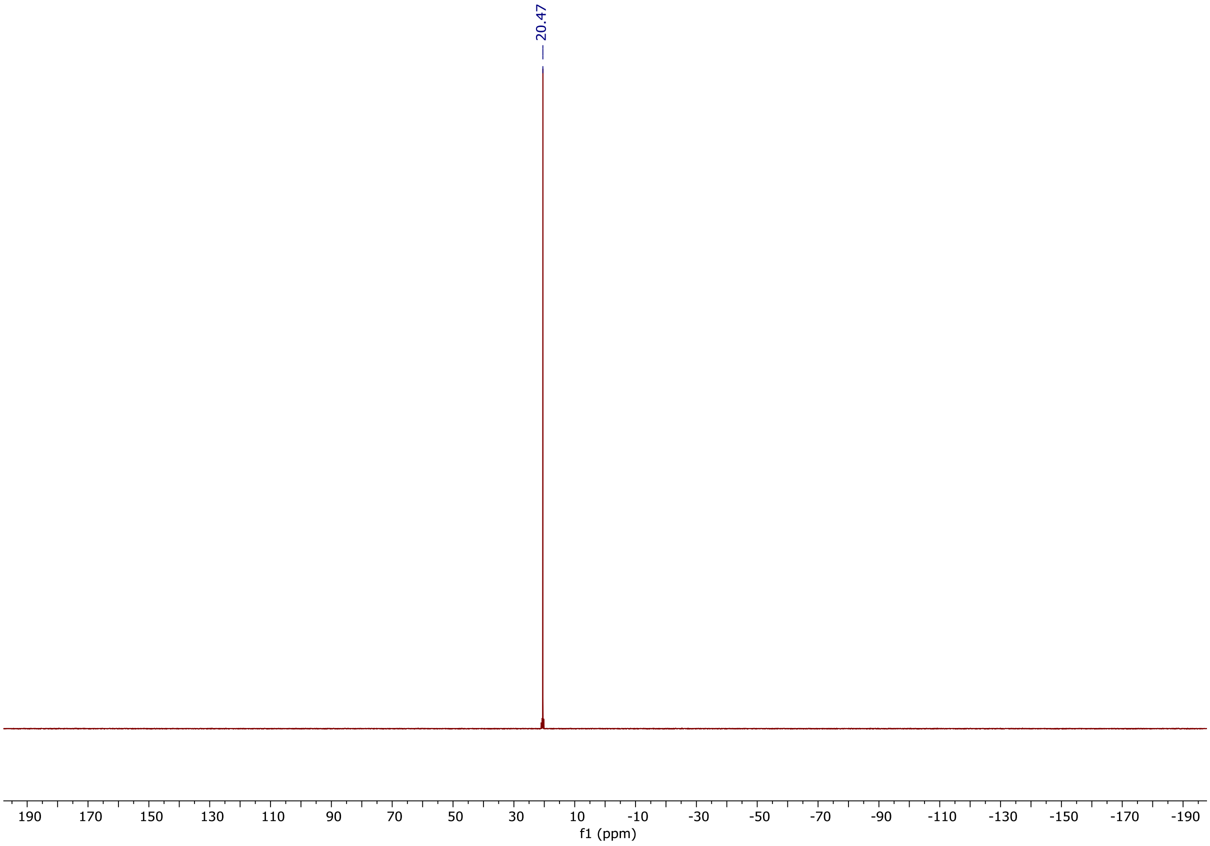

^{1}H NMR (400 MHz, $CDCl_3$) Methyl (*E*)-3-(1-methoxy-1-oxoocta-2,7-dien-3-yl)-2,3-dihydrobenzofuran-3-carboxylate (74):

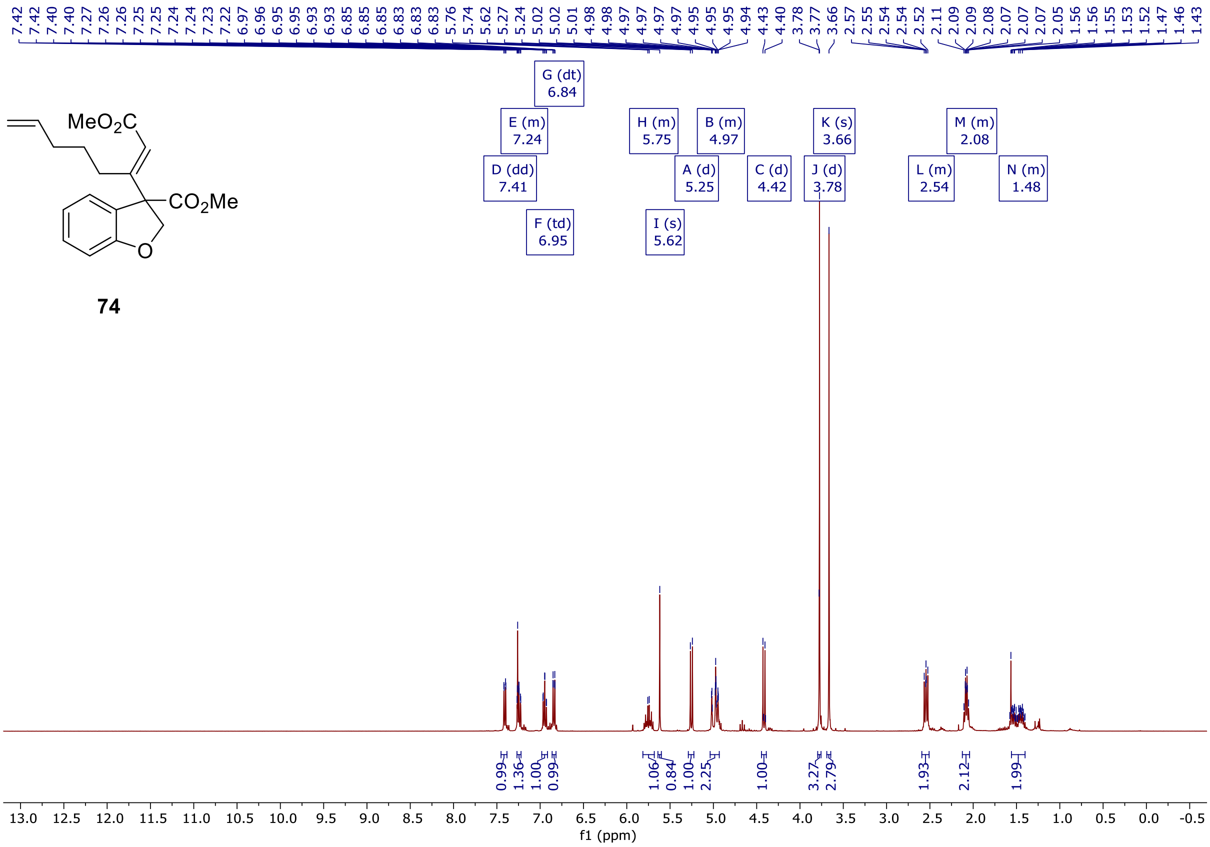

^{13}C NMR (126 MHz, $CDCl_3$):

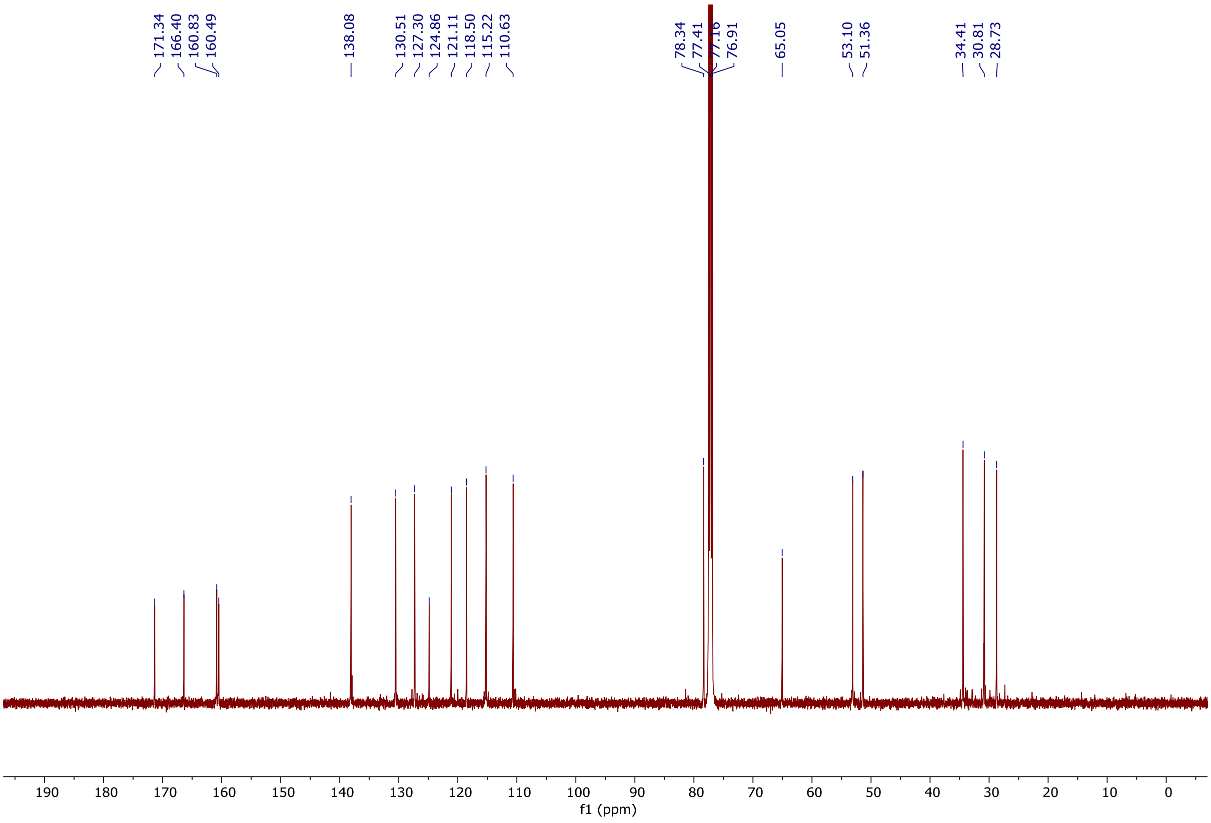

^{1}H NMR (400 MHz, $CDCl_3$) Methyl (*Z*)-3-(1-methoxy-1-oxoocta-2,7-dien-3-yl)-2,3-dihydrobenzofuran-3-carboxylate (75)

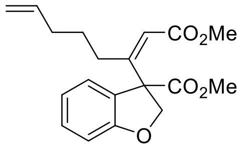

75

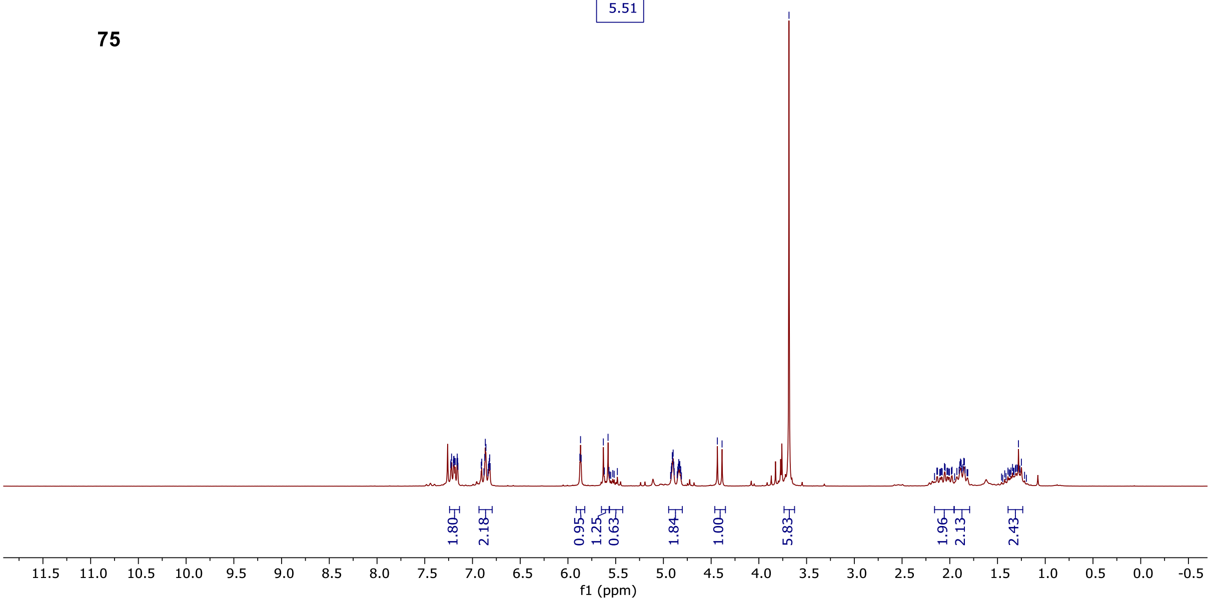

^{13}C NMR (101 MHz, $CDCl_3$):

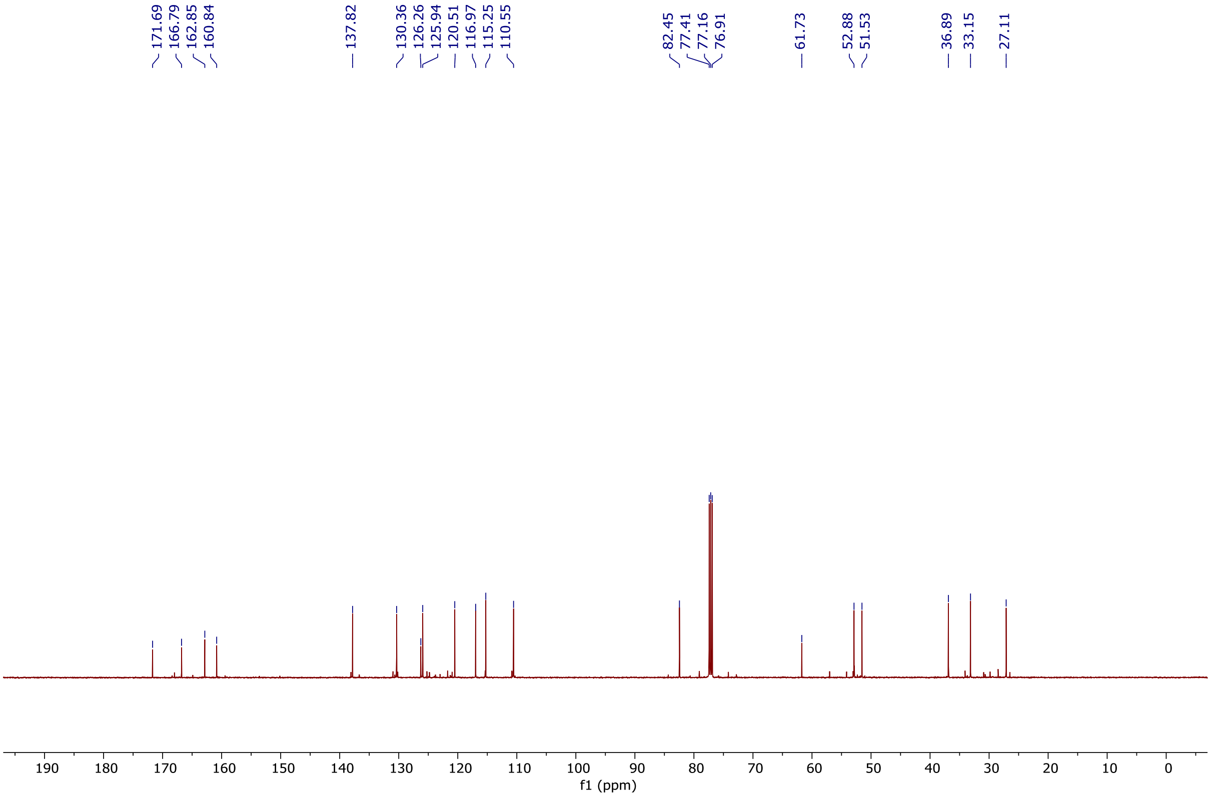

1.4 NOESY spectra of enone 31, 32, 33, 74, and 75

Methyl (*Z*)-3-(4-methoxy-4-oxobut-2-en-2-yl)-2,3-dihydrobenzofuran-3-carboxylate (31)

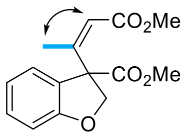

31

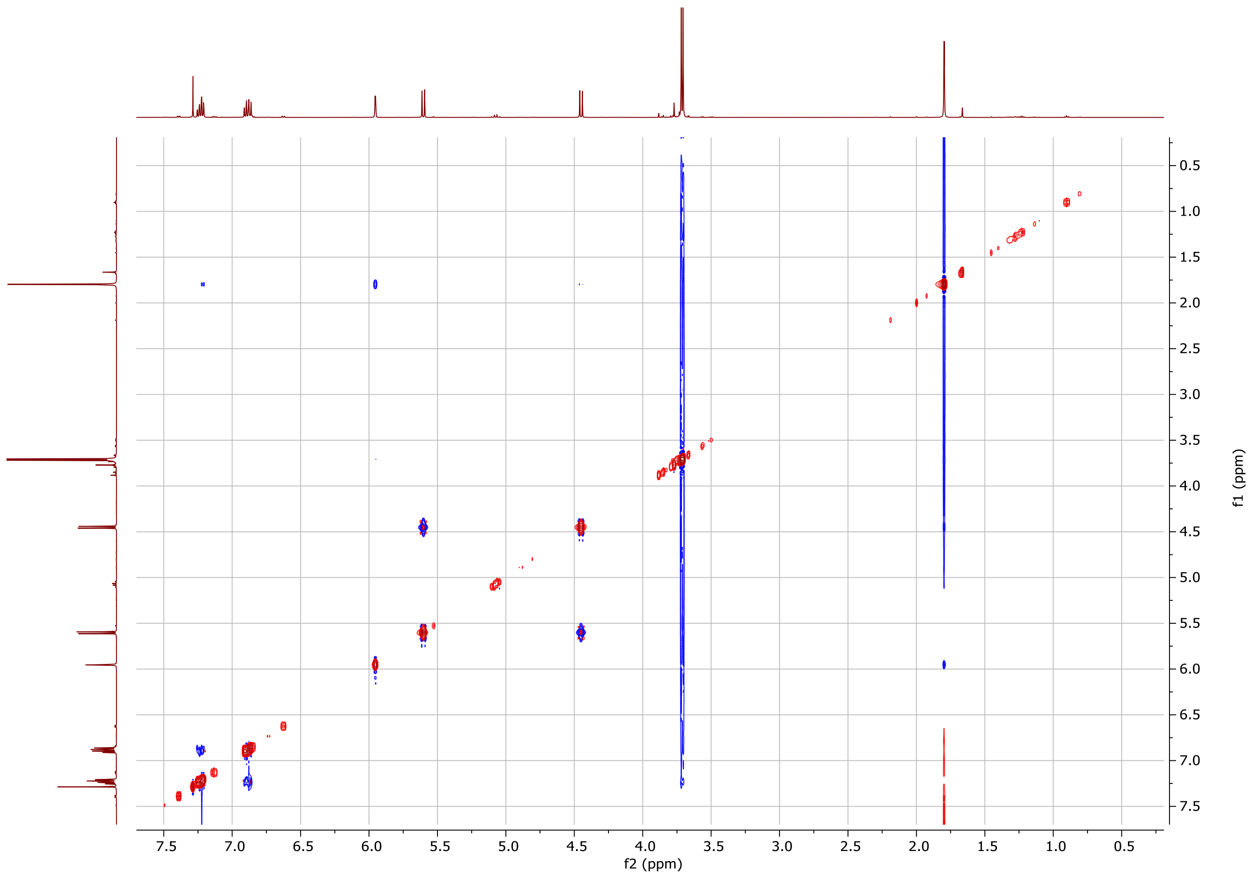

Methyl (Z)-3-(1-methoxy-1-oxohept-2-en-3-yl)-2,3-dihydrobenzofuran-3-carboxylate (32):

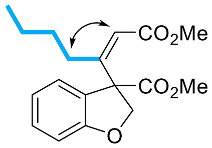

32

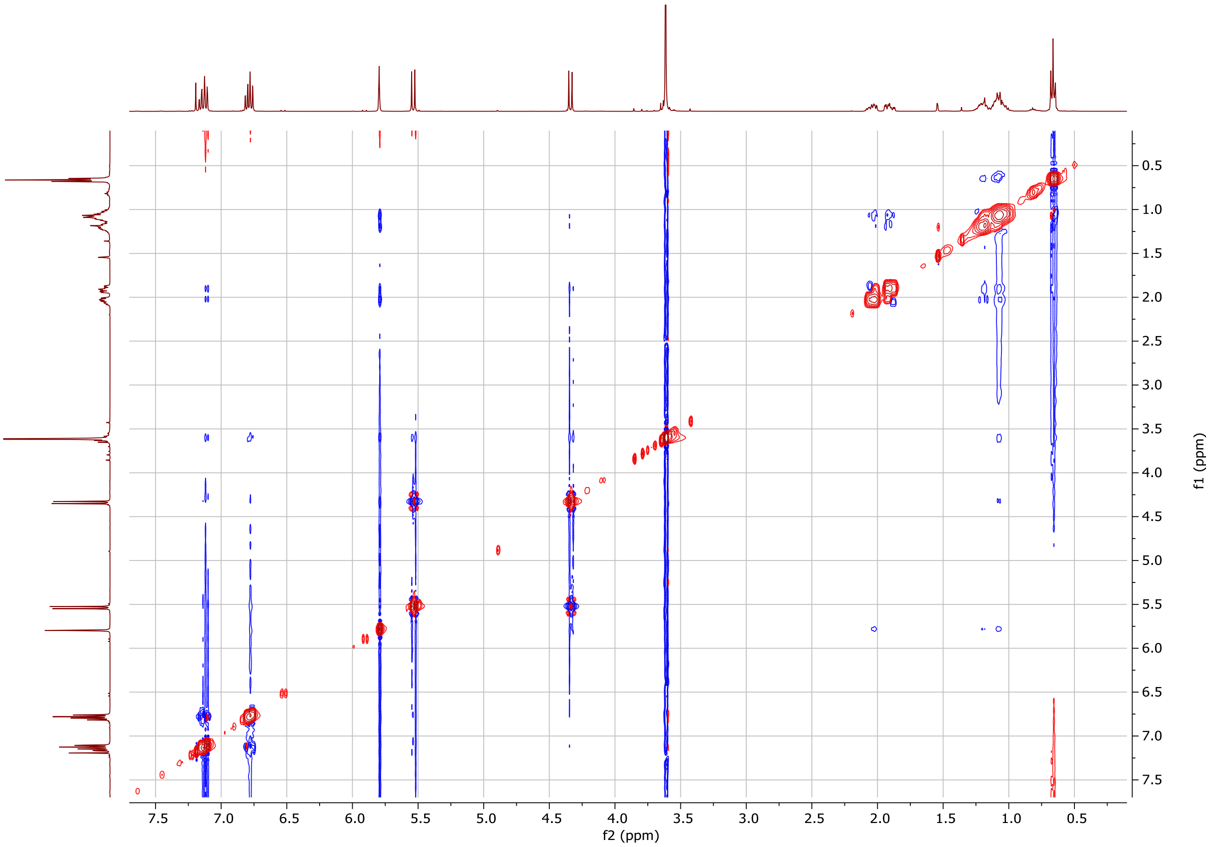

Methyl (Z)-3-(1-methoxy-5-methyl-1-oxohex-2-en-3-yl)-2,3-dihydrobenzofuran-3-carboxylate (33):

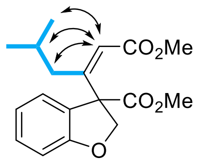

33

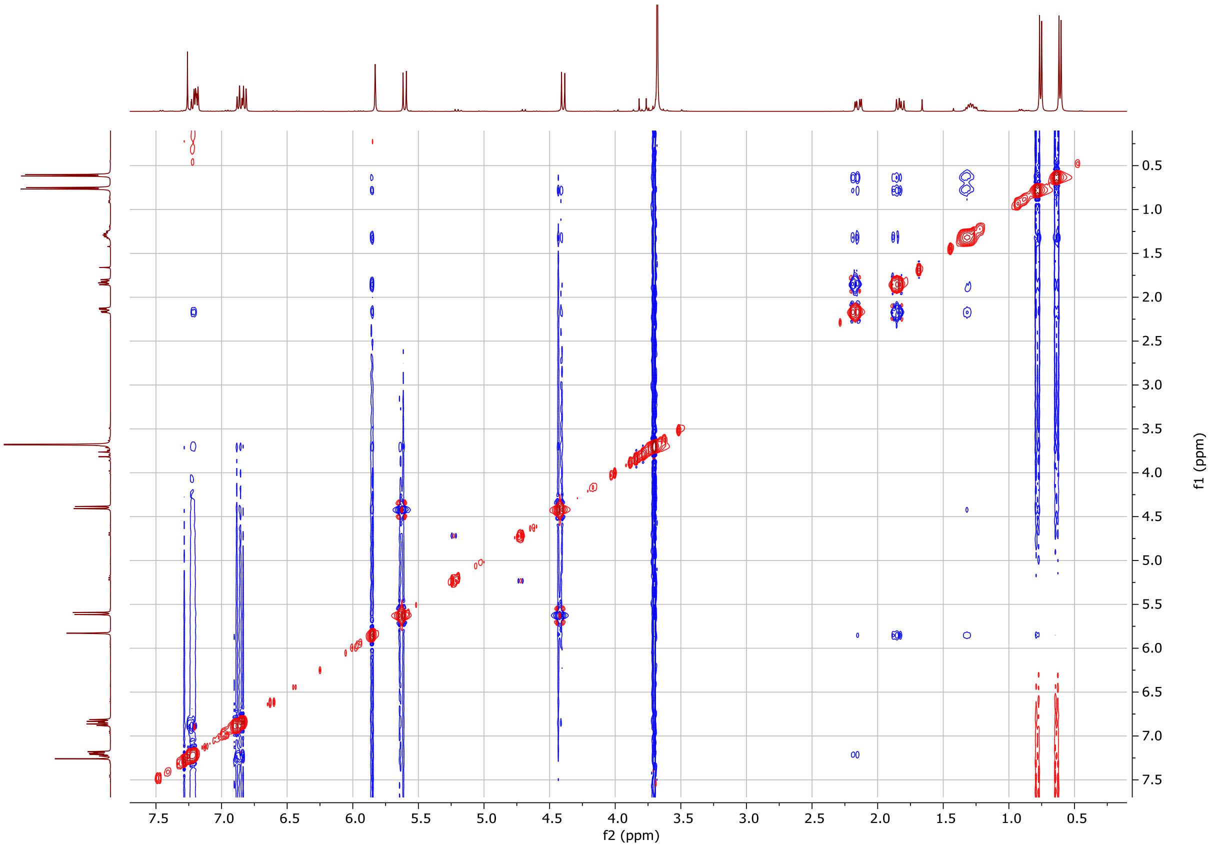

^{1}H NMR (400 MHz, $CDCl_3$) Methyl (*E*)-3-(1-methoxy-1-oxoocta-2,7-dien-3-yl)-2,3-dihydrobenzofuran-3-carboxylate (74):

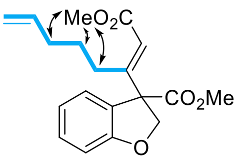

74

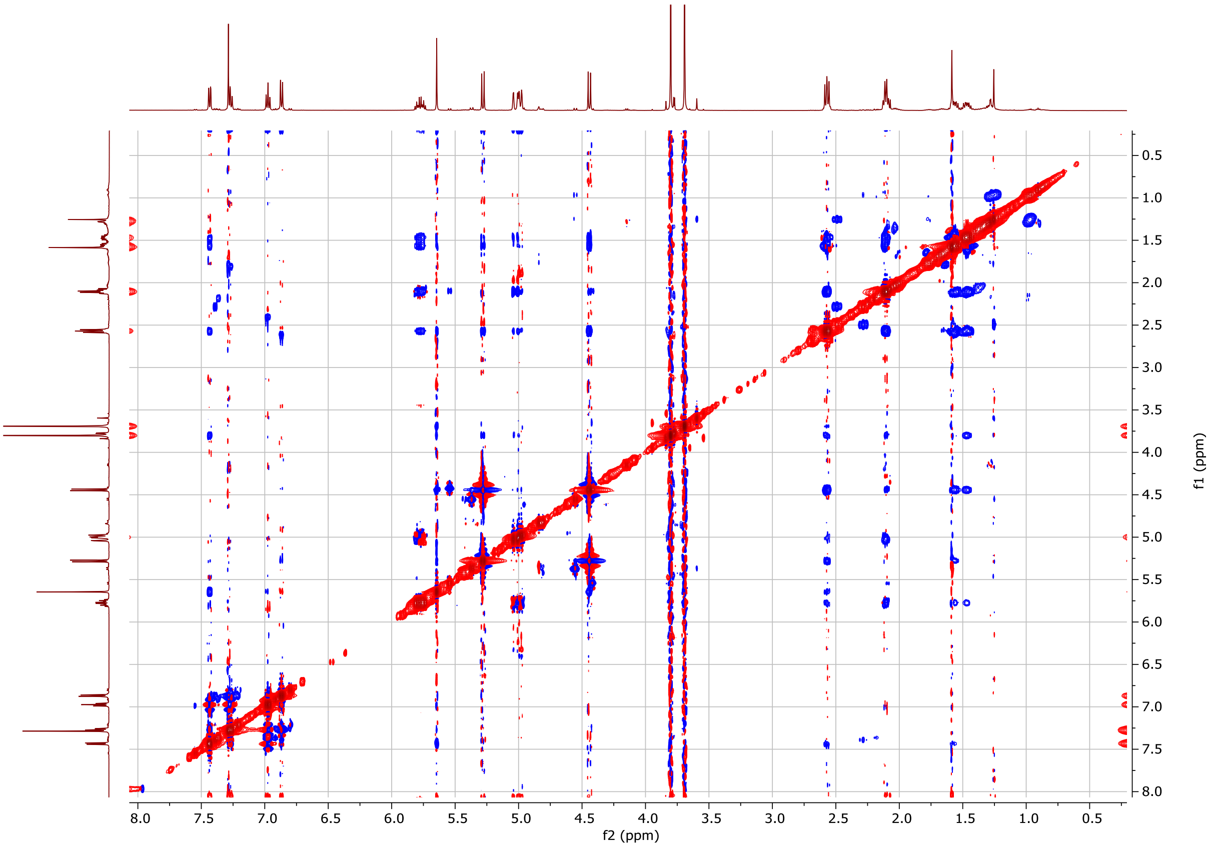

^{1}H NMR (400 MHz, CDCl3) Methyl (*Z*)-3-(1-methoxy-1-oxoocta-2,7-dien-3-yl)-2,3-dihydrobenzofuran-3-carboxylate (75):

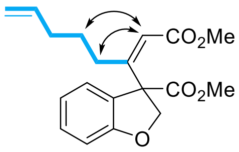

75

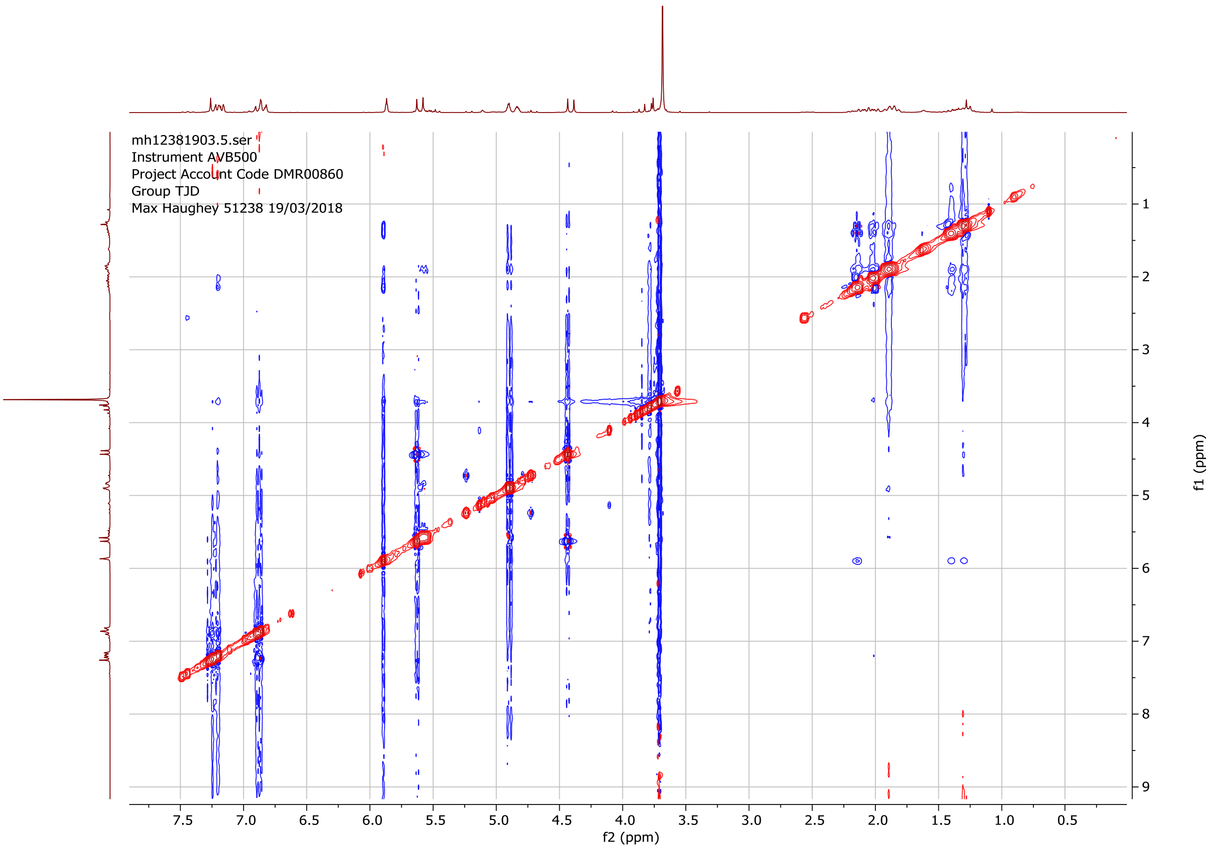

1.5 Supplementary CIF data

Methyl (*Z*)-3-(4-methoxy-4-oxobut-2-en-2-yl)-2,3-dihydrobenzofuran-3-carboxylate (31)

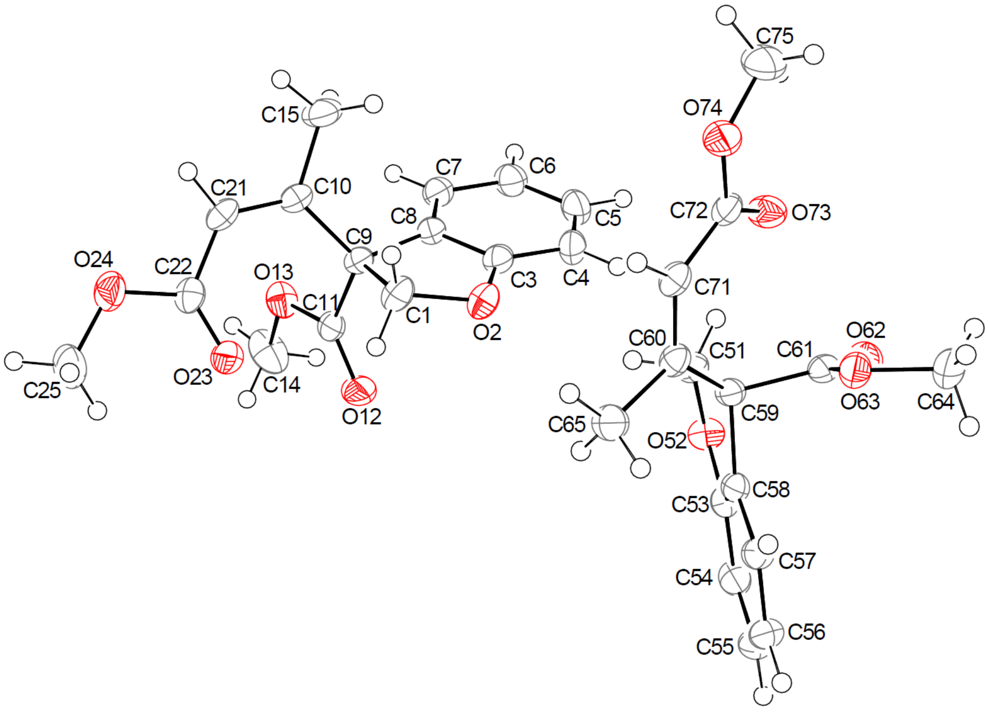

The structure contains two molecules in the asymmetric unit. The numbering scheme has been offset by 50 between the two molecules.

Table 1. Crystal data and structure refinement

Identification code	7002	
Empirical formula	$C_{15}H_{16}O_5$	
Formula weight	276.29	
Temperature	150 K	
Wavelength	1.54184 Å	
Crystal system	Orthorhombic	
Space group	P c a 21	
Unit cell dimensions	a = 14.0413(3) Å	α = 90°
	b = 7.0334(2) Å	β = 90°
	c = 27.4163(9) Å	γ = 90°

Volume	2707.58(13) Å^3
Z	8
Density (calculated)	1.355 Mg/m^3
Absorption coefficient	0.851 mm^{-1}
F(000)	1167.994
Crystal size	0.21 × 0.18 × 0.03 mm^3
Theta range for data collection	3.224 to 76.653°.
Index ranges	$-10 \le h \le 17$, $-8 \le k \le 8$, $-34 \le l \le 33$
Reflections collected	13755
Independent reflections	5388 [R(int) = 0.027]
Completeness to theta = 76.653°	99.6%
Absorption correction	Semi–empirical from equivalents
Max. and min. transmission	0.97 and 0.72
Refinement method	Full–matrix least–squares on F^2
Data / restraints / parameters	5386 / 1 / 362
Goodness–of–fit on F^2	0.9983
Final R indices [I>2sigma(I)]	R1 = 0.0355, wR2 = 0.0906
R indices (all data)	R1 = 0.0391, wR2 = 0.0950
Absolute structure parameter	–0.05(9)
Largest diff. peak and hole	0.25 and –0.18 e.Å^{-3}

Table 2. Atomic coordinates (× 10^4) and equivalent isotropic displacement parameters (Å^2 × 10^3). U(eq) is defined as one third of the trace of the orthogonalized U^{ij} tensor.

	X	y	z	U (eq)
C(1)	5946(2)	3292(3)	5000(1)	33
O(2)	6778(1)	3733(2)	5293(1)	35
C(3)	7029(1)	5560(3)	5189(1)	31
C(4)	7755(2)	6552(3)	5425(1)	37
C(5)	7943(2)	8380(3)	5266(1)	40

C(6)	7437(2)	9202(3)	4886(1)	37
C(7)	6718(1)	8191(3)	4651(1)	31
C(8)	6509(1)	6368(3)	4812(1)	26
C(9)	5844(1)	4843(3)	4605(1)	25
C(10)	4796(1)	5401(3)	4552(1)	27
C(11)	6354(1)	4275(3)	4133(1)	27
O(12)	7007(1)	3174(2)	4121(1)	40
O(13)	6063(1)	5331(2)	3755(1)	34
C(14)	6657(2)	5196(4)	3323(1)	50
C(15)	4480(2)	7148(3)	4822(1)	42
C(21)	4143(1)	4361(3)	4316(1)	30
C(22)	4310(1)	2520(3)	4076(1)	29
O(23)	5024(1)	1571(2)	4098(1)	37
O(24)	3533(1)	1990(2)	3824(1)	41
C(25)	3561(2)	124(4)	3604(1)	51
C(51)	8188(1)	1771(3)	6459(1)	28
O(52)	9065(1)	1376(2)	6196(1)	31
C(53)	9323(1)	–444(3)	6311(1)	26
C(54)	10080(1)	–1388(3)	6097(1)	32
C(55)	10267(1)	–3231(3)	6258(1)	34
C(56)	9721(1)	–4076(3)	6620(1)	32
C(57)	8974(1)	–3080(3)	6839(1)	29
C(58)	8773(1)	–1255(3)	6675(1)	24
C(59)	8093(1)	271(2)	6871(1)	24
C(60)	7051(1)	–315(3)	6938(1)	27

C(61)	8609(1)	912(3)	7333(1)	27
O(62)	9253(1)	2040(2)	7333(1)	36
O(63)	8345(1)	–106(2)	7721(1)	33
C(64)	8956(2)	83(4)	8146(1)	45
C(65)	6731(1)	–2083(3)	6675(1)	36
C(71)	6407(1)	712(3)	7181(1)	29
C(72)	6570(1)	2551(3)	7419(1)	29
O(73)	7263(1)	3552(2)	7375(1)	36
O(74)	5818(1)	3030(2)	7696(1)	40
C(75)	5842(2)	4906(4)	7915(1)	53

Table 3. Bond lengths [Å] and angles [°]

C(1)–O(2)	1.452(2)	C(1)–C(9)–C(10)	109.71(15)
C(1)–C(9)	1.543(3)	C(8)–C(9)–C(10)	116.26(15)
C(1)–H(12)	0.977	C(1)–C(9)–C(11)	111.40(16)
C(1)–H(11)	0.97	C(8)–C(9)–C(11)	102.17(14)
O(2)–C(3)	1.363(3)	C(10)–C(9)–C(11)	115.82(16)
C(3)–C(4)	1.395(3)	C(9)–C(10)–C(15)	116.57(17)
C(3)–C(8)	1.387(3)	C(9)–C(10)–C(21)	124.38(17)
C(4)–C(5)	1.383(3)	C(15)–C(10)–C(21)	118.83(18)
C(4)–H(41)	0.93	C(9)–C(11)–O(12)	123.23(19)
C(5)–C(6)	1.387(3)	C(9)–C(11)–O(13)	111.57(15)
C(5)–H(51)	0.928	O(12)–C(11)–O(13)	124.67(19)
C(6)–C(7)	1.394(3)	C(11)–O(13)–C(14)	114.89(18)

C(6)–H(61)	0.957	O(13)–C(14)–H(141)	104.8
C(7)–C(8)	1.388(3)	O(13)–C(14)–H(142)	107.4
C(7)–H(71)	0.964	H(141)–C(14)–H(142)	110
C(8)–C(9)	1.531(3)	O(13)–C(14)–H(143)	111.7
C(9)–C(10)	1.529(2)	H(141)–C(14)–H(143)	109.6
C(9)–C(11)	1.532(3)	H(142)–C(14)–H(143)	112.8
C(10)–C(15)	1.502(3)	C(10)–C(15)–H(152)	108.8
C(10)–C(21)	1.339(3)	C(10)–C(15)–H(153)	111
C(11)–O(12)	1.201(2)	H(152)–C(15)–H(153)	110.1
C(11)–O(13)	1.338(3)	C(10)–C(15)–H(151)	110.5
O(13)–C(14)	1.450(2)	H(152)–C(15)–H(151)	108.4
C(14)–H(141)	0.963	H(153)–C(15)–H(151)	108
C(14)–H(142)	0.973	C(10)–C(21)–C(22)	125.97(17)
C(14)–H(143)	0.965	C(10)–C(21)–H(211)	117.6
C(15)–H(152)	0.958	C(22)–C(21)–H(211)	116.4
C(15)–H(153)	0.995	C(21)–C(22)–O(23)	126.63(18)
C(15)–H(151)	0.962	C(21)–C(22)–O(24)	110.17(17)
C(21)–C(22)	1.472(3)	O(23)–C(22)–O(24)	123.19(19)
C(21)–H(211)	0.933	C(22)–O(24)–C(25)	116.40(18)
C(22)–O(23)	1.205(2)	O(24)–C(25)–H(252)	105.5
C(22)–O(24)	1.344(2)	O(24)–C(25)–H(253)	108
O(24)–C(25)	1.445(3)	H(252)–C(25)–H(253)	111.5
C(25)–H(252)	0.984	O(24)–C(25)–H(251)	110.9
C(25)–H(253)	0.996	H(252)–C(25)–H(251)	111.5
C(25)–H(251)	0.948	H(253)–C(25)–H(251)	109.4

C(51)–O(52)	1.453(2)	O(52)–C(51)–C(59)	107.68(15)
C(51)–C(59)	1.553(3)	O(52)–C(51)–H(512)	108.7
C(51)–H(512)	0.961	C(59)–C(51)–H(512)	113.1
C(51)–H(511)	0.977	O(52)–C(51)–H(511)	108.2
O(52)–C(53)	1.367(2)	C(59)–C(51)–H(511)	109.3
C(53)–C(54)	1.383(3)	H(512)–C(51)–H(511)	109.8
C(53)–C(58)	1.385(3)	C(51)–O(52)–C(53)	106.79(15)
C(54)–C(55)	1.394(3)	O(52)–C(53)–C(54)	123.68(19)
C(54)–H(541)	0.926	O(52)–C(53)–C(58)	113.89(17)
C(55)–C(56)	1.387(3)	C(54)–C(53)–C(58)	122.40(18)
C(55)–H(551)	0.925	C(53)–C(54)–C(55)	117.21(19)
C(56)–C(57)	1.398(3)	C(53)–C(54)–H(541)	120.2
C(56)–H(561)	0.968	C(55)–C(54)–H(541)	122.5
C(57)–C(58)	1.389(3)	C(54)–C(55)–C(56)	121.37(19)
C(57)–H(571)	0.96	C(54)–C(55)–H(551)	119.9
C(58)–C(59)	1.534(2)	C(56)–C(55)–H(551)	118.8
C(59)–C(60)	1.531(2)	C(55)–C(56)–C(57)	120.49(19)
C(59)–C(61)	1.527(3)	C(55)–C(56)–H(561)	119.5
C(60)–C(65)	1.506(3)	C(57)–C(56)–H(561)	120
C(60)–C(71)	1.336(3)	C(56)–C(57)–C(58)	118.44(19)
C(61)–O(62)	1.202(2)	C(56)–C(57)–H(571)	120.6
C(61)–O(63)	1.335(3)	C(58)–C(57)–H(571)	120.9
O(63)–C(64)	1.455(2)	C(57)–C(58)–C(53)	120.06(17)
C(64)–H(641)	0.978	C(57)–C(58)–C(59)	131.30(18)
C(64)–H(642)	0.992	C(53)–C(58)–C(59)	108.11(16)

C(64)–H(643)	0.969	C(58)–C(59)–C(51)	99.63(15)
C(65)–H(652)	0.963	C(58)–C(59)–C(60)	116.65(14)
C(65)–H(653)	0.977	C(51)–C(59)–C(60)	110.65(15)
C(65)–H(651)	0.987	C(58)–C(59)–C(61)	101.59(14)
C(71)–C(72)	1.467(3)	C(51)–C(59)–C(61)	111.24(15)
C(71)–H(711)	0.941	C(60)–C(59)–C(61)	115.72(16)
C(72)–O(73)	1.207(2)	C(59)–C(60)–C(65)	116.79(16)
C(72)–O(74)	1.343(2)	C(59)–C(60)–C(71)	124.15(17)
O(74)–C(75)	1.450(3)	C(65)–C(60)–C(71)	118.86(17)
C(75)–H(752)	0.94	C(59)–C(61)–O(62)	123.56(18)
C(75)–H(753)	0.933	C(59)–C(61)–O(63)	111.73(15)
C(75)–H(751)	0.968	O(62)–C(61)–O(63)	124.18(18)
O(2)–C(1)–C(9)	108.15(16)	C(61)–O(63)–C(64)	115.20(17)
O(2)–C(1)–H(12)	106.6	O(63)–C(64)–H(641)	107.4
C(9)–C(1)–H(12)	110	O(63)–C(64)–H(642)	112.7
O(2)–C(1)–H(11)	108.7	H(641)–C(64)–H(642)	111.7
C(9)–C(1)–H(11)	109.4	O(63)–C(64)–H(643)	107.6
H(12)–C(1)–H(11)	113.8	H(641)–C(64)–H(643)	107.2
C(1)–O(2)–C(3)	107.12(16)	H(642)–C(64)–H(643)	110
O(2)–C(3)–C(4)	124.34(19)	C(60)–C(65)–H(652)	110.2
O(2)–C(3)–C(8)	113.92(18)	C(60)–C(65)–H(653)	109.1
C(4)–C(3)–C(8)	121.7(2)	H(652)–C(65)–H(653)	109.7
C(3)–C(4)–C(5)	117.3(2)	C(60)–C(65)–H(651)	109.6
C(3)–C(4)–H(41)	120.8	H(652)–C(65)–H(651)	111.2
C(5)–C(4)–H(41)	121.9	H(653)–C(65)–H(651)	107

C(4)–C(5)–C(6)	121.8(2)	C(60)–C(71)–C(72)	126.39(17)
C(4)–C(5)–H(51)	119.5	C(60)–C(71)–H(711)	119.2
C(6)–C(5)–H(51)	118.8	C(72)–C(71)–H(711)	114.4
C(5)–C(6)–C(7)	120.4(2)	C(71)–C(72)–O(73)	126.53(18)
C(5)–C(6)–H(61)	121	C(71)–C(72)–O(74)	110.50(16)
C(7)–C(6)–H(61)	118.6	O(73)–C(72)–O(74)	122.96(19)
C(6)–C(7)–C(8)	118.5(2)	C(72)–O(74)–C(75)	116.34(17)
C(6)–C(7)–H(71)	121	O(74)–C(75)–H(752)	109.6
C(8)–C(7)–H(71)	120.5	O(74)–C(75)–H(753)	104.7
C(7)–C(8)–C(3)	120.33(18)	H(752)–C(75)–H(753)	112.3
C(7)–C(8)–C(9)	131.12(18)	O(74)–C(75)–H(751)	110.1
C(3)–C(8)–C(9)	108.09(17)	H(752)–C(75)–H(751)	110.1
C(1)–C(9)–C(8)	100.30(15)	H(753)–C(75)–H(751)	109.8

Table 4. Anisotropic displacement parameters ($Å^2 \times 10^3$). The anisotropic displacement factor exponent takes the form: $-2\pi^2[h^2 a^{*2}U^{11} + ... + 2 h k a^* b^* U^{12}]$

	U^{11}	U^{22}	U^{33}	U^{23}	U^{13}	U^{12}
C(1)	29(1)	33(1)	39(1)	8(1)	–4(1)	1(1)
O(2)	31(1)	38(1)	34(1)	7(1)	–6(1)	3(1)
C(3)	27(1)	36(1)	28(1)	–3(1)	1(1)	6(1)
C(4)	32(1)	50(1)	29(1)	–6(1)	–8(1)	4(1)
C(5)	32(1)	47(1)	41(1)	–15(1)	–5(1)	–4(1)
C(6)	32(1)	33(1)	45(1)	–6(1)	–1(1)	–4(1)
C(7)	28(1)	29(1)	35(1)	–2(1)	–1(1)	1(1)

C(8)	23(1)	29(1)	26(1)	–3(1)	0(1)	2(1)
C(9)	22(1)	24(1)	29(1)	1(1)	1(1)	2(1)
C(10)	23(1)	29(1)	30(1)	3(1)	3(1)	4(1)
C(11)	18(1)	31(1)	33(1)	–5(1)	1(1)	–4(1)
O(12)	23(1)	44(1)	52(1)	–16(1)	4(1)	3(1)
O(13)	32(1)	44(1)	26(1)	–1(1)	4(1)	–7(1)
C(14)	53(1)	63(2)	33(1)	–7(1)	16(1)	–20(1)
C(15)	28(1)	38(1)	61(2)	–9(1)	6(1)	8(1)
C(21)	18(1)	36(1)	37(1)	5(1)	2(1)	2(1)
C(22)	21(1)	37(1)	30(1)	3(1)	2(1)	–6(1)
O(23)	26(1)	36(1)	49(1)	–9(1)	–4(1)	0(1)
O(24)	25(1)	54(1)	44(1)	–6(1)	–4(1)	–4(1)
C(25)	33(1)	59(2)	60(2)	–18(1)	–5(1)	–13(1)
C(51)	25(1)	27(1)	33(1)	0(1)	1(1)	0(1)
O(52)	30(1)	29(1)	34(1)	3(1)	6(1)	0(1)
C(53)	25(1)	26(1)	27(1)	–3(1)	–3(1)	–2(1)
C(54)	26(1)	39(1)	31(1)	–6(1)	3(1)	–3(1)
C(55)	28(1)	38(1)	36(1)	–10(1)	1(1)	6(1)
C(56)	30(1)	28(1)	39(1)	–3(1)	–3(1)	5(1)
C(57)	26(1)	27(1)	32(1)	–2(1)	–1(1)	0(1)
C(58)	19(1)	27(1)	28(1)	–5(1)	–1(1)	–1(1)
C(59)	21(1)	22(1)	28(1)	–2(1)	0(1)	1(1)
C(60)	21(1)	26(1)	33(1)	2(1)	–3(1)	–2(1)
C(61)	20(1)	30(1)	31(1)	–4(1)	1(1)	4(1)
O(62)	24(1)	38(1)	45(1)	–11(1)	–1(1)	–5(1)

O(63)	29(1)	41(1)	28(1)	–2(1)	–3(1)	4(1)
C(64)	43(1)	59(2)	33(1)	–4(1)	–12(1)	14(1)
C(65)	25(1)	32(1)	52(1)	–8(1)	–1(1)	–5(1)
C(71)	18(1)	34(1)	35(1)	2(1)	–1(1)	–1(1)
C(72)	20(1)	37(1)	31(1)	–1(1)	–3(1)	5(1)
O(73)	28(1)	32(1)	48(1)	–8(1)	5(1)	–2(1)
O(74)	25(1)	52(1)	42(1)	–12(1)	5(1)	3(1)
C(75)	35(1)	66(2)	59(2)	–26(1)	7(1)	7(1)

Table 5. Hydrogen coordinates (× 10^4) and isotropic displacement parameters (Å^2 × 10^3).

	x	y	z	U(eq)
H(12)	5398	3328	5220	41
H(11)	6042	2068	4844	39
H(41)	8099	5993	5677	44
H(51)	8422	9079	5416	49
H(61)	7561	10481	4785	43
H(71)	6361	8753	4387	37
H(152)	3800	7228	4807	63
H(153)	4694	7109	5168	63
H(151)	4742	8272	4674	62
H(211)	3522	4830	4304	37
H(252)	3163	217	3309	76
H(253)	3291	–800	3842	77
H(251)	4195	–230	3526	77

H(512)	8212	3054	6578	34
H(511)	7657	1633	6231	34
H(541)	10447	–788	5863	38
H(551)	10772	–3904	6127	41
H(561)	9871	–5356	6725	38
H(571)	8612	–3643	7099	35
H(652)	6047	–2189	6687	54
H(653)	7019	–3193	6830	54
H(651)	6962	–2052	6335	54
H(711)	5776	269	7202	35
H(752)	5257	5522	7863	81
H(753)	5960	4685	8245	80
H(751)	6357	5646	7776	80
H(141)	6350	5999	3086	74
H(142)	7279	5710	3407	75
H(143)	6688	3911	3202	74
H(641)	8610	–438	8425	66
H(642)	9160	1414	8205	67
H(643)	9509	–717	8094	68

Table 6. Hydrogen bonds [Å and °].

D–H...A	d(D–H)	d(H...A)	d(D...A)	<(DHA)
C(21)–H(211)...O(12)#1	0.93	2.6	3.506(3)	164
C(25)–H(253)...O(12)#2	1	2.57	3.486(3)	152

1.6 References

1. M. E. Dudley, M. M. Morshed and M. M. Hossain, *Synthesis*, 2006, **10**, 1711.
2. P. Bongen, J. Pietruszka and R. C. Simon, *Chem. Eur. J.*, 2012, **18**, 11063.
3. W. Lewis, C. J. Moody, E. Packard, A. T. Murray, G. Jones, D. Hamza and A. Nortcliffe, *Eur. J. Org. Chem.*, 2017, 138.
4. T. B. Poulsen, L. Bernardi, J. Alemá, J. Overgaard and K. A. Jørgensen, *J. Am. Chem. Soc.*, 2007, **129**, 441.
5. B. B. Snider, D. M. Roush, D. J. Rodini, D. Gonzalez and D. Spindell, *J. Org. Chem*., 1980, **45**, 2773.
6. E. J. Corey and J. A. Katzenellenbogen, *J. Am. Chem. Soc.*, 1969, **91**, 1851.
7. J. S. Wzorek, T. F. Knöpfel, I. Sapountzis and D. A. Evans, *Org. Lett.*, 2012, **14**, 5840.
8. E. B. Corcoran, A. B. Williams and R. N. Hanson, *Org. Lett.*, 2012, **14**, 4630.
9. Y. A. Lin, J. M. Chalker, F. Nicola, G. J. L. Bernardes and B. G. Davis, *J. Am. Chem. Soc.*, 2008, **130**, 9642.
10. J-M. Gaudin, *Tetrahedron Lett.*, 1991, **32**, 6113.
11. P. Wipf, C. R. J. Stephenson and M. A. A. Walczak, *Org. Lett.*, 2004, **6**, 3009.
12. J. Lei, A. R. Lippert and J. W. Bode, *J. Am. Chem. Soc.*, 2008, **10**, 4253.
13. H. H. Wasserman and A. K. Petersen, *Tetrahedron Lett.*, 1997, **38**, 953.
14. J. H. Rigby, U. S. M. Maharoof and M. E. Mateo, *J. Am. Chem. Soc.*, 2000, **122**, 6624.
15. Ø. W. Akselsen and L. Skattebøl, *Tetrahedron Lett.*, 2009, **50**, 6339.
16. L. J. Gooßen and A-R. S. Ferwanah, *Synlett*, 2000, **12**, 1801.
17. M-K. Wong, C-W. Yu, W-H. Yuen and D. Yang, *J. Am. Chem. Soc.*, 2000, **66**, 3606.
18. R. A., Valiulin and A. G. Kutateladze, *J. Org. Chem. Soc.*, 2008, **73**, 335.
19. D. W. Manley, R. T. McBurney, P. Miller and J. C. Walton, *J. Org. Chem.* 2014, **79**, 1386.
